# Mitochondrial Akt Signaling Modulated Reprogramming of Somatic Cells

**DOI:** 10.1038/s41598-019-46359-6

**Published:** 2019-07-09

**Authors:** Yu-Han Chen, Ching-Chieh Su, Wu Deng, Leslie F. Lock, Peter J. Donovan, Matthew A. Kayala, Pierre Baldi, Hsiao-Chen Lee, Yumay Chen, Ping H. Wang

**Affiliations:** 1 UC Irvine Diabetes Center, University of California, Irvine, California USA; 2 Sue & Bill Gross Stem Cell Research Center, University of California, Irvine, California USA; 3 Department of Physiology and Biophysics, University of California, Irvine, California USA; 4 Graduate Institute of Applied Science and Engineering and School of Medicine, Fu Jen Catholic University, Taipei, Taiwan; 5 Cardinal Tien Hospital, New Taipei City, Taiwan; 6 Departments of Biological Chemistry, and Developmental & Cell Biology, University of California, Irvine, California USA; 7 Department of Computer Science, University of California, Irvine, California USA; 8 Department of Plastic Surgery, Kaohsiung Medical University, Kaohsiung, Taiwan

**Keywords:** Reprogramming, Cardiovascular biology

## Abstract

The signaling mechanisms controlling somatic cell reprogramming are not fully understood. In this study, we report a novel role for mitochondrial Akt1 signaling that enhanced somatic cell reprogramming efficiency. The role of mitochondrial Akt1 in somatic cell reprogramming was investigated by transducing fibroblasts with the four reprogramming factors (Oct4, Sox2, Klf4, c-Myc) in conjunction with Mito-Akt1, Mito-dnAkt1, or control virus. Mito-Akt1 enhanced reprogramming efficiency whereas Mito-dnAkt1 inhibited reprogramming. The resulting iPSCs formed embryoid bodies *in vitro* and teratomas *in vivo*. Moreover, Oct4 and Nanog promoter methylation was reduced in the iPSCs generated in the presence of Mito-Akt1. Akt1 was activated and translocated into mitochondria after growth factor stimulation in embryonic stem cells (ESCs). To study the effect of mitochondrial Akt in ESCs, a mitochondria-targeting constitutively active Akt1 (Mito-Akt1) was expressed in ESCs. Gene expression profiling showed upregulation of genes that promote stem cell proliferation and survival and down-regulation of genes that promote differentiation. Analysis of cellular respiration indicated similar metabolic profile in the resulting iPSCs and ESCs, suggesting comparable bioenergetics. These findings showed that activation of mitochondrial Akt1 signaling was required during somatic cell reprogramming.

## Introduction

Uncovering the molecular mechanisms underlying somatic cell reprogramming and stem cell differentiation will present new opportunities to modulate cell fate decision and help realize the potential of regenerative medicine^1^. Recent investigations indicate a role for metabolism in the regulation of stem cell differentiation and somatic cell reprogramming^2^. Differentiation of stem cells and reprogramming of somatic cells are accompanied by changes in cell respiration and oxidative phosphorylation^3^. Accumulating evidence suggests a complex cross-talk network that comprises cellular signaling, metabolic pathways, and cell fate specification^4,5^. How growth factor signaling and metabolic regulation modulate reprogramming of induced pluripotent stem cells (iPSCs) is just beginning to unfold.

ESCs are known to depend on glycolysis for cell growth and metabolism^6,7^. Glucose is transported into cells and metabolized to pyruvate. ESC mitochondria respiration is low and an increase in glycolysis is not associated with higher mitochondrial oxidative phosphorylation. The increased glycolysis products in ESCs are diverted to anabolic pathways for cell growth and proliferation rather than the mitochondria oxidative phosphorylation (the Warburg effect)^6^. In addition, reprogramming of fibroblasts into iPSCs was accompanied by an increase in glycolysis before the expression of stemness genes^7,8^, consistent with the high glycolysis state in ESCs. Mitochondria play a pivotal role in the regulation of oxidative phosphorylation and oxidative stress. Inhibition of mitochondrial oxidative phosphorylation complex III increased the stemness of hESCs^9^. A transition from anaerobic glycolysis to aerobic oxidative phosphorylation occurred during ESCs differentiation into specialized cells^10^. These studies suggested that proper state of mitochondrial oxidative metabolism may play a role during specification of cell fate in differentiation and dedifferentiation, however, the mechanism underlying mitochondrial regulation of somatic cell reprogramming is not fully understood.

Akt/PKB is a serine/threonine kinase that was first discovered as an oncogene within the mouse leukemia virus AKT8^11^. Three isoforms comprise the protein kinase B family: Akt1, Akt2, and Akt3. Akt is a downstream target of phosphatidylinositol 3-kinase (PI3K), and mediates diverse signaling cascades regulating cell proliferation, survival, growth, glucose metabolism, cell migration/invasion, genome stability, and angiogenesis^12,13^. In addition to the classical signaling pathways in the cytosolic compartment, recent studies showed that Akt1 can be activated and translocated to the mitochondria^14–16^. Our laboratory had shown that impaired translocation of Akt1 to mitochondria in cardiac muscle led to myocardial metabolic dysfunction^17–20^. Inhibition of cellular Akt signaling negatively modulated ESC pluripotency and self-renewal^21^. Expression of an active Akt1 anchored to the plasma membrane helped maintain the pluripotency of ESC^22^, and increased the efficiency of iPSC induction^23^. However, these previous studies on ESC/iPSC used experimental approaches that modulated global Akt signaling or cytosolic Akt action, and therefore could not address the effect of mitochondrial Akt signaling on somatic cell reprogramming. The aim of this study was to investigate whether mitochondrial Akt1 signaling plays a role in the regulation of somatic cells reprogramming.

## Results

### Mitochondrial Akt1 signaling enhanced somatic cell reprogramming efficiency

**Figure 1:**
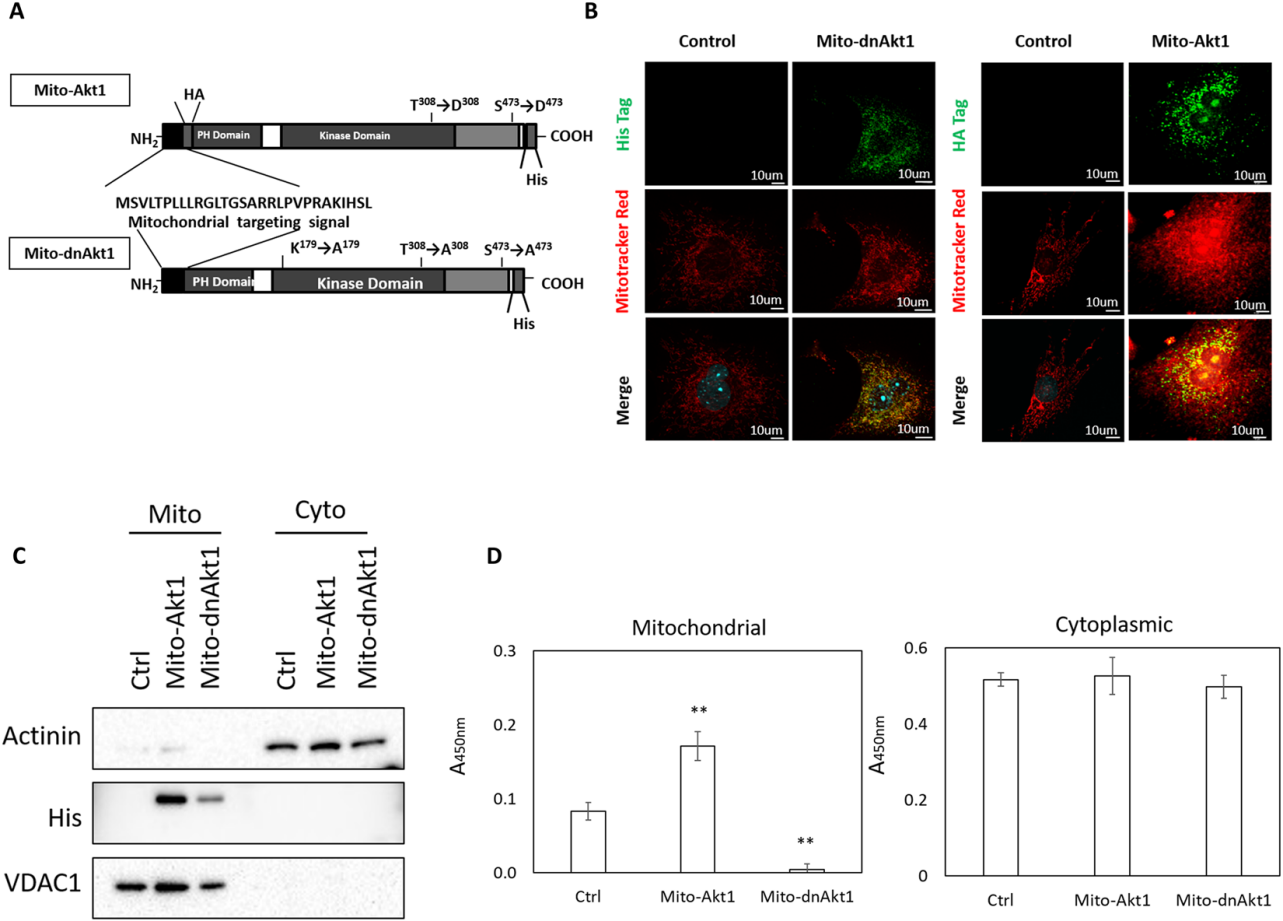
Mitochondria-targeting adenoviral vectors. (**A**) Constructs of adenoviral vectors expressing mitochondria-targeting constitutively active and dominant negative Akt. The constructs were tagged with either HA or His. (**B**) Mitochondria-targeting was achieved in murine fibroblasts (MEF). The cells were transduced with the control adenovirus, Mito-dnAkt1 or Mito-Akt1 and the protein products of transgene was stained with anti-His tag or anti-HA tag antibodies, and mitochondria stained with Mitotracker Red. Scale bar −10 um. (**C**) Distribution of mutant Akt in MEF mitochondria and cytoplasmic fractions. MEFs were transduced with adenoviral vectors and harvested 48 hours post transduction. Mitochondria and cytoplasmic fractions were subfractionated as described in the method section. Both Mito-Akt1 and Mito-dnAkt1 were His-tag labelled. Actinin was used as cytoplasmic marker, while VDAC1 was used as mitochondrial marker. The mutant Akts specifically localized to mitochondria. (**D**) Akt activity assays. Protein lysates from mitochondria (20 ug) and cytoplasmic fractions (60 ug) were used to determine Akt kinase activity assay as described in the Methods section. Bar graph represents the results summarized from 3 independent experiments in duplicates. **p ≤ 0.01.

**Figure 2:**
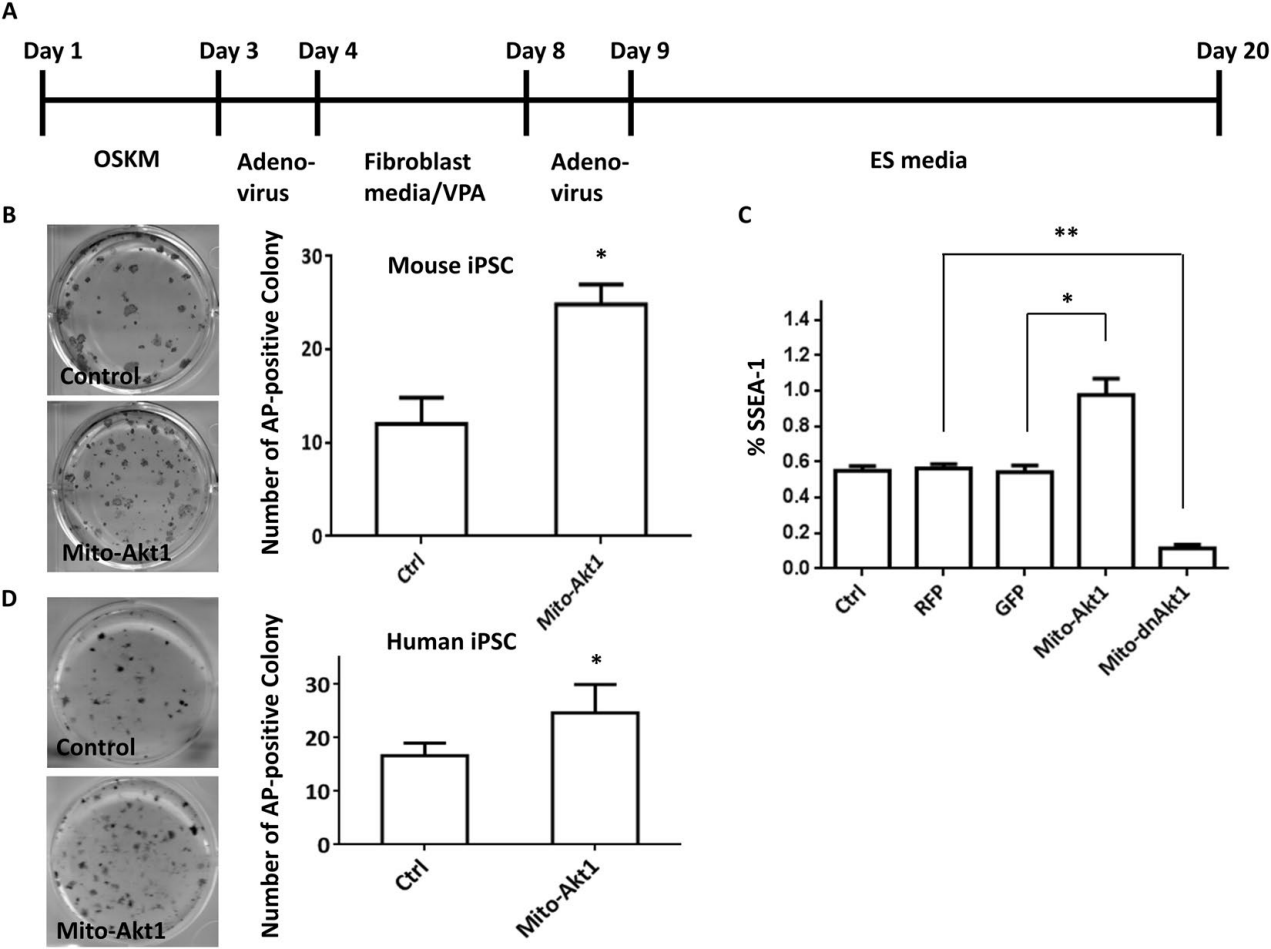
Mitochondrial Akt1 enhanced reprogramming of murine and human fibroblasts. (**A**) The scheme of iPSC induction procedure. O: Oct4. S: SOX2. K: Klf4. M: c-Myc. VPA: valporic acid. Detailed reprogramming protocol is described in the Materials and Methods. (**B**) The number of mouse iPSC colonies was determined by counting the number of alkaline phosphatase-positive colonies on day 20. Photos were taken from a 6 well plate from each group. Representative photo of AP staining is shown here. Bar graph represents the results summarized from 3 independent experiments in triplicates. *p < 0.0001. (**C**) Reprogramming efficiency analyzed by SSEA1 positive cells. Mito-Akt1 significantly increased the number of cells stained positive for SSEA1, while Mito-dnAkt1 reduced SSEA1 staining to background level. Ctrl: control media. RFP: lenti-RFP virus. GFP: Ad-GFP virus. The percentage of SSEA1-positive cell was determined by flow cytometry on day 21. Bar graph represents the results summarized from 3 independent experiments in triplicates. *p < 0.005, **p < 0.0001. (**D**) The number of human iPSC colonies was determined by counting the number of alkaline phosphatase-positive colonies in each well on day 20. Representative photo of AP staining is shown here. Bar graph represents the results summarized from 3 independent experiments in triplicates. *p < 0.01.

The first series of experiments were to study the effect of mitochondrial Akt1 on reprogramming of somatic cells into iPSCs. To this end, we altered mitochondrial Akt1 signaling during reprogramming of somatic cells. Mouse embryonic fibroblasts (MEFs) were infected with retroviral vectors carrying four transcription factors, Oct4, Sox2, Klf4 and c-Myc (OSKM) to induce reprogramming. At the end of reprogramming, presumptive mouse iPSC colonies were selected based on their morphology^24^, and the colonies were further expanded. To define the role of mitochondrial Akt signaling, we used two adenoviral constructs to dissect mitochondria Akt signaling in this study, a dominant negative and a constitutively active Akt (Fig. 1A). Mitochondria targeting of the constructs was achieved by the mitochondria-targeting sequence (Fig. 1B), which was clipped upon entry into mitochondria^25^. We had used this strategy to dissect mitochondrial Akt signaling in various cells in our previous studies, without perturbation of cytosolic Akt signaling^17–20^. To confirm mitochondria-targeting, we subfractionated mitochondria and cytoplasmic fractions and used western blots to analyze the presence of transgene products (Fig. 1C). The results showed that the mutant Akt proteins exclusively localized to mitochondria. To verify the activity of the Akt kinase activities were modulated as we had intended, we also analyzed Akt kinase activities (Fig. 1D), which showed specific modulation of Akt kinase activities in mitochondria without altering cytoplasmic kinase activity. The experimental protocol of reprogramming was outlined in Fig. 2A^26^. Stem cell-like cells appeared around day 12. On day 20, the colonies were fixed and stained for alkaline phosphatase activity (Fig. 2B). More alkaline phosphatase-positive colonies were observed in the MEFs transduced with the OSKM four factors and Mito-Akt1 as compared to MEFs transduced with the four factors and Ad-GFP (Fig. 2B). These results suggested that mitochondrial Akt1 signaling either enhanced reprogramming of somatic cells or increased the transduction efficiency of the four reprogramming factors. To exclude the potential effect of the constitutively active mitochondria-targeting Akt (Mito-Akt1) on enhancing the transduction efficiency of the four factors, Mito-Akt1 and control Ad-GFP were introduced into MEFs stably transduced with a doxycycline-inducible Oct4-Sox2-Klf4-cMyc polycistronic cassette (MEFɛA)^27–29^. Expression of the four factors in MEFɛA was induced with doxycycline and the number of cells that expressed SSEA-1 was determined by flow cytometry. On day 18 after induction with doxycycline, more SSEA-1-positive cells were observed in cells transduced with Mito-Akt1 than Ad-GFP control (Fig. 2C). These results confirmed that activation of mitochondrial Akt signaling during somatic cell reprogramming increased the efficiency of reprogramming. To determine whether mitochondrial Akt signaling was required for 4 factor reprogramming, mitochondria Akt action was inhibited with the mitochondria-targeting dominant negative Akt1 construct (Mito-dnAkt1). Transduction of the MEFɛA cells with Mito-dnAkt1 significantly reduced the number of cells stained positive for SSEA1, thus confirmed the essential role of mitochondrial Akt signaling during reprogramming (Fig. 2C). To determine of the effect of mitochondrial Akt1 signaling on human somatic cell reprogramming, Mito-Akt1 and control adenoviruses were introduced into human dermal fibroblasts simultaneously with the 4 reprogramming factors (Fig. 2D). An increased number of alkaline phosphatase-positive colonies were observed in the human fibroblasts co-transduced with the Mito-Akt1 as compared to the Ad-GFP control suggesting that mitochondrial Akt-1 signaling also enhances reprogramming of human somatic cells.

### Stem cell marker and pluripotency of the iPSCs

**Figure 3:**
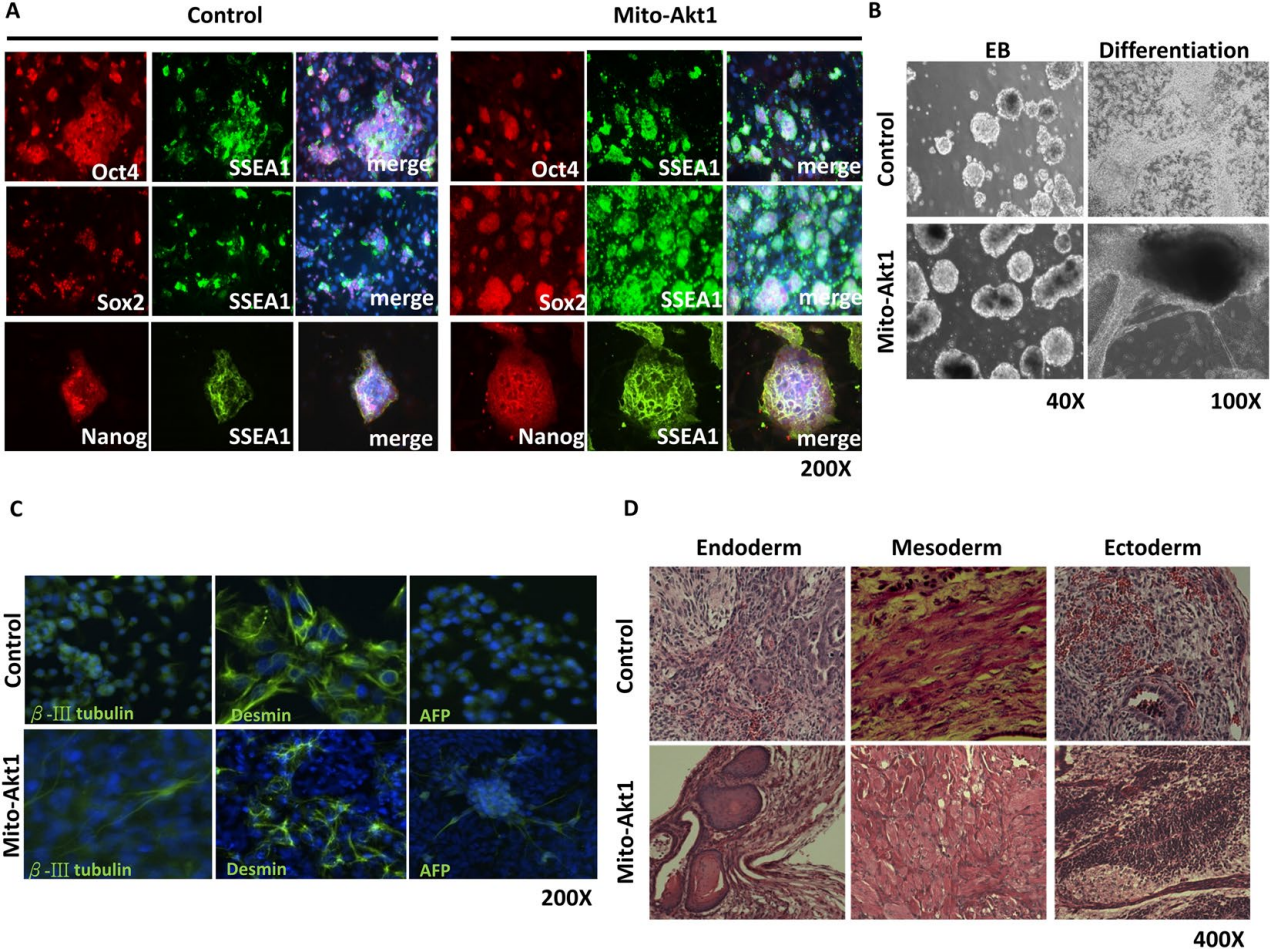
Characterization of iPSC pluripotency. (**A**) The iPSCs expressed embryonic stem cell surface markers. iPSC colonies derived from both groups were stained for SSEA1, Sox2, and Nanog. (**B**) *In vitro* differentiation assay. iPSCs from both groups were subjected to embryoid body formation. Embryoid bodies were plated onto 6 well plates, various cell types emerged from embryoid bodies. (**C**) The resulting tissues from EB were subjected to immunostaining with lineage markers of three germ layers. βIII tubulin for ectoderm, Desmin for mesoderm and α-fetoprotein (AFP) for endoderm. Representative pictures were taken at 200X magnification. (**D**) *In vivo* differentiation assay. iPSC from both groups injected to SCID mice for teratoma formation. After 6 weeks, teratomas were sectioned and stained with Hematoxylin and Eosin. Representative photos of three germ layers are shown.

At passage 8, AP staining was used to re-confirm the presumptive mouse iPSC lines, and cells derived from the same line were expanded, and at passage 10 the cells were used for characterization of pluripotency markers. Mouse iPSCs derived from the 4-factor (Oct4, Sox2, Klf4 and c-Myc) and Mito-Akt1 transduction were morphologically indistinguishable from mouse ESCs (data not shown), both Mito-Akt1 iPSCs and Ad-GFP iPSC colonies were stained positive for Oct4, Sox2, Nanog and SSEA-1 (Fig. 3A). To analyze pluripotency, we carried out embryoid body formation assay *in vitro* and teratoma formation assay *in vivo*. For embryoid body formation, equal number of Mito-Akt1 miPSCs and mESCs were cultured in suspension for 10 days, then allowed to adhere to a tissue culture-treated dish for an additional 5 to 7 days. At day 10, the size of the embryoid bodies was similar in both groups (Fig. 3B). A variety of cell types were observed after 5–7 days in adherent culture and were positive for three germ layers markers. (Fig. 3C). For teratoma formation, an equal number of Mito-Akt1 iPSCs and mESCs were injected into severe combined immunodeficiency (SCID) mice. After 6 weeks, the teratomas that had formed were sectioned, stained with hematoxylin and eosin. Based on cell morphology, the tumors contained cells derived from three primary germ layers (Figs 3D and S2). These studies showed that the iPSCs derived with mitochondrial Akt1 activation during reprogramming were capable of differentiating into endoderm, mesoderm and ectoderm *in vitro* and *in vivo*.

### Methylation of oct4 and nanog promoters in the iPSCs

**Figure 4:**
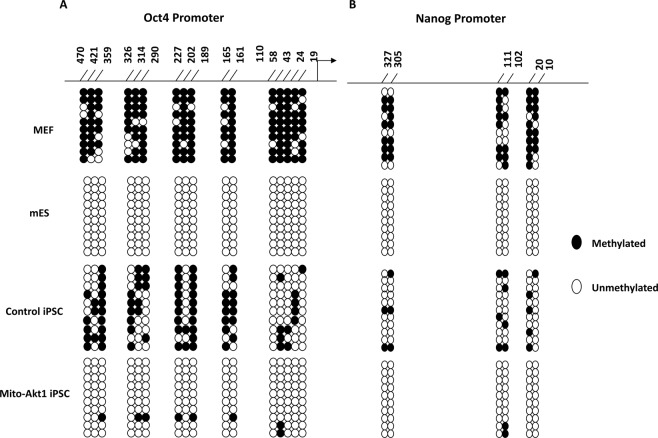
Methylation of Oct4 and Nanog promoters in mouse iPSCs. (**A**) Bisulfite sequencing of the promoter region of Oct4. (**B**) Bisulfite sequencing of the promoter region of Nanog. Genomic DNA were extracted from MEF, mouse ESC and iPSCs for bisulfite sequencing to determine the methylation status of the CpG islets at Oct4 and Nanog promoters. 10 random colonies from each group were used for this assay.

Negative regulation of Oct4 and Nanog promoter methylation had been linked to increased pluripotency^30^. To further characterize Mito-Akt1 iPSCs, we analyzed the methylation profile of Oct4 and Nanog promoters in the Mito-Akt1 iPSCs, mESC, and MEFs (Fig. 4). At passage 10 after reprogramming, mouse iPSC colonies that were positive with AP staining were used for DNA isolation and bisulfite sequencing. Oct4 and Nanog promoters were heavily methylated in MEFs and unmethylated in mESCs, the methylation profile in the iPSCs reprogrammed from Mito-Akt1-transduced fibroblasts is very similar to that for mESCs. Interestingly, iPSCs reprogrammed with the 4 factors in the absence of Mito-Akt1 were more methylated than the iPSCs reprogrammed with the 4 factors in the presence of Mito-Akt1. These data indicate that mitochondrial Akt1 signaling during reprogramming was associated with more profound de-methylation of pluripotency gene promoters in the resulting iPSCs.

### Akt1 is activated and translocated into mitochondria in hESC

**Figure 5:**
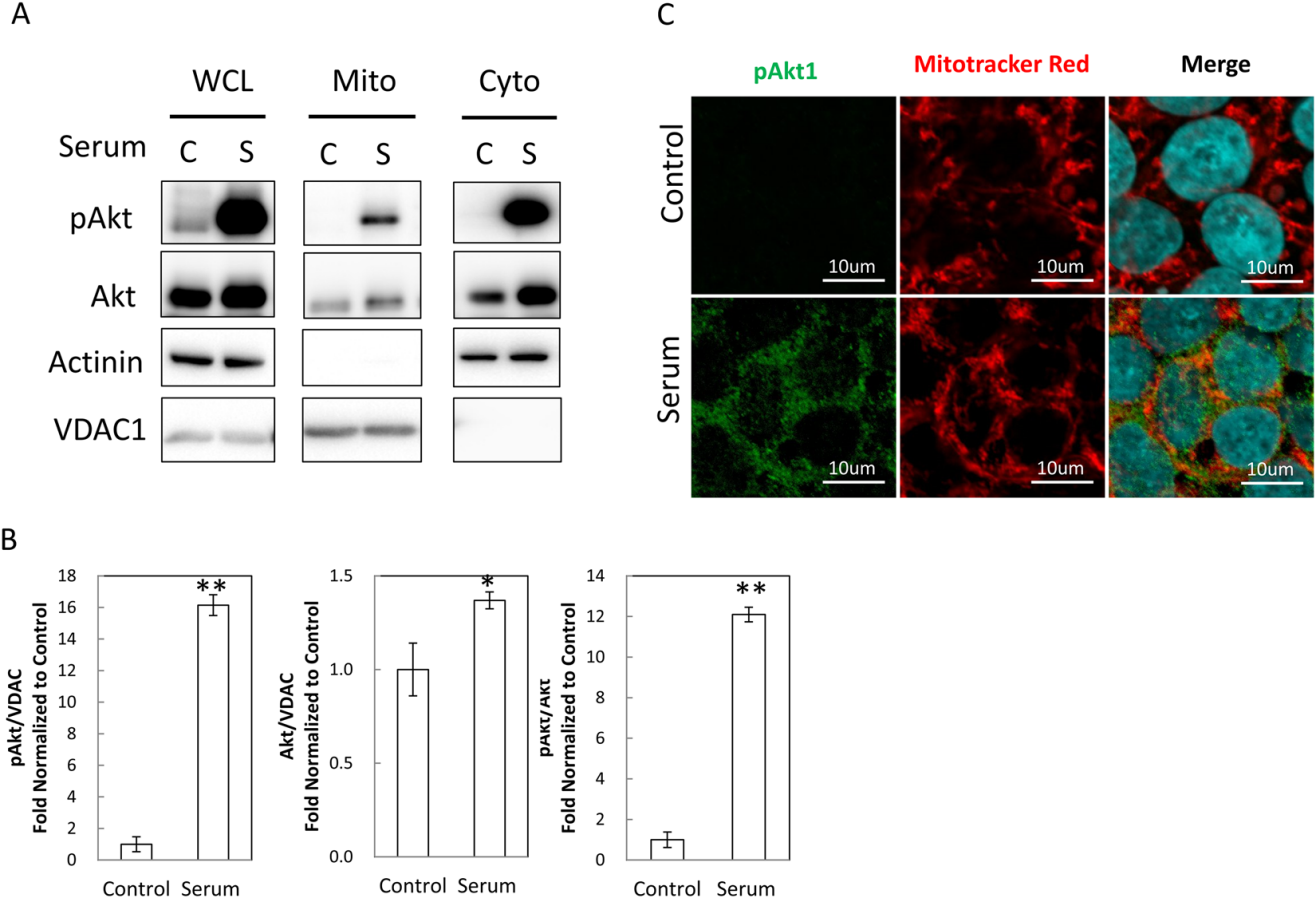
Akt translocation to mitochondria in human embryonic stem cells. (**A**) Akt1 was activated and translocated into mitochondria following growth factor stimulation in H9 hESC. H9 cells were serum deprived with E8 basal medium for 8 hours, stimulated with E8 full medium for 10 min, and collected for mitochondria subfractionation. Whole cell lysate (WCL), mitochondria fraction (Mito), and cytosolic fraction (Cyto) were solubilized and resolved with SDS-PAGE for immunoblots with anti-Akt1, anti-pAkt, anti-Actinin, or anti-VDAC1 antibodies. C: Control. S: Serum stimulation. The presence of VDAC indicated mitochondria fraction. (**B**) Quantitation of pAkt in mitochondria. Western blots from 3–4 independent experiments were analyzed for the content of pAkt, Akt, and pAkt/Akt ratio in hESC in response to growth factor stimulation. The contents of pAkt and Akt were determined by densitometry and normalized with the content of VDAC in each sample. **p < 0.01, *p < 0.05. (**C**) Mitochondrial translocation of pAkt in H9 cells. H9 cells were serum-deprived for 8 hours and then stimulated with full medium for 10 minutes when indicated. The cells were fixed for immunofluorescence study, pAkt1 were stained with anti-pAkt1 antibodies and mitochondria were stained with Mitotracker Red. Significant proportions of pAkt localized to mitochondria upon serum stimulation. Nuclei were stained with DAPI in blue. Scale bar −10 um.

**Figure 6:**
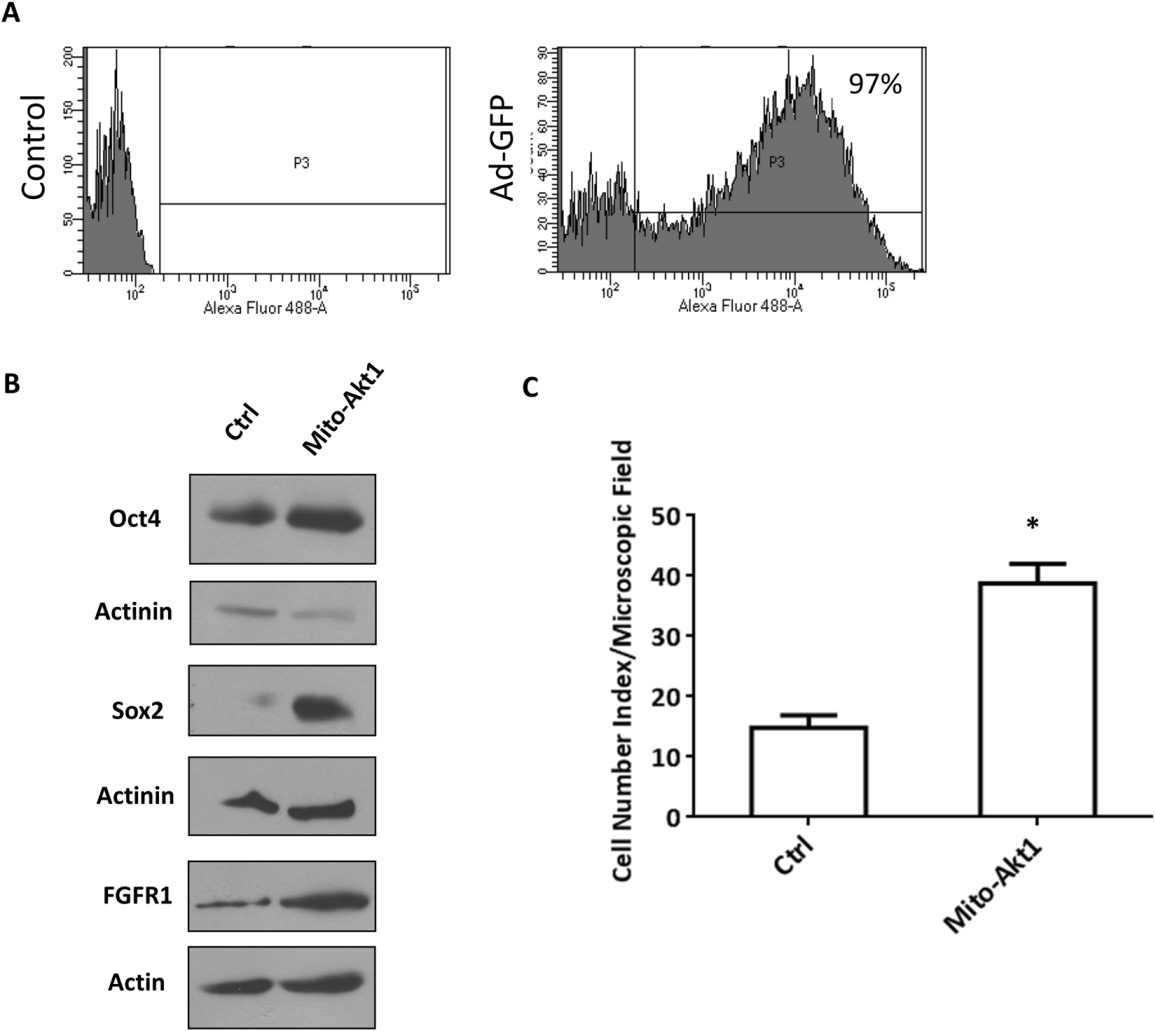
The effect of mitochondrial Akt in human embryonic stem cells. (**A**) Transduction efficiency of adenoviral vector in H9 cells. H9 cells were transduced with Ad-GFP (control) for 72 h. After viral transduction, the cells were washed and analyzed with FACS. 97% of cells were positive for GFP expression. (**B**) Mitochondrial Akt1 enhanced stem cell pluripotency marker expression in hESC. H9 cells were transduced with Mito-Akt1 or Ad-GFP (control) in hES media without bFGF for 72 hours. Protein lysates were resolved with SDS-PAGE and immunoblotted with specific antibodies. The abundance of Oct4, Sox2, and FGFR1 was increased in the cells transduced with Mito-Akt1 (full size western blot image in Supplementary Information), actinin and actin served as loading control. (**C**) Activation of mitochondrial Akt1 increased cell proliferation in hESC. H9 cells were transduced with Mito-Akt1 or control virus for 65 hours and the cell number per microscopic field was counted in 10 random fields on each plate. The bar graph represents the results from 4 independent experiments in triplicates. *P < 0.01.

Akt is a major downstream effector of PI3K. Akt can be phosphorylated at Thr308 by PDK1 and Ser473 by mTORC2. Upon growth factor stimulation, Akt1 was phosphorylated and translocated to mitochondria in human embryonic stem cell (hESC) (Fig. 5). Increased Akt phosphorylation in mitochondria could be attributed to a combination of Akt activation and translocation to mitochondria (Fig. 5B). Confocal microscopy analysis showed that a significant proportion of activated Akt translocated to mitochondria (Fig. 5C). These data indicated that Akt can be translocated to mitochondria and became activated in the human embryonic stem cells. Since mitochondrial Akt positively promoted reprogramming of somatic cells, we speculated that mitochondrial Akt may modulate hESC stemness. We used our adenoviral constructs to study the effect of mitochondrial Akt on hESC gene expression. In H9 hESC, 97% of the cells were successfully transduced with the adenoviral constructs (Fig. 6A).

In order to explore the effect of mitochondria Akt1 signaling on hESCs, we analyzed gene expression profile with the Affimetrix DNA microarray. Genes were defined to be significantly up (or down) regulated if the mean expression is greater (or less) with a BH adjusted Cyber-T p-value < 0.05. Compared to the H9 cells transduced with control Ad-GFP, expression of 209 genes was significantly up-regulated while expression of 578 genes was down-regulated in the hESCs transduced with Mito-Akt1. Over-representation of GO annotations was analyzed with the DAVID tool. A term is considered significantly overrepresented if the BH corrected EASE (adjusted Fisher Exact Test) p-value < 0.05. The most significant annotations for the up and down-regulated genes are listed in Table 1 and the complete gene list can be found in Supplementary Table 1. The up-regulated genes are involved in positive regulation of nucleosome positioning, chromatin assembly/organization, nucleotide synthesis, and cell survival. The down-regulated genes are involved in cell differentiation, and regulation of biological processes of differentiated cells. These results suggest that mitochondrial Akt1 signaling might lead to retention of stem cell attributes in hESCs by promoting expression of genes involved with cell self-renewal and survival, and suppressing genes involved in cell differentiation.

**Table 1:** GO Analysis of the differentially expressed genes in the H9 cells transduced with mitochondria-targeting constitutively active Akt.

*GO BP ID*	*P Value*	*Odds Ratio*	*Function*
**Up-Regulated Genes GO Analysis**			
0016584	0	45	Nucleosome positioning
0009219	0.003	45	Pyrimidine deoxyribonucleotide metabolic process
0008634	0.007	22	Negative regulation of survival gene product expression
0019692	0.007	22	Deoxyribose phosphate metabolic process
0048286	0.007	22	Lung alveolus development
0006334	0	20	Nucleosome assembly
0009948	0.01	18	Anterior/posterior axis specification
0070227	0.01	18	Negative regulation of apoptosis
0006323	0	13	DNA packaging
0006333	0	12	Chromatin assembly or disassembly
0019321	0.02	11	Pentose metabolic process
**Down-Regulated Genes GO Analysis**			
0002645	0.01	33	Positive regulation of tolerance induction
0006568	0.01	33	Tryptophan metabolic process
0008347	0.01	33	Glial cell migration
0030219	0.01	33	Megakaryocyte differentiation
0042572	0.01	33	Retinol metabolic process
0043616	0.01	33	Keratinocyte proliferation
0045060	0.01	33	Negative thymic T cell selection
0045110	0.01	33	Nntermediate filament bundle assembly
0045408	0.01	33	Regulation of interleukin-6 biosynthetic process
0045620	0.01	33	Regulation of lymphocyte differentiation
0046218	0.01	33	Indolalkylamine catabolic process
0060052	0.002	25	Neurofilament cytoskeleton organization
0050772	0.019	16	Positive regulation of axonogenesis

The expression of genes characteristic of pluripotent cells, Oct4, Sox2, and FGFR1, was increased in the hESC transduced with Mito-Akt1 in our microarray study. To confirm that mitochondrial Akt1 signaling increased the protein expression, we analyzed Oct4, Sox2, and FGFR1 proteins in the H9 cells transduced with Mito-Akt1 or Ad-GFP by western blots (Fig. 6B). Oct4, Sox2, and FGFR1 proteins were increased in the cells with Mito-Akt1 as compared to the control cells which were transduced with Ad-GFP, thus confirmed induction of selective pluripotency genes after mitochondrial Akt1 activation. Mitochondrial Akt1 activation increased the number of H9 cells (Fig. 6C), which collaborates the results of GO analysis and supported our hypothesis that Mito-Akt1 promoted self-renewal and survival of hESC.

### Mitochondrial Akt1 modulated cellular bioenergetics

**Figure 7:**
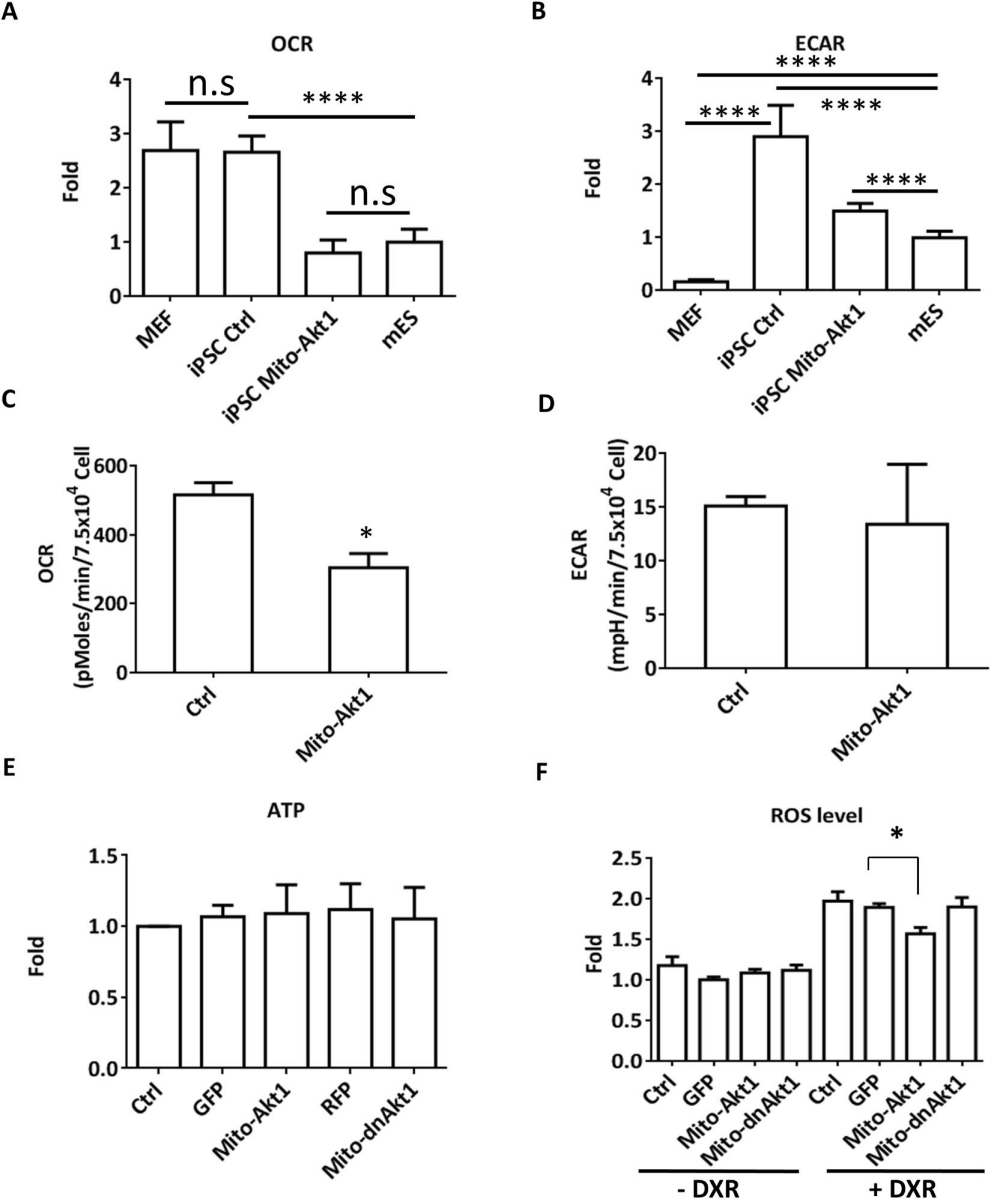
Mitochondrial Akt1 modulated cellular respiration. iPSCs at passage 10 were used for these studies. (**A**) Basal oxygen consumption rate (OCR) in MEF, mESC, and iPSC. (**B**) Basal extracellular acidification rate (ECAR) in MEF, mESC, and iPSC. (**C**) The effect of mitochondrial Akt1 activation on OCR. MEFs were transduced with Ad-Mito-Akt1 (Mito-Akt1) or Ad-GFP (control). 72 hours after transduction, the cells were analyzed with a Seahorse XF24 analyzer. Bar graph represents the results summarized from 3 independent experiments in triplicates. *p < 0.005 (**D**) The effect of mitochondrial Akt1 activation on ECAR in MEF. The cells were transduced with Ad-Mito-Akt1 (Mito-Akt1) or Ad-GFP (control). 72 hours after transduction, cells were studied with a Seahorse XF24 analyzer. (**E**) Cellular ATP contents in MEF. ATP level was quantified by mass spectrometry. (**F**) The effect of Mito-Akt1 on oxidative stress in MEF. Mitochondrial ROS was analyzed with MitoSOX Red and quantified by flow cytometry. Bar graph represents the results summarized from 3 independent experiments in triplicates. *p < 0.005. DXR: Stress induction with doxorubicin.

Since somatic cell reprogramming is associated with changes of cellular respiration, we evaluated cellular bioenergetics with a Seahorse analyzer. The oxygen consumption rate (OCR) represents measurements of oxidative respiration and extracellular acidification rate (ECAR) represents magnitudes of glycolysis (Fig. 7). The respiration profile of the Mito-Akt1 miPSCs was nearly identical to the mESCs, whereas the OCR of the control miPSCs was similar to that of MEFs (Fig. 7A). In contrast, the gylcolysis profile (ECAR) of both Mito-Akt1 miPSCs and control miPSCs was similar to mESCs and differed from that of MEFs (Fig. 7B). MEFs had lower ECAR and higher OCR than the mESCs. These findings suggest activation of mitochondrial Akt1 pathway during reprogramming helped generate a miPSC respiration state comparable to mESC.

Since more efficient respiration could have helped MEFs tolerate the stress during reprogramming, we also investigated the effect of mitochondrial Akt1 signaling on cellular respiration in MEFs (Fig. 7C,D). Although Mito-Akt1 did not alter ECAR, it significantly reduced OCR. Since the ATP levels in these cells were not altered (Fig. 7E), the reduction of OCR in Mito-Akt1 iPSCs and mESCs suggests tightly coupled oxidative phosphorylation. In unstressed MEFs the levels of mitochondria ROS were the same in all groups, and mitochondrial Akt1 did not change ROS levels. When the MEFs were under stress, mitochondria ROS was lowered in the MEFs transduced with Mito-Akt1 (Fig. 7F). It is possible that activation of mitochondrial Akt1 reduced mitochondrial oxidative stress in MEFs during reprogramming.

## Discussion

Gurdon *et al*. demonstrated the basic concept that somatic cells could be reprogrammed to pluripotency in 1958^31^. The reprogramming factors were defined by Takahashi and Yamanaka in 2006. Using the four factors, Oct4, Sox2, Klf4, and c-Myc, iPSCs were recreated from somatic cells^26^. Subsequently, various laboratories developed different protocols and vehicles to produce iPSCs by genetically manipulating critical transcription factors or with small molecule chemical compounds. However, the exact signaling network underlying reprogramming remains elusive and the efficiency of reprogramming has been relatively low. Our study indicates that mitochondrial Akt1 is involved in the signaling network regulating somatic cell reprogramming.

In our DNA microarray analysis, the top ranking genes positively modulated by activation of mitochondrial Akt1 in hESCs were those regulating nucleosome positioning and chromatin organization. Chromatin remodeling through changing nucleosome positioning and histone modification is a critical mechanism that modulates gene transcription. Nucleosome positioning and DNA methylation are important components of epigenetic regulation of transcription initiation and gene expression. While the pattern of nucleosome positioning and regulators of positioning are just beginning to be recognized, its role in cell fate decision in ESCs and iPSCs are not fully understood. Recent studies suggested that nucleosome occupancy correlated with histone modification and the length of nucleosome occupancy increased with differentiation^32^. Our laboratory have completed a map of genome-wide nucleosome positioning in hESC (http://www.dtd.nlm.nih.gov/geo/query/acc.cgi?acc=GSE49140)^33^, future studies may reveal the mechanisms through which growth factor signaling such as Akt1 modulates nucleosome organization and cell fate specification.

ESCs and iPSCs shared significant similarity in pluripotency. But there are distinctive differences in epigenetic signatures between iPSCs and ESCs. Unique pattern of DNA methylation in iPSCs has been reported when compared to ECSs^34–38^, and aberrant DNA methylation in iPSCs showed striking resemblance to cancer cells^39,40^. The results of our control iPSC methylation data are consistent with these literatures. Epigenetic regulation not only played an important role in the reprogramming process, but also affected the quality of iPSCs^41^. The findings in this paper suggest that activation of mitochondrial Akt1 signaling modulated mitochondria respiration during somatic cell reprogramming, increased the efficiency of reprogramming, and enhanced demethylation of Oct4 and Nanog promoters in the resulting iPSCs.

Regulation of stem cell differentiation and reprogramming falls into two major mechanisms, intrinsic and extrinsic^3^. The intrinsic mechanisms included transcription factors and epigenetic factors, while the extrinsic mechanisms include local environment and growth factor signaling. Recent studies have begun to investigate the complex interplay between intrinsic and extrinsic factors^3^. Mitochondrial Akt1 regulation of cellular oxidative phosphorylation, ROS, and cell survival was independent of cytosolic Akt1 and nuclear Akt1^18^. Stem cells have lower mitochondrial oxidative phosphorylation and oxidative stress than the differentiated cells^7^. In terminally differentiated cells, glycolysis decreases while mitochondria respiration increases. Reprogramming of fibroblasts into iPSC is accompanied by increased glycolysis and reduced oxidative phosphorylation. However, to support active cell division, embryonic stem cells rely on glycolysis for anabolic biosynthesis, i.e. the classical Warburg effect. Only part of the pyruvate generated from glycolysis enters mitochondria and TCA cycle for oxidative phosphorylation^6^. Recent studies have shown that certain mitochondria metabolites, such as 2-hydroxyglutarate and FAD, modulated DNA and histone methylation and thereby regulated gene transcription^42,43^. Therefore, alteration of metabolism may play a role in modulating gene transcription during the interplay of extrinsic growth factor singling and intrinsic epigenetic mechanism of reprogramming. Mitochondrial Akt1 signaling lowered oxygen consumption without affecting ECAR and ATP production in MEF, which suggested tighter coupling of oxidative phosphorylation. Although we could not assess how mitochondrial Akt1 signaling affected respiration during reprogramming in each individual cell as it was not possible to identify the specific cell that would reprogram, we had compared the changes of cell respiration before and after reprogramming and confirmed reduction of mitochondria respiration (OCR) and increase of glycolysis (ECAR) in both iPSCs and ESCs.

Akt is the main downstream effector of PI3K signaling pathway^44^. The activated Akt interacts with different proteins at various subcellular localizations to exert concerted biological actions. PI3K/Akt pathway positively regulates embryonic stem cell self-renewal and inhibition of PI3K led to differentiation of hESC^21,45,46^. Convergence of Wnt and Akt pathways could alter lineage determination in stem cells^47^. Activation of Akt signaling increased the pro-stemness Nanog expression in hESCs^48^. These observations suggested that Akt signaling was involved in the extrinsic mechanisms modulating cell renewal and cell fate determination in stem cells. Only small fractions of somatic cells could be successfully reprogrammed into pluripotent stem cells. Current methods can not reliably measure Akt activities specifically in those cells that will be reprogrammed at the end of experiment, therefore we do not know how Akt activities change during reprogramming process. Previous studies on PI3K/Akt pathway used tools that altered global Akt signaling in stem cells, whereas our study focused on the effect of mitochondrial Akt1 signaling without changing cytosolic Akt actions. Our group and other laboratories identified Akt translocation to mitochondria as a key component of Akt signaling in cells^14–20^. Akt family has three members Akt1, Akt2 and Akt3, but only Akt1 can be translocated to mitochondria^49^. When we inhibited mitochondrial Akt1 signaling with the mitochondria-targeting dominant negative construct, fibroblasts failed to reprogram with the four factors. Therefore, activation of mitochondrial Akt1 signaling is required for somatic cell reprogramming. In our study, we used adenovirus construct Mito-Akt1 in the reprogramming experiments. Type 5 Adenovirus vectors allowed transient and efficient expression of mitochondria-targeting Akt1 during reprogramming without disrupting nucleus genome.

Understanding the mechanisms underlying the signaling network that modulates cell metabolism and epigenetic regulation of gene transcription may help identify new opportunities that can be used to develop new strategy to control somatic cell reprogramming and stem cell differentiation. To this end, our findings suggest that mitochondrial Akt1 signaling is a piece of the puzzle that regulates the cell fate specification process.

## Methods

### Reagents

Knockout serum replacement (KOSR), Knockout DMEM/F-12 medium, fetal bovine serum (FBS), Glutamax, non-essential amino acids, Dulbecco’s modified eagle medium-GlutaMax, β-mercaptoethanol, basic fibroblast growth factors (bFGF), leukemia inhibitory factor (LIF),collagenase IV, 0.25% trypsin/EDTA, MitoTracker®, Florophore 2^nd^ antibodies, were purchased from Invitrogen (Carlsbad, CA, USA). Rho kinase (ROCK) inhibitor was from Calbiochem (Gibbstown, NJ, USA). Immobilon-P membranes were from Millipore Co. (Bedford, MA). Anti-SRY(sex determining region Y)-box 2(SOX2), anti-Oct3/4, anti-Klf4, anti-cMyc, anti-Nanog, anti-SSEA1, anti-beta tubulin 3, anti-alpha-fetoprotein, anti-desmin were purchased from GeneTex Inc. (Irvine, CA). Anti-β actin, anti-α-actinin anti-phospho-Akt1 and anti-Akt antibodies were purchased from Santa Cruz Biolabs (Santa Cruz, CA). Anti-porin antibody was purchased from MitoSciences (Eugene, Oregon, CA). Antio-HA tag, anti-His tag antibodies were purchased from Cell Signaling Technology. Peroxidase-conjugated 2nd antibodies were purchased from Santa Cruz Biolabs (Santa Cruz, CA) or GeneTex Inc. (Irvine, CA). RNeasy kit was from QIAGEN (Valencia, CA, USA). Human gene 1.0 ST microarray was purchased from Affymetrix (Santa Clara, CA, USA). All other chemicals were purchased from Sigma or Fisher Scientific. H9 hESCs were obtained from WiCell (Madison, WI) and human fibroblasts were from ScienCell Research Laboratories (Carlsbad, CA).

### Cell culture and transduction of viral constructs

Undifferentiated hESC H9 line were maintained on mouse embryo fibroblast feeder layer in DMEM/F12 supplemented with 20% (vol/vol) KnockOut serum replacement, 0.1 mM nonessential amino acids, 2 mM Glutamax, 0.1 mM 2-mercaptoethanol, and 4 ng/ml bFGF. Cultures were passaged with collagenase IV at a 1:2–1:4 split ratio every 5–7 days.

For those experiments for Akt activation/phosphorylation, E8 medium was used for hESC culture and maintained in pluripotent state. E8 full medium were removed from human ES cells and washed twice with PBS. hESCs were then incubated in E8 basal medium (no supplement) at 37 °C for 15 minutes. After washing, hESCs were incubated in E8 basal medium at 37 °C for 8 hours for serum deprivation. Thereafter, hESCs were stimulated with E8 full medium at 37 °C for 10 minutes and collected for western blot experiment to study mitochondrial translocation of Akt. When indicated, hESCs were transduced with adenoviral vectors. The experimental procedure and protocol were approved by the Human Stem Cell Research Oversight Committee at the University of California, Irvine.

Mouse embryonic fibroblasts (MEF) were isolated from E13.5 embryos by standard isolation procedures. Mouse embryonic stem cells were derived from C57BL/6 mice. MEF (MEFɛA) transgenic for the reprogramming factors were a kind gift from Dr. Konrad Hochedlinger (Boston, MA). MEF were cultured in Dulbecco’s Modified Eagle Medium (DMEM) containing 10% fetal bovine serum (FBS), 1% non-essential amino acid (NEAA), and 1% sodium pyruvate (MEF media). Mouse embryonic/induced pluripotent stem cells were cultured in DMEM containing 15% FBS, 1% NEAA, 1% sodium pyruvate and 55 μM β-mercaptoethanol, supplemented with 10 ng/ml leukemia inhibitory factor (LIF) (ES media). All cells were incubated at 37 °C, 5% CO2. Constitutively active Akt1 was created by mutating Thr308 and Ser473 to aspartic acid residues, which mimics phosphorylation. Mitochondrial targeting was achieved by fusing a mitochondria targeting sequence (MSVLTPLLLRGLTGSARRLPV PRAKIHSL) to the N-terminus as we reported earlier^17–20^. The fused construct was subcloned into an adenoviral vector (Mito-Akt1). A His-tagged dominant negative Akt1 (substitutions at K179A, T308A, and S473A) with the mitochondria targeting sequence at the N-terminus was subcloned into the adenoviral vector (Mito-dnAkt1)^17–20^. Human fibroblasts were cultured in Dulbecco’s Modified Eagle Medium (DMEM) containing 10% fetal bovine serum (FBS), 1% non-essential amino acid (NEAA), and 1% sodium pyruvate.

### Gene expression profiling and gene ontology annotation

Total RNA was extracted and the quality of each RNA sample was assessed with the Agilent Bioanalyzer 2100. Each RNA sample used for this experiment was pooled from 3 independent cell preparations, and all RNA samples used in this study passed quality assurance from electrophoresis. Three RNA samples were included in each experimental group. Double-stranded cDNA was generated for *in vitro* transcription reactions to produce labeled cRNA for hybridization onto the GeneChip Human Genome U133A 2.0 Array (Affymetrix, Santa Clara, Calif) at the University of California, Irvine Genomics High-Throughput Facility as we previously reported^50^. All analysis was performed using the R statistical environment (http://www.r-project.org). Microarray data was normalized and probeset summarized using the ‘affy’ and ‘vsn’^51^ packages of the Bioconductor suite (http://www.bioconductor.org). Then, non-specific filtering was applied to limit the size of the dataset and improve statistical power in differential expression analysis^52^. Datasets not mapping to an Entrez ID were removed. Then, Entrez ID representation was de-duplicated by retaining only the probeset with the highest interquartile range (IQR) for each Entrez ID. Finally, 50% of the remaining datasets with the lowest IQR were removed.

Differential expression was determined using the Bayes-regularized t-test CyberT (p < 0.05) (http://cybert.ics.uci.edu/)^53,54^. The Benjamini and Hochberg method (BH) was used to control the false discovery rate. NIAID’s Database for Annotation, Visualization and Integrated Discovery (DAVID) tool (http://david.abcc.ncifcrf.gov/) was used to cluster and analyze over over-represented Gene Ontology annotations from the GO FAT term set^55^. Only the non-specifically filtered data were used as the background for enrichment analysis. Unsupervised hierarchical clustering was performed in the R environment.

### Reprogramming of fibroblasts

Plasmid DNA encoding the Yamanaka factors were purchased from Addgene and plasmid DNA were prepared using QIAGEN Plasmid Midi Kit. Retroviruses carrying Yamanaka factors were produced according to the original literature^26^. For retroviral vector reprogramming, 5 × 10^4^/well P3-6 MEF were plated on 6 well plate on day 0. Retroviral vectors carrying Oct4, Sox2, Klf4 and c-Myc were spinoculated at 1200 g, 120 minutes, room temperature on day 1 and day 2. Adenoviral vectors carrying either Mito-Akt1 or GFP (control) were added on day 3. From day 4–7, cells were incubated with media supplemented with 2 mM valporic acid. Adenoviral vectors carrying either Mito-Akt1 or GFP (control) were added again on day 8. Cells were trypsinized and counted, equal number of cells was plated into each well of a 6 well plate or 10 cm dish of feeders on day 9. From day 9 to 20, cells were maintained in ES media. For doxycycline-induced reprogramming, P3-6 MEFɛA carrying polycistronic cassette expressing Oct4, Sox2, Klf4 and c-Myc were plated at 5 × 10^4^ cell/well on 6 well-plate on day 0. Four factors expression was induced with 2 μg/ml doxycycline in mouse ES media throughout the whole procedure as described previously^27–29^. Adenoviral vectors carrying either Mito-Akt1 or GFP (control) were added on day 2 and day 6. Cells were trypsinized and counted, and equal number of cells was plated into each well of a 6 well plate or 10 cm dish of feeders on day 7. From day 7 to 20, cells were maintained in mouse ES media.

### Alkaline phosphatase and immunofluorescence staining

For alkaline phosphatase (AP) staining, paraformaldehyde-fixed cells were rinsed with deionized water and stained with FastRed/Napthol or nitroblue tetrazolium/ 5-Bromo-4-chloro-3-indolyl phosphate (NBT/BCIP). For immunofluorescence staining, paraformaldehyde-fixed cells were rinsed with PBS and blocked with 10% normal goat serum. After blocking, cells were incubated with indicated primary antibodies overnight at 4 °C, followed by PBS wash and 1 hour incubation with conjugated secondary antibodies, counter-stained with DAPI, and analyzed with an Eclipse Ti fluorescence microscope (Nikon). To visualize the effect of insulin on Akt1 subcellular localization, MEFs were fixed with 4% formaldehyde for 30 min at room temperature. After washing with PBS, cells were treated with 0.05% saponin in ddH_2_O for 20 min and blocked with 10% normal sera for 30 min. The fixed cells were incubated with specific primary antibodies overnight at 4 °C, conjugated secondary antibodies for 1 hour, counter-stained with DAPI, and analyzed with Eclipse Ti fluorescence microscope (Nikon)^19^.

### Mitochondria preparation and subfractionation

Mitochondria fractionation procedure was modified from the previous studies^17–20,56^. Briefly, cells were harvested 48 hours post transduction and subjected to mitochondria isolation using a mitochondria isolation kit according to the manufacturer’s instruction (Miltenyi Biotec #130-096-946). Briefly, cells were homogenized with 20 strokes of loose pestle and 50 strokes of tight pestle in a lysis buffer supplemented with protease inhibitors cocktails (Sigma Aldrich #S8830) and phosphatase inhibitors (20 mM NaF, and 2 mM Na_3_VO_4_). Homogenates were centrifuged at 1000 g for 10 minutes at 4 °C to remove nucleus and unbroken cells. The supernatants were incubated with Anti-TOM22-conjugating MicroBeads, the magnetically labeled mitochondria were isolated by magnet and column. The flow through containing cytoplasmic fraction was collected and concentrated by a centrifugal filter (Millipore #UFC500396).

### Akt kinase assay

Identical amount of protein lysates isolated from the mitochondrial and cytoplasmic fractions were used for kinase assay, the protein contents were quantified by the Bradford method. 20 µg of mitochondrial lysates and 60 µg of cytoplasmic lysates were analyzed with a Akt kinase assay kit (Abcam #ab139436) following the manufacturer’s instructions.

### Western blots

Western blots method was modified from the previous studies^17–20^. Briefly, equal amounts of proteins from each sample were separated by SDS-PAGE and transferred to polyvinylidene difluoride membrane, and incubated with a blocking buffer (3% BSA in 20 mM Tris–HCl [pH7.5], 137 mM NaCl, and 0.1% Tween 20) for 1 hour at room temperature. The membranes were incubated sequentially with primary antibodies overnight at 4 °C, washed three times with TBS-T (20 mM Tris–HCl [pH7.5], 137 mM NaCl, and 0.1% Tween 20), incubated with respective horseradish peroxidase-conjugated secondary antibodies (1:5000 to 1:20,000 dilution in TBS-T), washed three times with TBS-T, and then incubated with West Pico Chemiluminescent Substrate to visualize the proteins (Thermo Scientific, Pittsburgh, PA).

### *In vitro* and *In vivo* differentiation assay

For *in vitro* differentiation assay, induced pluripotent stem cells were trypsinized into cell chunks and grown in suspension culture in low-attachment plates for 10 days. This was followed by plating into 6-well plates or 10 cm dishes and grown for another 5–7 days. iPSC-derived cells were then fixed and stained with the indicated primary antibodies. For *in vivo* differentiation assay, 1 × 10^6^ of iPSC from different groups were injected into severe combined immunodeficiency (SCID) mice (Charles River). Teratomas were formed after 4–6 weeks.

The animal experimental procedures were approved by the Institutional Animal Care and Use Committee at University of California, Irvine.

### DNA methylation

Genomic DNA was extracted and purified using the Wizard Genomic DNA Purification Kit (Promega) according to the manufacturer’s instruction. Bisulfite conversion of DNA was performed using the EZ DNA Methylation Kit (Zymo Research). For maximal conversion, 500 ng genomic DNA was used for bisulfite reaction. Converted DNA was amplified by PCR using primers that are specific for the promoter region of OCT4 and NANOG. Each 50 µl PCR reaction mix contained 3 μl of bisulfite-treated DNA, 200 nM of forward and reverse primers, 200 µM of dNTP, and 0.5 unit Taq DNA polymerase and PCR buffer (Invitrogen). PCR was performed under the following conditions: the initial denaturation of 10 min at 95 °C, followed by 35 cycles of 1 min at 95 °C, 30 sec at 55 °C, and 30 sec at 72 °C; and the final extension of 10 min at 72 °C. The PCR products were extracted from the gel and purified using QIAquick Gel Extraction Kit (Qiagen). The purified PCR products were subcloned using CloneJET PCR Cloning Kit (Thermo Sientific). For each region, 10 clones were randomly picked and the plasmid DNA was prepared with QuickLyse Miniprep Kit (Qiagen) for DNA sequencing (GENEWIZ).

### Analysis of mitochondrial O_2_ respiration by extracellular flux measurement

Cellular respiration analysis was performed according to the previous studies^17–20,57^. To measure mitochondrial function in cells, we employed a Seahorse Bioscience XF24 Extracellular Flux Analyzer (Seahorse Bioscience, North Billerica, MA) and follow the manufacturer’s protocol. Briefly, cells were plated in a 0.2% gelatin or Matrigel coated 24-well Seahorse XF-24 assay plate at 7.5 × 10^4^ cells/well, and grown for 16 hrs before analysis. On the day of metabolic flux analysis, cells were washed once with freshly prepared KHB buffer (111 mM NaCl, 4.7 mM KCl, 2 mM MgSO_4_, 1.2 mM Na_2_HPO_4_, 2.5 mM glucose and 0.5 mM carnitine; pH 7.4) and incubated in KHB buffer at 37 °C in a non-CO_2_ incubator for 1 hr. Three baseline measurements of oxygen consumption rate (OCR) were taken before sequential injection of following mitochondrial inhibitors and final concentration: oligomycin (1 μg/ml), carbonilcyanide p-triflouromethoxyphenylhydrazone (FCCP) (3 µM) and rotenone (0.1 µM). Three measurements were taken after addition of each inhibitor. OCR values were automatically calculated and recorded by the Seahorse XF-24 software. The basal respiration was calculated by averaging the three measurements of OCR before injection of inhibitors.

### FACS analysis

To analyze GFP expression in embryonic stem cells, the cells were infected with the adenoviral construct for 72 hours, washed twice with PBS and dissociated with 0.05% trypsin. The cell pellets were resuspended in PBS with 3%BSA and analyzed with BD LSR II Flow Cytometer. The fluorescence was measured with FITC setting (excitation at 488 nm and emission at 530 nm). The data was analyzed with the BD FACSDiVa software.

### Statistical analysis

Data are presented as mean ± SD, unless noted otherwise. FACS data were analyzed with BD FACSDiVa software. Student’s t test and one-way repeated measures ANOVA with Holm–Sidak method were performed with SigmaStat 3.11. The statistical significance level was set at p < 0.05.

### Ethics and regulatory statement

All experiments were done in accordance with the standards of the US federal and state guidelines and regulations. The experimental procedure and protocol were approved by the Human Stem Cell Research Oversight Committee at the University of California, Irvine. The animal experimental procedures were approved by the Institutional Animal Care and Use Committee at University of California, Irvine.

## Supplementary information

Supplementary information
