# Supplementary material for: Mitochondrial Akt Signaling Modulated Reprogramming of Somatic Cells
Source: Sci Rep. 2019 Jul 9;9:9919. doi: 10.1038/s41598-019-46359-6 (PMC6616364; doi:10.1038/s41598-019-46359-6)
Supplement: Supplementary file 1 — Supplementary information [file 41598_2019_46359_MOESM1_ESM.pdf]

Mitochondrial Akt Signaling Modulated Reprogramming of Somatic Cells

Supplementary Information

Yu-Han Chen[1,2,3], Ching-Chieh Su[1,4], Wu Deng[1,2], Leslie F. Lock[2,5], Peter J. Donovan[1,2,5], Matthew A Kayala[6], Pierre Baldi[6], Hsiao-Chen Lee[1,2,7] , Yumay Chen[1,2], Ping H. Wang[1,2,3]

1. UC Irvine Diabetes Center, University of California, Irvine, California
2. Sue & Bill Gross Stem Cell Research Center, University of California, Irvine, California,
3. Department of Physiology and Biophysics, University of California, Irvine, California
4. Graduate Institute of Applied Science and Engineering and School of Medicine, Fu Jen Catholic University, Taipei, Taiwan, and Cardinal Tien Hospital, New Taipei City, Taiwan
5. Departments of Biological Chemistry, and Developmental & Cell Biology, University of California, Irvine, California
6. Department of Computer Science, University of California, Irvine, California
7. Department of Plastic Surgery, Kaohsiung Medical University, Kaohsiung, Taiwan

Figure S1. Mito-Akt1 and Mito-dnAkt1 exclusively expressed in mitochondria. Mouse embryonic fibroblasts (MEF) were transduced with adenoviral vector with GFP (Ctrl), Mito-Akt1 or Mito-dnAkt1. Cells were harvested 48 hours post transduction. Mitochondria and cytoplasmic fractions were isolated as described in the method section. Both Mito-Akt1 and Mito-dnAkt1 were His-tag labelled. Actinin was used as cytoplasmic marker, while VDAC1 was used as mitochondrial marker. The original blots used to compile the data in Figure 1C.

Figure S1 (Fig 1 Supplementary Information)

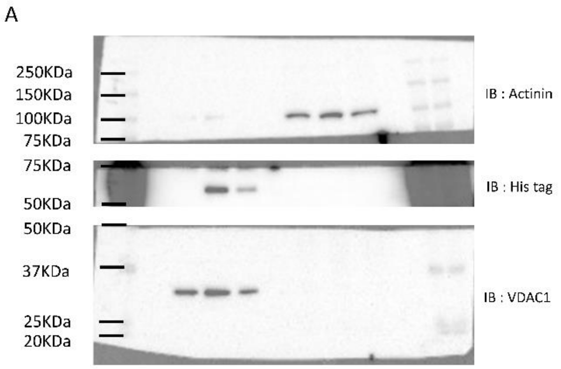

Figure S2. In vivo differentiation of mouse iPSCs (control and Mito-Akt1). Additional photos of teratoma HE stains.

Figure S2 (Fig 3 Supplementary Information)

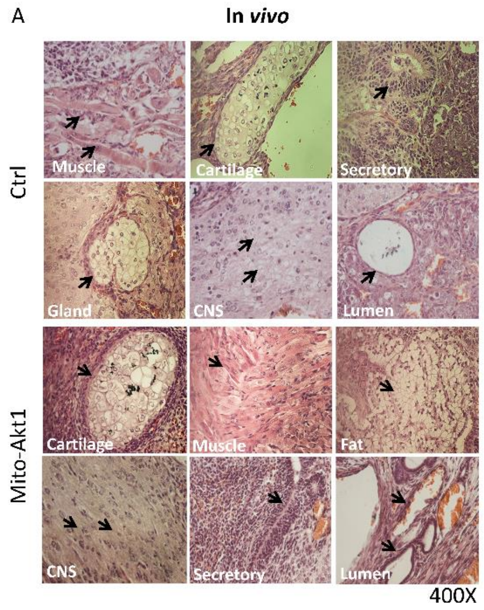

Figure S3. Akt translocation to mitochondria in human embryonic stem cells. The original blots used to compile Fig 5A.

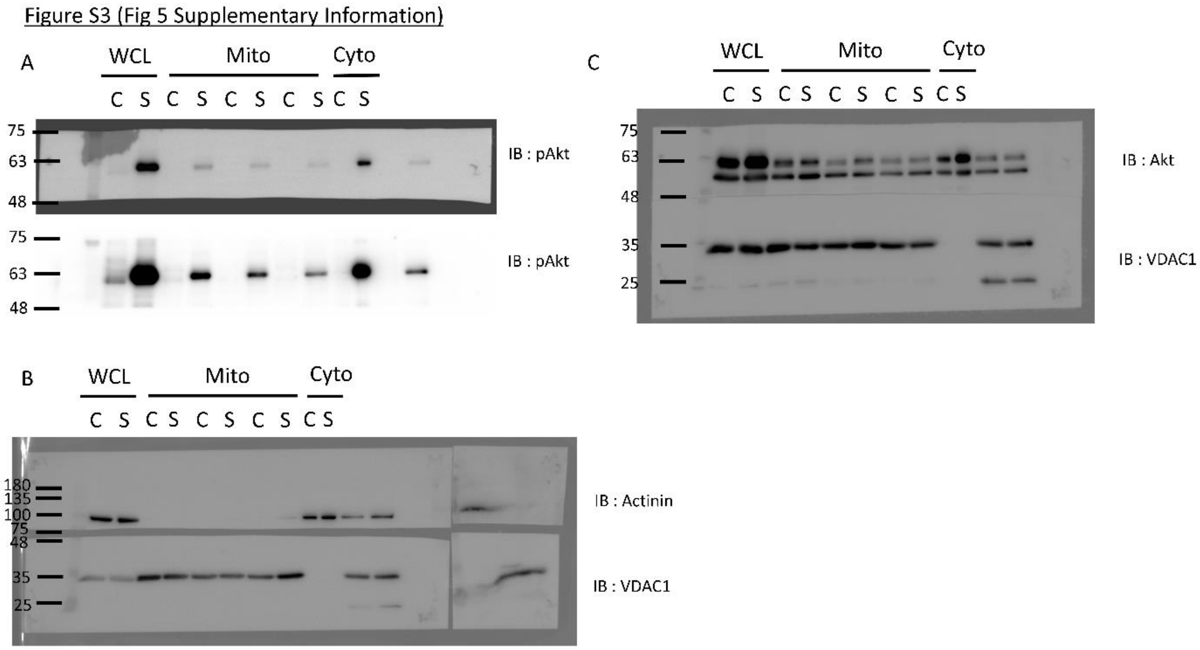

Figure S4. The effects of mitochondrial Akt in human embryonic stem cells. The original blots used to compile Fig 6B.

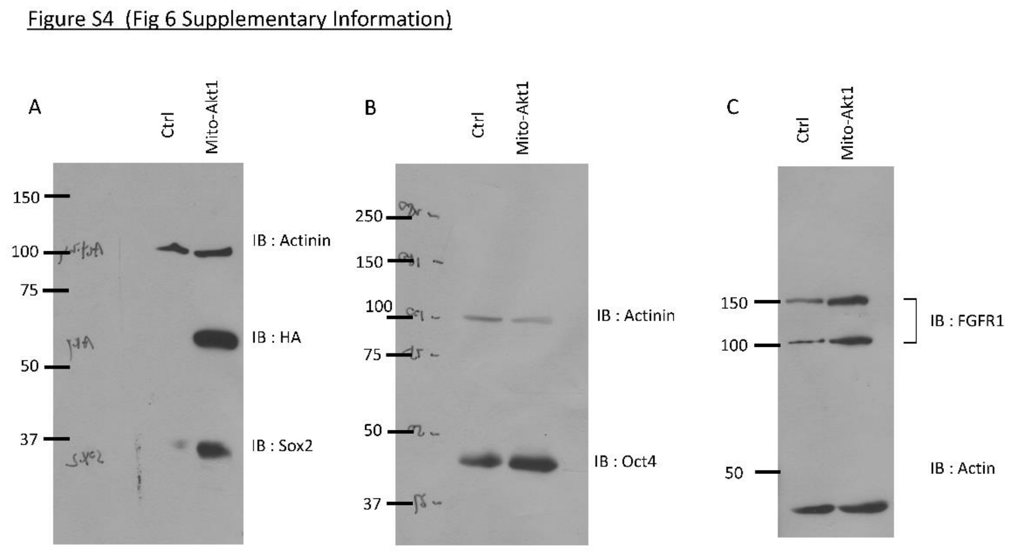

**Supplementary Table 1.** Complete list of the differentially expressed genes in the H9 cells transduced with Mito-Akt1.				
**Functional Group 1**	**Transcript Description**	**Gene Title**	**Bayes.lnp**	**Fold**
PMPCB	Mitochondrial-processing peptidase subunit beta gene:ENSG00000105819 /// Homo sapiens cDNA FLJ78497 complete cds. /// Homo sapiens peptidase (mitochondrial processing) beta (PMPCB), nuclear gene encoding mitochondrial protein, mRNA.	peptidase (mitochondrial processing) beta	0.009160823	1.141642507
MSRB2	Methionine-R-sulfoxide reductase B2 gene:ENSG00000148450 /// Putative uncharacterized protein MSRB2 gene:ENSG00000148450 /// Putative uncharacterized protein MSRB2 gene:ENSG00000148450 /// Homo sapiens methionine sulfoxide reductase B2, mRNA (cDNA clone MGC:151080 IMAGE:40126022), complete cds. /// Homo sapiens methionine sulfoxide reductase B2 (MSRB2), mRNA.	methionine sulfoxide reductase B2	0.002684038	1.174899964
RPS27L	40S ribosomal protein S27-like protein gene:ENSG00000185088 /// 13 kDa protein gene:ENSG00000185088 /// Homo sapiens ribosomal protein S27-like, mRNA (cDNA clone MGC:12175 IMAGE:3827362), complete cds. /// Homo sapiens ribosomal protein S27-like (RPS27L), mRNA.	ribosomal protein S27-like	0.001063446	-1.254328897

COX17	cdna:known chromosome:NCBI36:13:45963033:45963393:1 gene:ENSG00000205105 /// Cytochrome c oxidase copper chaperone gene:ENSG00000138495 /// Homo sapiens COX17 cytochrome c oxidase assembly homolog (S. cerevisiae), mRNA (cDNA clone MGC:104397 IMAGE:6739165), complete cds. /// Homo sapiens COX17 cytochrome c oxidase assembly homolog (S. cerevisiae) (COX17), nuclear gene encoding mitochondrial protein, mRNA.	COX17 cytochrome c oxidase assembly homolog (S. cerevisiae)	0.000841851	1.217993343
CISD1	CDGSH iron sulfur domain-containing protein 1 gene:ENSG00000122873 /// Homo sapiens CDGSH iron sulfur domain 1, mRNA (cDNA clone MGC:14684 IMAGE:4132410), complete cds. /// Homo sapiens CDGSH iron sulfur domain 1 (CISD1), mRNA.	CDGSH iron sulfur domain 1	0.002452111	1.259452186
**Functional Group 2**	**Transcript Description**	**Gene Title**	**Bayes.lnp**	**Fold**
LETM1	LETM1 and EF-hand domain-containing protein 1, mitochondrial gene:ENSG00000168924 /// Homo sapiens leucine zipper-EF-hand containing transmembrane protein 1 (LETM1) mRNA, complete cds. /// Homo sapiens leucine zipper-EF-hand containing transmembrane protein 1 (LETM1), nuclear gene encoding mitochondrial protein, mRNA.	leucine zipper-EF-hand containing transmembrane protein 1	0.001545727	1.200565539

NDUFS7	cDNA FLJ45860 fis, clone OCBBF2036019, moderately similar to NADH-ubiquinone oxidoreductase 20 kDa subunit, mitochondrial gene:ENSG00000115286 /// NADH dehydrogenase [ubiquinone] iron-sulfur protein 7, mitochondrial gene:ENSG00000115286 /// Putative uncharacterized protein NDUFS7 gene:ENSG00000115286 /// Homo sapiens NADH dehydrogenase (ubiquinone) Fe-S protein 7, 20kDa (NADH-coenzyme Q reductase), mRNA (cDNA clone MGC:14592 IMAGE:4276489), complete cds. /// Homo sapiens NADH dehydrogenase (ubiquinone) Fe-S protein 7, 20kDa (NADH-coenzyme Q reductase) (NDUFS7), nuclear gene encoding mitochondrial protein, mRNA.	NADH dehydrogenase (ubiquinone) Fe-S protein 7, 20kDa (NADH-coenzyme Q reductase)	0.000674 651	1.312687 464
NDUFS8	NADH dehydrogenase [ubiquinone] iron-sulfur protein 8, mitochondrial gene:ENSG00000110717 /// Human mitochondrial NADH dehydrogenase-ubiquinone Fe-S protein 8, 23 kDa subunit precursor (NDUFS8) nuclear mRNA encoding mitochondrial protein, complete cds. /// Homo sapiens NADH dehydrogenase (ubiquinone) Fe-S protein 8, 23kDa (NADH-coenzyme Q reductase) (NDUFS8), mRNA.	NADH dehydrogenase (ubiquinone) Fe-S protein 8, 23kDa (NADH-coenzyme Q reductase)	0.000865 971	1.254163 795

BCS1L	Mitochondrial chaperone BCS1 gene:ENSG00000074582 /// Mitochondrial chaperone BCS1 gene:ENSG00000074582 /// Mitochondrial chaperone BCS1 gene:ENSG00000074582 /// Mitochondrial chaperone BCS1 gene:ENSG00000074582 /// Homo sapiens cDNA FLJ38891 fis, clone NHNPC2000062, highly similar to Mitochondrial chaperone BCS1. /// Homo sapiens BCS1-like (yeast) (BCS1L), transcript variant 1, mRNA. /// Homo sapiens BCS1-like (yeast) (BCS1L), nuclear gene encoding mitochondrial protein, transcript variant 2, mRNA.	BCS1-like (yeast)	0.006166574	1.209016657
DNAJC19	Homo sapiens DnaJ (Hsp40) homolog, subfamily C, member 19, mRNA (cDNA clone MGC:9296 IMAGE:3892297), complete cds.	DnaJ (Hsp40) homolog, subfamily C, member 19	0.0012145	1.203453384
TIMM9	Mitochondrial import inner membrane translocase subunit Tim9 gene:ENSG00000100575 /// Mitochondrial import inner membrane translocase subunit Tim9 gene:ENSG00000100575 /// Mitochondrial import inner membrane translocase subunit Tim9 gene:ENSG00000100575 /// Homo sapiens translocase of inner mitochondrial membrane 9 homolog (yeast), mRNA (cDNA clone MGC:31874 IMAGE:4640518), complete cds. /// Homo sapiens translocase of inner mitochondrial membrane 9 homolog (yeast) (TIMM9), nuclear gene encoding mitochondrial protein, mRNA.	translocase of inner mitochondrial membrane 9 homolog (yeast)	0.007083968	1.208403743

TOMM20	Mitochondrial import receptor subunit TOM20 homolog gene:ENSG00000173726 /// Homo sapiens cDNA FLJ75096 complete cds, highly similar to Homo sapiens translocase of outer mitochondrial membrane 20 homolog (yeast) (TOMM20), mRNA. /// Homo sapiens translocase of outer mitochondrial membrane 20 homolog (yeast) (TOMM20), nuclear gene encoding mitochondrial protein, mRNA.	translocase of outer mitochondrial membrane 20 homolog (yeast)	0.006951 31	1.147794 448
AKAP1	cDNA FLJ56047, highly similar to A kinase anchor protein 1, mitochondrial gene:ENSG00000121057 /// Isoform 2 of A kinase anchor protein 1, mitochondrial gene:ENSG00000121057 /// Homo sapiens A kinase (PRKA) anchor protein 1, mRNA (cDNA clone MGC:1807 IMAGE:3509111), complete cds. /// Homo sapiens A kinase (PRKA) anchor protein 1 (AKAP1), nuclear gene encoding mitochondrial protein, mRNA.	A kinase (PRKA) anchor protein 1	0.008499 269	1.153290 063
COX8A	Cytochrome c oxidase polypeptide 8A, mitochondrial gene:ENSG00000176340 /// Homo sapiens cytochrome c oxidase subunit 8A (ubiquitous), mRNA (cDNA clone MGC:70795 IMAGE:6018884), complete cds. /// Homo sapiens cytochrome c oxidase subunit 8A (ubiquitous) (COX8A), mRNA.	cytochrome c oxidase subunit 8A (ubiquitous)	0.000106 919	1.543219 165
CYC1	Cytochrome c1, heme protein, mitochondrial gene:ENSG00000179091 /// Homo sapiens cytochrome c-1, mRNA (cDNA clone MGC:21555 IMAGE:4157475), complete cds. /// Homo sapiens cytochrome c-1 (CYC1), mRNA.	cytochrome c-1	0.007302 705	1.175839 529

COX7C	Cytochrome c oxidase subunit 7C, mitochondrial gene:ENSG00000127184 /// Homo sapiens cDNA: FLJ22852 fis, clone KAT00780. /// Homo sapiens cytochrome c oxidase subunit VIIc (COX7C), nuclear gene encoding mitochondrial protein, mRNA.	cytochrome c oxidase subunit VIIc	0.000769 468	1.233980 908
SLC25A1	Tricarboxylate transport protein, mitochondrial gene:ENSG00000100075 /// Human citrate transporter protein mRNA, nuclear gene encoding mitochondrial protein, complete cds. /// Homo sapiens solute carrier family 25 (mitochondrial carrier; citrate transporter), member 1 (SLC25A1), nuclear gene encoding mitochondrial protein, mRNA.	solute carrier family 25 (mitochondrial carrier; citrate transporter), member 1	0.001141 429	1.205023 561
SLC25A12	cDNA FLJ33752 fis, clone BRCAN2000364, highly similar to Calcium-binding mitochondrial carrier protein Aralar1 gene:ENSG00000115840 /// Calcium-binding mitochondrial carrier protein Aralar1 gene:ENSG00000115840 /// Homo sapiens mRNA for mitochondrial aspartate-glutamate carrier protein (SLC25A12 gene). /// Homo sapiens solute carrier family 25 (mitochondrial carrier, Aralar), member 12 (SLC25A12), nuclear gene encoding mitochondrial protein, mRNA.	solute carrier family 25 (mitochondrial carrier, Aralar), member 12	0.007177 625	1.196424 029
SLC25A15	Mitochondrial ornithine transporter 1 gene:ENSG00000102743 /// Solute carrier family 25 (Mitochondrial carrier gene:ENSG00000102743 /// Homo sapiens ornithine transporter (ORNT1) mRNA, complete cds; nuclear gene for mitochondrial product. /// Homo sapiens solute carrier family 25 (mitochondrial carrier; ornithine transporter) member 15 (SLC25A15), nuclear gene encoding mitochondrial protein, mRNA.	solute carrier family 25 (mitochondrial carrier; ornithine transporter) member 15	0.006833 59	1.178797 763

FECH	ferrochelatase isoform a precursor gene:ENSG00000066926 /// Ferrochelatase, mitochondrial gene:ENSG00000066926 /// Homo sapiens ferrochelatase (protoporphyria), mRNA (cDNA clone MGC:48846 IMAGE:6042757), complete cds. /// Homo sapiens ferrochelatase (protoporphyria) (FECH), nuclear gene encoding mitochondrial protein, transcript variant 1, mRNA. /// Homo sapiens ferrochelatase (protoporphyria) (FECH), nuclear gene encoding mitochondrial protein, transcript variant 2, mRNA.	ferrochelatase (protoporphyria)	0.000989151	1.218840193
SLC25A20	Mitochondrial carnitine/acylcarnitine carrier protein gene:ENSG00000178537 /// Homo sapiens solute carrier family 25 (carnitine/acylcarnitine translocase), member 20, mRNA (cDNA clone MGC:1207 IMAGE:3050073), complete cds. /// Homo sapiens solute carrier family 25 (carnitine/acylcarnitine translocase), member 20 (SLC25A20), nuclear gene encoding mitochondrial protein, mRNA.	solute carrier family 25 (carnitine/acylcarnitine translocase), member 20	0.005186068	1.238336908
NDUFA8	NADH dehydrogenase [ubiquinone] 1 alpha subcomplex subunit 8 gene:ENSG00000119421 /// Homo sapiens NADH:ubiquinone oxidoreductase PGIV subunit mRNA, nuclear gene encoding mitochondrial protein, complete cds. /// Homo sapiens NADH dehydrogenase (ubiquinone) 1 alpha subcomplex, 8, 19kDa (NDUFA8), nuclear gene encoding mitochondrial protein, mRNA.	NADH dehydrogenase (ubiquinone) 1 alpha subcomplex, 8, 19kDa	0.007435462	1.153044821

SDHD	Succinate dehydrogenase [ubiquinone] cytochrome b small subunit, mitochondrial gene:ENSG00000204370 /// Homo sapiens full open reading frame cDNA clone RZPDo834C0116D for gene SDHD, succinate dehydrogenase complex, subunit D, integral membrane protein; complete cds, incl. stopcodon. /// Homo sapiens succinate dehydrogenase complex, subunit D, integral membrane protein (SDHD), nuclear gene encoding mitochondrial protein, mRNA.	succinate dehydrogenase complex, subunit D, integral membrane protein	1.37489E-06	1.493722854
NDUFA1	NADH dehydrogenase [ubiquinone] 1 alpha subcomplex subunit 1 gene:ENSG00000125356 /// Homo sapiens NADH dehydrogenase (ubiquinone) 1 alpha subcomplex, 1, 7.5kDa, mRNA (cDNA clone MGC:2066 IMAGE:3352028), complete cds. /// Homo sapiens NADH dehydrogenase (ubiquinone) 1 alpha subcomplex, 1, 7.5kDa (NDUFA1), nuclear gene encoding mitochondrial protein, mRNA.	NADH dehydrogenase (ubiquinone) 1 alpha subcomplex, 1, 7.5kDa	0.004081918	1.338054193
NDUFS2	NADH dehydrogenase [ubiquinone] iron-sulfur protein 2, mitochondrial gene:ENSG00000158864 /// NADH dehydrogenase [ubiquinone] iron-sulfur protein 2, mitochondrial gene:ENSG00000158864 /// Homo sapiens NADH dehydrogenase (ubiquinone) Fe-S protein 2, 49kDa (NADH-coenzyme Q reductase), mRNA (cDNA clone MGC:15322 IMAGE:4131430), complete cds. /// Homo sapiens NADH dehydrogenase (ubiquinone) Fe-S protein 2, 49kDa (NADH-coenzyme Q reductase) (NDUFS2), mRNA.	NADH dehydrogenase (ubiquinone) Fe-S protein 2, 49kDa (NADH-coenzyme Q reductase)	0.000671587	1.224200737

NDUFB4	NADH dehydrogenase [ubiquinone] 1 beta subcomplex subunit 4 gene:ENSG00000215727 /// cdna:known chromosome:NCBI36:4:43595231:43596125:1 gene:ENSG00000124399 /// NADH dehydrogenase [ubiquinone] 1 beta subcomplex subunit 4 gene:ENSG00000065518 /// Homo sapiens NADH dehydrogenase (ubiquinone) 1 beta subcomplex, 4, 15kDa, mRNA (cDNA clone MGC:5105 IMAGE:3459215), complete cds. /// Homo sapiens NADH dehydrogenase (ubiquinone) 1 beta subcomplex, 4, 15kDa (NDUFB4), nuclear gene encoding mitochondrial protein, mRNA.	NADH dehydrogenase (ubiquinone) 1 beta subcomplex, 4, 15kDa	0.00060312	1.247841764
NDUFV2	cdna:known chromosome:NCBI36:19:58419036:58419949:-1 gene:ENSG00000188699 /// NADH dehydrogenase [ubiquinone] flavoprotein 2, mitochondrial gene:ENSG00000178127 /// 28 kDa protein gene:ENSG00000178127 /// Human nuclear-encoded mitochondrial NADH-ubiquinone reductase 24Kd subunit mRNA, complete cds. /// Homo sapiens NADH dehydrogenase (ubiquinone) flavoprotein 2, 24kDa (NDUFV2), nuclear gene encoding mitochondrial protein, mRNA.	NADH dehydrogenase (ubiquinone) flavoprotein 2, 24kDa	0.004499827	1.218647614

NDUFA3	Putative uncharacterized protein NDUFA3 (Fragment) gene:ENSG00000170906 /// Putative uncharacterized protein NDUFA3 gene:ENSG00000170906 /// NADH dehydrogenase [ubiquinone] 1 alpha subcomplex subunit 3 gene:ENSG00000170906 /// Putative uncharacterized protein NDUFA3 gene:ENSG00000170906 /// Putative uncharacterized protein NDUFA3 gene:ENSG00000170906 /// Homo sapiens NADH:ubiquinone oxidoreductase B9 subunit mRNA, nuclear gene encoding mitochondrial protein, complete cds. /// Homo sapiens NADH dehydrogenase (ubiquinone) 1 alpha subcomplex, 3, 9kDa (NDUFA3), mRNA.	NADH dehydrogenase (ubiquinone) 1 alpha subcomplex, 3, 9kDa	0.001764802	1.219098599
NDUFA2	NADH dehydrogenase [ubiquinone] 1 alpha subcomplex subunit 2 gene:ENSG00000131495 /// Homo sapiens NADH-ubiquinone oxidoreductase subunit CI-B8 mRNA, complete cds. /// Homo sapiens NADH dehydrogenase (ubiquinone) 1 alpha subcomplex, 2, 8kDa (NDUFA2), mRNA.	NADH dehydrogenase (ubiquinone) 1 alpha subcomplex, 2, 8kDa	0.002760904	1.222661835
NDUFB2	NADH dehydrogenase [ubiquinone] 1 beta subcomplex subunit 2, mitochondrial gene:ENSG00000090266 /// NADH-ubiquinone oxidoreductase AGGG subunit gene:ENSG00000090266 /// Homo sapiens NADH dehydrogenase (ubiquinone) 1 beta subcomplex, 2, 8kDa, mRNA (cDNA clone MGC:70788 IMAGE:6064434), complete cds. /// Homo sapiens NADH dehydrogenase (ubiquinone) 1 beta subcomplex, 2, 8kDa (NDUFB2), nuclear gene encoding mitochondrial protein, mRNA.	NADH dehydrogenase (ubiquinone) 1 beta subcomplex, 2, 8kDa	0.009293967	1.175886258

NDUFC1	NADH dehydrogenase [ubiquinone] 1 subunit C1, mitochondrial gene:ENSG00000109390 /// NADH dehydrogenase [ubiquinone] 1 subunit C1, mitochondrial gene:ENSG00000109390 /// NADH dehydrogenase [ubiquinone] 1 subunit C1, mitochondrial gene:ENSG00000109390 /// NADH dehydrogenase [ubiquinone] 1 subunit C1, mitochondrial gene:ENSG00000109390 /// Homo sapiens NADH dehydrogenase (ubiquinone) 1, subcomplex unknown, 1, 6kDa, mRNA (cDNA clone MGC:117464 IMAGE:5557684), complete cds. /// Homo sapiens NADH dehydrogenase (ubiquinone) 1, subcomplex unknown, 1, 6kDa (NDUFC1), mRNA.	NADH dehydrogenase (ubiquinone) 1, subcomplex unknown, 1, 6kDa	0.003609 495	1.182048 219
NDUFS6	NADH dehydrogenase [ubiquinone] iron-sulfur protein 6, mitochondrial gene:ENSG00000145494 /// Homo sapiens NADH:ubiquinone oxidoreductase NDUFS6 subunit mRNA, nuclear gene encoding mitochondrial protein, complete cds. /// Homo sapiens NADH dehydrogenase (ubiquinone) Fe-S protein 6, 13kDa (NADH-coenzyme Q reductase) (NDUFS6), mRNA.	NADH dehydrogenase (ubiquinone) Fe-S protein 6, 13kDa (NADH-coenzyme Q reductase)	0.002664 039	1.174295 423

NDUFB6	NADH dehydrogenase [ubiquinone] 1 beta subcomplex subunit 6 gene:ENSG00000165264 /// NADH dehydrogenase (ubiquinone) 1 beta subcomplex, 6, 17kDa isoform 2 gene:ENSG00000165264 /// Homo sapiens cDNA FLJ76517 complete cds, highly similar to Homo sapiens NADH dehydrogenase (ubiquinone) 1 beta subcomplex, 6, 17kDa (NDUFB6), nuclear gene encoding mitochondrial protein, transcript variant 1, mRNA. /// Homo sapiens NADH dehydrogenase (ubiquinone) 1 beta subcomplex, 6, 17kDa (NDUFB6), nuclear gene encoding mitochondrial protein, transcript variant 2, mRNA. /// Homo sapiens NADH dehydrogenase (ubiquinone) 1 beta subcomplex, 6, 17kDa (NDUFB6), nuclear gene encoding mitochondrial protein, transcript variant 1, mRNA.	NADH dehydrogenase (ubiquinone) 1 beta subcomplex, 6, 17kDa	0.004457401	1.397064706
NDUFB10	NADH dehydrogenase [ubiquinone] 1 beta subcomplex subunit 10 gene:ENSG00000140990 /// Homo sapiens NADH dehydrogenase (ubiquinone) 1 beta subcomplex, 10, 22kDa, mRNA (cDNA clone MGC:4153 IMAGE:3030207), complete cds. /// Homo sapiens NADH dehydrogenase (ubiquinone) 1 beta subcomplex, 10, 22kDa (NDUFB10), mRNA.	NADH dehydrogenase (ubiquinone) 1 beta subcomplex, 10, 22kDa	0.004386163	1.154936832
FIS1	Mitochondrial fission 1 protein gene:ENSG00000214253 /// Homo sapiens CGI-135 protein mRNA, complete cds. /// Homo sapiens fission 1 (mitochondrial outer membrane) homolog (S. cerevisiae) (FIS1), nuclear gene encoding mitochondrial protein, mRNA.	fission 1 (mitochondrial outer membrane) homolog (S. cerevisiae)	0.004805165	1.187836666

SLC25A11	Mitochondrial 2-oxoglutarate/malate carrier protein gene:ENSG00000108528 /// Homo sapiens solute carrier family 25 (mitochondrial carrier; oxoglutarate carrier), member 11, mRNA (cDNA clone MGC:2449 IMAGE:2960933), complete cds. /// Homo sapiens solute carrier family 25 (mitochondrial carrier; oxoglutarate carrier), member 11 (SLC25A11), nuclear gene encoding mitochondrial protein, mRNA.	solute carrier family 25 (mitochondrial carrier; oxoglutarate carrier), member 11	0.009551499	1.150444011
SLC25A16	Graves disease carrier protein gene:ENSG00000122912 /// 9 kDa protein gene:ENSG00000122912 /// Homo sapiens solute carrier family 25 (mitochondrial carrier; Graves disease autoantigen), member 16, mRNA (cDNA clone MGC:39851 IMAGE:5241247), complete cds. /// Homo sapiens solute carrier family 25 (mitochondrial carrier; Graves disease autoantigen), member 16 (SLC25A16), nuclear gene encoding mitochondrial protein, mRNA.	solute carrier family 25 (mitochondrial carrier; Graves disease autoantigen), member 16	0.000186041	-1.364807187
ETFDH	Isoform 1 of Electron transfer flavoprotein-ubiquinone oxidoreductase, mitochondrial gene:ENSG00000171503 /// Isoform 2 of Electron transfer flavoprotein-ubiquinone oxidoreductase, mitochondrial gene:ENSG00000171503 /// Homo sapiens electron-transferring-flavoprotein dehydrogenase, mRNA (cDNA clone MGC:20062 IMAGE:3938660), complete cds. /// Homo sapiens electron-transferring-flavoprotein dehydrogenase (ETFDH), nuclear gene encoding mitochondrial protein, mRNA.	electron-transferring-flavoprotein dehydrogenase	0.000685736	1.353307611

TOMM34	Mitochondrial import receptor subunit TOM34 gene:ENSG00000025772 /// cDNA FLJ26027 fis, clone PNC04328, highly similar to Homo sapiens translocase of outer mitochondrial membrane 34 gene:ENSG00000025772 /// Homo sapiens translocase of outer mitochondrial membrane 34, mRNA (cDNA clone MGC:3515 IMAGE:2960090), complete cds. /// Homo sapiens translocase of outer mitochondrial membrane 34 (TOMM34), nuclear gene encoding mitochondrial protein, mRNA.	translocase of outer mitochondrial membrane 34	0.006754355	1.167816537
NIPSNAP1	Protein NipSnap homolog 1 gene:ENSG00000184117 /// Homo sapiens mRNA for NIPSNAP1 protein. /// Homo sapiens nipsnap homolog 1 (C. elegans) (NIPSNAP1), mRNA.	nipsnap homolog 1 (C. elegans)	0.001274444	1.209693015
SLC27A3	Isoform 1 of Long-chain fatty acid transport protein 3 gene:ENSG00000143554 /// Isoform 1 of Long-chain fatty acid transport protein 3 gene:ENSG00000143554 /// 11 kDa protein gene:ENSG00000143554 /// Solute carrier family 27 (Fatty acid transporter), member 3 gene:ENSG00000143554 /// Putative uncharacterized protein gene:ENSG00000216243 /// Homo sapiens cDNA FLJ43112 fis, clone CTONG2028124, weakly similar to Homo sapiens VLCS-H1 protein (VLCS-H1). /// Homo sapiens solute carrier family 27 (fatty acid transporter), member 3 (SLC27A3), mRNA.	solute carrier family 27 (fatty acid transporter), member 3	0.002911584	1.206127894

C17orf61	UPF0451 protein C17orf61 gene:ENSG00000205544 /// Homo sapiens cDNA FLJ31260 fis, clone KIDNE2005854, highly similar to Phospholipid scramblase 3. /// Homo sapiens cDNA FLJ77856 complete cds, highly similar to Homo sapiens phospholipid scramblase 3 (PLSCR3), mRNA. /// Homo sapiens chromosome 17 open reading frame 61, mRNA (cDNA clone MGC:40107 IMAGE:5418259), complete cds.	phospholipid scramblase 3 /// chromosome 17 open reading frame 61	0.001099691	1.276122626
CCDC56	Coiled-coil domain-containing protein 56 gene:ENSG00000183978 /// Homo sapiens mRNA for HSPC009 variant, clone: PNC06538. /// Homo sapiens coiled-coil domain containing 56 (CCDC56), mRNA.	coiled-coil domain containing 56	0.003281488	1.232223987
**Functional Group 3**	**Transcript Description**	**Gene Title**	**Bayes.lnp**	**Fold**
THEM2	Thioesterase superfamily member 2 gene:ENSG00000112304 /// Homo sapiens uncharacterized hypothalamus protein HT012 mRNA, complete cds. /// Homo sapiens thioesterase superfamily member 2 (THEM2), mRNA.	thioesterase superfamily member 2	0.009507814	1.144254435
DRAM	Damage-regulated autophagy modulator gene:ENSG00000136048 /// Homo sapiens damage-regulated autophagy modulator, mRNA (cDNA clone MGC:21716 IMAGE:4474297), complete cds. /// Homo sapiens damage-regulated autophagy modulator (DRAM), mRNA.	damage-regulated autophagy modulator	0.000780827	-1.247606428
FKTN	Fukutin gene:ENSG00000106692 /// 26 kDa protein gene:ENSG00000106692 /// 27 kDa protein gene:ENSG00000106692 /// Homo sapiens FCMD mRNA for fukutin, complete cds. /// Homo sapiens fukutin (FKTN), transcript variant 2, mRNA. /// Homo sapiens fukutin (FKTN), transcript variant 1, mRNA.	fukutin	0.003004349	1.219800207

ERGIC1	cdna:known chromosome:NCBI36:5:172193928:172258194:1 gene:ENSG00000113719 /// 35 kDa protein gene:ENSG00000113719 /// Isoform 1 of Endoplasmic reticulum-Golgi intermediate compartment protein 1 gene:ENSG00000113719 /// cdna:known chromosome:NCBI36:5:172194020:172280180:1 gene:ENSG00000113719 /// Isoform 3 of Endoplasmic reticulum-Golgi intermediate compartment protein 1 gene:ENSG00000113719 /// Homo sapiens endoplasmic reticulum-golgi intermediate compartment (ERGIC) 1, mRNA (cDNA clone MGC:16233 IMAGE:3677787), complete cds. /// Homo sapiens endoplasmic reticulum-golgi intermediate compartment (ERGIC) 1 (ERGIC1), mRNA.	endoplasmic reticulum-golgi intermediate compartment (ERGIC) 1	0.000491703	1.243551427
TMED3	Transmembrane emp24 domain-containing protein 3 gene:ENSG00000166557 /// Homo sapiens cDNA FLJ75946 complete cds, highly similar to Homo sapiens transmembrane emp24 protein transport domain containing 3 (TMED3), mRNA. /// Homo sapiens transmembrane emp24 protein transport domain containing 3 (TMED3), mRNA.	transmembrane emp24 protein transport domain containing 3	0.009634817	1.172139107
SVIP	Small VCP/p97-interacting protein gene:ENSG00000198168 /// Homo sapiens mRNA; cDNA DKFZp313A2432 (from clone DKFZp313A2432). /// Homo sapiens small VCP/p97-interacting protein (SVIP), mRNA.	small VCP/p97-interacting protein	0.004605461	1.268457487
SLC35A5	Probable UDP-sugar transporter protein SLC35A5 gene:ENSG00000138459 /// Homo sapiens clone DNA23334 SLC35A5 (UNQ164) mRNA, complete cds. /// Homo sapiens solute carrier family 35, member A5 (SLC35A5), mRNA.	solute carrier family 35, member A5	0.001150165	1.208143764

MANBAL	Protein MANBAL gene:ENSG00000101363 /// Protein MANBAL gene:ENSG00000101363 /// Mannosidase, beta A, lysosomal-like gene:ENSG00000101363 /// Protein MANBAL gene:ENSG00000101363 /// Protein MANBAL gene:ENSG00000101363 /// Protein MANBAL gene:ENSG00000101363 /// Homo sapiens cDNA: FLJ23055 fis, clone LNG03262. /// Homo sapiens mannosidase, beta A, lysosomal-like (MANBAL), transcript variant 2, mRNA. /// Homo sapiens mannosidase, beta A, lysosomal-like (MANBAL), transcript variant 1, mRNA.	mannosidase, beta A, lysosomal-like	9.61227E-05	1.298557075
LAPTM5	Lysosomal-associated transmembrane protein 5 gene:ENSG00000162511 /// Homo sapiens cDNA FLJ61683 complete cds, moderately similar to Lysosomal-associated multitransmembrane protein. /// Human lysosomal-associated multitransmembrane protein (LAPTm5) mRNA, complete cds. /// Homo sapiens lysosomal multispanning membrane protein 5 (LAPTM5), mRNA.	actin filament associated protein 1-like 1 /// lysosomal multispanning membrane protein 5	0.000670384	-1.424435353
INSIG1	insulin induced gene 1 isoform 2 gene:ENSG00000186480 /// Insulin-induced gene 1 protein gene:ENSG00000186480 /// insulin induced gene 1 isoform 3 gene:ENSG00000186480 /// Homo sapiens insulin induced gene 1, mRNA (cDNA clone MGC:1405 IMAGE:3546928), complete cds. /// Homo sapiens insulin induced gene 1 (INSIG1), transcript variant 1, mRNA. /// Homo sapiens insulin induced gene 1 (INSIG1), transcript variant 2, mRNA. /// Homo sapiens insulin induced gene 1 (INSIG1), transcript variant 3, mRNA.	insulin induced gene 1	0.000115835	1.293557142

IER3IP1	Homo sapiens PRO2309 mRNA, complete cds.	immediate early response 3 interacting protein 1	0.416335366	-1.125829568
TOR1AIP2	Torsin-1A-interacting protein 2 gene:ENSG00000169905 /// IFRG15 protein gene:ENSG00000218839 /// Homo sapiens cDNA FLJ77012 complete cds, highly similar to Homo sapiens interferon responsive gene 15 (IFRG15), mRNA. /// Homo sapiens torsin A interacting protein 2, mRNA (cDNA clone MGC:126581 IMAGE:8069038), complete cds. /// Homo sapiens torsin A interacting protein 2 (TOR1AIP2), mRNA.	interferon responsive gene 15 /// torsin A interacting protein 2	0.001893375	-1.192769275
SEC22C	Isoform 2 of Vesicle-trafficking protein SEC22c gene:ENSG00000093183 /// Isoform 3 of Vesicle-trafficking protein SEC22c gene:ENSG00000093183 /// Isoform 1 of Vesicle-trafficking protein SEC22c gene:ENSG00000093183 /// Homo sapiens cDNA FLJ57251 complete cds, highly similar to Homo sapiens SEC22 vesicle trafficking protein-like 3, transcript variant 2, mRNA. /// Homo sapiens SEC22 vesicle trafficking protein homolog C (S. cerevisiae) (SEC22C), transcript variant 1, mRNA. /// Homo sapiens SEC22 vesicle trafficking protein homolog C (S. cerevisiae) (SEC22C), transcript variant 2, mRNA.	SEC22 vesicle trafficking protein homolog C (S. cerevisiae)	0.003178567	1.201000039
CCDC3	Novel protein gene:ENSG00000151468 /// Coiled-coil domain-containing protein 3 gene:ENSG00000151468 /// Homo sapiens coiled-coil domain containing 3, mRNA (cDNA clone MGC:59716 IMAGE:6302570), complete cds. /// Homo sapiens coiled-coil domain containing 3 (CCDC3), mRNA.	coiled-coil domain containing 3	9.73491E-06	-1.45881522

TMEM126A	Transmembrane protein 126A gene:ENSG00000171202 /// Homo sapiens transmembrane protein 126A, mRNA (cDNA clone MGC:14138 IMAGE:3948518), complete cds. /// Homo sapiens transmembrane protein 126A (TMEM126A), mRNA.	transmembrane protein 126A	0.003177148	1.274946674
C17orf61	UPF0451 protein C17orf61 gene:ENSG00000205544 /// Homo sapiens cDNA FLJ31260 fis, clone KIDNE2005854, highly similar to Phospholipid scramblase 3. /// Homo sapiens cDNA FLJ77856 complete cds, highly similar to Homo sapiens phospholipid scramblase 3 (PLSCR3), mRNA. /// Homo sapiens chromosome 17 open reading frame 61, mRNA (cDNA clone MGC:40107 IMAGE:5418259), complete cds.	phospholipid scramblase 3 /// chromosome 17 open reading frame 61	0.001099691	1.276122626
DKFZP564O0823	Protein PARM-1 gene:ENSG00000169116 /// Homo sapiens clone DNA92223 VYKT1879 (UNQ1879) mRNA, complete cds. /// Homo sapiens DKFZP564O0823 protein (DKFZP564O0823), mRNA.	DKFZP564O0823 protein	5.61324E-05	-1.348721612
C6orf162	Uncharacterized protein C6orf162 gene:ENSG00000111850 /// Uncharacterized protein C6orf162 gene:ENSG00000111850 /// Homo sapiens chromosome 6 open reading frame 162, mRNA (cDNA clone MGC:88247 IMAGE:6452586), complete cds. /// Homo sapiens chromosome 6 open reading frame 162 (C6orf162), transcript variant 2, mRNA. /// Homo sapiens chromosome 6 open reading frame 162 (C6orf162), transcript variant 1, mRNA.	chromosome 6 open reading frame 162	0.003066727	1.199326745
PXMP2	Homo sapiens peroxisomal membrane protein 2, 22kDa, mRNA (cDNA clone IMAGE:4098463), complete cds.	peroxisomal membrane protein 2, 22kDa	0.121401801	1.149864533

SMCR7L	Putative uncharacterized protein DKFZp434F0116 gene:ENSG00000100335 /// Smith-Magenis syndrome chromosome region candidate gene 7 protein-like gene:ENSG00000100335 /// Putative uncharacterized protein DKFZp434F0116 gene:ENSG00000100335 /// Smith-Magenis syndrome chromosome region candidate gene 7 protein-like gene:ENSG00000100335 /// Homo sapiens Smith-Magenis syndrome chromosome region, candidate 7-like, mRNA (cDNA clone MGC:15774 IMAGE:3502711), complete cds. /// Homo sapiens Smith-Magenis syndrome chromosome region, candidate 7-like (SMCR7L), mRNA.	Smith-Magenis syndrome chromosome region, candidate 7-like	0.003381 395	1.214924 348
C9orf46	Transmembrane protein C9orf46 gene:ENSG00000107020 /// Homo sapiens uncharacterized hematopoietic stem/progenitor cells protein MDS030 mRNA, complete cds. /// Homo sapiens AD025 mRNA, complete cds.	chromosome 9 open reading frame 46	0.003702 152	1.261456 637
STARD3NL	Isoform 1 of MLN64 N-terminal domain homolog gene:ENSG00000010270 /// Isoform 1 of MLN64 N-terminal domain homolog gene:ENSG00000010270 /// Homo sapiens cDNA FLJ34260 fis, clone FEBRA2000782, moderately similar to MLN 64 PROTEIN. /// Homo sapiens STARD3 N-terminal like (STARD3NL), mRNA.	STARD3 N-terminal like	0.000773 235	1.251229 806
SCAMP2	Secretory carrier-associated membrane protein 2 gene:ENSG00000140497 /// Homo sapiens cDNA FLJ39475 fis, clone PROST2013121, highly similar to SECRETORY CARRIER-ASSOCIATED MEMBRANE PROTEIN 2. /// Homo sapiens secretory carrier membrane protein 2 (SCAMP2), mRNA.	secretory carrier membrane protein 2	0.001183 903	1.201401 381

SLC44A5	Isoform 2 of Choline transporter-like protein 5 gene:ENSG00000137968 /// Isoform 2 of Choline transporter-like protein 5 gene:ENSG00000137968 /// Isoform 1 of Choline transporter-like protein 5 gene:ENSG00000137968 /// Putative uncharacterized protein SLC44A5 gene:ENSG00000137968 /// Homo sapiens solute carrier family 44, member 5, mRNA (cDNA clone MGC:42574 IMAGE:4824861), complete cds. /// Homo sapiens solute carrier family 44, member 5 (SLC44A5), transcript variant 1, mRNA. /// Homo sapiens solute carrier family 44, member 5 (SLC44A5), transcript variant 2, mRNA.	solute carrier family 44, member 5	0.001243544	-1.364925707
CCDC56	Coiled-coil domain-containing protein 56 gene:ENSG00000183978 /// Homo sapiens mRNA for HSPC009 variant, clone: PNC06538. /// Homo sapiens coiled-coil domain containing 56 (CCDC56), mRNA.	coiled-coil domain containing 56	0.003281488	1.232223987
C9orf123	Isoform 2 of Transmembrane protein C9orf123 gene:ENSG00000137038 /// Homo sapiens cDNA FLJ76577 complete cds. /// Homo sapiens chromosome 9 open reading frame 123 (C9orf123), mRNA.	chromosome 9 open reading frame 123	2.58832E-05	1.339680345
SCAMP3	Isoform 1 of Secretory carrier-associated membrane protein 3 gene:ENSG00000116521 /// Isoform 2 of Secretory carrier-associated membrane protein 3 gene:ENSG00000116521 /// Homo sapiens secretory carrier membrane protein (SCAMP3) mRNA, complete cds. /// Homo sapiens secretory carrier membrane protein 3 (SCAMP3), transcript variant 2, mRNA. /// Homo sapiens secretory carrier membrane protein 3 (SCAMP3), transcript variant 1, mRNA.	secretory carrier membrane protein 3	0.000826331	1.216168119

HINT2	Histidine triad nucleotide-binding protein 2 gene:ENSG00000137133 /// Homo sapiens histidine triad nucleotide binding protein 2, mRNA (cDNA clone MGC:54098 IMAGE:6018376), complete cds. /// Homo sapiens histidine triad nucleotide binding protein 2 (HINT2), mRNA.	histidine triad nucleotide binding protein 2	0.001865597	1.243596734
NIPSNAP1	Protein NipSnap homolog 1 gene:ENSG00000184117 /// Homo sapiens mRNA for NIPSNAP1 protein. /// Homo sapiens nipsnap homolog 1 (C. elegans) (NIPSNAP1), mRNA.	nipsnap homolog 1 (C. elegans)	0.001274444	1.209693015
MTERFD1	Isoform 1 of mTERF domain-containing protein 1, mitochondrial gene:ENSG00000156469 /// Homo sapiens MTERF domain containing 1, mRNA (cDNA clone MGC:3363 IMAGE:3529518), complete cds. /// Homo sapiens MTERF domain containing 1 (MTERFD1), mRNA.	MTERF domain containing 1	0.006977788	1.239823683
C22orf32	UPF0466 protein C22orf32, mitochondrial gene:ENSG00000183172 /// Homo sapiens cDNA FLJ38317 fis, clone FCBBF3023704. /// Homo sapiens chromosome 22 open reading frame 32, mRNA (cDNA clone MGC:32898 IMAGE:4798793), complete cds. /// Homo sapiens dJ186O1.1 full length open reading frame (ORF) cDNA clone (cDNA clone C22ORF:pGEM.dJ186O1.1.V4).	chromosome 22 open reading frame 32	0.004270924	1.201164319
KIAA0101	PCNA-associated factor gene:ENSG00000166803 /// hypothetical protein LOC9768 isoform 2 gene:ENSG00000166803 /// GKGM353 gene:ENSG00000219244 /// Homo sapiens KIAA0101, mRNA (cDNA clone MGC:2250 IMAGE:2988995), complete cds. /// Homo sapiens KIAA0101 (KIAA0101), transcript variant 1, mRNA. /// Homo sapiens	KIAA0101	0.001796385	1.29487092

	KIAA0101 (KIAA0101), transcript variant 2, mRNA.			
CISD1	CDGSH iron sulfur domain-containing protein 1 gene:ENSG00000122873 /// Homo sapiens CDGSH iron sulfur domain 1, mRNA (cDNA clone MGC:14684 IMAGE:4132410), complete cds. /// Homo sapiens CDGSH iron sulfur domain 1 (CISD1), mRNA.	CDGSH iron sulfur domain 1	0.002452111	1.259452186
**Functional Group 4**	**Transcript Description**	**Gene Title**	**Bayes.lnp**	**Fold**
ETFDH	Isoform 1 of Electron transfer flavoprotein-ubiquinone oxidoreductase, mitochondrial gene:ENSG00000171503 /// Isoform 2 of Electron transfer flavoprotein-ubiquinone oxidoreductase, mitochondrial gene:ENSG00000171503 /// Homo sapiens electron-transferring-flavoprotein dehydrogenase, mRNA (cDNA clone MGC:20062 IMAGE:3938660), complete cds. /// Homo sapiens electron-transferring-flavoprotein dehydrogenase (ETFDH), nuclear gene encoding mitochondrial protein, mRNA.	electron-transferring-flavoprotein dehydrogenase	0.000685736	1.353307611

GATM	Isoform Cytoplasmic of Glycine amidinotransferase, mitochondrial gene:ENSG00000171766 /// Isoform Mitochondrial of Glycine amidinotransferase, mitochondrial gene:ENSG00000171766 /// Homo sapiens glycine amidinotransferase (L-arginine:glycine amidinotransferase), mRNA (cDNA clone MGC:1744 IMAGE:3010128), complete cds. /// Homo sapiens glycine amidinotransferase (L-arginine:glycine amidinotransferase) (GATM), nuclear gene encoding mitochondrial protein, mRNA.	glycine amidinotransferase (L-arginine:glycine amidinotransferase)	0.002893 872	1.190716 588
ACADVL	cDNA FLJ56425, highly similar to Very-long-chain specific acyl-CoAdehydrogenase, mitochondrial gene:ENSG00000072778 /// cDNA FLJ56425, highly similar to Very-long-chain specific acyl-CoAdehydrogenase, mitochondrial gene:ENSG00000072778 /// Isoform 2 of Very long-chain specific acyl-CoA dehydrogenase, mitochondrial gene:ENSG00000072778 /// Homo sapiens acyl-Coenzyme A dehydrogenase, very long chain, mRNA (cDNA clone MGC:21196 IMAGE:4471040), complete cds. /// Homo sapiens acyl-Coenzyme A dehydrogenase, very long chain (ACADVL), nuclear gene encoding mitochondrial protein, transcript variant 1, mRNA. /// Homo sapiens acyl-Coenzyme A dehydrogenase, very long chain (ACADVL), nuclear gene encoding mitochondrial protein, transcript variant 2, mRNA.	acyl-Coenzyme A dehydrogenase, very long chain	0.008181 275	- 1.142638 64

DCI	Isoform 1 of 3,2-trans-enoyl-CoA isomerase, mitochondrial gene:ENSG00000167969 /// Homo sapiens cDNA FLJ76046 complete cds, highly similar to Homo sapiens dodecenoyl-Coenzyme A delta isomerase (3,2trans-enoyl-Coenzyme A isomerase) (DCI), mRNA. /// Homo sapiens dodecenoyl-Coenzyme A delta isomerase (3,2 trans-enoyl-Coenzyme A isomerase) (DCI), nuclear gene encoding mitochondrial protein, mRNA.	dodecenoyl-Coenzyme A delta isomerase (3,2 trans-enoyl-Coenzyme A isomerase)	0.003412221	1.180928766
FDXR	Isoform Long of NADPH:adrenodoxin oxidoreductase, mitochondrial gene:ENSG00000161513 /// Homo sapiens ferredoxin reductase, mRNA (cDNA clone MGC:1505 IMAGE:3537008), complete cds. /// Homo sapiens ferredoxin reductase (FDXR), nuclear gene encoding mitochondrial protein, transcript variant 1, mRNA. /// Homo sapiens ferredoxin reductase (FDXR), nuclear gene encoding mitochondrial protein, transcript variant 2, mRNA.	ferredoxin reductase	0.000485311	-1.246605036
ETFA	Electron transfer flavoprotein subunit alpha, mitochondrial gene:ENSG00000140374 /// Homo sapiens cDNA FLJ76486 complete cds, highly similar to Homo sapiens electron-transfer-flavoprotein, alpha polypeptide (glutaric aciduria II) (ETFA), nuclear gene encoding mitochondrial protein, mRNA. /// Homo sapiens electron-transfer-flavoprotein, alpha polypeptide (ETFA), nuclear gene encoding mitochondrial protein, transcript variant 1, mRNA. /// Homo sapiens electron-transfer-flavoprotein, alpha polypeptide (ETFA), transcript variant 2, mRNA.	electron-transfer-flavoprotein, alpha polypeptide	0.002297232	1.203357136

DLD	Dihydrolipoyl dehydrogenase, mitochondrial gene:ENSG00000091140 /// Homo sapiens cDNA FLJ56061 complete cds, highly similar to Dihydrolipoyl dehydrogenase, mitochondrial precursor (EC 1.8.1.4). /// Homo sapiens dihydrolipoamide dehydrogenase (DLD), mRNA.	dihydrolipoamide dehydrogenase	0.008705796	1.146281234
PCCB	Propionyl-CoA carboxylase beta chain, mitochondrial gene:ENSG00000114054 /// Homo sapiens propionyl Coenzyme A carboxylase, beta polypeptide, mRNA (cDNA clone MGC:61502 IMAGE:6054692), complete cds. /// Homo sapiens propionyl Coenzyme A carboxylase, beta polypeptide (PCCB), nuclear gene encoding mitochondrial protein, mRNA.	propionyl Coenzyme A carboxylase, beta polypeptide	0.002563897	1.175017123
SLC27A3	Isoform 1 of Long-chain fatty acid transport protein 3 gene:ENSG00000143554 /// Isoform 1 of Long-chain fatty acid transport protein 3 gene:ENSG00000143554 /// 11 kDa protein gene:ENSG00000143554 /// Solute carrier family 27 (Fatty acid transporter), member 3 gene:ENSG00000143554 /// Putative uncharacterized protein gene:ENSG00000216243 /// Homo sapiens cDNA FLJ43112 fis, clone CTONG2028124, weakly similar to Homo sapiens VLCS-H1 protein (VLCS-H1). /// Homo sapiens solute carrier family 27 (fatty acid transporter), member 3 (SLC27A3), mRNA.	solute carrier family 27 (fatty acid transporter), member 3	0.002911584	1.206127894
**Functional Group 5**	**Transcript Description**	**Gene Title**	**Bayes.lnp**	**Fold**

CCDC50	70 kDa protein gene:ENSG00000152492 /// 50 kDa protein gene:ENSG00000152492 /// Isoform 2 of Coiled-coil domain-containing protein 50 gene:ENSG00000152492 /// Isoform 1 of Coiled-coil domain-containing protein 50 gene:ENSG00000152492 /// Homo sapiens coiled-coil domain containing 50, mRNA (cDNA clone MGC:70346 IMAGE:6049272), complete cds. /// Homo sapiens coiled-coil domain containing 50 (CCDC50), transcript variant 2, mRNA. /// Homo sapiens coiled-coil domain containing 50 (CCDC50), transcript variant 1, mRNA.	coiled-coil domain containing 50	0.004869264	-1.177310654
FAM125A	Isoform 1 of Protein FAM125A gene:ENSG00000141971 /// Isoform 2 of Protein FAM125A gene:ENSG00000141971 /// Homo sapiens cDNA FLJ32495 fis, clone SKNSH2000163. /// Homo sapiens family with sequence similarity 125, member A (FAM125A), mRNA.	family with sequence similarity 125, member A	0.001402382	1.237034533

KIAA1217	sickle tail isoform 2 gene:ENSG00000120549 /// Likely orthologue of Mus musculus enhancer trap locus 4 gene:ENSG00000120549 /// sickle tail isoform 2 gene:ENSG00000120549 /// Isoform 1 of Sickle tail protein homolog gene:ENSG00000120549 /// Putative uncharacterized protein KIAA1217 gene:ENSG00000120549 /// Isoform 6 of Sickle tail protein homolog gene:ENSG00000120549 /// Isoform 4 of Sickle tail protein homolog gene:ENSG00000120549 /// Isoform 7 of Sickle tail protein homolog gene:ENSG00000120549 /// Isoform 3 of Sickle tail protein homolog gene:ENSG00000120549 /// Homo sapiens mRNA; cDNA DKFZp686E0469 (from clone DKFZp686E0469); complete cds. /// Homo sapiens KIAA1217 (KIAA1217), transcript variant 1, mRNA. /// Homo sapiens KIAA1217 (KIAA1217), transcript variant 2, mRNA. /// Homo sapiens KIAA1217 (KIAA1217), transcript variant 3, mRNA.	KIAA1217	0.000528524	-1.241607706
NBPF15	Putative uncharacterized protein ENSP00000349255 gene:ENSG00000162825 /// Neuroblastoma breakpoint family, member 10 gene:ENSG00000203832 /// Homo sapiens cDNA FLJ78393 complete cds. /// Homo sapiens cDNA FLJ60520 complete cds, weakly similar to Homo sapiens phosphodiesterase 4D interacting protein, transcript variant 1, mRNA. /// Homo sapiens mRNA; cDNA DKFZp586O031 (from clone DKFZp586O031). /// Homo sapiens clone IIIb5 NBPF15 mRNA, complete cds.	neuroblastoma breakpoint family, member 15 /// neuroblastoma breakpoint family, member 14 /// neuroblastoma breakpoint family, member 10	0.012580751	-1.15646752
**Functional Group 6**	**Transcript Description**	**Gene Title**	**Bayes.lnp**	**Fold**

GINS2	DNA replication complex GINS protein PSF2 gene:ENSG00000131153 /// Homo sapiens GINS complex subunit 2 (Psf2 homolog), mRNA (cDNA clone MGC:19836 IMAGE:4098007), complete cds. /// Homo sapiens GINS complex subunit 2 (Psf2 homolog) (GINS2), mRNA.	GINS complex subunit 2 (Psf2 homolog)	0.001926016	1.188229666
CABYR	Isoform 3 of Calcium-binding tyrosine phosphorylation-regulated protein gene:ENSG00000154040 /// Isoform 5 of Calcium-binding tyrosine phosphorylation-regulated protein gene:ENSG00000154040 /// Isoform 5 of Calcium-binding tyrosine phosphorylation-regulated protein gene:ENSG00000154040 /// Isoform 1 of Calcium-binding tyrosine phosphorylation-regulated protein gene:ENSG00000154040 /// Isoform 2 of Calcium-binding tyrosine phosphorylation-regulated protein gene:ENSG00000154040 /// Isoform 1 of Calcium-binding tyrosine phosphorylation-regulated protein gene:ENSG00000154040 /// Isoform 2 of Calcium-binding tyrosine phosphorylation-regulated protein gene:ENSG00000154040 /// Isoform 3 of Calcium-binding tyrosine phosphorylation-regulated protein gene:ENSG00000154040 /// Homo sapiens fibrousheathin II mRNA, complete cds. /// Homo sapiens calcium binding tyrosine-(Y)-phosphorylation regulated (CABYR), transcript variant 1, mRNA. /// Homo sapiens calcium binding tyrosine-(Y)-phosphorylation regulated (CABYR), transcript variant 2, mRNA. /// Homo sapiens calcium binding tyrosine-(Y)-phosphorylation regulated (CABYR), transcript variant 7, mRNA. /// Homo sapiens calcium binding tyrosine-(Y)-phosphorylation regulated (CABYR), transcript variant 6, mRNA. /// Homo sapiens calcium binding tyrosine-(Y)-phosphorylation regulated (CABYR),	calcium binding tyrosine-(Y)-phosphorylation regulated	0.000373376	-1.235811167

	transcript variant 5, mRNA. /// Homo sapiens calcium binding tyrosine-(Y)-phosphorylation regulated (CABYR), transcript variant 3, mRNA.			
ZFAND2A	cDNA FLJ76351, highly similar to Homo sapiens zinc finger, AN1-type domain 2A (ZFAND2A), mRNA gene:ENSG00000178381 /// Putative uncharacterized protein ZFAND2A gene:ENSG00000178381 /// AN1-type zinc finger protein 2A gene:ENSG00000178381 /// Homo sapiens zinc finger, AN1-type domain 2A, mRNA (cDNA clone MGC:39501 IMAGE:5263788), complete cds. /// Homo sapiens zinc finger, AN1-type domain 2A (ZFAND2A), mRNA.	zinc finger, AN1-type domain 2A	0.000191425	-1.355119998

SPANXF1	Sperm protein associated with the nucleus on the X chromosome B/F gene:ENSG00000198820 /// Sperm protein associated with the nucleus on the X chromosome B/F gene:ENSG00000203929 /// Putative uncharacterized protein SPANXC gene:ENSG00000198573 /// Homo sapiens nuclear-associated protein SPAN-Xb (SPANX) mRNA, complete cds. /// Homo sapiens SPAN-Xd mRNA, complete cds. /// Homo sapiens SPANX family, member B1 (SPANXB1), mRNA. /// Homo sapiens SPANX family, member B2 (SPANXB2), mRNA. /// Homo sapiens SPANX family, member F1 (SPANXF1), mRNA.	SPANX family, member B1 /// SPANX family, member B2 /// SPANX family, member D /// SPANX family, member F1	1.20E-05	-1.617080841
NDRG1	cDNA FLJ39243 fis, clone OCBBF2008283, highly similar to Protein NDRG1 gene:ENSG00000104419 /// Protein NDRG1 gene:ENSG00000104419 /// Homo sapiens cDNA FLJ44977 fis, clone BRAWH3002513, highly similar to Protein NDRG1. /// Homo sapiens N-myc downstream regulated 1 (NDRG1), transcript variant 2, mRNA. /// Homo sapiens N-myc downstream regulated 1 (NDRG1), transcript variant 1, mRNA.	N-myc downstream regulated 1	3.39352E-05	-1.362878196
LPIN1	Putative uncharacterized protein LPIN1 gene:ENSG00000134324 /// cDNA FLJ56073, highly similar to Lipin-1 gene:ENSG00000134324 /// Lipin-1 gene:ENSG00000134324 /// cDNA FLJ51642, highly similar to Lipin-1 gene:ENSG00000134324 /// Putative uncharacterized protein LPIN1 gene:ENSG00000134324 /// Homo sapiens lipin 1, mRNA (cDNA clone MGC:40396 IMAGE:3906281), complete cds. /// Homo sapiens lipin 1 (LPIN1), mRNA.	lipin 1	0.001923399	1.25332879

SPANXA1	Sperm protein associated with the nucleus on the X chromosome C gene:ENSG00000198573 /// Putative uncharacterized protein SPANXC gene:ENSG00000198573 /// Sperm protein associated with the nucleus on the X chromosome A gene:ENSG00000198021 /// Putative uncharacterized protein SPANXA2 gene:ENSG00000198021 /// Sperm protein associated with the nucleus on the X chromosome A gene:ENSG00000203926 /// Putative uncharacterized protein SPANXA2 gene:ENSG00000203926 /// Sperm protein associated with the nucleus on the X chromosome D gene:ENSG00000196406 /// Homo sapiens nuclear-associated protein SPAN-Xa (SPANX) mRNA, complete cds. /// Homo sapiens SPANX family, member E, mRNA (cDNA clone MGC:71908 IMAGE:4047937), complete cds. /// Homo sapiens SPANX family, member D, mRNA (cDNA clone MGC:119769 IMAGE:40013988), complete cds. /// Homo sapiens SPANX family, member A2 (SPANXA2), mRNA. /// Homo sapiens sperm protein associated with the nucleus, X-linked, family member A1 (SPANXA1), mRNA. /// Homo sapiens SPANX family, member D (SPANXD), mRNA. /// Homo sapiens SPANX family, member C (SPANXC), mRNA. /// Homo sapiens SPANX family, member E (SPANXE), mRNA.	sperm protein associated with the nucleus, X-linked, family member A1 /// SPANX family, member A2 /// SPANX family, member E /// SPANX family, member D /// SPANX family, member C	7.10E-06	-1.428106945

SPANXC	Sperm protein associated with the nucleus on the X chromosome C gene:ENSG00000198573 /// Putative uncharacterized protein SPANXC gene:ENSG00000198573 /// Sperm protein associated with the nucleus on the X chromosome A gene:ENSG00000198021 /// Putative uncharacterized protein SPANXA2 gene:ENSG00000198021 /// Sperm protein associated with the nucleus on the X chromosome A gene:ENSG00000203926 /// Putative uncharacterized protein SPANXA2 gene:ENSG00000203926 /// Sperm protein associated with the nucleus on the X chromosome D gene:ENSG00000196406 /// Homo sapiens nuclear-associated protein SPAN-Xa (SPANX) mRNA, complete cds. /// Homo sapiens SPANX family, member E, mRNA (cDNA clone MGC:71908 IMAGE:4047937), complete cds. /// Homo sapiens SPANX family, member D, mRNA (cDNA clone MGC:119769 IMAGE:40013988), complete cds. /// Homo sapiens SPANX family, member A2 (SPANXA2), mRNA. /// Homo sapiens sperm protein associated with the nucleus, X-linked, family member A1 (SPANXA1), mRNA. /// Homo sapiens SPANX family, member D (SPANXD), mRNA. /// Homo sapiens SPANX family, member C (SPANXC), mRNA. /// Homo sapiens SPANX family, member E (SPANXE), mRNA.	sperm protein associated with the nucleus, X-linked, family member A1 /// SPANX family, member A2 /// SPANX family, member E /// SPANX family, member D /// SPANX family, member C	7.10E-06	-1.428106945

SPANXE	Sperm protein associated with the nucleus on the X chromosome D gene:ENSG00000196406 /// Homo sapiens SPANX family, member E, mRNA (cDNA clone MGC:12501 IMAGE:3935644), complete cds. /// Homo sapiens SPANX family, member D, mRNA (cDNA clone MGC:150331 IMAGE:40119569), complete cds. /// Homo sapiens SPANX family, member D (SPANXD), mRNA. /// Homo sapiens SPANX family, member E (SPANXE), mRNA.	SPANX family, member E /// SPANX family, member D	3.23E-06	-1.458595391
SPANXD	Sperm protein associated with the nucleus on the X chromosome D gene:ENSG00000196406 /// Homo sapiens SPANX family, member E, mRNA (cDNA clone MGC:12501 IMAGE:3935644), complete cds. /// Homo sapiens SPANX family, member D, mRNA (cDNA clone MGC:150331 IMAGE:40119569), complete cds. /// Homo sapiens SPANX family, member D (SPANXD), mRNA. /// Homo sapiens SPANX family, member E (SPANXE), mRNA.	SPANX family, member E /// SPANX family, member D	3.23E-06	-1.458595391

PPP2R5D	Isoform Delta-1 of Serine/threonine-protein phosphatase 2A 56 kDa regulatory subunit delta isoform gene:ENSG00000112640 /// Isoform Delta-3 of Serine/threonine-protein phosphatase 2A 56 kDa regulatory subunit delta isoform gene:ENSG00000112640 /// Homo sapiens cDNA FLJ55863 complete cds, highly similar to Serine/threonine-protein phosphatase 2A 56 kDa regulatory subunit delta isoform. /// Homo sapiens protein phosphatase 2, regulatory subunit B', delta isoform (PPP2R5D), transcript variant 1, mRNA. /// Homo sapiens protein phosphatase 2, regulatory subunit B', delta isoform (PPP2R5D), transcript variant 2, mRNA. /// Homo sapiens protein phosphatase 2, regulatory subunit B', delta isoform (PPP2R5D), transcript variant 3, mRNA.	protein phosphatase 2, regulatory subunit B', delta isoform	0.007509104	1.149557844
C7orf11	TTD non-photosensitive 1 protein gene:ENSG00000168303 /// Homo sapiens cDNA FLJ76008 complete cds. /// Homo sapiens chromosome 7 open reading frame 11 (C7orf11), mRNA.	chromosome 7 open reading frame 11	0.005149876	1.190751951
DACT1	60 kDa protein gene:ENSG00000165617 /// Dapper homolog 1 gene:ENSG00000165617 /// dapper 1 isoform 2 gene:ENSG00000165617 /// Homo sapiens heptacellular carcinoma novel gene-3 protein mRNA, complete cds. /// Homo sapiens dapper, antagonist of beta-catenin, homolog 1 (Xenopus laevis) (DACT1), transcript variant 2, mRNA. /// Homo sapiens dapper, antagonist of beta-catenin, homolog 1 (Xenopus laevis) (DACT1), transcript variant 1, mRNA.	dapper, antagonist of beta-catenin, homolog 1 (Xenopus laevis)	1.30555E-05	-1.286163469

TSNAX	Translin-associated protein X gene:ENSG00000116918 /// Translin-associated factor X, isoform CRA_a gene:ENSG00000116918 /// H.sapiens mRNA for translin associated protein X. /// Homo sapiens translin-associated factor X (TSNAX), mRNA.	translin-associated factor X	0.000995975	1.222170944
PNMA2	Paraneoplastic antigen Ma2 gene:ENSG00000171362 /// Homo sapiens paraneoplastic antigen MA2, mRNA (cDNA clone MGC:43166 IMAGE:5262276), complete cds. /// Homo sapiens paraneoplastic antigen MA2 (PNMA2), mRNA.	paraneoplastic antigen MA2	0.001727172	1.323093403
NPM3	Nucleoplasmin-3 gene:ENSG00000107833 /// Homo sapiens nucleophosmin/nucleoplasmin 3 (NPM3) mRNA, complete cds. /// Homo sapiens nucleophosmin/nucleoplasmin, 3 (NPM3), mRNA.	nucleophosmin/nucleoplasmin, 3	0.005508727	1.180459759
PTMA	Homo sapiens prothymosin a14 mRNA, complete cds. /// Homo sapiens fetal thymus prothymosin alpha mRNA, complete cds.	prothymosin, alpha pseudogene 7 /// prothymosin, alpha	0.003530362	1.126089959
UBASH3B	Ubiquitin associated and SH3 domain-containing protein B gene:ENSG00000154127 /// Homo sapiens ubiquitin associated and SH3 domain containing, B, mRNA (cDNA clone MGC:15437 IMAGE:2958242), complete cds. /// Homo sapiens ubiquitin associated and SH3 domain containing, B (UBASH3B), mRNA.	ubiquitin associated and SH3 domain containing, B	0.000424412	-1.285686466
PGAM5	Isoform 2 of Phosphoglycerate mutase family member 5 gene:ENSG00000176894 /// Homo sapiens phosphoglycerate mutase family member 5, mRNA (cDNA clone MGC:5352 IMAGE:3048106), complete cds. /// Homo sapiens phosphoglycerate mutase family member 5 (PGAM5), mRNA.	phosphoglycerate mutase family member 5	0.620267202	-1.023484193

MAGEA11	melanoma antigen family A, 11 isoform b gene:ENSG00000185247 /// Isoform 1 of Melanoma-associated antigen 11 gene:ENSG00000185247 /// Homo sapiens cDNA FLJ45952 fis, clone PLACE7009563, highly similar to Melanoma-associated antigen 11. /// Homo sapiens melanoma antigen family A, 11 (MAGEA11), transcript variant 1, mRNA. /// Homo sapiens melanoma antigen family A, 11 (MAGEA11), transcript variant 2, mRNA.	melanoma antigen family A, 11	0.005554017	1.331664483
FAM125A	Isoform 1 of Protein FAM125A gene:ENSG00000141971 /// Isoform 2 of Protein FAM125A gene:ENSG00000141971 /// Homo sapiens cDNA FLJ32495 fis, clone SKNSH2000163. /// Homo sapiens family with sequence similarity 125, member A (FAM125A), mRNA.	family with sequence similarity 125, member A	0.001402382	1.237034533
COPS7A	COP9 signalosome complex subunit 7a gene:ENSG00000111652 /// Homo sapiens COP9 constitutive photomorphogenic homolog subunit 7A (Arabidopsis), mRNA (cDNA clone MGC:19475 IMAGE:4025790), complete cds. /// Homo sapiens COP9 constitutive photomorphogenic homolog subunit 7A (Arabidopsis) (COPS7A), mRNA.	COP9 constitutive photomorphogenic homolog subunit 7A (Arabidopsis)	0.003162298	1.21724726
YPEL1	Protein yippee-like 1 gene:ENSG00000100027 /// Putative uncharacterized protein YPEL1 gene:ENSG00000100027 /// Homo sapiens yippee-like 1 (Drosophila), mRNA (cDNA clone MGC:26488 IMAGE:4826997), complete cds. /// Homo sapiens yippee-like 1 (Drosophila) (YPEL1), mRNA.	yippee-like 1 (Drosophila)	0.007113213	1.248356741

SPAG7	Sperm-associated antigen 7 gene:ENSG00000091640 /// Homo sapiens sperm associated antigen 7, mRNA (cDNA clone MGC:20134 IMAGE:4330461), complete cds. /// Homo sapiens sperm associated antigen 7 (SPAG7), mRNA.	sperm associated antigen 7	0.003398118	1.176136848
BRD3	Isoform 1 of Bromodomain-containing protein 3 gene:ENSG00000169925 /// 21 kDa protein gene:ENSG00000169925 /// Isoform 2 of Bromodomain-containing protein 3 gene:ENSG00000169925 /// Isoform 2 of Bromodomain-containing protein 3 gene:ENSG00000169925 /// Bromodomain containing 3 gene:ENSG00000169925 /// Homo sapiens bromodomain containing 3, mRNA (cDNA clone MGC:29612 IMAGE:4856840), complete cds. /// Homo sapiens bromodomain containing 3 (BRD3), mRNA.	bromodomain containing 3	0.002066872	1.198202243
IFI35	Isoform 2 of Interferon-induced 35 kDa protein gene:ENSG00000068079 /// Homo sapiens interferon-induced protein 35, mRNA (cDNA clone MGC:2935 IMAGE:3050452), complete cds. /// Homo sapiens interferon-induced protein 35 (IFI35), mRNA.	interferon-induced protein 35	0.008660787	1.22464887
IWS1	Isoform 1 of Protein IWS1 homolog gene:ENSG00000163166 /// cdna:known chromosome:NCBI36:2:127954875:128000533:-1 gene:ENSG00000163166 /// cdna:known chromosome:NCBI36:2:127979086:128000932:-1 gene:ENSG00000163166 /// Homo sapiens cDNA FLJ54017 complete cds, highly similar to IWS1 homolog. /// Homo sapiens IWS1 homolog (S. cerevisiae) (IWS1), mRNA.	IWS1 homolog (S. cerevisiae)	0.001302013	1.201789809

GPKOW	G patch domain and KOW motifs-containing protein gene:ENSG00000068394 /// Putative uncharacterized protein GPKOW gene:ENSG00000068394 /// G patch domain and KOW motifs-containing protein gene:ENSG00000068394 /// Homo sapiens G patch domain and KOW motifs, mRNA (cDNA clone MGC:3986 IMAGE:2822421), complete cds. /// Homo sapiens G patch domain and KOW motifs (GPKOW), mRNA.	G patch domain and KOW motifs	0.006703132	1.172773344
ANP32B	Isoform 1 of Acidic leucine-rich nuclear phosphoprotein 32 family member B gene:ENSG00000136938 /// Human silver-stainable protein SSP29 mRNA, complete cds. /// Homo sapiens acidic (leucine-rich) nuclear phosphoprotein 32 family, member B (ANP32B), mRNA.	acidic (leucine-rich) nuclear phosphoprotein 32 family, member B	4.44331E-05	1.309988011
SGOL2	cdna:known chromosome:NCBI36:2:201099135:201143110:1 gene:ENSG00000163535 /// Isoform 1 of Shugoshin-like 2 gene:ENSG00000163535 /// 145 kDa protein gene:ENSG00000163535 /// Homo sapiens shugoshin-like 2 (S. pombe), mRNA (cDNA clone MGC:102910 IMAGE:30383194), complete cds. /// Homo sapiens shugoshin-like 2 (S. pombe) (SGOL2), mRNA.	shugoshin-like 2 (S. pombe)	0.003419706	1.190295916
C6orf108	putative c-Myc-responsive isoform 2 gene:ENSG00000112667 /// c-Myc-responsive protein Rcl gene:ENSG00000112667 /// Homo sapiens RCL (Rcl) mRNA, complete cds. /// Homo sapiens chromosome 6 open reading frame 108 (C6orf108), transcript variant 2, mRNA. /// Homo sapiens chromosome 6 open reading frame 108 (C6orf108), transcript variant 1, mRNA.	chromosome 6 open reading frame 108	0.007549731	1.189410284

C17orf49	Uncharacterized potential DNA-binding protein C17orf49 gene:ENSG00000161939 /// Homo sapiens cDNA FLJ31238 fis, clone KIDNE2004864. /// Homo sapiens ribonuclease, RNase K, mRNA (cDNA clone MGC:48891 IMAGE:5588971), complete cds.	chromosome 17 open reading frame 49 /// ribonuclease, RNase K	0.000150309	1.246491795
**Functional Group 7**	**Transcript Description**	**Gene Title**	**Bayes.lnp**	**Fold**
HMG20A	Isoform 1 of High mobility group protein 20A gene:ENSG00000140382 /// Isoform 1 of High mobility group protein 20A gene:ENSG00000140382 /// Homo sapiens high-mobility group 20A, mRNA (cDNA clone MGC:8813 IMAGE:3908842), complete cds. /// Homo sapiens high-mobility group 20A (HMG20A), mRNA.	high-mobility group 20A	0.008452154	1.152143821
NSBP1	Nucleosome-binding protein 1 gene:ENSG00000198157 /// Nucleosomal binding protein 1 gene:ENSG00000198157 /// Nucleosomal binding protein 1 gene:ENSG00000198157 /// Nucleosome-binding protein 1 gene:ENSG00000198157 /// Homo sapiens cDNA FLJ36739 fis, clone UTERU2013262. /// Homo sapiens nucleosomal binding protein 1 (NSBP1), mRNA.	nucleosomal binding protein 1	0.008542632	1.222686506
HMGB2	High mobility group protein B2 gene:ENSG00000164104 /// Homo sapiens high-mobility group box 2, mRNA (cDNA clone MGC:110999 IMAGE:6163784), complete cds. /// Homo sapiens high-mobility group box 2 (HMGB2), transcript variant 1, mRNA. /// Homo sapiens high-mobility group box 2 (HMGB2), transcript variant 2, mRNA. /// Homo sapiens high-mobility group box 2 (HMGB2), transcript variant 3, mRNA.	high-mobility group box 2	0.003119943	1.181512538

HIST1H2AD	Histone H3.1 gene:ENSG00000197409 /// cDNA, FLJ92409, highly similar to Homo sapiens histone 1, H2ad (HIST1H2AD), mRNA gene:ENSG00000197409 /// Histone H2A type 1-D gene:ENSG00000197409 /// Homo sapiens histone cluster 1, H3d, mRNA (cDNA clone MGC:45668 IMAGE:3608479), complete cds. /// Homo sapiens histone cluster 1, H2ad, mRNA (cDNA clone MGC:120842 IMAGE:7939652), complete cds. /// Homo sapiens histone cluster 1, H2ad (HIST1H2AD), mRNA. /// Homo sapiens histone cluster 1, H3d (HIST1H3D), mRNA.	histone cluster 1, H3d /// histone cluster 1, H2ad	4.74E-05	1.303067009
RPA2	Isoform 1 of Replication protein A 32 kDa subunit gene:ENSG00000117748 /// Isoform 2 of Replication protein A 32 kDa subunit gene:ENSG00000117748 /// Isoform 3 of Replication protein A 32 kDa subunit gene:ENSG00000117748 /// Homo sapiens replication protein A2, 32kDa, mRNA (cDNA clone MGC:29683 IMAGE:4111194), complete cds. /// Homo sapiens replication protein A2, 32kDa (RPA2), mRNA.	replication protein A2, 32kDa	0.000372642	1.268836391
HMGN4	High mobility group nucleosome-binding domain-containing protein 4 gene:ENSG00000182952 /// High mobility group nucleosome-binding domain-containing protein 4 gene:ENSG00000182952 /// Homo sapiens high mobility group nucleosomal binding domain 4, mRNA (cDNA clone MGC:5145 IMAGE:3452950), complete cds. /// Homo sapiens high mobility group nucleosomal binding domain 4 (HMGN4), mRNA.	high mobility group nucleosomal binding domain 4	0.009913848	1.162668484

HMGN3	Isoform 1 of High mobility group nucleosome-binding domain-containing protein 3 gene:ENSG00000118418 /// 11 kDa protein gene:ENSG00000118418 /// Isoform 2 of High mobility group nucleosome-binding domain-containing protein 3 gene:ENSG00000118418 /// Homo sapiens TRIP7-like protein mRNA, complete cds. /// Homo sapiens high mobility group nucleosomal binding domain 3 (HMGN3), transcript variant 2, mRNA. /// Homo sapiens high mobility group nucleosomal binding domain 3 (HMGN3), transcript variant 1, mRNA.	high mobility group nucleosomal binding domain 3	0.002626091	1.19648497
HIST1H1C	Histone H1.2 gene:ENSG00000187837 /// Homo sapiens cDNA FLJ76039 complete cds, highly similar to Homo sapiens histone 1, H1c (HIST1H1C), mRNA. /// Homo sapiens histone cluster 1, H1c (HIST1H1C), mRNA.	histone cluster 1, H1c	0.00012822	1.284945067
HIST2H2AC	BolA-like protein 1 gene:ENSG00000178096 /// Histone H2A type 2-C gene:ENSG00000184260 /// Homo sapiens histone cluster 2, H2ac, mRNA (cDNA clone MGC:74460 IMAGE:3892650), complete cds. /// Homo sapiens histone cluster 2, H2ac (HIST2H2AC), mRNA.	histone cluster 2, H2ac	0.000191411	1.266224894

HIST2H2BF	Similar to Histone H2b gene:ENSG00000203851 /// Similar to Histone H2b gene:ENSG00000203819 /// Histone H3 gene:ENSG00000203818 /// Histone H2B type 2-F gene:ENSG00000203814 /// Homo sapiens cDNA FLJ56780 complete cds, highly similar to Histone H2B type 2-F. /// Homo sapiens cDNA FLJ56787 complete cds, highly similar to Histone H2B type 2-F. /// Homo sapiens histone cluster 2, H2bf, mRNA (cDNA clone MGC:131639 IMAGE:5224812), complete cds. /// Homo sapiens histone cluster 2, H2ba, mRNA (cDNA clone MGC:126029 IMAGE:40032203), complete cds. /// Homo sapiens histone cluster 2, H2bf (HIST2H2BF), mRNA.	histone cluster 1, H2bm /// histone cluster 2, H3, pseudogene 2 /// histone cluster 2, H2bf /// histone cluster 2, H2ba	0.000105 439	1.242569 531
HIST2H2AA 3	Histone H2A type 2-A gene:ENSG00000183558 /// Histone H2A type 2-A gene:ENSG00000203812 /// Homo sapiens histone H2A.2 mRNA, complete cds. /// Homo sapiens histone cluster 2, H2aa3 (HIST2H2AA3), mRNA. /// Homo sapiens histone cluster 2, H2aa4 (HIST2H2AA4), mRNA.	histone cluster 2, H2aa3 /// histone cluster 2, H2aa4	0.000274 373	1.222531 456
HIST1H2BM	Histone H2B type 1-M gene:ENSG00000196374 /// Homo sapiens histone cluster 1, H2bm, mRNA (cDNA clone MGC:79335 IMAGE:7002058), complete cds. /// Homo sapiens histone cluster 1, H2bm (HIST1H2BM), mRNA.	histone cluster 1, H2bm	3.6798E-05	1.289974 724
HIST1H1E	Histone H1.4 gene:ENSG00000168298 /// Homo sapiens histone cluster 1, H1e, mRNA (cDNA clone MGC:116818 IMAGE:40003408), complete cds. /// Homo sapiens histone cluster 1, H1e (HIST1H1E), mRNA.	histone cluster 1, H1e	6.77285E -05	1.274697 578

HIST2H3A	Histone H3 gene:ENSG00000203818 /// Histone H3.2 gene:ENSG00000183598 /// Histone H3.2 gene:ENSG00000183598 /// Histone H3.2 gene:ENSG00000203811 /// Histone H3.2 gene:ENSG00000203852 /// Homo sapiens histone cluster 2, H3c, mRNA (cDNA clone MGC:104061 IMAGE:30915509), complete cds. /// Homo sapiens histone cluster 2, H3c (HIST2H3C), mRNA. /// Homo sapiens histone cluster 2, H3a (HIST2H3A), mRNA. /// Homo sapiens histone cluster 2, H3d (HIST2H3D), mRNA.	histone cluster 2, H3c /// histone cluster 2, H3a /// histone cluster 2, H3d	7.08E-06	1.290707904
HIST3H2A	Histone H2A type 3 gene:ENSG00000181218 /// Homo sapiens histone cluster 3, H2a (HIST3H2A), mRNA.	histone cluster 3, H2a	0.001136069	1.31228178
HIST1H3J	Histone H3.1 gene:ENSG00000197153 /// Homo sapiens histone cluster 1, H3j (HIST1H3J), mRNA.	histone cluster 1, H3j	6.05293E-05	1.346160535
HIST2H3PS2	Similar to Histone H2b gene:ENSG00000203851 /// Similar to Histone H2b gene:ENSG00000203819 /// Histone H3 gene:ENSG00000203818 /// Histone H2B type 2-F gene:ENSG00000203814 /// Homo sapiens cDNA FLJ56780 complete cds, highly similar to Histone H2B type 2-F. /// Homo sapiens cDNA FLJ56787 complete cds, highly similar to Histone H2B type 2-F. /// Homo sapiens histone cluster 2, H2bf, mRNA (cDNA clone MGC:131639 IMAGE:5224812), complete cds. /// Homo sapiens histone cluster 2, H2ba, mRNA (cDNA clone MGC:126029 IMAGE:40032203), complete cds. /// Homo sapiens histone cluster 2, H2bf (HIST2H2BF), mRNA.	histone cluster 1, H2bm /// histone cluster 2, H3, pseudogene 2 /// histone cluster 2, H2bf /// histone cluster 2, H2ba	0.000105439	1.242569531

HIST1H2BL	Histone H2B type 1-L gene:ENSG00000185130 /// Homo sapiens histone cluster 1, H2bl (HIST1H2BL), mRNA.	histone cluster 1, H2bl	0.003136722	1.301821445
HIST1H1B	Histone H1.5 gene:ENSG00000184357 /// Homo sapiens histone cluster 1, H1b, mRNA (cDNA clone MGC:95376 IMAGE:7216915), complete cds. /// Homo sapiens histone cluster 1, H1b (HIST1H1B), mRNA.	histone cluster 1, H1b	4.48375E-05	1.359470401
HIST2H2AB	Histone H2A type 2-B gene:ENSG00000184270 /// Homo sapiens histone cluster 2, H2ab (HIST2H2AB), mRNA.	histone cluster 2, H2ab	3.93892E-05	1.351416002
HIST1H2BK	cDNA FLJ56780, highly similar to Histone H2B type 2-F gene:ENSG00000197903 /// Homo sapiens histone cluster 1, H2bk, mRNA (cDNA clone MGC:131989 IMAGE:4707078), complete cds. /// Homo sapiens histone cluster 1, H2bk (HIST1H2BK), mRNA.	histone cluster 1, H2bk	2.72625E-11	-2.243269001
HIST1H2BN	Histone H2B type 1-N gene:ENSG00000220323 /// Histone H2B type 1-N gene:ENSG00000220323 /// Homo sapiens histone cluster 1, H2bn, mRNA (cDNA clone MGC:125415 IMAGE:40021693), complete cds. /// Homo sapiens histone cluster 1, H2bn (HIST1H2BN), mRNA.	histone cluster 1, H2bn	0.000468844	1.312754607
HIST1H2BH	Histone H2B type 1-H gene:ENSG00000197459 /// Homo sapiens histone cluster 1, H2bh, mRNA (cDNA clone MGC:116762 IMAGE:40002316), complete cds. /// Homo sapiens histone cluster 1, H2bh (HIST1H2BH), mRNA.	histone cluster 1, H2bh	4.97124E-05	1.263745617

HIST2H2BA	Similar to Histone H2b gene:ENSG00000203851 /// Similar to Histone H2b gene:ENSG00000203819 /// Histone H3 gene:ENSG00000203818 /// Histone H2B type 2-F gene:ENSG00000203814 /// Homo sapiens cDNA FLJ56780 complete cds, highly similar to Histone H2B type 2-F. /// Homo sapiens cDNA FLJ56787 complete cds, highly similar to Histone H2B type 2-F. /// Homo sapiens histone cluster 2, H2bf, mRNA (cDNA clone MGC:131639 IMAGE:5224812), complete cds. /// Homo sapiens histone cluster 2, H2ba, mRNA (cDNA clone MGC:126029 IMAGE:40032203), complete cds. /// Homo sapiens histone cluster 2, H2bf (HIST2H2BF), mRNA.	histone cluster 1, H2bm /// histone cluster 2, H3, pseudogene 2 /// histone cluster 2, H2bf /// histone cluster 2, H2ba	0.000105439	1.242569531
H2BFS	cDNA FLJ56780, highly similar to Histone H2B type 2-F gene:ENSG00000197903 /// cDNA FLJ56780, highly similar to Histone H2B type 2-F gene:ENSG00000197903 /// Homo sapiens histone cluster 1, H2bk, mRNA (cDNA clone MGC:131989 IMAGE:4707078), complete cds. /// Homo sapiens H2B histone family, member S, mRNA (cDNA clone MGC:161617 IMAGE:8992055), complete cds. /// Homo sapiens histone cluster 1, H2bk (HIST1H2BK), mRNA.	histone cluster 1, H2bk /// H2B histone family, member S	0.00187647	1.218709719

H2AFY	H2A histone family, member Y isoform 2 gene:ENSG00000113648 /// Isoform 2 of Core histone macro-H2A.1 gene:ENSG00000113648 /// Isoform 1 of Core histone macro-H2A.1 gene:ENSG00000113648 /// H2A histone family, member Y isoform 2 gene:ENSG00000113648 /// Homo sapiens H2A histone family, member Y, mRNA (cDNA clone MGC:110894 IMAGE:30348703), complete cds. /// Homo sapiens H2A histone family, member Y (H2AFY), transcript variant 3, mRNA. /// Homo sapiens H2A histone family, member Y (H2AFY), transcript variant 4, mRNA. /// Homo sapiens H2A histone family, member Y (H2AFY), transcript variant 2, mRNA. /// Homo sapiens H2A histone family, member Y (H2AFY), transcript variant 1, mRNA.	H2A histone family, member Y	1.00695E-08	1.635137938
HIST1H1D	Histone H1.3 gene:ENSG00000124575 /// Homo sapiens histone cluster 1, H1d, mRNA (cDNA clone MGC:132534 IMAGE:8143877), complete cds. /// Homo sapiens histone cluster 1, H1d (HIST1H1D), mRNA.	histone cluster 1, H1d	0.000117414	1.309558265
HIST2H3D	Histone H3 gene:ENSG00000203818 /// Histone H3 gene:ENSG00000203818 /// Histone H3.2 gene:ENSG00000183598 /// Histone H3.2 gene:ENSG00000183598 /// Homo sapiens histone cluster 2, H3d (HIST2H3D), mRNA.	histone cluster 2, H3d	0.000108608	1.287768241

HIST2H4B	Histone H4 gene:ENSG00000183941 /// Histone H4 gene:ENSG00000183941 /// Histone H4 gene:ENSG00000183941 /// Histone H4 gene:ENSG00000182217 /// Histone H4 gene:ENSG00000182217 /// Histone H4 gene:ENSG00000182217 /// Homo sapiens histone cluster 2, H4b, mRNA (cDNA clone IMAGE:5113140). /// Homo sapiens histone cluster 2, H4b (HIST2H4B), mRNA. /// Homo sapiens histone cluster 2, H4a (HIST2H4A), mRNA.	histone cluster 2, H4a /// histone cluster 2, H4b	1.07E-05	-1.411322045
**Functional Group 8**	**Transcript Description**	**Gene Title**	**Bayes.lnp**	**Fold**
PTGS2	Prostaglandin G/H synthase 2 gene:ENSG00000073756 /// Homo sapiens cyclooxygenase 2b mRNA, complete cds; alternatively spliced. /// Homo sapiens prostaglandin-endoperoxide synthase 2 (prostaglandin G/H synthase and cyclooxygenase) (PTGS2), mRNA.	prostaglandin-endoperoxide synthase 2 (prostaglandin G/H synthase and cyclooxygenase)	0.000943863	-1.404109712
SDF2	Stromal cell-derived factor 2 gene:ENSG00000132581 /// Homo sapiens stromal cell-derived factor 2, mRNA (cDNA clone MGC:2977 IMAGE:3140716), complete cds. /// Homo sapiens stromal cell-derived factor 2 (SDF2), mRNA.	stromal cell-derived factor 2	0.002266752	1.187169025
PSEN2	Isoform 1 of Presenilin-2 gene:ENSG00000143801 /// Isoform 2 of Presenilin-2 gene:ENSG00000143801 /// Presenilin 2 gene:ENSG00000143801 /// Presenilin 2 gene:ENSG00000143801 /// Homo sapiens presenilin 2 (Alzheimer disease 4), mRNA (cDNA clone MGC:12801 IMAGE:4131408), complete cds. /// Homo sapiens presenilin 2 (Alzheimer disease 4) (PSEN2), transcript variant 2, mRNA. /// Homo sapiens presenilin 2	presenilin 2 (Alzheimer disease 4)	0.002091012	-1.214994292

	(Alzheimer disease 4) (PSEN2), transcript variant 1, mRNA.			
SPCS1	signal peptidase complex subunit 1 homolog gene:ENSG00000114902 /// Homo sapiens signal peptidase complex subunit 1 homolog (S. cerevisiae), mRNA (cDNA clone MGC:4899 IMAGE:3462307), complete cds. /// Homo sapiens signal peptidase complex subunit 1 homolog (S. cerevisiae) (SPCS1), mRNA.	signal peptidase complex subunit 1 homolog (S. cerevisiae)	0.000448454	1.229641641
PRKAB2	5'-AMP-activated protein kinase subunit beta-2 gene:ENSG00000131791 /// Homo sapiens protein kinase, AMP-activated, beta 2 non-catalytic subunit, mRNA (cDNA clone MGC:61468 IMAGE:6091476), complete cds. /// Homo sapiens protein kinase, AMP-activated, beta 2 non-catalytic subunit (PRKAB2), mRNA.	protein kinase, AMP-activated, beta 2 non-catalytic subunit	7.74101E-05	-1.244887053

B3GALT5	Beta-1,3-galactosyltransferase 5 gene:ENSG00000183778 /// Beta-1,3-galactosyltransferase 5 gene:ENSG00000183778 /// Beta-1,3-galactosyltransferase 5 gene:ENSG00000183778 /// Beta-1,3-galactosyltransferase 5 gene:ENSG00000183778 /// Homo sapiens beta-1,3-galactosyltransferase 5 (B3GALT5) mRNA, complete cds. /// Homo sapiens UDP-Gal:betaGlcNAc beta 1,3-galactosyltransferase, polypeptide 5 (B3GALT5), transcript variant 2, mRNA. /// Homo sapiens UDP-Gal:betaGlcNAc beta 1,3-galactosyltransferase, polypeptide 5 (B3GALT5), transcript variant 3, mRNA. /// Homo sapiens UDP-Gal:betaGlcNAc beta 1,3-galactosyltransferase, polypeptide 5 (B3GALT5), transcript variant 1, mRNA. /// Homo sapiens UDP-Gal:betaGlcNAc beta 1,3-galactosyltransferase, polypeptide 5 (B3GALT5), transcript variant 5, mRNA. /// Homo sapiens UDP-Gal:betaGlcNAc beta 1,3-galactosyltransferase, polypeptide 5 (B3GALT5), transcript variant 4, mRNA.	UDP-Gal:betaGlcNAc beta 1,3-galactosyltransferase, polypeptide 5	0.00398875	-1.210837361
PIGU	Isoform 1 of Phosphatidylinositol glycan anchor biosynthesis class U protein gene:ENSG00000101464 /// Isoform 2 of Phosphatidylinositol glycan anchor biosynthesis class U protein gene:ENSG00000101464 /// Homo sapiens transamidase complex subunit PIG-U (PIGU) mRNA, complete cds. /// Homo sapiens phosphatidylinositol glycan anchor biosynthesis, class U (PIGU), mRNA.	phosphatidylinositol glycan anchor biosynthesis, class U	0.001502803	1.20150259

GALNT11	Isoform 1 of Polypeptide N-acetylgalactosaminyltransferase 11 gene:ENSG00000178234 /// Homo sapiens cDNA FLJ42944 fis, clone BRSTN2004863, highly similar to PolypeptideN-acetylgalactosaminyltransferase 11 (EC 2.4.1.41). /// Homo sapiens UDP-N-acetyl-alpha-D-galactosamine:polypeptide N-acetylgalactosaminyltransferase 11 (GalNAc-T11) (GALNT11), mRNA.	UDP-N-acetyl-alpha-D-galactosamine:polypeptide N-acetylgalactosaminyltransferase 11 (GalNAc-T11)	6.29581E-06	-1.315071526
A4GALT	Lactosylceramide 4-alpha-galactosyltransferase gene:ENSG00000128274 /// Lactosylceramide 4-alpha-galactosyltransferase gene:ENSG00000128274 /// Lactosylceramide 4-alpha-galactosyltransferase gene:ENSG00000128274 /// Lactosylceramide 4-alpha-galactosyltransferase gene:ENSG00000128274 /// Homo sapiens alpha 1,4-galactosyltransferase, mRNA (cDNA clone MGC:9631 IMAGE:3913851), complete cds. /// Homo sapiens alpha 1,4-galactosyltransferase (A4GALT), mRNA.	alpha 1,4-galactosyltransferase	0.006056806	-1.23334869
GALNT5	Polypeptide N-acetylgalactosaminyltransferase 5 gene:ENSG00000136542 /// Homo sapiens UDP-N-acetyl-alpha-D-galactosamine:polypeptide N-acetylgalactosaminyltransferase 5 (GalNAc-T5), mRNA (cDNA clone MGC:164948 IMAGE:40148207), complete cds. /// Homo sapiens UDP-N-acetyl-alpha-D-galactosamine:polypeptide N-acetylgalactosaminyltransferase 5 (GalNAc-T5) (GALNT5), mRNA.	UDP-N-acetyl-alpha-D-galactosamine:polypeptide N-acetylgalactosaminyltransferase 5 (GalNAc-T5)	0.002734554	-1.341674351

SAR1B	GTP-binding protein SAR1b gene:ENSG00000152700 /// GTP-binding protein SAR1b gene:ENSG00000152700 /// GTP-binding protein SAR1b gene:ENSG00000152700 /// Homo sapiens GTP binding protein mRNA, complete cds. /// Homo sapiens SAR1 homolog B (S. cerevisiae) (SAR1B), transcript variant 2, mRNA. /// Homo sapiens SAR1 homolog B (S. cerevisiae) (SAR1B), transcript variant 1, mRNA.	SAR1 homolog B (S. cerevisiae)	0.007631315	-1.149369021
HMGCS1	Hydroxymethylglutaryl-CoA synthase, cytoplasmic gene:ENSG00000112972 /// Homo sapiens 3-hydroxy-3-methylglutaryl-Coenzyme A synthase 1 (soluble), mRNA (cDNA clone MGC:90332 IMAGE:4443624), complete cds. /// Homo sapiens 3-hydroxy-3-methylglutaryl-Coenzyme A synthase 1 (soluble) (HMGCS1), transcript variant 1, mRNA. /// Homo sapiens 3-hydroxy-3-methylglutaryl-Coenzyme A synthase 1 (soluble) (HMGCS1), transcript variant 2, mRNA.	3-hydroxy-3-methylglutaryl-Coenzyme A synthase 1 (soluble)	0.007339614	1.183424159
ALG6	Dolichyl pyrophosphate Man9GlcNAc2 alpha-1,3-glucosyltransferase gene:ENSG00000088035 /// Dolichyl pyrophosphate Man9GlcNAc2 alpha-1,3-glucosyltransferase gene:ENSG00000088035 /// Asparagine-linked glycosylation 6 homolog gene:ENSG00000088035 /// Homo sapiens dolichyl-P-Glc:Man9GlcNAc2-PP-dolichyl glucosyltransferase (ALG6) mRNA, complete cds. /// Homo sapiens asparagine-linked glycosylation 6 homolog (S. cerevisiae, alpha-1,3-glucosyltransferase) (ALG6), mRNA.	asparagine-linked glycosylation 6, alpha-1,3-glucosyltransferase homolog (S. cerevisiae)	0.004797828	1.180850212

ERO1L	Putative uncharacterized protein ERO1L gene:ENSG00000197930 /// ERO1-like protein alpha gene:ENSG00000197930 /// Homo sapiens ERO1-like protein (ERO1-L) mRNA, complete cds. /// Homo sapiens ERO1-like (S. cerevisiae) (ERO1L), mRNA.	ERO1-like (S. cerevisiae)	5.9695E-05	1.258963743
CYP1B1	Cytochrome P450 1B1 gene:ENSG00000138061 /// Cytochrome P450 1B1 gene:ENSG00000138061 /// Human dioxin-inducible cytochrome P450 (CYP1B1) mRNA, complete cds. /// Homo sapiens cytochrome P450, family 1, subfamily B, polypeptide 1 (CYP1B1), mRNA.	cytochrome P450, family 1, subfamily B, polypeptide 1	0.003705431	1.319220391
IDI1	isopentenyl-diphosphate delta isomerase gene:ENSG00000067064 /// Homo sapiens isopentenyl-diphosphate delta isomerase 1, mRNA (cDNA clone MGC:71798 IMAGE:30337530), complete cds. /// Homo sapiens isopentenyl-diphosphate delta isomerase 1 (IDI1), mRNA.	isopentenyl-diphosphate delta isomerase 1	0.000835945	1.184844529
SSR4	Translocon-associated protein subunit delta precursor gene:ENSG00000180879 /// Translocon-associated protein subunit delta precursor gene:ENSG00000180879 /// Translocon-associated protein subunit delta precursor gene:ENSG00000180879 /// Putative uncharacterized protein SSR4 gene:ENSG00000180879 /// Homo sapiens signal sequence receptor, delta (translocon-associated protein delta), mRNA (cDNA clone MGC:5151 IMAGE:2900758), complete cds. /// Homo sapiens signal sequence receptor, delta (translocon-associated protein delta) (SSR4), mRNA.	signal sequence receptor, delta (translocon-associated protein delta)	0.009030827	1.182949288

FDPS	Farnesyl pyrophosphate synthetase gene:ENSG00000160752 /// Farnesyl pyrophosphate synthetase gene:ENSG00000160752 /// Homo sapiens farnesyl diphosphate synthase (farnesyl pyrophosphate synthetase, dimethylallyltranstransferase, geranyltranstransferase), mRNA (cDNA clone MGC:15352 IMAGE:4132071), complete cds. /// Homo sapiens farnesyl diphosphate synthase (farnesyl pyrophosphate synthetase, dimethylallyltranstransferase, geranyltranstransferase) (FDPS), transcript variant 3, mRNA. /// Homo sapiens farnesyl diphosphate synthase (farnesyl pyrophosphate synthetase, dimethylallyltranstransferase, geranyltranstransferase) (FDPS), transcript variant 1, mRNA. /// Homo sapiens farnesyl diphosphate synthase (farnesyl pyrophosphate synthetase, dimethylallyltranstransferase, geranyltranstransferase) (FDPS), transcript variant 2, mRNA.	farnesyl diphosphate synthase (farnesyl pyrophosphate synthetase, dimethylallyltranstransferase, geranyltranstransferase)	0.001186871	1.181324614
FDFT1	Squalene synthetase gene:ENSG00000079459 /// Homo sapiens farnesyl-diphosphate farnesyltransferase 1, mRNA (cDNA clone MGC:4480 IMAGE:2961623), complete cds. /// Homo sapiens farnesyl-diphosphate farnesyltransferase 1 (FDFT1), mRNA.	farnesyl-diphosphate farnesyltransferase 1	0.004783371	1.170365198
UBE2J1	Ubiquitin-conjugating enzyme E2 J1 gene:ENSG00000198833 /// Homo sapiens mRNA full length insert cDNA clone EUROIMAGE 1658099. /// Homo sapiens ubiquitin-conjugating enzyme E2, J1 (UBC6 homolog, yeast) (UBE2J1), mRNA.	ubiquitin-conjugating enzyme E2, J1 (UBC6 homolog, yeast)	9.10548E-05	1.323235774

PIGV	GPI mannosyltransferase 2 gene:ENSG00000060642 /// GPI mannosyltransferase 2 gene:ENSG00000060642 /// Homo sapiens phosphatidylinositol glycan anchor biosynthesis, class V, mRNA (cDNA clone MGC:8856 IMAGE:3890907), complete cds. /// Homo sapiens phosphatidylinositol glycan anchor biosynthesis, class V (PIGV), mRNA.	phosphatidylinositol glycan anchor biosynthesis, class V	0.003604587	1.223380356
LMAN2L	Isoform 2 of VIP36-like protein gene:ENSG00000114988 /// Isoform 1 of VIP36-like protein gene:ENSG00000114988 /// Homo sapiens clone DNA50911 AATL368 (UNQ368) mRNA, complete cds. /// Homo sapiens lectin, mannose-binding 2-like (LMAN2L), mRNA.	lectin, mannose-binding 2-like	0.007512864	1.166835736
NDST1	Isoform 1 of Bifunctional heparan sulfate N-deacetylase/N-sulfotransferase 1 gene:ENSG00000070614 /// Human heparan sulfate-N-deacetylase/N-sulfotransferase mRNA, clone HSST, complete cds. /// Homo sapiens N-deacetylase/N-sulfotransferase (heparan glucosaminyl) 1 (NDST1), mRNA.	N-deacetylase/N-sulfotransferase (heparan glucosaminyl) 1	0.004813946	1.154625255
CHST9	GalNAc-4-sulfotransferase 2 gene:ENSG00000154080 /// Homo sapiens clone DNA108682 CHST9 (UNQ2549) mRNA, complete cds. /// Homo sapiens carbohydrate (N-acetylgalactosamine 4-0) sulfotransferase 9 (CHST9), mRNA.	carbohydrate (N-acetylgalactosamine 4-0) sulfotransferase 9	0.001430994	-1.282843659
EBP	3-beta-hydroxysteroid-Delta(8),Delta(7)-isomerase gene:ENSG00000147155 /// Homo sapiens emopamil binding protein (sterol isomerase), mRNA (cDNA clone MGC:29952 IMAGE:5106272), complete cds. /// Homo sapiens emopamil binding protein (sterol isomerase) (EBP), mRNA.	emopamil binding protein (sterol isomerase)	2.22369E-05	1.291827881

MECR	trans-2-enoyl-CoA reductase, mitochondrial isoform b gene:ENSG00000116353 /// Mitochondrial trans-2-enoyl-CoA reductase gene:ENSG00000116353 /// trans-2-enoyl-CoA reductase, mitochondrial isoform b gene:ENSG00000116353 /// Trans-2-enoyl-CoA reductase, mitochondrial gene:ENSG00000116353 /// Homo sapiens CGI-63 protein mRNA, complete cds. /// Homo sapiens mitochondrial trans-2-enoyl-CoA reductase (MECR), nuclear gene encoding mitochondrial protein, transcript variant 1, mRNA. /// Homo sapiens mitochondrial trans-2-enoyl-CoA reductase (MECR), nuclear gene encoding mitochondrial protein, transcript variant 2, mRNA.	mitochondrial trans-2-enoyl-CoA reductase	0.002235 35	1.183629 316
DHCR7	7-dehydrocholesterol reductase gene:ENSG00000172893 /// 7-dehydrocholesterol reductase gene:ENSG00000172893 /// Homo sapiens delta7-sterol reductase mRNA, complete cds. /// Homo sapiens 7-dehydrocholesterol reductase (DHCR7), mRNA.	7-dehydrocholesterol reductase	0.000110 779	1.266709 411
CYP51A1	cytochrome P450, family 51 gene:ENSG00000001630 /// Human lanosterol 14-demethylase cytochrome P450 (CYP51) mRNA, complete cds. /// Homo sapiens cytochrome P450, family 51, subfamily A, polypeptide 1 (CYP51A1), mRNA.	cytochrome P450, family 51, subfamily A, polypeptide 1	0.000529 737	1.229132 469
SQLE	Squalene monooxygenase gene:ENSG00000104549 /// Homo sapiens mRNA for squalene epoxidase, complete cds. /// Homo sapiens squalene epoxidase (SQLE), mRNA.	squalene epoxidase	0.009875 803	1.130439 884

FADS2	Isoform 2 of Fatty acid desaturase 2 gene:ENSG00000134824 /// Isoform 1 of Fatty acid desaturase 2 gene:ENSG00000134824 /// Homo sapiens p5327 mRNA, complete cds. /// Homo sapiens fatty acid desaturase 2 (FADS2), mRNA.	fatty acid desaturase 2	0.008103918	1.142409322
FADS3	Fatty acid desaturase 3 gene:ENSG00000149485 /// Homo sapiens fatty acid desaturase 3 (FADS3) mRNA, complete cds. /// Homo sapiens fatty acid desaturase 3 (FADS3), mRNA.	fatty acid desaturase 3	0.000260387	-1.226671196
DERL1	Derlin-1 gene:ENSG00000136986 /// cdna:known chromosome:NCBI36:8:124095691:124096380:-1 gene:ENSG00000220455 /// Homo sapiens clone DNA16435 SDIG243 (UNQ243) mRNA, complete cds. /// Homo sapiens Der1-like domain family, member 1 (DERL1), transcript variant 1, mRNA. /// Homo sapiens Der1-like domain family, member 1 (DERL1), transcript variant 2, mRNA.	Der1-like domain family, member 1	0.008077664	1.136355696
PECR	Peroxisomal trans-2-enoyl-CoA reductase gene:ENSG00000115425 /// Homo sapiens peroxisomal trans 2-enoyl CoA reductase mRNA, complete cds. /// Homo sapiens peroxisomal trans-2-enoyl-CoA reductase (PECR), mRNA.	peroxisomal trans-2-enoyl-CoA reductase	0.000520923	1.345997976
ALOX5	Arachidonate 5-lipoxygenase variant (Fragment) gene:ENSG00000215136 /// Arachidonate 5-lipoxygenase gene:ENSG00000012779 /// Human 5-lipoxygenase mRNA, complete cds. /// Homo sapiens arachidonate 5-lipoxygenase (ALOX5), mRNA.	arachidonate 5-lipoxygenase	2.36753E-06	-1.569368707

MGST2	Microsomal glutathione S-transferase 2 gene:ENSG00000085871 /// Homo sapiens microsomal glutathione S-transferase 2, mRNA (cDNA clone MGC:14097 IMAGE:3937750), complete cds. /// Homo sapiens microsomal glutathione S-transferase 2 (MGST2), mRNA.	microsomal glutathione S-transferase 2	8.47008E-06	1.513497757
FKTN	Fukutin gene:ENSG00000106692 /// 26 kDa protein gene:ENSG00000106692 /// 27 kDa protein gene:ENSG00000106692 /// Homo sapiens FCMD mRNA for fukutin, complete cds. /// Homo sapiens fukutin (FKTN), transcript variant 2, mRNA. /// Homo sapiens fukutin (FKTN), transcript variant 1, mRNA.	fukutin	0.003004349	1.219800207
ELOVL1	Elongation of very long chain fatty acids protein 1 gene:ENSG00000066322 /// Homo sapiens elongation of very long chain fatty acids (FEN1/Elo2, SUR4/Elo3, yeast)-like 1, mRNA (cDNA clone MGC:701 IMAGE:3347272), complete cds. /// Homo sapiens elongation of very long chain fatty acids (FEN1/Elo2, SUR4/Elo3, yeast)-like 1 (ELOVL1), mRNA.	elongation of very long chain fatty acids (FEN1/Elo2, SUR4/Elo3, yeast)-like 1	0.00075859	1.232326481
ANTXR2	Isoform 4 of Anthrax toxin receptor 2 gene:ENSG00000163297 /// ANTXR2 protein gene:ENSG00000163297 /// Isoform 4 of Anthrax toxin receptor 2 gene:ENSG00000163297 /// Isoform 1 of Anthrax toxin receptor 2 gene:ENSG00000163297 /// Isoform 2 of Anthrax toxin receptor 2 gene:ENSG00000163297 /// Isoform 3 of Anthrax toxin receptor 2 gene:ENSG00000163297 /// Homo sapiens cDNA FLJ31074 fis, clone HSYRA2001476. /// Homo sapiens anthrax toxin receptor 2 (ANTXR2), mRNA.	anthrax toxin receptor 2	0.000582301	-1.248326997

LASS5	LAG1 longevity assurance homolog 5 gene:ENSG00000139624 /// cDNA FLJ59438, highly similar to LAG1 longevity assurance homolog 5 gene:ENSG00000139624 /// Homo sapiens LAG1 homolog, ceramide synthase 5, mRNA (cDNA clone IMAGE:4826780), complete cds. /// Homo sapiens LAG1 homolog, ceramide synthase 5 (LASS5), mRNA.	LAG1 homolog, ceramide synthase 5	0.006949266	-1.156374089
OLAH	Oleoyl-ACP hydrolase gene:ENSG00000152463 /// Thioesterase domain containing 1, isoform CRA_a gene:ENSG00000152463 /// S-acyl fatty acid synthase thioesterase, medium chain gene:ENSG00000152463 /// Homo sapiens oleoyl-ACP hydrolase, mRNA (cDNA clone MGC:51852 IMAGE:5742377), complete cds. /// Homo sapiens oleoyl-ACP hydrolase (OLAH), transcript variant 1, mRNA. /// Homo sapiens oleoyl-ACP hydrolase (OLAH), transcript variant 2, mRNA.	oleoyl-ACP hydrolase	6.65941E-06	-1.729750177
CDS1	Phosphatidate cytidylyltransferase 1 gene:ENSG00000163624 /// Human CDP-diacylglycerol synthase mRNA, complete cds. /// Homo sapiens CDP-diacylglycerol synthase (phosphatidate cytidylyltransferase) 1 (CDS1), mRNA.	CDP-diacylglycerol synthase (phosphatidate cytidylyltransferase) 1	0.003871784	-1.179875975
SCD	Acyl-CoA desaturase gene:ENSG00000099194 /// Homo sapiens stearoyl-CoA desaturase (SCD) mRNA, complete cds. /// Homo sapiens stearoyl-CoA desaturase (delta-9-desaturase) (SCD), mRNA.	stearoyl-CoA desaturase (delta-9-desaturase)	0.001709222	1.205611478

PIGS	Isoform 2 of GPI transamidase component PIG-S gene:ENSG00000087111 /// Isoform 1 of GPI transamidase component PIG-S gene:ENSG00000087111 /// HSPC309 (Fragment) gene:ENSG00000087111 /// Homo sapiens clone DNA94713 PIGS (UNQ1873) mRNA, complete cds. /// Homo sapiens phosphatidylinositol glycan anchor biosynthesis, class S (PIGS), mRNA.	phosphatidylinositol glycan anchor biosynthesis, class S	0.005657295	1.179282937
FUT11	Isoform 2 of Alpha-(1,3)-fucosyltransferase 11 gene:ENSG00000196968 /// Isoform 1 of Alpha-(1,3)-fucosyltransferase 11 gene:ENSG00000196968 /// Homo sapiens fucosyltransferase 11 (alpha (1,3) fucosyltransferase), mRNA (cDNA clone MGC:33202 IMAGE:5271548), complete cds. /// Homo sapiens fucosyltransferase 11 (alpha (1,3) fucosyltransferase) (FUT11), mRNA.	fucosyltransferase 11 (alpha (1,3) fucosyltransferase)	0.001430649	1.246784793
PLP1	cDNA FLJ54326, highly similar to Myelin proteolipid protein gene:ENSG00000123560 /// 31 kDa protein gene:ENSG00000123560 /// Isoform 1 of Myelin proteolipid protein gene:ENSG00000123560 /// Human myelin proteolipid protein mRNA, complete cds. /// Homo sapiens proteolipid protein 1 (PLP1), transcript variant 3, mRNA. /// Homo sapiens proteolipid protein 1 (PLP1), transcript variant 2, mRNA. /// Homo sapiens proteolipid protein 1 (PLP1), transcript variant 1, mRNA.	proteolipid protein 1	0.000798801	1.217445851
LASS6	LASS6 protein gene:ENSG00000172292 /// LAG1 longevity assurance homolog 6 gene:ENSG00000172292 /// Homo sapiens LAG1 homolog, ceramide synthase 6, mRNA (cDNA clone MGC:129949 IMAGE:40031838), complete cds. /// Homo sapiens LAG1	LAG1 homolog, ceramide synthase 6	0.00473864	-1.155431587

	homolog, ceramide synthase 6 (LASS6), mRNA.			
INSIG1	insulin induced gene 1 isoform 2 gene:ENSG00000186480 /// Insulin-induced gene 1 protein gene:ENSG00000186480 /// insulin induced gene 1 isoform 3 gene:ENSG00000186480 /// Homo sapiens insulin induced gene 1, mRNA (cDNA clone MGC:1405 IMAGE:3546928), complete cds. /// Homo sapiens insulin induced gene 1 (INSIG1), transcript variant 1, mRNA. /// Homo sapiens insulin induced gene 1 (INSIG1), transcript variant 2, mRNA. /// Homo sapiens insulin induced gene 1 (INSIG1), transcript variant 3, mRNA.	insulin induced gene 1	0.000115835	1.293557142
TRAPPC3	Trafficking protein particle complex subunit 3 gene:ENSG00000054116 /// Putative uncharacterized protein TRAPPC3 gene:ENSG00000054116 /// Putative uncharacterized protein TRAPPC3 gene:ENSG00000054116 /// Putative uncharacterized protein TRAPPC3 gene:ENSG00000054116 /// Homo sapiens trafficking protein particle complex 3, mRNA (cDNA clone MGC:2035 IMAGE:3051295), complete cds. /// Homo sapiens trafficking protein particle complex 3 (TRAPPC3), mRNA.	trafficking protein particle complex 3	0.00805562	1.153669285

MCFD2	cdna:known chromosome:NCBI36:2:46982517:46996697:-1 gene:ENSG00000180398 /// cdna:known chromosome:NCBI36:2:46982517:46996459:-1 gene:ENSG00000180398 /// cdna:known chromosome:NCBI36:2:46982517:46996423:-1 gene:ENSG00000180398 /// Multiple coagulation factor deficiency protein 2 gene:ENSG00000180398 /// cdna:known chromosome:NCBI36:2:46985601:47022375:-1 gene:ENSG00000180398 /// cdna:known chromosome:NCBI36:2:46985914:46996453:-1 gene:ENSG00000180398 /// cdna:known chromosome:NCBI36:2:46986011:47022498:-1 gene:ENSG00000180398 /// cdna:known chromosome:NCBI36:2:46986037:46996755:-1 gene:ENSG00000180398 /// Homo sapiens multiple coagulation factor deficiency 2, mRNA (cDNA clone MGC:9537 IMAGE:3925166), complete cds. /// Homo sapiens multiple coagulation factor deficiency 2 (MCFD2), mRNA.	multiple coagulation factor deficiency 2	0.001766448	1.189254788
CYB5A	Isoform 1 of Cytochrome b5 gene:ENSG00000166347 /// Isoform 2 of Cytochrome b5 gene:ENSG00000166347 /// Putative uncharacterized protein CYB5A gene:ENSG00000166347 /// Homo sapiens mRNA for cytochrome b-5 isoform 1 variant protein. /// Homo sapiens cytochrome b5 type A (microsomal) (CYB5A), transcript variant 1, mRNA. /// Homo sapiens cytochrome b5 type A (microsomal) (CYB5A), transcript variant 2, mRNA.	cytochrome b5 type A (microsomal)	0.004823073	1.187411258

CYP26A1	cytochrome P450, family 26, subfamily A, polypeptide 1 isoform 2 gene:ENSG00000095596 /// Cytochrome P450 26A1 gene:ENSG00000095596 /// cytochrome P450, family 26, subfamily A, polypeptide 1 isoform 2 gene:ENSG00000095596 /// Homo sapiens cDNA PSEC0064 fis, clone NT2RP2000957, highly similar to CYTOCHROME P450 26 (EC 1.14.-.-). /// Homo sapiens cytochrome P450, family 26, subfamily A, polypeptide 1 (CYP26A1), transcript variant 1, mRNA. /// Homo sapiens cytochrome P450, family 26, subfamily A, polypeptide 1 (CYP26A1), transcript variant 2, mRNA.	cytochrome P450, family 26, subfamily A, polypeptide 1	7.1195E-09	1.832081174
CYP2R1	Vitamin D 25-hydroxylase gene:ENSG00000186104 /// Homo sapiens cytochrome P450 2R1 (CYP2R1) mRNA, complete cds. /// Homo sapiens cytochrome P450, family 2, subfamily R, polypeptide 1 (CYP2R1), mRNA.	cytochrome P450, family 2, subfamily R, polypeptide 1	0.000786597	1.255785406
TMEM109	Transmembrane protein 109 gene:ENSG00000110108 /// Homo sapiens transmembrane protein 109, mRNA (cDNA clone MGC:5508 IMAGE:3453616), complete cds. /// Homo sapiens transmembrane protein 109 (TMEM109), mRNA.	transmembrane protein 109	0.001754008	1.194580934

HERPUD1	homocysteine-inducible, endoplasmic reticulum stress-inducible, ubiquitin-like domain member 1 isoform 3 gene:ENSG00000051108 /// Isoform 1 of Homocysteine-responsive endoplasmic reticulum-resident ubiquitin-like domain member 1 protein (Fragment) gene:ENSG00000051108 /// Isoform 3 of Homocysteine-responsive endoplasmic reticulum-resident ubiquitin-like domain member 1 protein (Fragment) gene:ENSG00000051108 /// Homo sapiens homocysteine-inducible, endoplasmic reticulum stress-inducible, ubiquitin-like domain member 1, mRNA (cDNA clone MGC:15680 IMAGE:3350209), complete cds. /// Homo sapiens homocysteine-inducible, endoplasmic reticulum stress-inducible, ubiquitin-like domain member 1 (HERPUD1), transcript variant 1, mRNA. /// Homo sapiens homocysteine-inducible, endoplasmic reticulum stress-inducible, ubiquitin-like domain member 1 (HERPUD1), transcript variant 2, mRNA. /// Homo sapiens homocysteine-inducible, endoplasmic reticulum stress-inducible, ubiquitin-like domain member 1 (HERPUD1), transcript variant 3, mRNA.	homocysteine-inducible, endoplasmic reticulum stress-inducible, ubiquitin-like domain member 1	0.002273983	-1.179925913
IER3IP1	Homo sapiens PRO2309 mRNA, complete cds.	immediate early response 3 interacting protein 1	0.416335366	-1.125829568
SVIP	Small VCP/p97-interacting protein gene:ENSG00000198168 /// Homo sapiens mRNA; cDNA DKFZp313A2432 (from clone DKFZp313A2432). /// Homo sapiens small VCP/p97-interacting protein (SVIP), mRNA.	small VCP/p97-interacting protein	0.004605461	1.268457487

ERGIC1	cdna:known chromosome:NCBI36:5:172193928:172258194:1 gene:ENSG00000113719 /// 35 kDa protein gene:ENSG00000113719 /// Isoform 1 of Endoplasmic reticulum-Golgi intermediate compartment protein 1 gene:ENSG00000113719 /// cdna:known chromosome:NCBI36:5:172194020:172280180:1 gene:ENSG00000113719 /// Isoform 3 of Endoplasmic reticulum-Golgi intermediate compartment protein 1 gene:ENSG00000113719 /// Homo sapiens endoplasmic reticulum-golgi intermediate compartment (ERGIC) 1, mRNA (cDNA clone MGC:16233 IMAGE:3677787), complete cds. /// Homo sapiens endoplasmic reticulum-golgi intermediate compartment (ERGIC) 1 (ERGIC1), mRNA.	endoplasmic reticulum-golgi intermediate compartment (ERGIC) 1	0.000491703	1.243551427
TOR1AIP2	Torsin-1A-interacting protein 2 gene:ENSG00000169905 /// IFRG15 protein gene:ENSG00000218839 /// Homo sapiens cDNA FLJ77012 complete cds, highly similar to Homo sapiens interferon responsive gene 15 (IFRG15), mRNA. /// Homo sapiens torsin A interacting protein 2, mRNA (cDNA clone MGC:126581 IMAGE:8069038), complete cds. /// Homo sapiens torsin A interacting protein 2 (TOR1AIP2), mRNA.	interferon responsive gene 15 /// torsin A interacting protein 2	0.001893375	-1.192769275
**Functional Group 10**	**Transcript Description**	**Gene Title**	**Bayes.lnp**	**Fold**

EHHADH	Peroxisomal bifunctional enzyme gene:ENSG00000113790 /// Homo sapiens enoyl-Coenzyme A, hydratase/3-hydroxyacyl Coenzyme A dehydrogenase, mRNA (cDNA clone MGC:47770 IMAGE:5533337), complete cds. /// Homo sapiens enoyl-Coenzyme A, hydratase/3-hydroxyacyl Coenzyme A dehydrogenase (EHHADH), mRNA.	enoyl-Coenzyme A, hydratase/3-hydroxyacyl Coenzyme A dehydrogenase	0.004094174	1.255710944
CROT	Peroxisomal carnitine O-octanoyltransferase gene:ENSG00000005469 /// Homo sapiens carnitine O-octanoyltransferase, mRNA (cDNA clone MGC:47608 IMAGE:5242962), complete cds. /// Homo sapiens carnitine O-octanoyltransferase (CROT), mRNA.	carnitine O-octanoyltransferase	0.000849813	-1.246471675
PECR	Peroxisomal trans-2-enoyl-CoA reductase gene:ENSG00000115425 /// Homo sapiens peroxisomal trans 2-enoyl CoA reductase mRNA, complete cds. /// Homo sapiens peroxisomal trans-2-enoyl-CoA reductase (PECR), mRNA.	peroxisomal trans-2-enoyl-CoA reductase	0.000520923	1.345997976
PECI	Peroxisomal 3,2-trans-enoyl-CoA isomerase gene:ENSG00000198721 /// Peroxisomal D3,D2-enoyl-CoA isomerase gene:ENSG00000198721 /// Peroxisomal 3,2-trans-enoyl-CoA isomerase gene:ENSG00000198721 /// Peroxisomal D3,D2-enoyl-CoA isomerase gene:ENSG00000198721 /// Peroxisomal 3,2-trans-enoyl-CoA isomerase gene:ENSG00000198721 /// Homo sapiens hepatocellular carcinoma-associated antigen 64 (HCA64) mRNA, complete cds. /// Homo sapiens peroxisomal D3,D2-enoyl-CoA isomerase (PECI), transcript variant 1, mRNA. /// Homo sapiens peroxisomal D3,D2-enoyl-CoA isomerase (PECI), transcript variant 2, mRNA.	peroxisomal D3,D2-enoyl-CoA isomerase	0.000130711	1.254021976

DCI	Isoform 1 of 3,2-trans-enoyl-CoA isomerase, mitochondrial gene:ENSG00000167969 /// Homo sapiens cDNA FLJ76046 complete cds, highly similar to Homo sapiens dodecenoyl-Coenzyme A delta isomerase (3,2trans-enoyl-Coenzyme A isomerase) (DCI), mRNA. /// Homo sapiens dodecenoyl-Coenzyme A delta isomerase (3,2 trans-enoyl-Coenzyme A isomerase) (DCI), nuclear gene encoding mitochondrial protein, mRNA.	dodecenoyl-Coenzyme A delta isomerase (3,2 trans-enoyl-Coenzyme A isomerase)	0.003412221	1.180928766
**Functional Group 9**	**Transcript Description**	**Gene Title**	**Bayes.ln p**	**Fold**
ODF2	Outer dense fiber of sperm tails 2 gene:ENSG00000136811 /// Isoform 3 of Outer dense fiber protein 2 gene:ENSG00000136811 /// Isoform 4 of Outer dense fiber protein 2 gene:ENSG00000136811 /// Isoform 5 of Outer dense fiber protein 2 gene:ENSG00000136811 /// Isoform 3 of Outer dense fiber protein 2 gene:ENSG00000136811 /// Isoform 5 of Outer dense fiber protein 2 gene:ENSG00000136811 /// Isoform 1 of Outer dense fiber protein 2 gene:ENSG00000136811 /// Outer dense fiber of sperm tails 2 gene:ENSG00000136811 /// Homo sapiens outer dense fiber of sperm tails 2, mRNA (cDNA clone MGC:111096 IMAGE:30378678), complete cds. /// Homo sapiens outer dense fiber of sperm tails 2 (ODF2), transcript variant 2, mRNA. /// Homo sapiens outer dense fiber of sperm tails 2 (ODF2), transcript variant 1, mRNA.	outer dense fiber of sperm tails 2	0.000974165	1.240670374

CP110	Isoform 2 of Centrosomal protein of 110 kDa gene:ENSG00000103540 /// Isoform 1 of Centrosomal protein of 110 kDa gene:ENSG00000103540 /// Isoform 1 of Centrosomal protein of 110 kDa gene:ENSG00000103540 /// Isoform 2 of Centrosomal protein of 110 kDa gene:ENSG00000103540 /// Homo sapiens CP110 protein, mRNA (cDNA clone MGC:33038 IMAGE:5267904), complete cds. /// Homo sapiens CP110 protein (CP110), mRNA.	CP110 protein	6.48832E-05	-1.31833147
CEP76	Centrosomal protein of 76 kDa gene:ENSG00000101624 /// Homo sapiens centrosomal protein 76kDa, mRNA (cDNA clone MGC:26034 IMAGE:4792211), complete cds. /// Homo sapiens centrosomal protein 76kDa (CEP76), mRNA.	centrosomal protein 76kDa	0.003484797	-1.219588703
SSNA1	Sjoegren syndrome nuclear autoantigen 1 gene:ENSG00000176101 /// Homo sapiens Sjogren syndrome nuclear autoantigen 1, mRNA (cDNA clone MGC:5078 IMAGE:3459939), complete cds. /// Homo sapiens Sjogren syndrome nuclear autoantigen 1 (SSNA1), mRNA.	Sjogren syndrome nuclear autoantigen 1	0.000344734	1.25758904

CEP110	Centrosomal protein 110kDa gene:ENSG00000119397 /// Isoform 1 of Centriolin gene:ENSG00000119397 /// Centrosomal protein 110kDa gene:ENSG00000119397 /// Centrosomal protein 110kDa gene:ENSG00000119397 /// Isoform 1 of Centriolin gene:ENSG00000119397 /// Centrosomal protein 110kDa gene:ENSG00000119397 /// Putative uncharacterized protein CEP110 gene:ENSG00000119397 /// Isoform 2 of Centriolin gene:ENSG00000119397 /// Centrosomal protein 110kDa gene:ENSG00000119397 /// Centrosomal protein 110kDa gene:ENSG00000119397 /// Putative uncharacterized protein CEP110 gene:ENSG00000119397 /// Homo sapiens centrosomal protein 110kDa, mRNA (cDNA clone MGC:168904 IMAGE:9021281), complete cds. /// Homo sapiens centrosomal protein 110kDa (CEP110), mRNA.	centrosomal protein 110kDa	0.007654753	1.260544664

BLOC1S2	Isoform 2 of Biogenesis of lysosome-related organelles complex 1 subunit 2 gene:ENSG00000196072 /// Isoform 1 of Biogenesis of lysosome-related organelles complex 1 subunit 2 gene:ENSG00000196072 /// Isoform 2 of Biogenesis of lysosome-related organelles complex 1 subunit 2 gene:ENSG00000196072 /// Isoform 2 of Biogenesis of lysosome-related organelles complex 1 subunit 2 gene:ENSG00000196072 /// Homo sapiens cDNA FLJ54853 complete cds, highly similar to Biogenesis of lysosome-related organellescomplex-1 subunit 2. /// Homo sapiens biogenesis of lysosomal organelles complex-1, subunit 2 (BLOC1S2), transcript variant 2, mRNA. /// Homo sapiens biogenesis of lysosomal organelles complex-1, subunit 2 (BLOC1S2), transcript variant 1, mRNA.	biogenesis of lysosomal organelles complex-1, subunit 2	0.000693186	-1.182610553
**Functional Group 11**	**Transcript Description**	**Gene Title**	**Bayes.lnp**	**Fold**
SYNGR2	Synaptogyrin-2 gene:ENSG00000108639 /// Homo sapiens synaptogyrin 2, mRNA (cDNA clone MGC:8571 IMAGE:2823026), complete cds. /// Homo sapiens synaptogyrin 2 (SYNGR2), mRNA.	synaptogyrin 2	0.008631148	1.17397342
IGF2R	274 kDa protein gene:ENSG00000197081 /// Cation-independent mannose-6-phosphate receptor gene:ENSG00000197081 /// Human cation-independent mannose 6-phosphate receptor mRNA, complete cds. /// Homo sapiens insulin-like growth factor 2 receptor (IGF2R), mRNA.	insulin-like growth factor 2 receptor	0.006758776	-1.165231099

CD99	Isoform I of CD99 antigen gene:ENSG00000002586 /// Putative uncharacterized protein CD99 gene:ENSG00000002586 /// CD99 antigen isoform b precursor gene:ENSG00000002586 /// Putative uncharacterized protein CD99 gene:ENSG00000002586 /// Putative uncharacterized protein CD99 gene:ENSG00000002586 /// Homo sapiens CD99 molecule, mRNA (cDNA clone MGC:19734 IMAGE:3606974), complete cds. /// Homo sapiens CD99 molecule (CD99), transcript variant 1, mRNA. /// Homo sapiens CD99 molecule (CD99), transcript variant 2, mRNA.	CD99 molecule	0.004074288	1.190899882
GPRC5A	Retinoic acid-induced protein 3 gene:ENSG00000013588 /// Homo sapiens cDNA FLJ16117 fis, clone ASTRO2003632, highly similar to Retinoic acid-induced protein 3 (G-proteincoupled receptor family C group 5 member A). /// Homo sapiens G protein-coupled receptor, family C, group 5, member A (GPRC5A), mRNA.	G protein-coupled receptor, family C, group 5, member A	4.48256E-06	-1.572536281
LAPTM5	Lysosomal-associated transmembrane protein 5 gene:ENSG00000162511 /// Homo sapiens cDNA FLJ61683 complete cds, moderately similar to Lysosomal-associated multitransmembrane protein. /// Human lysosomal-associated multitransmembrane protein (LAPTm5) mRNA, complete cds. /// Homo sapiens lysosomal multispanning membrane protein 5 (LAPTM5), mRNA.	actin filament associated protein 1-like 1 /// lysosomal multispanning membrane protein 5	0.000670384	-1.424435353

LETM1	LETM1 and EF-hand domain-containing protein 1, mitochondrial gene:ENSG00000168924 /// Homo sapiens leucine zipper-EF-hand containing transmembrane protein 1 (LETM1) mRNA, complete cds. /// Homo sapiens leucine zipper-EF-hand containing transmembrane protein 1 (LETM1), nuclear gene encoding mitochondrial protein, mRNA.	leucine zipper-EF-hand containing transmembrane protein 1	0.001545727	1.200565539
M6PR	Cation-dependent mannose-6-phosphate receptor gene:ENSG00000003056 /// Homo sapiens mannose-6-phosphate receptor (cation dependent), mRNA (cDNA clone MGC:4080 IMAGE:3530887), complete cds. /// Homo sapiens mannose-6-phosphate receptor (cation dependent) (M6PR), mRNA.	mannose-6-phosphate receptor (cation dependent)	0.006495577	-1.174415302
LANCL1	LanC-like protein 1 gene:ENSG00000115365 /// Homo sapiens LanC lantibiotic synthetase component C-like 1 (bacterial), mRNA (cDNA clone MGC:26280 IMAGE:4829574), complete cds. /// Homo sapiens LanC lantibiotic synthetase component C-like 1 (bacterial) (LANCL1), transcript variant 3, mRNA. /// Homo sapiens LanC lantibiotic synthetase component C-like 1 (bacterial) (LANCL1), transcript variant 1, mRNA. /// Homo sapiens LanC lantibiotic synthetase component C-like 1 (bacterial) (LANCL1), transcript variant 2, mRNA.	LanC lantibiotic synthetase component C-like 1 (bacterial)	0.005249954	1.171764212

C17orf61	UPF0451 protein C17orf61 gene:ENSG00000205544 /// Homo sapiens cDNA FLJ31260 fis, clone KIDNE2005854, highly similar to Phospholipid scramblase 3. /// Homo sapiens cDNA FLJ77856 complete cds, highly similar to Homo sapiens phospholipid scramblase 3 (PLSCR3), mRNA. /// Homo sapiens chromosome 17 open reading frame 61, mRNA (cDNA clone MGC:40107 IMAGE:5418259), complete cds.	phospholipid scramblase 3 /// chromosome 17 open reading frame 61	0.001099 691	1.276122 626
CCDC56	Coiled-coil domain-containing protein 56 gene:ENSG00000183978 /// Homo sapiens mRNA for HSPC009 variant, clone: PNC06538. /// Homo sapiens coiled-coil domain containing 56 (CCDC56), mRNA.	coiled-coil domain containing 56	0.003281 488	1.232223 987
SMCR7L	Putative uncharacterized protein DKFZp434F0116 gene:ENSG00000100335 /// Smith-Magenis syndrome chromosome region candidate gene 7 protein-like gene:ENSG00000100335 /// Putative uncharacterized protein DKFZp434F0116 gene:ENSG00000100335 /// Smith-Magenis syndrome chromosome region candidate gene 7 protein-like gene:ENSG00000100335 /// Homo sapiens Smith-Magenis syndrome chromosome region, candidate 7-like, mRNA (cDNA clone MGC:15774 IMAGE:3502711), complete cds. /// Homo sapiens Smith-Magenis syndrome chromosome region, candidate 7-like (SMCR7L), mRNA.	Smith-Magenis syndrome chromosome region, candidate 7-like	0.003381 395	1.214924 348

PSMG1	Isoform 1 of Proteasome assembly chaperone 1 gene:ENSG00000183527 /// Isoform 2 of Proteasome assembly chaperone 1 gene:ENSG00000183527 /// Homo sapiens proteasome (prosome, macropain) assembly chaperone 1, mRNA (cDNA clone MGC:15154 IMAGE:3343732), complete cds. /// Homo sapiens proteasome (prosome, macropain) assembly chaperone 1 (PSMG1), transcript variant 1, mRNA. /// Homo sapiens proteasome (prosome, macropain) assembly chaperone 1 (PSMG1), transcript variant 2, mRNA.	proteasome (prosome, macropain) assembly chaperone 1	0.005576	1.172940102
**Functional Group 12**	**Transcript Description**	**Gene Title**	**Bayes.lnp**	**Fold**
MRPS25	28S ribosomal protein S25, mitochondrial gene:ENSG00000131368 /// Homo sapiens mitochondrial ribosomal protein S25, mRNA (cDNA clone MGC:4330 IMAGE:2820823), complete cds. /// Homo sapiens mitochondrial ribosomal protein S25 (MRPS25), nuclear gene encoding mitochondrial protein, mRNA.	mitochondrial ribosomal protein S25	0.007258278	1.175421702
RPL39L	60S ribosomal protein L39-like gene:ENSG00000163923 /// Homo sapiens ribosomal protein L39-like, mRNA (cDNA clone MGC:20168 IMAGE:4555759), complete cds. /// Homo sapiens ribosomal protein L39-like (RPL39L), mRNA.	ribosomal protein L39-like	0.004152236	1.255115192

RPL31	cdna:known chromosome:NCBI36:2:100985123:100987498:1 gene:ENSG00000071082 /// 60S ribosomal protein L31 gene:ENSG00000071082 /// cdna:known chromosome:NCBI36:2:100985177:101002510:1 gene:ENSG00000071082 /// cdna:known chromosome:NCBI36:2:100985187:100989312:1 gene:ENSG00000071082 /// cdna:known chromosome:NCBI36:2:100985187:100989313:1 gene:ENSG00000071082 /// cdna:known chromosome:NCBI36:2:100985187:101001200:1 gene:ENSG00000071082 /// cdna:known chromosome:NCBI36:2:100985187:101001183:1 gene:ENSG00000071082 /// cdna:known chromosome:NCBI36:2:100985585:100989261:1 gene:ENSG00000071082 /// Homo sapiens ribosomal protein L31, mRNA (cDNA clone MGC:88191 IMAGE:4714258), complete cds. /// Homo sapiens ribosomal protein L31 (RPL31), transcript variant 2, mRNA. /// Homo sapiens ribosomal protein L31 (RPL31), transcript variant 4, mRNA. /// Homo sapiens ribosomal protein L31 (RPL31), transcript variant 1, mRNA.	ribosomal protein L31	0.001704748	1.280233424
RPL27	60S ribosomal protein L27 gene:ENSG00000131469 /// 60S ribosomal protein L27 gene:ENSG00000131469 /// Homo sapiens ribosomal protein L27 (RPL27) mRNA, complete cds. /// Homo sapiens ribosomal protein L27 (RPL27), mRNA.	ribosomal protein L27	0.009406362	1.217826077

RPL13	60S ribosomal protein L13 gene:ENSG00000167526 /// 60S ribosomal protein L13 gene:ENSG00000167526 /// Homo sapiens cDNA FLJ45674 fis, clone D9OST2004417, highly similar to 60S ribosomal protein L13. /// Homo sapiens ribosomal protein L13 (RPL13), transcript variant 2, mRNA. /// Homo sapiens ribosomal protein L13 (RPL13), transcript variant 1, mRNA.	ribosomal protein L13	0.041636024	1.129877612
MRPL23	Putative uncharacterized protein MRPL23 gene:ENSG00000214026 /// Putative uncharacterized protein MRPL23 (Fragment) gene:ENSG00000214026 /// 39S ribosomal protein L23, mitochondrial gene:ENSG00000214026 /// Putative uncharacterized protein MRPL23 gene:ENSG00000214026 /// Putative uncharacterized protein MRPL23 gene:ENSG00000214026 /// Putative uncharacterized protein MRPL23 gene:ENSG00000214026 /// 39S ribosomal protein L23, mitochondrial gene:ENSG00000214026 /// Homo sapiens mitochondrial ribosomal protein L23, mRNA (cDNA clone MGC:12795 IMAGE:4298000), complete cds. /// Homo sapiens mitochondrial ribosomal protein L23 (MRPL23), nuclear gene encoding mitochondrial protein, mRNA.	mitochondrial ribosomal protein L23	0.001049646	1.230894556

MRPL24	39S ribosomal protein L24, mitochondrial gene:ENSG00000143314 /// 39S ribosomal protein L24, mitochondrial gene:ENSG00000143314 /// Homo sapiens mitochondrial ribosomal protein L24, mRNA (cDNA clone MGC:22737 IMAGE:4108596), complete cds. /// Homo sapiens mitochondrial ribosomal protein L24 (MRPL24), nuclear gene encoding mitochondrial protein, transcript variant 1, mRNA. /// Homo sapiens mitochondrial ribosomal protein L24 (MRPL24), nuclear gene encoding mitochondrial protein, transcript variant 2, mRNA.	mitochondrial ribosomal protein L24	3.21024E-05	1.308731098
JTV1	Multisynthetase complex auxiliary component p38 gene:ENSG00000106305 /// Putative uncharacterized protein ENSP00000383327 gene:ENSG00000106305 /// 28 kDa protein gene:ENSG00000106305 /// Homo sapiens JTV1 gene, mRNA (cDNA clone MGC:17435 IMAGE:3446074), complete cds. /// Homo sapiens JTV1 gene (JTV1), mRNA.	JTV1 gene	0.000115209	1.285672543

MTRF1L	Isoform 4 of Peptide chain release factor 1-like, mitochondrial gene:ENSG00000112031 /// Isoform 1 of Peptide chain release factor 1-like, mitochondrial gene:ENSG00000112031 /// Isoform 2 of Peptide chain release factor 1-like, mitochondrial gene:ENSG00000112031 /// cdna:known chromosome:NCBI36:4:189896500:189897680:1 gene:ENSG00000180015 /// Homo sapiens cDNA FLJ76265 complete cds, highly similar to Homo sapiens mitochondrial translational release factor 1-like (MTRF1L), mRNA. /// Homo sapiens mitochondrial translational release factor 1-like (MTRF1L), nuclear gene encoding mitochondrial protein, transcript variant 1, mRNA. /// Homo sapiens mitochondrial translational release factor 1-like (MTRF1L), nuclear gene encoding mitochondrial protein, transcript variant 2, mRNA.	mitochondrial translational release factor 1-like	0.006968843	-1.159501335
MRPS26	28S ribosomal protein S26, mitochondrial gene:ENSG00000125901 /// Homo sapiens mitochondrial ribosomal protein S26, mRNA (cDNA clone MGC:4583 IMAGE:3051126), complete cds. /// Homo sapiens mitochondrial ribosomal protein S26 (MRPS26), nuclear gene encoding mitochondrial protein, mRNA.	mitochondrial ribosomal protein S26	0.003409606	1.169242609

QRSL1	Isoform 1 of Glutamyl-tRNA(Gln) amidotransferase subunit A homolog gene:ENSG00000130348 /// Isoform 2 of Glutamyl-tRNA(Gln) amidotransferase subunit A homolog gene:ENSG00000130348 /// Homo sapiens glutaminyl-tRNA synthase (glutamine-hydrolyzing)-like 1, mRNA (cDNA clone MGC:12589 IMAGE:3605968), complete cds. /// Homo sapiens glutaminyl-tRNA synthase (glutamine-hydrolyzing)-like 1 (QRSL1), mRNA.	glutaminyl-tRNA synthase (glutamine-hydrolyzing)-like 1	0.002795037	1.188231082
RPS27L	40S ribosomal protein S27-like protein gene:ENSG00000185088 /// 13 kDa protein gene:ENSG00000185088 /// Homo sapiens ribosomal protein S27-like, mRNA (cDNA clone MGC:12175 IMAGE:3827362), complete cds. /// Homo sapiens ribosomal protein S27-like (RPS27L), mRNA.	ribosomal protein S27-like	0.001063446	-1.254328897
MRPL16	39S ribosomal protein L16, mitochondrial gene:ENSG00000166902 /// Homo sapiens 28.4 kDa protein mRNA, complete cds. /// Homo sapiens mitochondrial ribosomal protein L16 (MRPL16), nuclear gene encoding mitochondrial protein, mRNA.	mitochondrial ribosomal protein L16	0.005442128	1.169424372
MRPS24	28S ribosomal protein S24, mitochondrial gene:ENSG00000062582 /// Putative uncharacterized protein MRPS24 gene:ENSG00000062582 /// Homo sapiens mitochondrial ribosomal protein S24, mRNA (cDNA clone MGC:20409 IMAGE:4637888), complete cds. /// Homo sapiens mitochondrial ribosomal protein S24 (MRPS24), nuclear gene encoding mitochondrial protein, mRNA.	mitochondrial ribosomal protein S24	0.004980842	1.177301531
LOC642892	PREDICTED: Homo sapiens similar to mCG7602 (LOC642892), mRNA.	similar to mCG7602	8.7572E-05	1.332199568

**Functional Group 13**	**Transcript Description**	**Gene Title**	**Bayes.lnp**	**Fold**
SRI	Sorcin gene:ENSG00000075142 /// sorcin isoform b gene:ENSG00000075142 /// Human sorcin (SRI) mRNA, complete cds. /// Homo sapiens sorcin (SRI), transcript variant 1, mRNA. /// Homo sapiens sorcin (SRI), transcript variant 2, mRNA.	sorcin	9.72486E-05	1.335394273
GCA	Grancalcin gene:ENSG00000115271 /// Homo sapiens grancalcin, EF-hand calcium binding protein, mRNA (cDNA clone MGC:12231 IMAGE:3996889), complete cds. /// Homo sapiens grancalcin, EF-hand calcium binding protein (GCA), mRNA.	grancalcin, EF-hand calcium binding protein	2.17768E-05	1.376423224
MYL4	Myosin light chain 4 gene:ENSG00000198336 /// Myosin light chain 4 gene:ENSG00000198336 /// Homo sapiens myosin, light chain 4, alkali; atrial, embryonic, mRNA (cDNA clone MGC:34523 IMAGE:5226518), complete cds. /// Homo sapiens myosin, light chain 4, alkali; atrial, embryonic (MYL4), transcript variant 1, mRNA. /// Homo sapiens myosin, light chain 4, alkali; atrial, embryonic (MYL4), transcript variant 2, mRNA.	myosin, light chain 4, alkali; atrial, embryonic	0.001866021	1.54330964
CALB1	Calbindin gene:ENSG00000104327 /// Homo sapiens calbindin 1, 28kDa, mRNA (cDNA clone MGC:24064 IMAGE:4592497), complete cds. /// Homo sapiens calbindin 1, 28kDa (CALB1), mRNA.	calbindin 1, 28kDa	2.05582E-08	-1.527780087

MCFD2	cdna:known chromosome:NCBI36:2:46982517:46996697:-1 gene:ENSG00000180398 /// cdna:known chromosome:NCBI36:2:46982517:46996459:-1 gene:ENSG00000180398 /// cdna:known chromosome:NCBI36:2:46982517:46996423:-1 gene:ENSG00000180398 /// Multiple coagulation factor deficiency protein 2 gene:ENSG00000180398 /// cdna:known chromosome:NCBI36:2:46985601:47022375:-1 gene:ENSG00000180398 /// cdna:known chromosome:NCBI36:2:46985914:46996453:-1 gene:ENSG00000180398 /// cdna:known chromosome:NCBI36:2:46986011:47022498:-1 gene:ENSG00000180398 /// cdna:known chromosome:NCBI36:2:46986037:46996755:-1 gene:ENSG00000180398 /// Homo sapiens multiple coagulation factor deficiency 2, mRNA (cDNA clone MGC:9537 IMAGE:3925166), complete cds. /// Homo sapiens multiple coagulation factor deficiency 2 (MCFD2), mRNA.	multiple coagulation factor deficiency 2	0.001766448	1.189254788
FKBP14	FK506-binding protein 14 gene:ENSG00000106080 /// Homo sapiens clone DNA44194 FKBP14 (UNQ322) mRNA, complete cds. /// Homo sapiens FK506 binding protein 14, 22 kDa (FKBP14), mRNA.	FK506 binding protein 14, 22 kDa	0.000327717	-1.275949087
**Functional Group 14**	**Transcript Description**	**Gene Title**	**Bayes.lnp**	**Fold**
GAS2L3	GAS2-like protein 3 gene:ENSG00000139354 /// Homo sapiens growth arrest-specific 2 like 3, mRNA (cDNA clone MGC:50047 IMAGE:5104644), complete cds. /// Homo sapiens growth arrest-specific 2 like 3 (GAS2L3), mRNA.	growth arrest-specific 2 like 3	0.000812828	1.268223614

PPP1R15A	Protein phosphatase 1 regulatory subunit 15A gene:ENSG00000087074 /// Homo sapiens cDNA FLJ51898 complete cds, highly similar to Homo sapiens protein phosphatase 1, regulatory (inhibitor) subunit 15A (PPP1R15A), mRNA. /// Homo sapiens protein phosphatase 1, regulatory (inhibitor) subunit 15A (PPP1R15A), mRNA.	protein phosphatase 1, regulatory (inhibitor) subunit 15A	4.03971E -07	- 1.544822 939
BTG3	Isoform 2 of Protein BTG3 gene:ENSG00000154640 /// Isoform 1 of Protein BTG3 gene:ENSG00000154640 /// Homo sapiens BTG family, member 3, mRNA (cDNA clone MGC:8928 IMAGE:3859781), complete cds. /// Homo sapiens BTG family, member 3 (BTG3), transcript variant 1, mRNA. /// Homo sapiens BTG family, member 3 (BTG3), transcript variant 2, mRNA.	BTG family, member 3	0.003335 818	- 1.190185 569
CGREF1	Putative uncharacterized protein CGREF1 gene:ENSG00000138028 /// Putative uncharacterized protein CGREF1 gene:ENSG00000138028 /// Putative uncharacterized protein CGREF1 gene:ENSG00000138028 /// Putative uncharacterized protein CGREF1 gene:ENSG00000138028 /// Putative uncharacterized protein CGREF1 gene:ENSG00000138028 /// Cell growth regulator with EF hand domain protein 1 gene:ENSG00000138028 /// Cell growth regulator with EF hand domain protein 1 gene:ENSG00000138028 /// Homo sapiens cell growth regulator with EF-hand domain 1, mRNA (cDNA clone MGC:34428 IMAGE:5182241), complete cds. /// Homo sapiens cell growth regulator with EF-hand domain 1 (CGREF1), mRNA.	cell growth regulator with EF-hand domain 1	0.000676 921	- 1.366963 823

RPRM	Protein reprimo gene:ENSG00000177519 /// Homo sapiens reprimo, TP53 dependent G2 arrest mediator candidate, mRNA (cDNA clone MGC:11260 IMAGE:3942270), complete cds. /// Homo sapiens reprimo, TP53 dependent G2 arrest mediator candidate (RPRM), mRNA.	reprimo, TP53 dependent G2 arrest mediator candidate	0.005161311	1.194146899
DLEU1	Leukemia-associated protein 1 gene:ENSG00000176124 /// Homo sapiens mRNA for B-cell neoplasia associated transcript, (BCMS gene), splice variant B, non coding transcript. /// Homo sapiens deleted in lymphocytic leukemia 1 (non-protein coding) (DLEU1), non-coding RNA.	deleted in lymphocytic leukemia 1 (non-protein coding)	9.35744E-05	1.34718688
MCC	93 kDa protein gene:ENSG00000171444 /// cdna:known chromosome:NCBI36:5:112390898:112852010:-1 gene:ENSG00000171444 /// Homo sapiens mRNA; cDNA DKFZp686B1231 (from clone DKFZp686B1231). /// Homo sapiens mutated in colorectal cancers (MCC), transcript variant 2, mRNA. /// Homo sapiens mutated in colorectal cancers (MCC), transcript variant 1, mRNA.	mutated in colorectal cancers	0.006328817	-1.237749327
**Functional Group 15**	**Transcript Description**	**Gene Title**	**Bayes.lnp**	**Fold**
GORASP1	Isoform 1 of Golgi reassembly-stacking protein 1 gene:ENSG00000114745 /// Homo sapiens cDNA FLJ42765 fis, clone BRAWH3002853, highly similar to Golgi reassembly-stacking protein 1. /// Homo sapiens golgi reassembly stacking protein 1, 65kDa (GORASP1), mRNA.	golgi reassembly stacking protein 1, 65kDa	0.009608401	1.188583408

STARD3NL	Isoform 1 of MLN64 N-terminal domain homolog gene:ENSG00000010270 /// Isoform 1 of MLN64 N-terminal domain homolog gene:ENSG00000010270 /// Homo sapiens cDNA FLJ34260 fis, clone FEBRA2000782, moderately similar to MLN 64 PROTEIN. /// Homo sapiens STARD3 N-terminal like (STARD3NL), mRNA.	STARD3 N-terminal like	0.000773 235	1.251229 806
TMED3	Transmembrane emp24 domain-containing protein 3 gene:ENSG00000166557 /// Homo sapiens cDNA FLJ75946 complete cds, highly similar to Homo sapiens transmembrane emp24 protein transport domain containing 3 (TMED3), mRNA. /// Homo sapiens transmembrane emp24 protein transport domain containing 3 (TMED3), mRNA.	transmembrane emp24 protein transport domain containing 3	0.009634 817	1.172139 107
CHMP2A	Charged multivesicular body protein 2a gene:ENSG00000130724 /// Charged multivesicular body protein 2a gene:ENSG00000130724 /// Homo sapiens chromatin modifying protein 2A, mRNA (cDNA clone MGC:806 IMAGE:3051024), complete cds. /// Homo sapiens chromatin modifying protein 2A (CHMP2A), transcript variant 1, mRNA. /// Homo sapiens chromatin modifying protein 2A (CHMP2A), transcript variant 2, mRNA.	chromatin modifying protein 2A	0.005334 726	1.168636 175
COG6	cDNA FLJ56431, highly similar to Conserved oligomeric Golgi complex component 6 gene:ENSG00000133103 /// Isoform 3 of Conserved oligomeric Golgi complex subunit 6 gene:ENSG00000133103 /// Homo sapiens component of oligomeric golgi complex 6, mRNA (cDNA clone MGC:48438 IMAGE:5263056), complete cds. /// Homo sapiens component of oligomeric golgi complex 6 (COG6), mRNA.	component of oligomeric golgi complex 6	0.004414 472	1.187367 828

TMEM9	Transmembrane protein 9 gene:ENSG00000116857 /// Transmembrane protein 9 gene:ENSG00000116857 /// Transmembrane protein 9 gene:ENSG00000116857 /// Transmembrane protein 9 gene:ENSG00000116857 /// Transmembrane protein 9 gene:ENSG00000116857 /// Transmembrane protein 9 gene:ENSG00000116857 /// 16 kDa protein gene:ENSG00000116857 /// Homo sapiens cDNA FLJ34407 fis, clone HEART1000173. /// Homo sapiens transmembrane protein 9 (TMEM9), mRNA.	transmembrane protein 9	0.001251575	1.213576556
VPS26A	Vacuolar protein sorting-associated protein 26A gene:ENSG00000122958 /// Vacuolar protein sorting-associated protein 26A gene:ENSG00000122958 /// vacuolar protein sorting 26 A isoform 2 gene:ENSG00000122958 /// Homo sapiens vacuolar sorting protein 26 (VPS26) mRNA, complete cds. /// Homo sapiens vacuolar protein sorting 26 homolog A (S. pombe) (VPS26A), transcript variant 1, mRNA. /// Homo sapiens vacuolar protein sorting 26 homolog A (S. pombe) (VPS26A), transcript variant 2, mRNA.	vacuolar protein sorting 26 homolog A (S. pombe)	0.00130143	1.200989301
VPS37B	Vacuolar protein sorting-associated protein 37B gene:ENSG00000139722 /// Homo sapiens vacuolar protein sorting 37 homolog B (S. cerevisiae), mRNA (cDNA clone MGC:4691 IMAGE:3533384), complete cds. /// Homo sapiens vacuolar protein sorting 37 homolog B (S. cerevisiae) (VPS37B), mRNA.	vacuolar protein sorting 37 homolog B (S. cerevisiae)	0.000343893	-1.298829859
**Functional Group 16**	**Transcript Description**	**Gene Title**	**Bayes.lnp**	**Fold**

NOG	Noggin gene:ENSG00000183691 /// Homo sapiens noggin, mRNA (cDNA clone MGC:23903 IMAGE:4737725), complete cds. /// Homo sapiens noggin (NOG), mRNA.	noggin	0.003703057	1.238350742
BMPER	BMP-binding endothelial regulator protein gene:ENSG00000164619 /// Homo sapiens crossveinless-2 mRNA, complete cds. /// Homo sapiens BMP binding endothelial regulator (BMPER), mRNA.	BMP binding endothelial regulator	1.35186E-09	-2.319014345
CER1	Cerberus gene:ENSG00000147869 /// Homo sapiens cerberus 1, cysteine knot superfamily, homolog (Xenopus laevis), mRNA (cDNA clone MGC:119894 IMAGE:40015380), complete cds. /// Homo sapiens cerberus 1, cysteine knot superfamily, homolog (Xenopus laevis) (CER1), mRNA.	cerberus 1, cysteine knot superfamily, homolog (Xenopus laevis)	5.35057E-08	1.47486324
GDF15	Growth/differentiation factor 15 gene:ENSG00000130513 /// Homo sapiens macrophage inhibitory cytokine-1 (MIC-1) mRNA, complete cds. /// Homo sapiens growth differentiation factor 15 (GDF15), mRNA.	growth differentiation factor 15	2.68327E-08	-1.724689975
SOSTDC1	Sclerostin domain-containing protein 1 gene:ENSG00000171243 /// Homo sapiens sclerostin domain containing 1, mRNA (cDNA clone MGC:14794 IMAGE:4048654), complete cds. /// Homo sapiens sclerostin domain containing 1 (SOSTDC1), mRNA.	sclerostin domain containing 1	1.57083E-06	-1.827702092
FRZB	Secreted frizzled-related protein 3 gene:ENSG00000162998 /// Human frezzled (fre) mRNA, complete cds. /// Homo sapiens frizzled-related protein (FRZB), mRNA.	frizzled-related protein	1.86211E-05	1.60632543
**Functional Group 17**	**Transcript Description**	**Gene Title**	**Bayes.lnp**	**Fold**

CDC27	Cell division cycle protein 27 homolog gene:ENSG00000004897 /// Homo sapiens cell division cycle 27 homolog (S. cerevisiae), mRNA (cDNA clone MGC:12709 IMAGE:4301175), complete cds. /// Homo sapiens cell division cycle 27 homolog (S. cerevisiae) (CDC27), transcript variant 1, mRNA. /// Homo sapiens cell division cycle 27 homolog (S. cerevisiae) (CDC27), transcript variant 2, mRNA.	cell division cycle 27 homolog (S. cerevisiae)	0.000880429	1.22261567
GPSM2	LGN protein gene:ENSG00000121957 /// LGN protein gene:ENSG00000121957 /// G-protein-signaling modulator 2 gene:ENSG00000121957 /// Homo sapiens cDNA FLJ56086 complete cds, highly similar to G-protein signaling modulator 2. /// Homo sapiens G-protein signaling modulator 2 (AGS3-like, C. elegans) (GPSM2), mRNA.	G-protein signaling modulator 2 (AGS3-like, C. elegans)	0.002637988	1.226955711
TOMM34	Mitochondrial import receptor subunit TOM34 gene:ENSG00000025772 /// cDNA FLJ26027 fis, clone PNC04328, highly similar to Homo sapiens translocase of outer mitochondrial membrane 34 gene:ENSG00000025772 /// Homo sapiens translocase of outer mitochondrial membrane 34, mRNA (cDNA clone MGC:3515 IMAGE:2960090), complete cds. /// Homo sapiens translocase of outer mitochondrial membrane 34 (TOMM34), nuclear gene encoding mitochondrial protein, mRNA.	translocase of outer mitochondrial membrane 34	0.006754355	1.167816537
TTC13	Isoform 1 of Tetratricopeptide repeat protein 13 gene:ENSG00000143643 /// tetratricopeptide repeat domain 13 isoform b gene:ENSG00000143643 /// Homo sapiens tetratricopeptide repeat domain 13 (TTC13), transcript variant 1, mRNA. /// Homo sapiens tetratricopeptide repeat domain 13 (TTC13), transcript variant 2, mRNA.	tetratricopeptide repeat domain 13	0.005835153	1.331225477

Functional Group 18	Transcript Description	Gene Title	Bayes.lnp	Fold
HSD17B7	17-beta-hydroxysteroid dehydrogenase type VII isoform gene:ENSG00000099251 /// Putative uncharacterized protein ENSP00000277668 gene:ENSG00000099251 /// cDNA FLJ60462, highly similar to 3-keto-steroid reductase gene:ENSG00000099251 /// 17beta-hydroxysteroid dehydrogenase type 7 form 2 gene:ENSG00000099251 /// Isoform 1 of 3-keto-steroid reductase gene:ENSG00000132196 /// Hydroxysteroid (17-beta) dehydrogenase 7 gene:ENSG00000132196 /// Isoform 3 of 3-keto-steroid reductase gene:ENSG00000132196 /// Hydroxysteroid (17-beta) dehydrogenase 7 gene:ENSG00000132196 /// Hydroxysteroid (17-beta) dehydrogenase 7 gene:ENSG00000132196 /// Homo sapiens hydroxysteroid (17-beta) dehydrogenase 7, mRNA (cDNA clone MGC:75018 IMAGE:6062803), complete cds. /// Homo sapiens hydroxysteroid (17-beta) dehydrogenase 7 pseudogene 2, mRNA (cDNA clone MGC:88684 IMAGE:4634372), complete cds. /// Homo sapiens hydroxysteroid (17-beta) dehydrogenase 7 pseudogene 2 (HSD17B7P2), non-coding RNA. /// Homo sapiens hydroxysteroid (17-beta) dehydrogenase 7 (HSD17B7), mRNA.	hydroxysteroid (17-beta) dehydrogenase 7 /// hydroxysteroid (17-beta) dehydrogenase 7 pseudogene 2	0.009368958	1.197695128

HSD17B8	Estradiol 17-beta-dehydrogenase 8 gene:ENSG00000206216 /// Estradiol 17-beta-dehydrogenase 8 gene:ENSG00000112474 /// Estradiol 17-beta-dehydrogenase 8 gene:ENSG00000204228 /// Homo sapiens hydroxysteroid (17-beta) dehydrogenase 8, mRNA (cDNA clone MGC:17181 IMAGE:4213813), complete cds. /// Homo sapiens hydroxysteroid (17-beta) dehydrogenase 8 (HSD17B8), mRNA.	hydroxysteroid (17-beta) dehydrogenase 8	0.006804593	1.255963668
HSD11B2	Corticosteroid 11-beta-dehydrogenase isozyme 2 gene:ENSG00000176387 /// Human 11-beta-hydroxysteroid dehydrogenase type 2 mRNA, complete cds. /// Homo sapiens hydroxysteroid (11-beta) dehydrogenase 2 (HSD11B2), mRNA.	hydroxysteroid (11-beta) dehydrogenase 2	0.007978617	1.237111113
HSD17B3	Testosterone 17-beta-dehydrogenase 3 gene:ENSG00000130948 /// Hydroxysteroid (17-beta) dehydrogenase 3 gene:ENSG00000130948 /// Human 17beta-hydroxysteroid dehydrogenase type 3 mRNA, complete cds. /// Homo sapiens hydroxysteroid (17-beta) dehydrogenase 3 (HSD17B3), mRNA.	hydroxysteroid (17-beta) dehydrogenase 3	0.000628457	-1.537172857
**Functional Group 19**	**Transcript Description**	**Gene Title**	**Bayes.lnp**	**Fold**
PTTG1	Securin gene:ENSG00000164611 /// Securin gene:ENSG00000164611 /// Homo sapiens pituitary tumor transforming gene protein (PTTG) mRNA, complete cds. /// Homo sapiens pituitary tumor-transforming 1 (PTTG1), mRNA.	pituitary tumor-transforming 1	0.006930506	1.144838702

SGOL1	Isoform 3 of Shugoshin-like 1 gene:ENSG00000129810 /// Isoform 7 of Shugoshin-like 1 gene:ENSG00000129810 /// Isoform 1 of Shugoshin-like 1 gene:ENSG00000129810 /// Homo sapiens Sgo1 mRNA for shugoshin 1EF protein, complete cds. /// Homo sapiens shugoshin-like 1 (S. pombe) (SGOL1), transcript variant A1, mRNA. /// Homo sapiens shugoshin-like 1 (S. pombe) (SGOL1), transcript variant B1, mRNA. /// Homo sapiens shugoshin-like 1 (S. pombe) (SGOL1), transcript variant C1, mRNA. /// Homo sapiens shugoshin-like 1 (S. pombe) (SGOL1), transcript variant C2, mRNA. /// Homo sapiens shugoshin-like 1 (S. pombe) (SGOL1), transcript variant B2, mRNA. /// Homo sapiens shugoshin-like 1 (S. pombe) (SGOL1), transcript variant A2, mRNA.	shugoshin-like 1 (S. pombe)	0.004828872	1.196577077
PIN1	Peptidyl-prolyl cis-trans isomerase NIMA-interacting 1 gene:ENSG00000127445 /// PIN1 protein gene:ENSG00000127445 /// Homo sapiens cDNA FLJ35651 fis, clone SPLEN2013670. /// Homo sapiens peptidylprolyl cis/trans isomerase, NIMA-interacting 1 (PIN1), mRNA.	peptidylprolyl cis/trans isomerase, NIMA-interacting 1	0.008175798	1.157712048
NCAPH	Condensin complex subunit 2 gene:ENSG00000121152 /// Homo sapiens non-SMC condensin I complex, subunit H, mRNA (cDNA clone MGC:4616 IMAGE:3504782), complete cds. /// Homo sapiens non-SMC condensin I complex, subunit H (NCAPH), mRNA.	non-SMC condensin I complex, subunit H	0.002237605	1.190448418
CCDC5	Isoform 1 of Coiled-coil domain-containing protein 5 gene:ENSG00000152240 /// Homo sapiens enhancer of invasion-cluster (HEIC) mRNA, complete cds. /// Homo sapiens coiled-coil domain containing 5 (spindle associated) (CCDC5), mRNA.	coiled-coil domain containing 5 (spindle associated)	0.006056777	1.211486834

MAD2L1	Mitotic spindle assembly checkpoint protein MAD2A gene:ENSG00000164109 /// MAD2 mitotic arrest deficient-like 1 (Yeast), isoform CRA_b gene:ENSG00000164109 /// Homo sapiens MAD2 mitotic arrest deficient-like 1 (yeast), mRNA (cDNA clone MGC:14577 IMAGE:4132892), complete cds. /// Homo sapiens MAD2 mitotic arrest deficient-like 1 (yeast) (MAD2L1), mRNA.	MAD2 mitotic arrest deficient-like 1 (yeast)	0.003259 19	1.169534 787
SPC24	Kinetochore protein Spc24 gene:ENSG00000161888 /// Homo sapiens cDNA FLJ90806 fis, clone Y79AA1000750. /// Homo sapiens SPC24, NDC80 kinetochore complex component, homolog (S. cerevisiae) (SPC24), mRNA.	SPC24, NDC80 kinetochore complex component, homolog (S. cerevisiae)	0.002954 064	1.178830 986
CEP55	Isoform 1 of Centrosomal protein of 55 kDa gene:ENSG00000138180 /// Isoform 2 of Centrosomal protein of 55 kDa gene:ENSG00000138180 /// Homo sapiens URCC6 mRNA for up-regulated in colon cancer 6, complete cds. /// Homo sapiens centrosomal protein 55kDa (CEP55), transcript variant 1, mRNA. /// Homo sapiens centrosomal protein 55kDa (CEP55), transcript variant 2, mRNA.	centrosomal protein 55kDa	0.003988 343	1.184133 567
NUF2	Kinetochore protein Nuf2 gene:ENSG00000143228 /// Kinetochore protein Nuf2 gene:ENSG00000143228 /// Homo sapiens NUF2R mRNA, complete cds. /// Homo sapiens NUF2, NDC80 kinetochore complex component, homolog (S. cerevisiae) (NUF2), transcript variant 2, mRNA. /// Homo sapiens NUF2, NDC80 kinetochore complex component, homolog (S. cerevisiae) (NUF2), transcript variant 1, mRNA.	NUF2, NDC80 kinetochore complex component, homolog (S. cerevisiae)	0.000173 175	1.215092 314
**Functional Group 20**	**Transcript Description**	**Gene Title**	**Bayes.ln p**	**Fold**

CTSL1	Cathepsin L1 gene:ENSG00000135047 /// Cathepsin L1 gene:ENSG00000135047 /// Cathepsin L1 gene:ENSG00000135047 /// Cathepsin L1 gene:ENSG00000135047 /// Homo sapiens cDNA FLJ31037 fis, clone HSYRA2000137, highly similar to CATHEPSIN L PRECURSOR (EC 3.4.22.15). /// Homo sapiens cathepsin L1 (CTSL1), transcript variant 2, mRNA. /// Homo sapiens cathepsin L1 (CTSL1), transcript variant 1, mRNA.	cathepsin L1	0.008398077	-1.150486902
AGA	N(4)-(beta-N-acetylglucosaminyl)-L-asparaginase gene:ENSG00000038002 /// Homo sapiens aspartylglucosaminidase, mRNA (cDNA clone MGC:9008 IMAGE:3859317), complete cds. /// Homo sapiens aspartylglucosaminidase (AGA), mRNA.	aspartylglucosaminidase	0.008023485	1.248389356
CTSC	Dipeptidyl-peptidase 1 gene:ENSG00000109861 /// Putative uncharacterized protein CTSC gene:ENSG00000109861 /// cathepsin C isoform c precursor gene:ENSG00000109861 /// cathepsin C isoform b precursor gene:ENSG00000109861 /// Homo sapiens cDNA FLJ55694 complete cds, highly similar to Dipeptidyl-peptidase 1 precursor (EC 3.4.14.1). /// Homo sapiens cathepsin C (CTSC), transcript variant 2, mRNA. /// Homo sapiens cathepsin C (CTSC), transcript variant 3, mRNA. /// Homo sapiens cathepsin C (CTSC), transcript variant 1, mRNA.	cathepsin C	0.000472055	1.213306947
CTSZ	34 kDa protein gene:ENSG00000101160 /// Cathepsin Z gene:ENSG00000101160 /// Homo sapiens cathepsin X precursor, mRNA, complete cds. /// Homo sapiens cathepsin Z (CTSZ), mRNA.	cathepsin Z	0.00861216	1.16781971

PCSK1	Neuroendocrine convertase 1 gene:ENSG00000175426 /// H.sapiens PC1 (NEC1) mRNA, complete cds. /// Homo sapiens proprotein convertase subtilisin/kexin type 1 (PCSK1), mRNA.	proprotein convertase subtilisin/kexin type 1	0.001541315	-1.474321169
**Functional Group 21**	**Transcript Description**	**Gene Title**	**Bayes.lnp**	**Fold**
NXF1	Nuclear RNA export factor 1 gene:ENSG00000162231 /// Homo sapiens nuclear RNA export factor 1, mRNA (cDNA clone MGC:40096 IMAGE:5248449), complete cds. /// Homo sapiens nuclear RNA export factor 1 (NXF1), transcript variant 1, mRNA. /// Homo sapiens nuclear RNA export factor 1 (NXF1), transcript variant 2, mRNA.	nuclear RNA export factor 1	0.000406125	-1.234504541
RANGAP1	Ran GTPase-activating protein 1 gene:ENSG00000100401 /// Ran GTPase-activating protein 1 gene:ENSG00000100401 /// Ran GTPase-activating protein 1 gene:ENSG00000100401 /// Ran GTPase-activating protein 1 gene:ENSG00000100401 /// Homo sapiens mRNA for KIAA1835 protein, partial cds. /// Homo sapiens Ran GTPase activating protein 1 (RANGAP1), mRNA.	Ran GTPase activating protein 1	0.009675834	-1.158389866

ANP32E	acidic (leucine-rich) nuclear phosphoprotein 32 family, member E isoform 3 gene:ENSG00000143401 /// Acidic leucine-rich nuclear phosphoprotein 32 family member E gene:ENSG00000143401 /// Acidic (Leucine-rich) nuclear phosphoprotein 32 family, member E gene:ENSG00000143401 /// Acidic (Leucine-rich) nuclear phosphoprotein 32 family, member E gene:ENSG00000143401 /// Acidic (Leucine-rich) nuclear phosphoprotein 32 family, member E gene:ENSG00000143401 /// Homo sapiens acidic (leucine-rich) nuclear phosphoprotein 32 family, member E, mRNA (cDNA clone MGC:5350 IMAGE:3047915), complete cds. /// Homo sapiens acidic (leucine-rich) nuclear phosphoprotein 32 family, member E (ANP32E), transcript variant 3, mRNA. /// Homo sapiens acidic (leucine-rich) nuclear phosphoprotein 32 family, member E (ANP32E), transcript variant 2, mRNA. /// Homo sapiens acidic (leucine-rich) nuclear phosphoprotein 32 family, member E (ANP32E), transcript variant 1, mRNA.	acidic (leucine-rich) nuclear phosphoprotein 32 family, member E	0.002654353	1.194348264
ANP32A	ANP32A protein (Fragment) gene:ENSG00000140350 /// cdna:known chromosome:NCBI36:15:66857928:66900261:-1 gene:ENSG00000140350 /// Acidic leucine-rich nuclear phosphoprotein 32 family member A gene:ENSG00000140350 /// hypothetical protein gene:ENSG00000214746 /// Homo sapiens acidic (leucine-rich) nuclear phosphoprotein 32 family, member A, mRNA (cDNA clone MGC:12667 IMAGE:3677623), complete cds. /// Homo sapiens acidic (leucine-rich) nuclear phosphoprotein 32 family, member A (ANP32A), mRNA.	acidic (leucine-rich) nuclear phosphoprotein 32 family, member A	0.000721366	1.225751887

Functional Group 22	Transcript Description	Gene Title	Bayes.lnp	Fold
MFAP5	Microfibrillar-associated protein 5 gene:ENSG00000197614 /// cDNA FLJ54936, highly similar to Microfibrillar-associated protein 5 gene:ENSG00000197614 /// Homo sapiens microfibrillar associated protein 5, mRNA (cDNA clone MGC:14490 IMAGE:4247343), complete cds. /// Homo sapiens microfibrillar associated protein 5 (MFAP5), mRNA.	microfibrillar associated protein 5	0.000367671	1.26566102
MAMDC2	Isoform 1 of MAM domain-containing protein 2 gene:ENSG00000165072 /// Homo sapiens MAM domain containing 2, mRNA (cDNA clone MGC:75273 IMAGE:4399866), complete cds. /// Homo sapiens MAM domain containing 2 (MAMDC2), mRNA.	MAM domain containing 2	0.000138833	1.243509214
OLFML3	Isoform 1 of Olfactomedin-like protein 3 gene:ENSG00000116774 /// Isoform 2 of Olfactomedin-like protein 3 gene:ENSG00000116774 /// Isoform 2 of Olfactomedin-like protein 3 gene:ENSG00000116774 /// Homo sapiens clone DNA64905 olfactomedin-like (UNQ663) mRNA, complete cds. /// Homo sapiens olfactomedin-like 3 (OLFML3), mRNA.	olfactomedin-like 3	2.91462E-05	1.316797443
SMPDL3A	Acid sphingomyelinase-like phosphodiesterase 3a gene:ENSG00000172594 /// Homo sapiens sphingomyelin phosphodiesterase, acid-like 3A, mRNA (cDNA clone MGC:20681 IMAGE:3138813), complete cds. /// Homo sapiens sphingomyelin phosphodiesterase, acid-like 3A (SMPDL3A), mRNA.	sphingomyelin phosphodiesterase, acid-like 3A	0.003253057	1.458250503

CA11	Carbonic anhydrase-related protein 11 gene:ENSG00000063180 /// Homo sapiens ca xi mRNA for carbonic anhydrase-related protein XI, complete cds. /// Homo sapiens carbonic anhydrase XI (CA11), mRNA.	carbonic anhydrase XI	0.000811052	1.252656016
FAM20B	Protein FAM20B gene:ENSG00000116199 /// Homo sapiens family with sequence similarity 20, member B, mRNA (cDNA clone MGC:57628 IMAGE:5492043), complete cds. /// Homo sapiens family with sequence similarity 20, member B (FAM20B), mRNA.	family with sequence similarity 20, member B	0.003883056	1.168512014
**Functional Group 23**	**Transcript Description**	**Gene Title**	**Bayes.lnp**	**Fold**
VARS	Valyl-tRNA synthetase gene:ENSG00000096171 /// Valyl-tRNA synthetase gene:ENSG00000204394 /// Homo sapiens valyl-tRNA synthetase, mRNA (cDNA clone MGC:3537 IMAGE:3529215), complete cds. /// Homo sapiens valyl-tRNA synthetase (VARS), nuclear gene encoding mitochondrial protein, mRNA.	valyl-tRNA synthetase	0.007596587	1.153742143
DARS	Aspartyl-tRNA synthetase, cytoplasmic gene:ENSG00000115866 /// Human aspartyl-tRNA synthetase alpha-2 subunit mRNA, complete cds. /// Homo sapiens aspartyl-tRNA synthetase (DARS), mRNA.	aspartyl-tRNA synthetase	0.00105772	1.226792589
HARS2	Probable histidyl-tRNA synthetase, mitochondrial gene:ENSG00000112855 /// Human histidyl-tRNA synthetase homolog (HO3) mRNA, complete cds. /// Homo sapiens histidyl-tRNA synthetase 2, mitochondrial (putative) (HARS2), nuclear gene encoding mitochondrial protein, mRNA.	histidyl-tRNA synthetase 2, mitochondrial (putative)	0.00124126	1.225075123

IARS2	Isoleucyl-tRNA synthetase, mitochondrial gene:ENSG00000067704 /// Isoleucyl-tRNA synthetase, mitochondrial gene:ENSG00000067704 /// Homo sapiens isoleucine tRNA synthetase mRNA, complete cds; nuclear gene for mitochondrial product. /// Homo sapiens isoleucyl-tRNA synthetase 2, mitochondrial (IARS2), nuclear gene encoding mitochondrial protein, mRNA.	isoleucyl-tRNA synthetase 2, mitochondrial	0.000497135	1.223551329
PLXNB2	16 kDa protein gene:ENSG00000196576 /// Plexin B2 gene:ENSG00000196576 /// Plexin-B2 gene:ENSG00000196576 /// Clone MM1 product (Fragment) gene:ENSG00000196576 /// Homo sapiens cDNA FLJ31981 fis, clone NT2RP7008543, weakly similar to PLEXIN 4 PRECURSOR. /// Homo sapiens plexin B2 (PLXNB2), mRNA.	plexin B2	0.000209484	-1.237411763
**Functional Group 24**	**Transcript Description**	**Gene Title**	**Bayes.lnp**	**Fold**
SEPSECS	Isoform 1 of O-phosphoseryl-tRNA(Sec) selenium transferase gene:ENSG00000109618 /// Isoform 2 of O-phosphoseryl-tRNA(Sec) selenium transferase gene:ENSG00000109618 /// Isoform 1 of O-phosphoseryl-tRNA(Sec) selenium transferase gene:ENSG00000109618 /// Homo sapiens mRNA; cDNA DKFZp686J1361 (from clone DKFZp686J1361). /// Homo sapiens Sep (O-phosphoserine) tRNA:Sec (selenocysteine) tRNA synthase (SEPSECS), transcript variant 2, mRNA. /// Homo sapiens Sep (O-phosphoserine) tRNA:Sec (selenocysteine) tRNA synthase (SEPSECS), transcript variant 1, mRNA.	Sep (O-phosphoserine) tRNA:Sec (selenocysteine) tRNA synthase	0.002586286	1.22761721

EIF4B	cDNA FLJ54492, highly similar to Eukaryotic translation initiation factor 4B gene:ENSG00000063046 /// Homo sapiens eukaryotic translation initiation factor 4B, mRNA (cDNA clone MGC:88035 IMAGE:6527540), complete cds. /// Homo sapiens eukaryotic translation initiation factor 4B (EIF4B), mRNA.	eukaryotic translation initiation factor 4B	0.188822525	1.066263671
EIF2C1	Eukaryotic translation initiation factor 2C, 1 gene:ENSG00000092847 /// Eukaryotic translation initiation factor 2C 1 gene:ENSG00000092847 /// Homo sapiens putative RNA-binding protein Q99 mRNA, complete cds. /// Homo sapiens eukaryotic translation initiation factor 2C, 1 (EIF2C1), mRNA.	eukaryotic translation initiation factor 2C, 1	0.006046515	1.163962818
CPEB2	66 kDa protein gene:ENSG00000137449 /// Isoform 1 of Cytoplasmic polyadenylation element-binding protein 2 gene:ENSG00000137449 /// Isoform 5 of Cytoplasmic polyadenylation element-binding protein 2 gene:ENSG00000137449 /// Isoform 4 of Cytoplasmic polyadenylation element-binding protein 2 gene:ENSG00000137449 /// Isoform 2 of Cytoplasmic polyadenylation element-binding protein 2 gene:ENSG00000137449 /// Homo sapiens cytoplasmic polyadenylation element binding protein 2, mRNA (cDNA clone MGC:119575 IMAGE:40008489), complete cds. /// Homo sapiens cytoplasmic polyadenylation element binding protein 2 (CPEB2), transcript variant B, mRNA. /// Homo sapiens cytoplasmic polyadenylation element binding protein 2 (CPEB2), transcript variant A, mRNA.	cytoplasmic polyadenylation element binding protein 2	0.001362494	-1.227181426

EIF4E2	Eukaryotic translation initiation factor 4E type 2 gene:ENSG00000135930 /// cdna:known chromosome:NCBI36:2:233123637:233156598:1 gene:ENSG00000135930 /// cdna:known chromosome:NCBI36:2:233123637:233156596:1 gene:ENSG00000135930 /// Eukaryotic translation initiation factor 4E member 2 variant (Fragment) gene:ENSG00000135930 /// cdna:known chromosome:NCBI36:2:233123643:233140974:1 gene:ENSG00000135930 /// cdna:known chromosome:NCBI36:2:233123643:233140481:1 gene:ENSG00000135930 /// cdna:known chromosome:NCBI36:2:233123646:233142132:1 gene:ENSG00000135930 /// cdna:known chromosome:NCBI36:2:233123658:233153910:1 gene:ENSG00000135930 /// Homo sapiens eukaryotic translation initiation factor 4E family member 2, mRNA (cDNA clone MGC:12525 IMAGE:3997986), complete cds. /// Homo sapiens eukaryotic translation initiation factor 4E family member 2 (EIF4E2), mRNA.	eukaryotic translation initiation factor 4E family member 2	0.00951327	1.169079777
**Functional Group 25**	**Transcript Description**	**Gene Title**	**Bayes.lnp**	**Fold**
NID1	Isoform 1 of Nidogen-1 gene:ENSG00000116962 /// Isoform 2 of Nidogen-1 gene:ENSG00000116962 /// Homo sapiens nidogen 1, mRNA (cDNA clone MGC:33141 IMAGE:5271590), complete cds. /// Homo sapiens nidogen 1 (NID1), mRNA.	nidogen 1	6.38654E-05	-1.269876893

FAT3	cdna:known chromosome:NCBI36:11:91724910:92269284:1 gene:ENSG00000165323 /// Isoform 1 of Protocadherin Fat 3 gene:ENSG00000165323 /// Homo sapiens FAT tumor suppressor homolog 3 (Drosophila) (FAT3), mRNA.	FAT tumor suppressor homolog 3 (Drosophila)	0.000181836	-1.302095111
NELL2	NEL-like 2 (Chicken), isoform CRA_a gene:ENSG00000184613 /// Protein kinase C-binding protein NELL2 gene:ENSG00000184613 /// Homo sapiens mRNA for nel-related protein 2, complete cds. /// Homo sapiens NEL-like 2 (chicken) (NELL2), mRNA.	NEL-like 2 (chicken)	0.009863348	-1.150640829
FBLN1	Isoform B of Fibulin-1 gene:ENSG00000077942 /// Isoform D of Fibulin-1 gene:ENSG00000077942 /// fibulin 1 isoform B precursor gene:ENSG00000077942 /// Isoform C of Fibulin-1 gene:ENSG00000077942 /// Isoform A of Fibulin-1 gene:ENSG00000077942 /// Fibulin 1 gene:ENSG00000077942 /// Homo sapiens fibulin-1 isoform D precursor, mRNA, complete cds. /// Homo sapiens fibulin 1 (FBLN1), transcript variant C, mRNA. /// Homo sapiens fibulin 1 (FBLN1), transcript variant D, mRNA. /// Homo sapiens fibulin 1 (FBLN1), transcript variant A, mRNA. /// Homo sapiens fibulin 1 (FBLN1), transcript variant B, mRNA.	fibulin 1	0.006874576	-1.1626722

FBLN7	Isoform 1 of Fibulin-7 gene:ENSG00000144152 /// cdna:known chromosome:NCBI36:2:112612474:112662246:1 gene:ENSG00000144152 /// cdna:known chromosome:NCBI36:2:112612631:112661693:1 gene:ENSG00000144152 /// cdna:known chromosome:NCBI36:2:112612631:112661687:1 gene:ENSG00000144152 /// Isoform 2 of Fibulin-7 gene:ENSG00000144152 /// Homo sapiens fibulin 7, mRNA (cDNA clone MGC:46268 IMAGE:5589120), complete cds. /// Homo sapiens fibulin 7 (FBLN7), transcript variant 1, mRNA. /// Homo sapiens fibulin 7 (FBLN7), transcript variant 2, mRNA.	fibulin 7	0.009131177	-1.274710834
SVEP1	391 kDa protein gene:ENSG00000165124 /// polydom gene:ENSG00000165124 /// Isoform 3 of Sushi, von Willebrand factor type A, EGF and pentraxin domain-containing protein 1 gene:ENSG00000165124 /// polydom gene:ENSG00000165124 /// Isoform 1 of Sushi, von Willebrand factor type A, EGF and pentraxin domain-containing protein 1 gene:ENSG00000165124 /// Isoform 2 of Sushi, von Willebrand factor type A, EGF and pentraxin domain-containing protein 1 gene:ENSG00000165124 /// Homo sapiens selectin-like protein mRNA, complete cds. /// Homo sapiens sushi, von Willebrand factor type A, EGF and pentraxin domain containing 1 (SVEP1), mRNA.	sushi, von Willebrand factor type A, EGF and pentraxin domain containing 1	0.000315517	-1.322326065

NOTCH2NL	cdna:known supercontig::NT_113872:169895:176054:1 gene:ENSG00000215620 /// NBPF8 isoform 4 gene:ENSG00000179571 /// Uncharacterized protein ENSP00000358372 gene:ENSG00000162825 /// Uncharacterized protein ENSP00000358380 (Fragment) gene:ENSG00000162825 /// Putative uncharacterized protein ENSP00000349255 gene:ENSG00000162825 /// Uncharacterized protein ENSP00000358384 (Fragment) gene:ENSG00000162825 /// Putative uncharacterized protein ENSP00000358379 (Fragment) gene:ENSG00000162825 /// NBPF8 isoform 2 gene:ENSG00000186275 /// 70 kDa protein gene:ENSG00000168614 /// Neuroblastoma breakpoint family member 20 gene:ENSG00000168614 /// Isoform 2 of Notch homolog 2 N-terminal-like protein gene:ENSG00000213240 /// Isoform 2 of Notch homolog 2 N-terminal-like protein gene:ENSG00000213240 /// Novel protein gene:ENSG00000163386 /// Putative uncharacterized protein NBPF9 gene:ENSG00000163386 /// Putative uncharacterized protein NBPF9 gene:ENSG00000163386 /// Putative uncharacterized protein NBPF9 gene:ENSG00000163386 /// Putative uncharacterized protein NBPF9 gene:ENSG00000163386 /// cDNA FLJ60244, weakly similar to Homo sapiens phosphodiesterase 4D interacting protein, transcript variant 1, mRNA gene:ENSG00000163386 /// Putative uncharacterized protein NBPF9 gene:ENSG00000163386 /// Putative uncharacterized protein NBPF9 gene:ENSG00000163386 ///	KIAA1245 /// neuroblastoma breakpoint family, member 1 /// Notch homolog 2 (Drosophila) N-terminal like /// neuroblastoma breakpoint family, member 9 /// neuroblastoma breakpoint family, member 14 /// hypothetical protein LOC200030 /// neuroblastoma breakpoint family, member 20 /// neuroblastoma breakpoint family, member 10 /// neuroblastoma breakpoint family, member 15 /// neuroblastoma breakpoint family, member 8 /// neuroblastoma breakpoint family, member 16	0.008807102	-1.129728487

	Putative uncharacterized protein NBPF9 gene:ENSG00000163386 /// Putative uncharacterized protein NBPF9 gene:ENSG00000163386 /// Putative uncharacterized protein NBPF9 gene:ENSG00000163386 /// 104 kDa protein gene:ENSG00000152042 /// 97 kDa protein gene:ENSG00000152042 /// Neuroblastoma breakpoint family member 11 gene:ENSG00000152042 /// hypothetical protein LOC200030 gene:ENSG00000152042 /// NBPF8 isoform 5 gene:ENSG00000152042 /// hypothetical protein gene:ENSG00000187820 /// 104 kDa protein gene:ENSG00000203836 /// 97 kDa protein gene:ENSG00000203836 /// Neuroblastoma breakpoint family member 21 gene:ENSG00000203836 /// Neuroblastoma breakpoint family, member 11 gene:ENSG00000203836 /// 90 kDa protein gene:ENSG00000203836 /// Neuroblastoma breakpoint family member 14 gene:ENSG00000122497 /// 71 kDa protein gene:ENSG00000122497 /// Neuroblastoma breakpoint family member 16 gene:ENSG00000122497 /// Putative uncharacterized protein NBPF14 gene:ENSG00000122497 /// Neuroblastoma breakpoint family, member 10 gene:ENSG00000203832 /// Novel protein gene:ENSG00000203832 /// Putative uncharacterized protein NBPF8 gene:ENSG00000203832 /// Putative uncharacterized protein NBPF8 gene:ENSG00000203832 /// CG10522-PA-like gene:ENSG00000203832 /// Putative uncharacterized protein NBPF8 gene:ENSG00000203832 /// Putative uncharacterized protein NBPF8 (Fragment) gene:ENSG00000203832 /// Neuroblastoma breakpoint family,			

	member 10 gene:ENSG00000203832 /// Isoform 2 of Neuroblastoma breakpoint family member 10 gene:ENSG00000203832 /// Neuroblastoma breakpoint family, member 15 gene:ENSG00000219481 /// Putative uncharacterized protein NBPF15 gene:ENSG00000219481 /// Neuroblastoma breakpoint family, member 16 gene:ENSG00000203827 /// Putative uncharacterized protein NBPF16 (Fragment) gene:ENSG00000203827 /// Homo sapiens mRNA for KIAA1245 protein, partial cds. /// Homo sapiens mRNA for KIAA1693 protein, partial cds. /// Homo sapiens cDNA FLJ90584 fis, clone PLACE1000740, highly similar to Neurogenic locus notch homolog protein 2 precursor. /// Homo sapiens cDNA FLJ60244 complete cds, weakly similar to Homo sapiens phosphodiesterase 4D interacting protein, transcript variant 1, mRNA. /// Homo sapiens cDNA FLJ60520 complete cds, weakly similar to Homo sapiens phosphodiesterase 4D interacting protein, transcript variant 1, mRNA. /// Homo sapiens mRNA; cDNA DKFZp451B1418 (from clone DKFZp451B1418); complete cds. /// Homo sapiens clone IIIc8 NBPF9 isoform 2 mRNA, complete cds, alternatively spliced. /// Homo sapiens neuroblastoma breakpoint family, member 10, mRNA (cDNA clone IMAGE:5090304). /// Homo sapiens neuroblastoma breakpoint family, member 15, mRNA (cDNA clone MGC:8902 IMAGE:3918932), complete cds. /// Homo sapiens neuroblastoma breakpoint family, member 8, mRNA (cDNA clone MGC:99641 IMAGE:5453529), complete cds. /// Homo sapiens hypothetical protein LOC200030 (LOC200030), mRNA. /// Homo sapiens neuroblastoma breakpoint			

	family, member 16 (NBPF16), mRNA. /// Homo sapiens neuroblastoma breakpoint family, member 14 (NBPF14), mRNA.			
**Functional Group 26**	**Transcript Description**	**Gene Title**	**Bayes.ln p**	**Fold**
HK2	Hexokinase-2 gene:ENSG00000159399 /// cdna:known chromosome:NCBI36:2:74915805:74973994:1 gene:ENSG00000159399 /// Homo sapiens hexokinase 2, mRNA (cDNA clone MGC:74789 IMAGE:5586773), complete cds. /// Homo sapiens hexokinase 2 (HK2), mRNA.	hexokinase 2	9.23437E-09	1.598668999

PFKM	cDNA FLJ44241 fis, clone THYMU3008436, highly similar to 6-phosphofructokinase, muscle type gene:ENSG00000152556 /// Isoform 1 of 6-phosphofructokinase, muscle type gene:ENSG00000152556 /// Isoform 2 of 6-phosphofructokinase, muscle type gene:ENSG00000152556 /// Homo sapiens cDNA FLJ44241 fis, clone THYMU3008436, highly similar to 6-phosphofructokinase, muscle type (EC 2.7.1.11). /// Homo sapiens phosphofructokinase, muscle (PFKM), mRNA.	phosphofructokinase, muscle	0.008182424	1.16915528
ENO2	Gamma-enolase gene:ENSG00000111674 /// Homo sapiens enolase 2 (gamma, neuronal), mRNA (cDNA clone MGC:3520 IMAGE:3629603), complete cds. /// Homo sapiens enolase 2 (gamma, neuronal) (ENO2), mRNA.	enolase 2 (gamma, neuronal)	0.00467409	-1.163118025
HIBADH	3-hydroxyisobutyrate dehydrogenase, mitochondrial gene:ENSG00000106049 /// Homo sapiens mRNA for 3'-hydroxyisobutyrate dehydrogenase, complete cds. /// Homo sapiens 3-hydroxyisobutyrate dehydrogenase (HIBADH), mRNA.	3-hydroxyisobutyrate dehydrogenase	0.000475911	1.196599175
PDHB	Isoform 2 of Pyruvate dehydrogenase E1 component subunit beta, mitochondrial gene:ENSG00000168291 /// Isoform 1 of Pyruvate dehydrogenase E1 component subunit beta, mitochondrial gene:ENSG00000168291 /// Homo sapiens mRNA for Pyruvate dehydrogenase E1 component beta subunit, mitochondrial precursor variant, clone: SPL02706. /// Homo sapiens pyruvate dehydrogenase (lipoamide) beta (PDHB), mRNA.	pyruvate dehydrogenase (lipoamide) beta	0.008282689	1.151141848

ALDOC	Fructose-bisphosphate aldolase C gene:ENSG00000109107 /// Fructose-bisphosphate aldolase gene:ENSG00000109107 /// Fructose-bisphosphate aldolase C gene:ENSG00000109107 /// Homo sapiens aldolase C, fructose-bisphosphate, mRNA (cDNA clone MGC:1449 IMAGE:2987869), complete cds. /// Homo sapiens aldolase C, fructose-bisphosphate (ALDOC), mRNA.	aldolase C, fructose-bisphosphate	0.000115049	1.286917053
LDHA	Isoform 1 of L-lactate dehydrogenase A chain gene:ENSG00000134333 /// Isoform 2 of L-lactate dehydrogenase A chain gene:ENSG00000134333 /// L-lactate dehydrogenase gene:ENSG00000134333 /// Homo sapiens lactate dehydrogenase A, mRNA (cDNA clone MGC:72033 IMAGE:4096518), complete cds. /// Homo sapiens lactate dehydrogenase A (LDHA), transcript variant 1, mRNA. /// Homo sapiens lactate dehydrogenase A (LDHA), transcript variant 2, mRNA.	lactate dehydrogenase A	0.003721276	1.1839922
**Functional Group 27**	**Transcript Description**	**Gene Title**	**Bayes.lnp**	**Fold**
IGFBP5	Insulin-like growth factor-binding protein 5 gene:ENSG00000115461 /// Human insulin-like growth factor binding protein 5 (IGFBP-5) mRNA, complete cds. /// Homo sapiens insulin-like growth factor binding protein 5 (IGFBP5), mRNA.	insulin-like growth factor binding protein 5	1.11688E-13	2.172203639
IGFBP2	Insulin-like growth factor-binding protein 2 gene:ENSG00000115457 /// Homo sapiens insulin-like growth factor binding protein 2, 36kDa, mRNA (cDNA clone MGC:10918 IMAGE:3627826), complete cds. /// Homo sapiens insulin-like growth factor binding protein 2, 36kDa (IGFBP2), mRNA.	insulin-like growth factor binding protein 2, 36kDa	0.004095392	1.163713326

IGFBP4	Insulin-like growth factor-binding protein 4 gene:ENSG00000141753 /// Homo sapiens insulin-like growth factor binding protein 4, mRNA (cDNA clone MGC:20162 IMAGE:4661581), complete cds. /// Homo sapiens insulin-like growth factor binding protein 4 (IGFBP4), mRNA.	insulin-like growth factor binding protein 4	7.12128E-06	-1.30573276
IGFBP6	Insulin-like growth factor-binding protein 6 gene:ENSG00000167779 /// Homo sapiens insulin-like growth factor binding protein 6, mRNA (cDNA clone MGC:19772 IMAGE:3640964), complete cds. /// Homo sapiens insulin-like growth factor binding protein 6 (IGFBP6), mRNA.	insulin-like growth factor binding protein 6	6.2741E-05	1.244184148
**Functional Group 28**	**Transcript Description**	**Gene Title**	**Bayes.lnp**	**Fold**
CA14	Carbonic anhydrase 14 gene:ENSG00000118298 /// Homo sapiens cDNA FLJ90284 fis, clone NT2RP1000613, weakly similar to CARBONIC ANHYDRASE VI (EC 4.2.1.1). /// Homo sapiens carbonic anhydrase XIV (CA14), mRNA.	carbonic anhydrase XIV	0.000351389	1.233556271
CA13	Carbonic anhydrase 13 gene:ENSG00000185015 /// Homo sapiens carbonic anhydrase XIII, mRNA (cDNA clone MGC:59868 IMAGE:6083105), complete cds. /// Homo sapiens carbonic anhydrase XIII (CA13), mRNA.	carbonic anhydrase XIII	0.002780801	-1.337627917
CA8	Carbonic anhydrase-related protein gene:ENSG00000178538 /// Homo sapiens carbonic anhydrase VIII, mRNA (cDNA clone MGC:120502 IMAGE:40025442), complete cds. /// Homo sapiens carbonic anhydrase VIII (CA8), mRNA.	carbonic anhydrase VIII	9.14267E-06	-1.437893272

CA6	CA6 protein gene:ENSG00000131686 /// Carbonic anhydrase VI nirs variant 1 gene:ENSG00000131686 /// cDNA FLJ60163, highly similar to Carbonic anhydrase 6 gene:ENSG00000131686 /// Carbonic anhydrase VI nirs variant 3 gene:ENSG00000131686 /// Human carbonic anhydrase isozyme VI (CA6) mRNA, complete cds. /// Homo sapiens carbonic anhydrase VI (CA6), mRNA.	carbonic anhydrase VI	0.009748868	-1.334004402
NOB1	RNA-binding protein NOB1 gene:ENSG00000141101 /// Homo sapiens RNA-binding protein NOB1 mRNA, complete cds. /// Homo sapiens NIN1/RPN12 binding protein 1 homolog (S. cerevisiae) (NOB1), mRNA.	NIN1/RPN12 binding protein 1 homolog (S. cerevisiae)	0.003266145	1.19290308
CA10	Carbonic anhydrase-related protein 10 gene:ENSG00000154975 /// Putative uncharacterized protein DKFZp686I20267 gene:ENSG00000154975 /// Homo sapiens carbonic anhydrase X, mRNA (cDNA clone MGC:87062 IMAGE:5260321), complete cds. /// Homo sapiens carbonic anhydrase X (CA10), transcript variant 2, mRNA. /// Homo sapiens carbonic anhydrase X (CA10), transcript variant 1, mRNA. /// Homo sapiens carbonic anhydrase X (CA10), transcript variant 3, mRNA.	carbonic anhydrase X	0.000139323	-1.481043797
CA11	Carbonic anhydrase-related protein 11 gene:ENSG00000063180 /// Homo sapiens ca xi mRNA for carbonic anhydrase-related protein XI, complete cds. /// Homo sapiens carbonic anhydrase XI (CA11), mRNA.	carbonic anhydrase XI	0.000811052	1.252656016
LOC91431	Isoform 1 of Uncharacterized protein FLJ44066 gene:ENSG00000188014 /// Homo sapiens mRNA; cDNA DKFZp313L226 (from clone DKFZp313L226). /// Homo sapiens prematurely terminated mRNA decay factor-like (LOC91431), mRNA.	prematurely terminated mRNA decay factor-like	0.000309304	-1.283344003

Functional Group 29	Transcript Description	Gene Title	Bayes.ln p	Fold
FEZ1	Isoform Short of Fasciculation and elongation protein zeta-1 gene:ENSG00000149557 /// Isoform Long of Fasciculation and elongation protein zeta-1 gene:ENSG00000149557 /// Homo sapiens fasciculation and elongation protein zeta 1 (zygin I), mRNA (cDNA clone MGC:10085 IMAGE:3897353), complete cds. /// Homo sapiens fasciculation and elongation protein zeta 1 (zygin I) (FEZ1), transcript variant 2, mRNA. /// Homo sapiens fasciculation and elongation protein zeta 1 (zygin I) (FEZ1), transcript variant 1, mRNA.	fasciculation and elongation protein zeta 1 (zygin I)	0.000100679	-1.260217091
SLITRK1	SLIT and NTRK-like protein 1 gene:ENSG00000178235 /// Homo sapiens SLIT and NTRK-like family, member 1, mRNA (cDNA clone MGC:51091 IMAGE:4816570), complete cds. /// Homo sapiens SLIT and NTRK-like family, member 1 (SLITRK1), mRNA.	SLIT and NTRK-like family, member 1	0.001538595	-1.29836266
CDH4	Cadherin-4 gene:ENSG00000179242 /// Homo sapiens cadherin 4, type 1, R-cadherin (retinal), mRNA (cDNA clone MGC:126700 IMAGE:8069157), complete cds. /// Homo sapiens cadherin 4, type 1, R-cadherin (retinal) (CDH4), mRNA.	cadherin 4, type 1, R-cadherin (retinal)	0.000300329	-1.313258303

LOC440258	My020 protein gene:ENSG00000104765 /// cDNA FLJ51086, highly similar to BCL2/adenovirus E1B 19 kDa protein-interacting protein 3 gene:ENSG00000104765 /// Homo sapiens ovarian epithelial carcinoma-related protein mRNA, complete cds. /// Homo sapiens BCL2/adenovirus E1B 19kDa interacting protein 3-like, mRNA (cDNA clone MGC:14695 IMAGE:4134765), complete cds. /// Homo sapiens BCL2/adenovirus E1B 19kDa interacting protein 3-like (BNIP3L), mRNA.	similar to p40 /// BCL2/adenovirus E1B 19kDa interacting protein 3-like	9.63E-06	1.325576518
ARTN	Isoform 1 of Artemin gene:ENSG00000117407 /// Isoform 3 of Artemin gene:ENSG00000117407 /// Isoform 1 of Artemin gene:ENSG00000117407 /// Homo sapiens pre-pro-neublastin mRNA, complete cds. /// Homo sapiens artemin (ARTN), transcript variant 5, mRNA. /// Homo sapiens artemin (ARTN), transcript variant 1, mRNA. /// Homo sapiens artemin (ARTN), transcript variant 4, mRNA. /// Homo sapiens artemin (ARTN), transcript variant 2, mRNA. /// Homo sapiens artemin (ARTN), transcript variant 3, mRNA.	artemin	0.009880482	1.225593498
NTN1	Netrin-1 gene:ENSG00000065320 /// Human netrin-1 mRNA, complete cds. /// Homo sapiens netrin 1 (NTN1), mRNA.	netrin 1	6.24025E-05	-1.308916499
**Functional Group 30**	**Transcript Description**	**Gene Title**	**Bayes.lnp**	**Fold**

LIMA1	LIM domain and actin binding 1 isoform a gene:ENSG00000050405 /// Isoform Beta of LIM domain and actin-binding protein 1 gene:ENSG00000050405 /// Homo sapiens LIM domain and actin binding 1, mRNA (cDNA clone MGC:131726 IMAGE:5115521), complete cds. /// Homo sapiens LIM domain and actin binding 1 (LIMA1), transcript variant b, mRNA. /// Homo sapiens LIM domain and actin binding 1 (LIMA1), transcript variant a, mRNA. /// Homo sapiens LIM domain and actin binding 1 (LIMA1), transcript variant c, mRNA.	LIM domain and actin binding 1	4.27226E-05	-1.266518523
SPTAN1	cDNA FLJ61399, highly similar to Spectrin alpha chain, brain gene:ENSG00000197694 /// Isoform 1 of Spectrin alpha chain, brain gene:ENSG00000197694 /// Putative uncharacterized protein SPTAN1 gene:ENSG00000197694 /// Putative uncharacterized protein SPTAN1 gene:ENSG00000197694 /// Isoform 2 of Spectrin alpha chain, brain gene:ENSG00000197694 /// Homo sapiens SPTAN1 mRNA for non-erythrocytic spectrin alpha, complete cds. /// Homo sapiens spectrin, alpha, non-erythrocytic 1 (alpha-fodrin) (SPTAN1), transcript variant 2, mRNA. /// Homo sapiens spectrin, alpha, non-erythrocytic 1 (alpha-fodrin) (SPTAN1), transcript variant 1, mRNA.	spectrin, alpha, non-erythrocytic 1 (alpha-fodrin)	0.007009349	-1.141263648
AVIL	Advillin gene:ENSG00000135407 /// Homo sapiens advillin mRNA, complete cds. /// Homo sapiens advillin (AVIL), mRNA.	advillin	0.083204586	-1.253473055

SCIN	Isoform 2 of Adseverin gene:ENSG00000006747 /// Isoform 1 of Adseverin gene:ENSG00000006747 /// Homo sapiens cDNA FLJ78088 complete cds. /// Homo sapiens scinderin (SCIN), transcript variant 2, mRNA. /// Homo sapiens scinderin (SCIN), transcript variant 1, mRNA.	scinderin	6.6255E-05	-1.709219593
GSN	Isoform 2 of Gelsolin gene:ENSG00000148180 /// Isoform 2 of Gelsolin gene:ENSG00000148180 /// Isoform 1 of Gelsolin gene:ENSG00000148180 /// gelsolin isoform c gene:ENSG00000148180 /// cDNA FLJ35478 fis, clone SMINT2007796, highly similar to Gelsolin gene:ENSG00000148180 /// Gelsolin gene:ENSG00000148180 /// Gelsolin gene:ENSG00000148180 /// Gelsolin gene:ENSG00000148180 /// Gelsolin gene:ENSG00000148180 /// Homo sapiens gelsolin (amyloidosis, Finnish type), mRNA (cDNA clone MGC:39262 IMAGE:4661084), complete cds. /// Homo sapiens gelsolin (amyloidosis, Finnish type) (GSN), transcript variant 8, mRNA. /// Homo sapiens gelsolin (amyloidosis, Finnish type) (GSN), transcript variant 3, mRNA. /// Homo sapiens gelsolin (amyloidosis, Finnish type) (GSN), transcript variant 4, mRNA. /// Homo sapiens gelsolin (amyloidosis, Finnish type) (GSN), transcript variant 2, mRNA. /// Homo sapiens gelsolin (amyloidosis, Finnish type) (GSN), transcript variant 5, mRNA. /// Homo sapiens gelsolin (amyloidosis, Finnish type) (GSN), transcript variant 1, mRNA. /// Homo sapiens gelsolin (amyloidosis, Finnish type) (GSN), transcript variant 6, mRNA. /// Homo sapiens gelsolin (amyloidosis, Finnish type) (GSN), transcript variant 7, mRNA.	gelsolin (amyloidosis, Finnish type)	0.00296844	1.229245807

Functional Group 31	Transcript Description	Gene Title	Bayes.lnp	Fold
LEFTY2	Left-right determination factor 2 gene:ENSG00000143768 /// Homo sapiens left-right determination factor 2, mRNA (cDNA clone IMAGE:6140189). /// Homo sapiens left-right determination factor 2 (LEFTY2), mRNA.	left-right determination factor 2	0.000672282	1.236944527
GDF3	Growth/differentiation factor 3 gene:ENSG00000184344 /// Homo sapiens clone DNA35674 GDF3 (UNQ222) mRNA, complete cds. /// Homo sapiens growth differentiation factor 3 (GDF3), mRNA.	growth differentiation factor 3	0.001822871	-1.282528509
GDF15	Growth/differentiation factor 15 gene:ENSG00000130513 /// Homo sapiens macrophage inhibitory cytokine-1 (MIC-1) mRNA, complete cds. /// Homo sapiens growth differentiation factor 15 (GDF15), mRNA.	growth differentiation factor 15	2.68327E-08	-1.724689975
INHBE	Inhibin beta E chain gene:ENSG00000139269 /// Homo sapiens activin beta E subunit mRNA, complete cds. /// Homo sapiens inhibin, beta E (INHBE), mRNA.	inhibin, beta E	0.000911092	1.360080078
LEFTY1	Left-right determination factor 1 gene:ENSG00000143787 /// Homo sapiens TGF-beta type secreted signaling protein LEFTYB mRNA, complete cds. /// Homo sapiens left-right determination factor 1 (LEFTY1), mRNA.	left-right determination factor 1	2.36431E-05	1.379151452

IL24	Interleukin 24 gene:ENSG00000162892 /// Interleukin-24 gene:ENSG00000162892 /// IL-24 splice variant delE5 gene:ENSG00000162892 /// interleukin 24 isoform 2 gene:ENSG00000162892 /// Melanocyte-associated Mda-7 splice variant gene:ENSG00000162892 /// interleukin 24 isoform 2 gene:ENSG00000162892 /// Homo sapiens IL-24 splice variant delE5 mRNA, complete cds, alternatively spliced. /// Homo sapiens interleukin 24 (IL24), transcript variant 1, mRNA. /// Homo sapiens interleukin 24 (IL24), transcript variant 2, mRNA.	interleukin 24	0.000865 021	- 1.370995 239
TFF1	Trefoil factor 1 gene:ENSG00000160182 /// Human pS2 mRNA induced by estrogen from human breast cancer cell line MCF-7. /// Homo sapiens trefoil factor 1 (TFF1), mRNA.	trefoil factor 1	0.000525 437	1.379149 944
**Functional Group 32**	**Transcript Description**	**Gene Title**	**Bayes.ln p**	**Fold**
EXOSC5	Exosome complex exonuclease RRP46 gene:ENSG00000077348 /// Homo sapiens chronic myelogenous leukemia tumor antigen 28 mRNA, complete cds. /// Homo sapiens exosome component 5 (EXOSC5), mRNA.	exosome component 5	0.001652 72	1.181146 779

EXOSC3	Isoform 2 of SH2 domain-containing adapter protein B gene:ENSG00000107338 /// exosome component 3 isoform 2 gene:ENSG00000107371 /// Exosome complex exonuclease RRP40 gene:ENSG00000107371 /// Homo sapiens cDNA FLJ75926 complete cds, highly similar to Homo sapiens exosome component 3 (EXOSC3), transcript variant 1, mRNA. /// Homo sapiens exosome component 3 (EXOSC3), transcript variant 1, mRNA. /// Homo sapiens exosome component 3 (EXOSC3), transcript variant 2, mRNA.	exosome component 3	0.003910206	1.19087611
ALS2CR8	Isoform 1 of Amyotrophic lateral sclerosis 2 chromosomal region candidate gene 8 protein gene:ENSG00000138380 /// Isoform 1 of Amyotrophic lateral sclerosis 2 chromosomal region candidate gene 8 protein gene:ENSG00000138380 /// Homo sapiens amyotrophic lateral sclerosis 2 (juvenile) chromosome region, candidate 8, mRNA (cDNA clone MGC:44837 IMAGE:4521845), complete cds. /// Homo sapiens amyotrophic lateral sclerosis 2 (juvenile) chromosome region, candidate 8 (ALS2CR8), mRNA. /// Homo sapiens amyotrophic lateral sclerosis 2 (juvenile) chromosome region, candidate 8 (ALS2CR8), mRNA.	amyotrophic lateral sclerosis 2 (juvenile) chromosome region, candidate 8	0.005081142	1.390537787
E2F7	Isoform 1 of Transcription factor E2F7 gene:ENSG00000165891 /// Putative uncharacterized protein E2F7 gene:ENSG00000165891 /// Homo sapiens E2F transcription factor 7, mRNA (cDNA clone MGC:167976 IMAGE:9020353), complete cds. /// Homo sapiens E2F transcription factor 7 (E2F7), mRNA.	E2F transcription factor 7	1.68088E-05	-1.315656104

POLR1E	Isoform 2 of DNA-directed RNA polymerase I subunit RPA49 gene:ENSG00000137054 /// Isoform 1 of DNA-directed RNA polymerase I subunit RPA49 gene:ENSG00000137054 /// Homo sapiens polymerase (RNA) I polypeptide E, 53kDa, mRNA (cDNA clone MGC:5609 IMAGE:3461710), complete cds. /// Homo sapiens polymerase (RNA) I polypeptide E, 53kDa (POLR1E), mRNA.	polymerase (RNA) I polypeptide E, 53kDa	0.005571166	1.163774111
BXDC5	14 kDa protein gene:ENSG00000117133 /// Ribosome production factor 1 gene:ENSG00000117133 /// Homo sapiens brix domain containing 5, mRNA (cDNA clone MGC:22954 IMAGE:4853072), complete cds. /// Homo sapiens brix domain containing 5 (BXDC5), mRNA.	brix domain containing 5	0.002256161	1.198776352
RCL1	RNA 3'-terminal phosphate cyclase-like protein gene:ENSG00000120158 /// RNA terminal phosphate cyclase-like 1 gene:ENSG00000120158 /// RNA terminal phosphate cyclase-like 1 gene:ENSG00000120158 /// RNA terminal phosphate cyclase-like 1 gene:ENSG00000120158 /// Homo sapiens RNA terminal phosphate cyclase-like 1, mRNA (cDNA clone MGC:1390 IMAGE:3343468), complete cds. /// Homo sapiens RNA terminal phosphate cyclase-like 1 (RCL1), mRNA.	RNA terminal phosphate cyclase-like 1	0.008619101	-1.149772905
RPS19BP1	40S ribosomal protein S19-binding protein 1 gene:ENSG00000187051 /// Homo sapiens ribosomal protein S19 binding protein 1, mRNA (cDNA clone MGC:45448 IMAGE:5399759), complete cds. /// Homo sapiens ribosomal protein S19 binding protein 1 (RPS19BP1), mRNA.	ribosomal protein S19 binding protein 1	0.00679716	1.16180038
**Functional Group 33**	**Transcript Description**	**Gene Title**	**Bayes.lnp**	**Fold**

DAPP1	Isoform 1 of Dual adapter for phosphotyrosine and 3-phosphotyrosine and 3-phosphoinositide gene:ENSG00000070190 /// Homo sapiens B lymphocyte adapter protein BAM32 (BAM32) mRNA, complete cds. /// Homo sapiens dual adaptor of phosphotyrosine and 3-phosphoinositides (DAPP1), mRNA.	dual adaptor of phosphotyrosine and 3-phosphoinositides	0.002380348	-1.448934867
CDC25C	Isoform 5 of M-phase inducer phosphatase 3 gene:ENSG00000158402 /// Isoform 4 of M-phase inducer phosphatase 3 gene:ENSG00000158402 /// Isoform 2 of M-phase inducer phosphatase 3 gene:ENSG00000158402 /// M-phase inducer phosphatase 3 gene:ENSG00000158402 /// Homo sapiens cDNA FLJ40391 fis, clone TESTI2036833, highly similar to M-PHASE INDUCER PHOSPHATASE 3 (EC 3.1.3.48). /// Homo sapiens cell division cycle 25 homolog C (S. pombe) (CDC25C), transcript variant 1, mRNA. /// Homo sapiens cell division cycle 25 homolog C (S. pombe) (CDC25C), transcript variant 2, mRNA.	cell division cycle 25 homolog C (S. pombe)	0.001950188	1.213706473
PTPRM	Receptor-type tyrosine-protein phosphatase mu gene:ENSG00000173482 /// PTPRM protein gene:ENSG00000173482 /// cDNA FLJ55898, highly similar to Receptor-type tyrosine-protein phosphatase mu gene:ENSG00000173482 /// Homo sapiens protein tyrosine phosphatase, receptor type, M, mRNA (cDNA clone MGC:166994 IMAGE:8860327), complete cds. /// Homo sapiens protein tyrosine phosphatase, receptor type, M (PTPRM), transcript variant 2, mRNA. /// Homo sapiens protein tyrosine phosphatase, receptor type, M (PTPRM), transcript variant 1, mRNA.	protein tyrosine phosphatase, receptor type, M	0.001477125	-1.226370184

DUSP14	Dual specificity protein phosphatase 14 gene:ENSG00000161326 /// Dual specificity protein phosphatase 14 gene:ENSG00000161326 /// Dual specificity protein phosphatase 14 gene:ENSG00000161326 /// Homo sapiens dual specificity phosphatase 14, mRNA (cDNA clone MGC:1861 IMAGE:3533971), complete cds. /// Homo sapiens dual specificity phosphatase 14 (DUSP14), mRNA.	dual specificity phosphatase 14	0.001267682	-1.242660659
PTPRH	Isoform 1 of Receptor-type tyrosine-protein phosphatase H gene:ENSG00000080031 /// Isoform 2 of Receptor-type tyrosine-protein phosphatase H gene:ENSG00000080031 /// Homo sapiens SAP-1 mRNA for protein tyrosine phosphatase precursor, complete cds. /// Homo sapiens protein tyrosine phosphatase, receptor type, H (PTPRH), mRNA.	protein tyrosine phosphatase, receptor type, H	0.005780601	-1.27183179
PPM1D	Protein phosphatase 1D gene:ENSG00000170836 /// Protein phosphatase 1D magnesium-dependent, delta isoform, isoform CRA_b gene:ENSG00000170836 /// Homo sapiens protein phosphatase 1D magnesium-dependent, delta isoform, mRNA (cDNA clone MGC:35090 IMAGE:5167004), complete cds. /// Homo sapiens protein phosphatase 1D magnesium-dependent, delta isoform (PPM1D), mRNA.	protein phosphatase 1D magnesium-dependent, delta isoform	0.000103361	-1.270424375

PTPRE	Receptor-type tyrosine-protein phosphatase epsilon precursor gene:ENSG00000132334 /// Isoform 2 of Receptor-type tyrosine-protein phosphatase epsilon gene:ENSG00000132334 /// Homo sapiens protein tyrosine phosphatase, receptor type, E, mRNA (cDNA clone MGC:48280 IMAGE:5274802), complete cds. /// Homo sapiens protein tyrosine phosphatase, receptor type, E (PTPRE), transcript variant 2, mRNA. /// Homo sapiens protein tyrosine phosphatase, receptor type, E (PTPRE), transcript variant 1, mRNA.	protein tyrosine phosphatase, receptor type, E	3.81593E-06	-1.667249907
DUSP4	dual specificity phosphatase 4 isoform 2 gene:ENSG00000120875 /// dual specificity phosphatase 4 isoform 2 gene:ENSG00000120875 /// Dual specificity protein phosphatase 4 gene:ENSG00000120875 /// Human dual specific protein phosphatase mRNA, complete cds. /// Homo sapiens dual specificity phosphatase 4 (DUSP4), transcript variant 2, mRNA. /// Homo sapiens dual specificity phosphatase 4 (DUSP4), transcript variant 1, mRNA.	dual specificity phosphatase 4	0.002213935	-1.315308582
PDP2	[Pyruvate dehydrogenase [acetyl-transferring]]-phosphatase 2, mitochondrial gene:ENSG00000172840 /// Homo sapiens pyruvate dehydrogenase phosphatase isoenzyme 2, mRNA (cDNA clone MGC:40210 IMAGE:5241881), complete cds. /// Homo sapiens pyruvate dehydrogenase phosphatase isoenzyme 2 (PDP2), mRNA.	pyruvate dehydrogenase phosphatase isoenzyme 2	0.000963476	1.405566356

DUSP1	Dual specificity protein phosphatase 1 gene:ENSG00000120129 /// Homo sapiens dual specificity phosphatase 1, mRNA (cDNA clone MGC:26153 IMAGE:4794895), complete cds. /// Homo sapiens dual specificity phosphatase 1 (DUSP1), mRNA.	dual specificity phosphatase 1	2.09169E-07	-1.624462873
PTPRU	Receptor-type tyrosine-protein phosphatase U gene:ENSG00000060656 /// protein tyrosine phosphatase, receptor type, U isoform 2 precursor gene:ENSG00000060656 /// protein tyrosine phosphatase, receptor type, U isoform 3 precursor gene:ENSG00000060656 /// cDNA FLJ37530 fis, clone BRCAN2012713, highly similar to Receptor-type tyrosine-protein phosphatase U gene:ENSG00000060656 /// FMI protein gene:ENSG00000060656 /// Homo sapiens protein tyrosine phosphatase, receptor type, U, mRNA (cDNA clone MGC:164848 IMAGE:40147934), complete cds. /// Homo sapiens protein tyrosine phosphatase, receptor type, U (PTPRU), transcript variant 3, mRNA. /// Homo sapiens protein tyrosine phosphatase, receptor type, U (PTPRU), transcript variant 2, mRNA. /// Homo sapiens protein tyrosine phosphatase, receptor type, U (PTPRU), transcript variant 1, mRNA.	protein tyrosine phosphatase, receptor type, U	0.004666787	-1.191944005
STYX	Serine/threonine/tyrosine-interacting protein gene:ENSG00000198252 /// Homo sapiens serine/threonine/tyrosine interacting protein, mRNA (cDNA clone MGC:9489 IMAGE:3922829), complete cds. /// Homo sapiens serine/threonine/tyrosine interacting protein (STYX), transcript variant 1, mRNA. /// Homo sapiens serine/threonine/tyrosine interacting protein (STYX), transcript variant 2, mRNA.	serine/threonine/tyrosine interacting protein	4.07395E-05	1.288356906

DUSP6	Isoform 1 of Dual specificity protein phosphatase 6 gene:ENSG00000139318 /// Isoform 2 of Dual specificity protein phosphatase 6 gene:ENSG00000139318 /// Homo sapiens dual specificity phosphatase 6, mRNA (cDNA clone MGC:12852 IMAGE:3954486), complete cds. /// Homo sapiens dual specificity phosphatase 6 (DUSP6), transcript variant 1, mRNA. /// Homo sapiens dual specificity phosphatase 6 (DUSP6), transcript variant 2, mRNA.	dual specificity phosphatase 6	5.9845E-05	1.282981939
DUSP10	Dual specificity protein phosphatase 10 gene:ENSG00000143507 /// cDNA FLJ52863, highly similar to Dual specificity protein phosphatase 10 gene:ENSG00000143507 /// cDNA FLJ52863, highly similar to Dual specificity protein phosphatase 10 gene:ENSG00000143507 /// cDNA FLJ52863, highly similar to Dual specificity protein phosphatase 10 gene:ENSG00000143507 /// Homo sapiens dual specificity phosphatase 10, mRNA (cDNA clone MGC:35356 IMAGE:5182027), complete cds. /// Homo sapiens dual specificity phosphatase 10 (DUSP10), transcript variant 1, mRNA. /// Homo sapiens dual specificity phosphatase 10 (DUSP10), transcript variant 2, mRNA. /// Homo sapiens dual specificity phosphatase 10 (DUSP10), transcript variant 3, mRNA.	dual specificity phosphatase 10	0.004632555	-1.210585825
**Functional Group 34**	**Transcript Description**	**Gene Title**	**Bayes.lnp**	**Fold**

ACE2	Isoform 1 of Angiotensin-converting enzyme 2 gene:ENSG00000130234 /// Homo sapiens ace2 mRNA for angiotensin-converting enzyme 2, complete cds, tissue_type: testis. /// Homo sapiens angiotensin I converting enzyme (peptidyl-dipeptidase A) 2 (ACE2), mRNA.	angiotensin I converting enzyme (peptidyl-dipeptidase A) 2	0.000106005	1.520959887
MME	Neprilysin gene:ENSG00000196549 /// Neprilysin gene:ENSG00000196549 /// Human common acute lymphoblastic leukemia antigen (CALLA) mRNA, complete cds. /// Homo sapiens membrane metallo-endopeptidase (MME), transcript variant 1, mRNA. /// Homo sapiens membrane metallo-endopeptidase (MME), transcript variant 1bis, mRNA. /// Homo sapiens membrane metallo-endopeptidase (MME), transcript variant 2a, mRNA. /// Homo sapiens membrane metallo-endopeptidase (MME), transcript variant 2b, mRNA.	membrane metallo-endopeptidase	0.004025551	-1.204458409
CPXM2	CPXM2 protein gene:ENSG00000121898 /// Carboxypeptidase-like protein X2 gene:ENSG00000121898 /// Homo sapiens clone DNA47394 carboxypeptidase Hlo (UNQ676) mRNA, complete cds. /// Homo sapiens carboxypeptidase X (M14 family), member 2 (CPXM2), mRNA.	carboxypeptidase X (M14 family), member 2	0.001282355	-1.266147419
CPA4	Carboxypeptidase A4 gene:ENSG00000128510 /// Homo sapiens carboxypeptidase A4, mRNA (cDNA clone MGC:59749 IMAGE:6106874), complete cds. /// Homo sapiens carboxypeptidase A4 (CPA4), mRNA.	carboxypeptidase A4	0.004630384	-1.331906934

CPE	Carboxypeptidase E precursor gene:ENSG00000109472 /// Carboxypeptidase E precursor gene:ENSG00000109472 /// Homo sapiens carboxypeptidase E, mRNA (cDNA clone MGC:45357 IMAGE:5182962), complete cds. /// Homo sapiens carboxypeptidase E (CPE), mRNA.	carboxypeptidase E	0.000154258	-1.321171932
CPN1	Carboxypeptidase N catalytic chain gene:ENSG00000120054 /// Homo sapiens carboxypeptidase N, polypeptide 1, mRNA (cDNA clone MGC:34611 IMAGE:5227795), complete cds. /// Homo sapiens carboxypeptidase N, polypeptide 1 (CPN1), mRNA.	carboxypeptidase N, polypeptide 1	0.000944897	1.555618434
MMP14	matrix metalloproteinase 14 preproprotein gene:ENSG00000157227 /// Homo sapiens matrix metallopeptidase 14 (membrane-inserted), mRNA (cDNA clone MGC:64960 IMAGE:6046773), complete cds. /// Homo sapiens matrix metallopeptidase 14 (membrane-inserted) (MMP14), mRNA.	matrix metallopeptidase 14 (membrane-inserted)	0.000150151	-1.237941375
ADAMTS1	A disintegrin and metalloproteinase with thrombospondin motifs 1 gene:ENSG00000154734 /// cDNA FLJ26363 fis, clone HRT05360, highly similar to ADAM-TS 1 gene:ENSG00000154734 /// Homo sapiens ADAM metallopeptidase with thrombospondin type 1 motif, 1, mRNA (cDNA clone MGC:32979 IMAGE:5266513), complete cds. /// Homo sapiens ADAM metallopeptidase with thrombospondin type 1 motif, 1 (ADAMTS1), mRNA.	ADAM metallopeptidase with thrombospondin type 1 motif, 1	0.004789118	-1.220346099
MMP1	Interstitial collagenase gene:ENSG00000196611 /// Human skin collagenase mRNA, complete cds. /// Homo sapiens matrix metallopeptidase 1 (interstitial collagenase) (MMP1), mRNA.	matrix metallopeptidase 1 (interstitial collagenase)	0.008062361	-1.335119543

ADAMTS9	Isoform 1 of A disintegrin and metalloproteinase with thrombospondin motifs 9 gene:ENSG00000163638 /// Homo sapiens ADAM metallopeptidase with thrombospondin type 1 motif, 9, mRNA (cDNA clone MGC:163450 IMAGE:40146609), complete cds. /// Homo sapiens ADAM metallopeptidase with thrombospondin type 1 motif, 9 (ADAMTS9), mRNA.	ADAM metallopeptidase with thrombospondin type 1 motif, 9	0.004115811	1.24865612
MMP10	Stromelysin-2 gene:ENSG00000166670 /// Homo sapiens mRNA for matrix metalloproteinase 10 preproprotein variant, clone: CAS05488. /// Homo sapiens matrix metallopeptidase 10 (stromelysin 2) (MMP10), mRNA.	matrix metallopeptidase 10 (stromelysin 2)	0.009815876	-1.361133759
ADAM12	Isoform 1 of ADAM 12 gene:ENSG00000148848 /// Isoform 4 of ADAM 12 gene:ENSG00000148848 /// Isoform 2 of ADAM 12 gene:ENSG00000148848 /// Homo sapiens meltrin-L precursor (ADAM12) mRNA, complete cds, alternatively spliced. /// Homo sapiens ADAM metallopeptidase domain 12 (ADAM12), transcript variant 1, mRNA. /// Homo sapiens ADAM metallopeptidase domain 12 (ADAM12), transcript variant 2, mRNA.	ADAM metallopeptidase domain 12	1.3884E-07	-1.822030214

MMP2	72 kDa type IV collagenase gene:ENSG00000087245 /// Homo sapiens matrix metallopeptidase 2 (gelatinase A, 72kDa gelatinase, 72kDa type IV collagenase), mRNA (cDNA clone MGC:2313 IMAGE:3161383), complete cds. /// Homo sapiens matrix metallopeptidase 2 (gelatinase A, 72kDa gelatinase, 72kDa type IV collagenase) (MMP2), transcript variant 1, mRNA. /// Homo sapiens matrix metallopeptidase 2 (gelatinase A, 72kDa gelatinase, 72kDa type IV collagenase) (MMP2), transcript variant 2, mRNA.	matrix metallopeptidase 2 (gelatinase A, 72kDa gelatinase, 72kDa type IV collagenase)	0.000312 145	- 1.235343 531
ADAMTS16	Isoform 1 of A disintegrin and metalloproteinase with thrombospondin motifs 16 gene:ENSG00000145536 /// Homo sapiens ADAMTS16s alternative splice form mRNA, complete cds. /// Homo sapiens ADAM metallopeptidase with thrombospondin type 1 motif, 16 (ADAMTS16), mRNA.	ADAM metallopeptidase with thrombospondin type 1 motif, 16	2.90552E -05	- 1.372515 565
OSGEPL1	Isoform 3 of Probable O-sialoglycoprotein endopeptidase 2 gene:ENSG00000128694 /// Homo sapiens O-sialoglycoprotein endopeptidase-like 1, mRNA (cDNA clone MGC:20293 IMAGE:4121450), complete cds. /// Homo sapiens O-sialoglycoprotein endopeptidase-like 1 (OSGEPL1), mRNA.	O-sialoglycoprotein endopeptidase-like 1	0.007468 574	1.235325 034
PAPPA2	Isoform 1 of Pappalysin-2 gene:ENSG00000116183 /// Isoform 2 of Pappalysin-2 gene:ENSG00000116183 /// Homo sapiens cDNA clone IMAGE:40147673. /// Homo sapiens pappalysin 2 (PAPPA2), transcript variant 2, mRNA. /// Homo sapiens pappalysin 2 (PAPPA2), transcript variant 1, mRNA.	pappalysin 2	0.009715 835	- 1.246859 297
OVCH2	Ovochymase-2 gene:ENSG00000183378 /// Homo sapiens ovochymase 2 (OVCH2), mRNA.	ovochymase 2	0.000527 571	- 1.427335 294

RNF43	RING finger protein 43 gene:ENSG00000108375 /// RING finger protein 43 gene:ENSG00000108375 /// Homo sapiens cDNA FLJ77466 complete cds. /// Homo sapiens ring finger protein 43 (RNF43), mRNA.	ring finger protein 43	0.000262242	-1.403402441
**Functional Group 35**	**Transcript Description**	**Gene Title**	**Bayes.lnp**	**Fold**
MDM2	Isoform Mdm2 of E3 ubiquitin-protein ligase Mdm2 gene:ENSG00000135679 /// Putative uncharacterized protein MDM2 gene:ENSG00000135679 /// Isoform Mdm2-C of E3 ubiquitin-protein ligase Mdm2 gene:ENSG00000135679 /// Isoform Mdm2-G of E3 ubiquitin-protein ligase Mdm2 gene:ENSG00000135679 /// Isoform Mdm2-A of E3 ubiquitin-protein ligase Mdm2 gene:ENSG00000135679 /// Isoform Mdm2-A1 of E3 ubiquitin-protein ligase Mdm2 gene:ENSG00000135679 /// p53-binding protein gene:ENSG00000135679 /// mouse double minute 2 homolog isoform MDM2 gene:ENSG00000135679 /// MDM2 variant FB29 gene:ENSG00000135679 /// mouse double minute 2 homolog isoform MDM2a gene:ENSG00000135679 /// Putative uncharacterized protein MDM2 gene:ENSG00000135679 /// Isoform Mdm2-G of E3 ubiquitin-protein ligase Mdm2 gene:ENSG00000135679 /// Isoform Mdm2-C of E3 ubiquitin-protein ligase Mdm2 gene:ENSG00000135679 /// p53-binding protein gene:ENSG00000135679 /// Isoform Mdm2-A of E3 ubiquitin-protein ligase Mdm2 gene:ENSG00000135679 /// Isoform Mdm2-A1 of E3 ubiquitin-protein ligase Mdm2 gene:ENSG00000135679 /// Homo sapiens cDNA FLJ75260 complete cds,	Mdm2 p53 binding protein homolog (mouse)	1.59086E-06	-1.340333826

	highly similar to H.sapiens mRNA for p53-associated gene. /// Homo sapiens Mdm2 p53 binding protein homolog (mouse) (MDM2), transcript variant MDM2, mRNA. /// Homo sapiens Mdm2 p53 binding protein homolog (mouse) (MDM2), transcript variant MDM2a, mRNA. /// Homo sapiens Mdm2 p53 binding protein homolog (mouse) (MDM2), transcript variant MDM2e, mRNA. /// Homo sapiens Mdm2 p53 binding protein homolog (mouse) (MDM2), transcript variant MDM2b, mRNA. /// Homo sapiens Mdm2 p53 binding protein homolog (mouse) (MDM2), transcript variant MDM2d, mRNA.			
LNX1	Isoform 2 of E3 ubiquitin-protein ligase LNX gene:ENSG00000072201 /// Isoform 1 of E3 ubiquitin-protein ligase LNX gene:ENSG00000072201 /// Isoform 1 of E3 ubiquitin-protein ligase LNX gene:ENSG00000072201 /// Homo sapiens multi-PDZ-domain-containing protein mRNA, complete cds. /// Homo sapiens ligand of numb-protein X 1 (LNX1), transcript variant 1, mRNA. /// Homo sapiens ligand of numb-protein X 1 (LNX1), transcript variant 2, mRNA.	ligand of numb-protein X 1	0.001422811	-1.346032277

ASB13	Putative uncharacterized protein ASB13 gene:ENSG00000196372 /// Isoform 1 of Ankyrin repeat and SOCS box protein 13 gene:ENSG00000196372 /// Isoform 2 of Ankyrin repeat and SOCS box protein 13 gene:ENSG00000196372 /// Homo sapiens ankyrin repeat and SOCS box-containing 13, mRNA (cDNA clone MGC:19879 IMAGE:4552643), complete cds. /// Homo sapiens ankyrin repeat and SOCS box-containing 13 (ASB13), mRNA.	ankyrin repeat and SOCS box-containing 13	0.001693865	1.297897898
ANAPC11	cdna:known supercontig::NT_113944:146301:154840:1 gene:ENSG00000215639 /// Isoform 2 of Anaphase-promoting complex subunit 11 gene:ENSG00000141552 /// Isoform 1 of Anaphase-promoting complex subunit 11 gene:ENSG00000141552 /// Isoform 1 of Anaphase-promoting complex subunit 11 gene:ENSG00000141552 /// Isoform 1 of Anaphase-promoting complex subunit 11 gene:ENSG00000141552 /// Homo sapiens APC11 anaphase promoting complex subunit 11, mRNA (cDNA clone MGC:882 IMAGE:3346039), complete cds. /// Homo sapiens anaphase promoting complex subunit 11 (ANAPC11), transcript variant 2, mRNA. /// Homo sapiens anaphase promoting complex subunit 11 (ANAPC11), transcript variant 1, mRNA. /// Homo sapiens anaphase promoting complex subunit 11 (ANAPC11), transcript variant 3, mRNA. /// Homo sapiens anaphase promoting complex subunit 11 (ANAPC11), transcript variant 4, mRNA. /// Homo sapiens anaphase promoting complex subunit 11 (ANAPC11), transcript variant 5, mRNA. /// Homo sapiens anaphase promoting complex subunit 11 (ANAPC11), transcript variant 6, mRNA. /// Homo sapiens anaphase	anaphase promoting complex subunit 11	0.007711672	1.250778357

	promoting complex subunit 11 (ANAPC11), transcript variant 7, mRNA.			

FBXW7	Isoform 4 of F-box/WD repeat-containing protein 7 gene:ENSG00000109670 /// Isoform 1 of F-box/WD repeat-containing protein 7 gene:ENSG00000109670 /// Isoform 2 of F-box/WD repeat-containing protein 7 gene:ENSG00000109670 /// Isoform 1 of F-box/WD repeat-containing protein 7 gene:ENSG00000109670 /// Homo sapiens archipelago alpha form mRNA, complete cds. /// Homo sapiens F-box and WD repeat domain containing 7 (FBXW7), transcript variant 1, mRNA. /// Homo sapiens F-box and WD repeat domain containing 7 (FBXW7), transcript variant 3, mRNA. /// Homo sapiens F-box and WD repeat domain containing 7 (FBXW7), transcript variant 2, mRNA.	F-box and WD repeat domain containing 7	0.000408298	-1.278626563
CDC20	Cell division cycle protein 20 homolog gene:ENSG00000117399 /// Cell division cycle protein 20 homolog gene:ENSG00000117399 /// Homo sapiens cDNA FLJ51449 complete cds, highly similar to Cell division cycle protein 20 homolog. /// Homo sapiens cell division cycle 20 homolog (S. cerevisiae) (CDC20), mRNA.	cell division cycle 20 homolog (S. cerevisiae)	0.006804429	1.15626757
SENP1	Isoform 2 of Sentrin-specific protease 1 gene:ENSG00000079387 /// Putative uncharacterized protein DKFZp686L1432 gene:ENSG00000079387 /// Homo sapiens SUMO1/sentrin specific peptidase 1, mRNA (cDNA clone MGC:41833 IMAGE:5298667), complete cds. /// Homo sapiens SUMO1/sentrin specific peptidase 1 (SENP1), mRNA.	SUMO1/sentrin specific peptidase 1	0.004842029	1.195776698

KLHL9	Kelch-like protein 9 gene:ENSG00000198642 /// Homo sapiens kelch-like 9 (Drosophila), mRNA (cDNA clone MGC:16975 IMAGE:4339111), complete cds. /// Homo sapiens kelch-like 9 (Drosophila) (KLHL9), mRNA.	kelch-like 9 (Drosophila)	0.009409303	1.164031936
USP44	Ubiquitin carboxyl-terminal hydrolase 44 gene:ENSG00000136014 /// Ubiquitin carboxyl-terminal hydrolase 44 gene:ENSG00000136014 /// Homo sapiens ubiquitin specific peptidase 44, mRNA (cDNA clone MGC:26981 IMAGE:4825887), complete cds. /// Homo sapiens ubiquitin specific peptidase 44 (USP44), transcript variant 1, mRNA. /// Homo sapiens ubiquitin specific peptidase 44 (USP44), transcript variant 2, mRNA.	ubiquitin specific peptidase 44	0.006599241	1.160784088
DUB4	Ubiquitin carboxyl-terminal hydrolase 17-like protein 6 gene:ENSG00000205946 /// Homo sapiens deubiquitinating enzyme DUB4 mRNA, complete cds.	deubiquitinating enzyme DUB4	0.000571368	-1.350321293
RAD23A	UV excision repair protein RAD23 homolog A gene:ENSG00000179262 /// Homo sapiens RAD23 homolog A (S. cerevisiae), mRNA (cDNA clone MGC:111083 IMAGE:30389208), complete cds. /// Homo sapiens RAD23 homolog A (S. cerevisiae) (RAD23A), mRNA.	RAD23 homolog A (S. cerevisiae)	6.02241E-05	1.337419968
DZIP3	Isoform 2 of E3 ubiquitin-protein ligase DZIP3 gene:ENSG00000198919 /// Isoform 1 of E3 ubiquitin-protein ligase DZIP3 gene:ENSG00000198919 /// Homo sapiens DAZ interacting protein 3, zinc finger, mRNA (cDNA clone MGC:75477 IMAGE:30387514), complete cds. /// Homo sapiens DAZ interacting protein 3, zinc finger (DZIP3), mRNA.	DAZ interacting protein 3, zinc finger	0.000680286	-1.196710337

FBXO9	Isoform 3 of F-box only protein 9 gene:ENSG00000112146 /// Isoform 2 of F-box only protein 9 gene:ENSG00000112146 /// Isoform 1 of F-box only protein 9 gene:ENSG00000112146 /// Homo sapiens cross-immune reaction antigen (VCIA1) mRNA, complete cds. /// Homo sapiens F-box protein 9 (FBXO9), transcript variant 1, mRNA. /// Homo sapiens F-box protein 9 (FBXO9), transcript variant 2, mRNA. /// Homo sapiens F-box protein 9 (FBXO9), transcript variant 3, mRNA.	F-box protein 9	0.004685052	1.19355982
RNF144B	Putative uncharacterized protein RNF144B (Fragment) gene:ENSG00000137393 /// E3 ubiquitin-protein ligase RNF144B gene:ENSG00000137393 /// Homo sapiens p53RFP mRNA for p53-inducible RING finger protein, complete cds. /// Homo sapiens ring finger 144B (RNF144B), mRNA.	ring finger protein 144B	0.001492364	-1.232445743
TTLL12	Tubulin--tyrosine ligase-like protein 12 gene:ENSG00000100304 /// Putative uncharacterized protein gene:ENSG00000100304 /// Tubulin tyrosine ligase-like family, member 12 gene:ENSG00000100304 /// Homo sapiens tubulin tyrosine ligase-like family, member 12, mRNA (cDNA clone MGC:2635 IMAGE:3504490), complete cds. /// Homo sapiens tubulin tyrosine ligase-like family, member 12 (TTLL12), mRNA.	tubulin tyrosine ligase-like family, member 12	0.004687885	1.159580407
UBE2T	Ubiquitin-conjugating enzyme E2 T gene:ENSG00000077152 /// Homo sapiens ubiquitin-conjugating enzyme E2 mRNA, complete cds. /// Homo sapiens ubiquitin-conjugating enzyme E2T (putative) (UBE2T), mRNA.	ubiquitin-conjugating enzyme E2T (putative)	0.007508601	1.17669488

RWDD4A	RWD domain-containing protein 4A gene:ENSG00000182552 /// Homo sapiens RWD domain containing 4A, mRNA (cDNA clone MGC:10198 IMAGE:3909581), complete cds. /// Homo sapiens RWD domain containing 4A (RWDD4A), mRNA.	RWD domain containing 4A	0.01070688	1.18168903
UBE2J1	Ubiquitin-conjugating enzyme E2 J1 gene:ENSG00000198833 /// Homo sapiens mRNA full length insert cDNA clone EUROIMAGE 1658099. /// Homo sapiens ubiquitin-conjugating enzyme E2, J1 (UBC6 homolog, yeast) (UBE2J1), mRNA.	ubiquitin-conjugating enzyme E2, J1 (UBC6 homolog, yeast)	9.10548E-05	1.323235774
UBE2W	Putative uncharacterized protein UBE2W (Fragment) gene:ENSG00000104343 /// Isoform 2 of Probable ubiquitin-conjugating enzyme E2 W gene:ENSG00000104343 /// Isoform 1 of Probable ubiquitin-conjugating enzyme E2 W gene:ENSG00000104343 /// Homo sapiens ubiquitin-conjugating enzyme 16 mRNA, complete cds. /// Homo sapiens ubiquitin-conjugating enzyme E2W (putative) (UBE2W), transcript variant 1, mRNA. /// Homo sapiens ubiquitin-conjugating enzyme E2W (putative) (UBE2W), transcript variant 2, mRNA.	ubiquitin-conjugating enzyme E2W (putative)	0.001379194	-1.173523405
FBXL7	F-box/LRR-repeat protein 7 gene:ENSG00000183580 /// Homo sapiens F-box and leucine-rich repeat protein 7, mRNA (cDNA clone MGC:104130 IMAGE:30915585), complete cds. /// Homo sapiens F-box and leucine-rich repeat protein 7 (FBXL7), mRNA.	F-box and leucine-rich repeat protein 7	0.004678404	-1.203793005
HERC5	hect domain and RLD 5 gene:ENSG00000138646 /// Homo sapiens HECT E3 ubiquitin ligase (HERC5) mRNA, complete cds. /// Homo sapiens hect domain and RLD 5 (HERC5), mRNA.	hect domain and RLD 5	2.58381E-05	-1.285115863

UBE2E1	Ubiquitin-conjugating enzyme E2 E1 gene:ENSG00000170142 /// ubiquitin-conjugating enzyme E2E 1 isoform 2 gene:ENSG00000170142 /// Homo sapiens ubiquitin-conjugating enzyme E2E 1 (UBC4/5 homolog, yeast), mRNA (cDNA clone MGC:9268 IMAGE:3853408), complete cds. /// Homo sapiens ubiquitin-conjugating enzyme E2E 1 (UBC4/5 homolog, yeast) (UBE2E1), transcript variant 2, mRNA. /// Homo sapiens ubiquitin-conjugating enzyme E2E 1 (UBC4/5 homolog, yeast) (UBE2E1), transcript variant 1, mRNA.	ubiquitin-conjugating enzyme E2E 1 (UBC4/5 homolog, yeast)	0.004134 323	1.192105 972
HLCS	Biotin--protein ligase gene:ENSG00000159267 /// Biotin--protein ligase gene:ENSG00000159267 /// Homo sapiens mRNA for HCS, complete cds. /// Homo sapiens holocarboxylase synthetase (biotin-(proprionyl-Coenzyme A-carboxylase (ATP-hydrolysing)) ligase) (HLCS), mRNA.	holocarboxylase synthetase (biotin-(proprionyl-Coenzyme A-carboxylase (ATP-hydrolysing)) ligase)	0.000262 767	- 1.263342 262
ATG4A	27 kDa protein gene:ENSG00000101844 /// Isoform 1 of Cysteine protease ATG4A gene:ENSG00000101844 /// Isoform 3 of Cysteine protease ATG4A gene:ENSG00000101844 /// Isoform 2 of Cysteine protease ATG4A gene:ENSG00000101844 /// 42 kDa protein gene:ENSG00000101844 /// 49 kDa protein gene:ENSG00000101844 /// Homo sapiens ATG4 autophagy related 4 homolog A (S. cerevisiae), mRNA (cDNA clone MGC:62085 IMAGE:4594225), complete cds. /// Homo sapiens ATG4 autophagy related 4 homolog A (S. cerevisiae) (ATG4A), transcript variant 2, mRNA. /// Homo sapiens ATG4 autophagy related 4 homolog A (S. cerevisiae) (ATG4A), transcript variant 1, mRNA.	ATG4 autophagy related 4 homolog A (S. cerevisiae)	1.66894E -06	- 1.416409 983

ATG7	Isoform 1 of Autophagy-related protein 7 gene:ENSG00000197548 /// Isoform 2 of Autophagy-related protein 7 gene:ENSG00000197548 /// Homo sapiens E1-like protein mRNA, complete cds. /// Homo sapiens ATG7 autophagy related 7 homolog (S. cerevisiae) (ATG7), mRNA. /// Homo sapiens ATG7 autophagy related 7 homolog (S. cerevisiae) (ATG7), mRNA.	ATG7 autophagy related 7 homolog (S. cerevisiae)	0.002426188	-1.208929496
TNFAIP3	Tumor necrosis factor, alpha-induced protein 3 gene:ENSG00000118503 /// Human tumor necrosis factor alpha inducible protein A20 mRNA, complete cds. /// Homo sapiens tumor necrosis factor, alpha-induced protein 3 (TNFAIP3), mRNA.	tumor necrosis factor, alpha-induced protein 3	0.001615021	-1.356807131
UFC1	Ufm1-conjugating enzyme 1 gene:ENSG00000143222 /// Homo sapiens ubiquitin-fold modifier conjugating enzyme 1, mRNA (cDNA clone MGC:12182 IMAGE:3961569), complete cds. /// Homo sapiens ubiquitin-fold modifier conjugating enzyme 1 (UFC1), mRNA.	ubiquitin-fold modifier conjugating enzyme 1	0.00316085	1.189479012
FBXL4	F-box/LRR-repeat protein 4 gene:ENSG00000112234 /// F-box/LRR-repeat protein 4 gene:ENSG00000112234 /// Homo sapiens F-box and leucine-rich repeat protein 4, mRNA (cDNA clone MGC:111014 IMAGE:6178874), complete cds. /// Homo sapiens F-box and leucine-rich repeat protein 4 (FBXL4), mRNA.	F-box and leucine-rich repeat protein 4	0.008299585	1.20773264
RNF26	RING finger protein 26 gene:ENSG00000173456 /// Homo sapiens ring finger protein 26, mRNA (cDNA clone MGC:2642 IMAGE:3507662), complete cds. /// Homo sapiens ring finger protein 26 (RNF26), mRNA.	ring finger protein 26	0.002465445	1.192581632

FANCL	Putative uncharacterized protein FANCL gene:ENSG00000115392 /// E3 ubiquitin-protein ligase FANCL gene:ENSG00000115392 /// Fanconi anemia, complementation group L isoform 1 gene:ENSG00000115392 /// Putative uncharacterized protein FANCL gene:ENSG00000115392 /// Homo sapiens Fanconi anemia, complementation group L, mRNA (cDNA clone MGC:60295 IMAGE:5180184), complete cds. /// Homo sapiens Fanconi anemia, complementation group L (FANCL), transcript variant 2, mRNA. /// Homo sapiens Fanconi anemia, complementation group L (FANCL), transcript variant 1, mRNA.	Fanconi anemia, complementation group L	0.00634169	1.239930586
RNF138	Isoform 1 of E3 ubiquitin-protein ligase RNF138 gene:ENSG00000134758 /// Isoform 2 of E3 ubiquitin-protein ligase RNF138 gene:ENSG00000134758 /// Isoform 1 of E3 ubiquitin-protein ligase RNF138 gene:ENSG00000134758 /// Homo sapiens ring finger protein 138, mRNA (cDNA clone MGC:8758 IMAGE:3914953), complete cds. /// Homo sapiens ring finger protein 138 (RNF138), transcript variant 1, mRNA. /// Homo sapiens ring finger protein 138 (RNF138), transcript variant 2, mRNA.	ring finger protein 138	0.005227981	1.167172517
UFM1	Ubiquitin-fold modifier 1 gene:ENSG00000120686 /// Ubiquitin-fold modifier 1 gene:ENSG00000120686 /// Ubiquitin-fold modifier 1 gene:ENSG00000120686 /// Homo sapiens ubiquitin-fold modifier 1, mRNA (cDNA clone MGC:12198 IMAGE:3829206), complete cds. /// Homo sapiens ubiquitin-fold modifier 1 (UFM1), mRNA.	ubiquitin-fold modifier 1	2.28199E-05	-1.317559431

UBC	ubiquitin C gene:ENSG00000150991 /// 44 kDa protein gene:ENSG00000150991 /// Ubiquitin C splice variant gene:ENSG00000150991 /// 44 kDa protein gene:ENSG00000150991 /// Ubiquitin C gene:ENSG00000150991 /// 44 kDa protein gene:ENSG00000150991 /// Homo sapiens mRNA for polyubiquitin UbC, complete cds. /// Homo sapiens ubiquitin C (UBC), mRNA.	ubiquitin C	0.007119941	-1.112166335
RNF43	RING finger protein 43 gene:ENSG00000108375 /// RING finger protein 43 gene:ENSG00000108375 /// Homo sapiens cDNA FLJ77466 complete cds. /// Homo sapiens ring finger protein 43 (RNF43), mRNA.	ring finger protein 43	0.000262242	-1.403402441
**Functional Group 36**	**Transcript Description**	**Gene Title**	**Bayes.lnp**	**Fold**
AP1G2	AP-1 complex subunit gamma-like 2 gene:ENSG00000213983 /// AP-1 complex subunit gamma-like 2 gene:ENSG00000213983 /// Homo sapiens adaptor-related protein complex 1, gamma 2 subunit, mRNA (cDNA clone MGC:60125 IMAGE:5589077), complete cds. /// Homo sapiens adaptor-related protein complex 1, gamma 2 subunit (AP1G2), mRNA.	adaptor-related protein complex 1, gamma 2 subunit	0.006144535	1.191765245
AP3M1	AP-3 complex subunit mu-1 gene:ENSG00000185009 /// AP-3 complex subunit mu-1 gene:ENSG00000185009 /// Homo sapiens adaptor-related protein complex 3, mu 1 subunit, mRNA (cDNA clone MGC:75195 IMAGE:6169822), complete cds. /// Homo sapiens adaptor-related protein complex 3, mu 1 subunit (AP3M1), transcript variant 1, mRNA. /// Homo sapiens adaptor-related protein complex 3, mu 1 subunit (AP3M1), transcript variant 2, mRNA.	adaptor-related protein complex 3, mu 1 subunit	0.001357981	1.181431132

GPRC5A	Retinoic acid-induced protein 3 gene:ENSG00000013588 /// Homo sapiens cDNA FLJ16117 fis, clone ASTRO2003632, highly similar to Retinoic acid-induced protein 3 (G-proteincoupled receptor family C group 5 member A). /// Homo sapiens G protein-coupled receptor, family C, group 5, member A (GPRC5A), mRNA.	G protein-coupled receptor, family C, group 5, member A	4.48256E-06	-1.572536281
STXBP5	Isoform 1 of Syntaxin-binding protein 5 gene:ENSG00000164506 /// Isoform 2 of Syntaxin-binding protein 5 gene:ENSG00000164506 /// Isoform 3 of Syntaxin-binding protein 5 gene:ENSG00000164506 /// Syntaxin binding protein 5 gene:ENSG00000164506 /// Syntaxin binding protein 5 gene:ENSG00000164506 /// Isoform 2 of Syntaxin-binding protein 5 gene:ENSG00000164506 /// Isoform 1 of Syntaxin-binding protein 5 gene:ENSG00000164506 /// Isoform 3 of Syntaxin-binding protein 5 gene:ENSG00000164506 /// Homo sapiens mRNA; cDNA DKFZp434L0335 (from clone DKFZp434L0335); complete cds. /// Homo sapiens syntaxin binding protein 5 (tomosyn) (STXBP5), transcript variant 1, mRNA. /// Homo sapiens syntaxin binding protein 5 (tomosyn) (STXBP5), transcript variant 2, mRNA.	syntaxin binding protein 5 (tomosyn)	0.004403472	-1.193162511
SNAPIN	SNARE-associated protein Snapin gene:ENSG00000143553 /// Homo sapiens SNAP-associated protein, mRNA (cDNA clone MGC:11087 IMAGE:3830132), complete cds. /// Homo sapiens SNAP-associated protein (SNAPIN), mRNA.	SNAP-associated protein	0.003472831	1.243222292

NECAP1	Isoform 1 of Adaptin ear-binding coat-associated protein 1 gene:ENSG00000089818 /// Homo sapiens NECAP endocytosis associated 1, mRNA (cDNA clone IMAGE:3940703), complete cds. /// Homo sapiens NECAP endocytosis associated 1 (NECAP1), transcript variant 1, mRNA. /// Homo sapiens NECAP endocytosis associated 1 (NECAP1), transcript variant 2, transcribed RNA.	NECAP endocytosis associated 1	0.009131076	1.17049267
**Functional Group 37**	**Transcript Description**	**Gene Title**	**Bayes.lnp**	**Fold**
CCL3	C-C motif chemokine 3 gene:ENSG00000006075 /// Human macrophage inflammatory protein (G0S19-1) mRNA, complete cds. /// Homo sapiens chemokine (C-C motif) ligand 3 (CCL3), mRNA.	chemokine (C-C motif) ligand 3	0.008993586	-1.26777808
IL8	11 kDa protein gene:ENSG00000169429 /// Isoform 1 of Interleukin-8 gene:ENSG00000169429 /// 15 kDa protein gene:ENSG00000169429 /// Human beta-thromboglobulin-like protein mRNA, complete cds. /// Homo sapiens interleukin 8 (IL8), mRNA.	interleukin 8	1.27948E-05	-1.638323447
CXCL1	Growth-regulated alpha protein gene:ENSG00000163739 /// Homo sapiens chemokine (C-X-C motif) ligand 1 (melanoma growth stimulating activity, alpha), mRNA (cDNA clone MGC:9049 IMAGE:3856841), complete cds. /// Homo sapiens chemokine (C-X-C motif) ligand 1 (melanoma growth stimulating activity, alpha) (CXCL1), mRNA.	chemokine (C-X-C motif) ligand 1 (melanoma growth stimulating activity, alpha)	0.00095477	-1.316125837

CXCL14	small inducible cytokine B14 precursor gene:ENSG00000145824 /// Homo sapiens clone DNA39523 SCYB14 (UNQ240) mRNA, complete cds. /// Homo sapiens chemokine (C-X-C motif) ligand 14 (CXCL14), mRNA.	chemokine (C-X-C motif) ligand 14	0.00146112	1.254131499
CXCL5	C-X-C motif chemokine 5 gene:ENSG00000163735 /// 17 kDa protein gene:ENSG00000163735 /// Homo sapiens chemokine (C-X-C motif) ligand 5, mRNA (cDNA clone MGC:12304 IMAGE:3826939), complete cds. /// Homo sapiens chemokine (C-X-C motif) ligand 5 (CXCL5), mRNA.	chemokine (C-X-C motif) ligand 5	0.003204862	1.212422535
**Functional Group 38**	**Transcript Description**	**Gene Title**	**Bayes.lnp**	**Fold**
HOXD1	Homeobox protein Hox-D1 gene:ENSG00000128645 /// HOXD1 protein (Fragment) gene:ENSG00000128645 /// cdna:known chromosome:NCBI36:2:176762257:176763880:1 gene:ENSG00000128645 /// Homo sapiens homeobox-containing transcripton factor HOXD1 (HOXD1) mRNA, complete cds. /// Homo sapiens homeobox D1 (HOXD1), mRNA.	homeobox D1	0.006838952	-1.24025445
HMX2	Homeobox protein HMX2 gene:ENSG00000188816 /// Homo sapiens H6 family homeobox 2, mRNA (cDNA clone MGC:164389 IMAGE:40146780), complete cds. /// Homo sapiens H6 family homeobox 2 (HMX2), mRNA.	H6 family homeobox 2	0.007097856	-1.292386395

PRRX1	Isoform PMX1-B of Paired mesoderm homeobox protein 1 gene:ENSG00000116132 /// Isoform PMX1-A of Paired mesoderm homeobox protein 1 gene:ENSG00000116132 /// Homo sapiens mRNA for paired mesoderm homeobox 1 isoform pmx-1b variant, clone: FCC113D08. /// Homo sapiens paired related homeobox 1 (PRRX1), transcript variant pmx-1b, mRNA. /// Homo sapiens paired related homeobox 1 (PRRX1), transcript variant pmx-1a, mRNA.	paired related homeobox 1	0.001019017	-1.297168695
GBX2	Homeobox protein GBX-2 gene:ENSG00000168505 /// Homo sapiens gastrulation brain homeobox 2, mRNA (cDNA clone MGC:169071 IMAGE:9021448), complete cds. /// Homo sapiens gastrulation brain homeobox 2 (GBX2), mRNA.	gastrulation brain homeobox 2	0.002958203	-1.262055554
OTX2	Isoform 2 of Homeobox protein OTX2 gene:ENSG00000165588 /// cdna:known chromosome:NCBI36:14:56338206:56341927:-1 gene:ENSG00000165588 /// Homo sapiens orthodenticle homeobox 2, mRNA (cDNA clone MGC:45000 IMAGE:5493541), complete cds. /// Homo sapiens orthodenticle homeobox 2 (OTX2), transcript variant 1, mRNA. /// Homo sapiens orthodenticle homeobox 2 (OTX2), transcript variant 2, mRNA.	orthodenticle homeobox 2	0.000136851	1.300210245
LASS6	LASS6 protein gene:ENSG00000172292 /// LAG1 longevity assurance homolog 6 gene:ENSG00000172292 /// Homo sapiens LAG1 homolog, ceramide synthase 6, mRNA (cDNA clone MGC:129949 IMAGE:40031838), complete cds. /// Homo sapiens LAG1 homolog, ceramide synthase 6 (LASS6), mRNA.	LAG1 homolog, ceramide synthase 6	0.00473864	-1.155431587

KHDRBS1	Isoform 3 of KH domain-containing, RNA-binding, signal transduction-associated protein 1 gene:ENSG00000121774 /// Isoform 1 of KH domain-containing, RNA-binding, signal transduction-associated protein 1 gene:ENSG00000121774 /// cDNA FLJ54590, highly similar to KH domain-containing, RNA-binding, signaltransduction-associated protein 1 gene:ENSG00000121774 /// Homo sapiens cDNA FLJ34027 fis, clone FCBBF2003549, highly similar to GAP-associated tyrosine phosphoprotein p62 (Sam68). /// Homo sapiens KH domain containing, RNA binding, signal transduction associated 1 (KHDRBS1), mRNA.	KH domain containing, RNA binding, signal transduction associated 1	0.001221063	1.178077995
ZNF578	cdna:known chromosome:NCBI36:19:57648641:57711943:1 gene:ENSG00000221895 gene:ENSG00000167555 /// Homo sapiens cDNA FLJ54871 complete cds, moderately similar to Zinc finger protein 611. /// Homo sapiens zinc finger protein 578 (ZNF578), mRNA.	zinc finger protein 578	0.001014207	-1.420350247
NARG1	Isoform 1 of NMDA receptor-regulated protein 1 gene:ENSG00000164134 /// Isoform 2 of NMDA receptor-regulated protein 1 gene:ENSG00000164134 /// Homo sapiens gastric cancer antigen Ga19 mRNA, complete cds. /// Homo sapiens NMDA receptor regulated 1 (NARG1), mRNA.	NMDA receptor regulated 1	0.008834882	1.149497243

AES	amino-terminal enhancer of split isoform a gene:ENSG00000104964 /// Amino-terminal enhancer of split gene:ENSG00000104964 /// Homo sapiens cDNA FLJ37272 fis, clone BRAMY2012091, highly similar to Homo sapiens amino-terminal enhancer of split (AES), transcript variant 1, mRNA. /// Homo sapiens amino-terminal enhancer of split (AES), transcript variant 2, mRNA. /// Homo sapiens amino-terminal enhancer of split (AES), transcript variant 1, mRNA. /// Homo sapiens amino-terminal enhancer of split (AES), transcript variant 3, mRNA.	amino-terminal enhancer of split	0.009996952	1.133735528
EPAS1	Endothelial PAS domain-containing protein 1 gene:ENSG00000116016 /// Homo sapiens endothelial PAS domain protein 1, mRNA (cDNA clone MGC:59860 IMAGE:6305604), complete cds. /// Homo sapiens endothelial PAS domain protein 1 (EPAS1), mRNA.	endothelial PAS domain protein 1	1.99014E-09	-1.802249992
UTF1	Undifferentiated embryonic cell transcription factor 1 gene:ENSG00000171794 /// Homo sapiens mRNA for UTF1, complete cds. /// Homo sapiens undifferentiated embryonic cell transcription factor 1 (UTF1), mRNA.	undifferentiated embryonic cell transcription factor 1	0.006904401	-1.173181823
TCF7L1	Transcription factor 7-like 1 gene:ENSG00000152284 /// Homo sapiens mRNA for HMG-box transcription factor TCF-3, complete cds. /// Homo sapiens transcription factor 7-like 1 (T-cell specific, HMG-box) (TCF7L1), mRNA.	transcription factor 7-like 1 (T-cell specific, HMG-box)	3.57799E-06	1.363561661
PLAGL2	Zinc finger protein PLAGL2 gene:ENSG00000126003 /// Homo sapiens zinc finger protein PLAGL2 (PLAGL2) mRNA, complete cds. /// Homo sapiens pleiomorphic adenoma gene-like 2 (PLAGL2), mRNA.	pleiomorphic adenoma gene-like 2	0.009657829	1.170076945

MED14	Mediator of RNA polymerase II transcription subunit 14 gene:ENSG00000180182 /// Homo sapiens vitamin D receptor-interacting protein complex component DRIP150 (DRIP150) mRNA, complete cds. /// Homo sapiens mediator complex subunit 14 (MED14), mRNA.	mediator complex subunit 14	0.005748327	1.173182204
FOSL1	Fos-related antigen 1 gene:ENSG00000175592 /// Homo sapiens FOS-like antigen 1, mRNA (cDNA clone MGC:9264 IMAGE:3925241), complete cds. /// Homo sapiens FOS-like antigen 1 (FOSL1), mRNA.	FOS-like antigen 1	0.004718702	-1.166432491
NR5A2	Isoform 2 of Nuclear receptor subfamily 5 group A member 2 gene:ENSG00000116833 /// Isoform 1 of Nuclear receptor subfamily 5 group A member 2 gene:ENSG00000116833 /// Alpha1-fetoprotein transcription factor short variant gene:ENSG00000116833 /// Nuclear receptor NR5A2 gene:ENSG00000116833 /// Homo sapiens mRNA for FTZ-F1 related protein, complete cds. /// Homo sapiens nuclear receptor subfamily 5, group A, member 2 (NR5A2), transcript variant 2, mRNA. /// Homo sapiens nuclear receptor subfamily 5, group A, member 2 (NR5A2), transcript variant 1, mRNA.	nuclear receptor subfamily 5, group A, member 2	1.32405E-05	-1.412950191
VPS36	Isoform 1 of Vacuolar protein-sorting-associated protein 36 gene:ENSG00000136100 /// Homo sapiens vacuolar protein sorting 36 homolog (S. cerevisiae), mRNA (cDNA clone MGC:33029 IMAGE:4814999), complete cds. /// Homo sapiens vacuolar protein sorting 36 homolog (S. cerevisiae) (VPS36), mRNA.	vacuolar protein sorting 36 homolog (S. cerevisiae)	0.000374871	1.276504481

MMS19	cDNA FLJ55586, highly similar to MMS19-like protein gene:ENSG00000155229 /// Putative uncharacterized protein gene:ENSG00000155229 /// Isoform 2 of MMS19 nucleotide excision repair protein homolog gene:ENSG00000155229 /// cDNA FLJ55586, highly similar to MMS19-like protein gene:ENSG00000155229 /// Isoform 5 of MMS19 nucleotide excision repair protein homolog gene:ENSG00000155229 /// Homo sapiens MMS19 nucleotide excision repair homolog (S. cerevisiae), mRNA (cDNA clone MGC:150738 IMAGE:40125680), complete cds. /// Homo sapiens MMS19 nucleotide excision repair homolog (S. cerevisiae) (MMS19), mRNA.	MMS19 nucleotide excision repair homolog (S. cerevisiae)	0.00818753	1.14010785
KLF5	Krueppel-like factor 5 gene:ENSG00000102554 /// Homo sapiens colon Kruppel-like factor (CKLF) mRNA, complete cds. /// Homo sapiens Kruppel-like factor 5 (intestinal) (KLF5), mRNA.	Kruppel-like factor 5 (intestinal)	0.000341289	-1.302502438
CITED2	Isoform 1 of Cbp/p300-interacting transactivator 2 gene:ENSG00000164442 /// Isoform 2 of Cbp/p300-interacting transactivator 2 gene:ENSG00000164442 /// Homo sapiens Cbp/p300-interacting transactivator, with Glu/Asp-rich carboxy-terminal domain, 2, mRNA (cDNA clone MGC:10574 IMAGE:3640855), complete cds. /// Homo sapiens Cbp/p300-interacting transactivator, with Glu/Asp-rich carboxy-terminal domain, 2 (CITED2), mRNA.	Cbp/p300-interacting transactivator, with Glu/Asp-rich carboxy-terminal domain, 2	0.000282325	-1.236838276

ZNF287	zinc finger protein 287 gene:ENSG00000141040 /// zinc finger protein 287 gene:ENSG00000141040 /// zinc finger protein 287 gene:ENSG00000141040 /// Homo sapiens zinc finger protein ZNF287 (ZNF287) mRNA, complete cds. /// Homo sapiens zinc finger protein 287 (ZNF287), mRNA.	zinc finger protein 287	0.003039452	1.437733624
EBF2	cdna:known chromosome:NCBI36:8:25758042:25958292:-1 gene:ENSG00000221818 /// Homo sapiens early B-cell factor 2 (EBF2) mRNA, complete cds, alternatively spliced. /// Homo sapiens early B-cell factor 2 (EBF2), mRNA.	early B-cell factor 2	0.004011726	1.377185453
FOXO4	Isoform 1 of Forkhead box protein O4 gene:ENSG00000184481 /// Isoform 1 of Forkhead box protein O4 gene:ENSG00000184481 /// Isoform Zeta of Forkhead box protein O4 gene:ENSG00000184481 /// Isoform Zeta of Forkhead box protein O4 gene:ENSG00000184481 /// Homo sapiens forkhead box O4, mRNA (cDNA clone MGC:120490 IMAGE:40025381), complete cds. /// Homo sapiens forkhead box O4 (FOXO4), mRNA.	forkhead box O4	0.005084212	1.183502061
CA13	Carbonic anhydrase 13 gene:ENSG00000185015 /// Homo sapiens carbonic anhydrase XIII, mRNA (cDNA clone MGC:59868 IMAGE:6083105), complete cds. /// Homo sapiens carbonic anhydrase XIII (CA13), mRNA.	carbonic anhydrase XIII	0.002780801	-1.337627917
ZNF350	Zinc finger protein 350 gene:ENSG00000171032 /// Homo sapiens KRAB zinc finger protein ZFQR mRNA, complete cds. /// Homo sapiens zinc finger protein 350 (ZNF350), mRNA.	zinc finger protein 350	0.000321715	-1.298051552

EID1	Isoform 1 of EP300-interacting inhibitor of differentiation 1 gene:ENSG00000178558 /// Homo sapiens Rb- and p300-binding protein EID-1 mRNA, complete cds. /// Homo sapiens EP300 interacting inhibitor of differentiation 1 (EID1), mRNA.	EP300 interacting inhibitor of differentiation 1	0.000375162	1.223215966
MDM4	Isoform 1 of Protein Mdm4 gene:ENSG00000198625 /// Mdm4 p53 binding protein homolog gene:ENSG00000198625 /// MDM4 protein gene:ENSG00000198625 /// Double minute 4 variant gene:ENSG00000198625 /// Mdm4 p53 binding protein homolog gene:ENSG00000198625 /// Mdm4 p53 binding protein homolog gene:ENSG00000198625 /// Homo sapiens MDM2-like p53-binding protein (MDMX) mRNA, complete cds. /// Homo sapiens Mdm4 p53 binding protein homolog (mouse) (MDM4), transcript variant 1, mRNA. /// Homo sapiens Mdm4 p53 binding protein homolog (mouse) (MDM4), transcript variant 2, transcribed RNA.	Mdm4 p53 binding protein homolog (mouse)	0.0008988	-1.253372911
RCOR2	REST corepressor 2 gene:ENSG00000167771 /// Homo sapiens REST corepressor 2, mRNA (cDNA clone MGC:23462 IMAGE:4762190), complete cds. /// Homo sapiens REST corepressor 2 (RCOR2), mRNA.	REST corepressor 2	0.003256755	1.192863771

ID1	Isoform ID-B of DNA-binding protein inhibitor ID-1 gene:ENSG00000125968 /// Isoform ID-A of DNA-binding protein inhibitor ID-1 gene:ENSG00000125968 /// Homo sapiens inhibitor of DNA binding 1, dominant negative helix-loop-helix protein, mRNA (cDNA clone MGC:9178 IMAGE:3862019), complete cds. /// Homo sapiens inhibitor of DNA binding 1, dominant negative helix-loop-helix protein (ID1), transcript variant 2, mRNA. /// Homo sapiens inhibitor of DNA binding 1, dominant negative helix-loop-helix protein (ID1), transcript variant 1, mRNA.	inhibitor of DNA binding 1, dominant negative helix-loop-helix protein	0.001705568	1.24362227
HEXIM1	Protein HEXIM1 gene:ENSG00000186834 /// Homo sapiens mRNA for HEXIM1 protein, complete cds. /// Homo sapiens hexamethylene bis-acetamide inducible 1 (HEXIM1), mRNA.	hexamethylene bis-acetamide inducible 1	0.009376537	-1.177238359
ARID5B	Isoform 1 of AT-rich interactive domain-containing protein 5B gene:ENSG00000150347 /// Isoform 2 of AT-rich interactive domain-containing protein 5B gene:ENSG00000150347 /// Homo sapiens AT rich interactive domain 5B (MRF1-like), mRNA (cDNA clone IMAGE:3681098), partial cds. /// Homo sapiens AT rich interactive domain 5B (MRF1-like) (ARID5B), mRNA.	AT rich interactive domain 5B (MRF1-like)	2.40718E-10	-1.951024175
SNAI2	Zinc finger protein SNAI2 gene:ENSG00000019549 /// Zinc finger protein SNAI2 gene:ENSG00000019549 /// Homo sapiens snail homolog 2 (Drosophila), mRNA (cDNA clone MGC:10182 IMAGE:3908245), complete cds. /// Homo sapiens snail homolog 2 (Drosophila) (SNAI2), mRNA.	snail homolog 2 (Drosophila)	1.23517E-07	-1.793109859

UIMC1	gi|17981852|ref|NC_001807.4|:c7517-7446; gene=TRNS1; product=tRNA-Ser /// Homo sapiens X2HRIP110 (X2HRIP110) mRNA, complete cds.	ubiquitin interaction motif containing 1	0.009654337	-1.151343323
LRRFIP1	leucine rich repeat (in FLII) interacting protein 1 isoform 1 gene:ENSG00000124831 /// Isoform 2 of Leucine-rich repeat flightless-interacting protein 1 gene:ENSG00000124831 /// Isoform 1 of Leucine-rich repeat flightless-interacting protein 1 gene:ENSG00000124831 /// Isoform 3 of Leucine-rich repeat flightless-interacting protein 1 gene:ENSG00000124831 /// Homo sapiens leucine rich repeat (in FLII) interacting protein 1, mRNA (cDNA clone MGC:119739 IMAGE:40027218), complete cds. /// Homo sapiens leucine rich repeat (in FLII) interacting protein 1 (LRRFIP1), transcript variant 2, mRNA. /// Homo sapiens leucine rich repeat (in FLII) interacting protein 1 (LRRFIP1), transcript variant 3, mRNA. /// Homo sapiens leucine rich repeat (in FLII) interacting protein 1 (LRRFIP1), transcript variant 5, mRNA. /// Homo sapiens leucine rich repeat (in FLII) interacting protein 1 (LRRFIP1), transcript variant 4, mRNA. /// Homo sapiens leucine rich repeat (in FLII) interacting protein 1 (LRRFIP1), transcript variant 1, mRNA.	leucine rich repeat (in FLII) interacting protein 1	0.970893984	1.001880254

PRDM1	PR domain containing 1, with ZNF domain isoform 1 gene:ENSG00000057657 /// PR domain containing 1, with ZNF domain isoform 1 gene:ENSG00000057657 /// PR domain containing 1, with ZNF domain gene:ENSG00000057657 /// PR domain containing 1, with ZNF domain isoform 2 gene:ENSG00000057657 /// Homo sapiens PR domain-containing protein 1 alpha (PRDM1) mRNA, complete cds. /// Homo sapiens PR domain containing 1, with ZNF domain (PRDM1), transcript variant 2, mRNA. /// Homo sapiens PR domain containing 1, with ZNF domain (PRDM1), transcript variant 1, mRNA.	PR domain containing 1, with ZNF domain	5.57321E-05	-1.419559737
OVOL1	Putative transcription factor Ovo-like 1 gene:ENSG00000172818 /// Homo sapiens ovo-like 1(Drosophila), mRNA (cDNA clone MGC:71812 IMAGE:30343789), complete cds. /// Homo sapiens ovo-like 1(Drosophila) (OVOL1), mRNA.	ovo-like 1(Drosophila)	0.005165018	-1.31341653
RABGGTB	Geranylgeranyl transferase type-2 subunit beta gene:ENSG00000137955 /// Rab geranylgeranyltransferase, beta subunit variant (Fragment) gene:ENSG00000137955 /// Rab geranylgeranyltransferase, beta subunit gene:ENSG00000137955 /// Homo sapiens Rab geranylgeranyltransferase, beta subunit, mRNA (cDNA clone MGC:23688 IMAGE:4290610), complete cds. /// Homo sapiens Rab geranylgeranyltransferase, beta subunit (RABGGTB), mRNA.	Rab geranylgeranyltransferase, beta subunit	0.738662271	1.015725969

HIPK1	Isoform 4 of Homeodomain-interacting protein kinase 1 gene:ENSG00000163349 /// cDNA FLJ57817, highly similar to Homeodomain-interacting protein kinase 1 gene:ENSG00000163349 /// Homeodomain interacting protein kinase 1 gene:ENSG00000163349 /// Isoform 2 of Homeodomain-interacting protein kinase 1 gene:ENSG00000163349 /// cDNA FLJ57817, highly similar to Homeodomain-interacting protein kinase 1 gene:ENSG00000163349 /// Homeodomain interacting protein kinase 1 gene:ENSG00000163349 /// Isoform 3 of Homeodomain-interacting protein kinase 1 gene:ENSG00000163349 /// Isoform 4 of Homeodomain-interacting protein kinase 1 gene:ENSG00000163349 /// Isoform 4 of Homeodomain-interacting protein kinase 1 gene:ENSG00000163349 /// Homo sapiens HIPK1 mRNA for homeodomain-interacting protein kinase-1, complete cds. /// Homo sapiens homeodomain interacting protein kinase 1 (HIPK1), transcript variant 2, mRNA. /// Homo sapiens homeodomain interacting protein kinase 1 (HIPK1), transcript variant 1, mRNA. /// Homo sapiens homeodomain interacting protein kinase 1 (HIPK1), transcript variant 3, mRNA. /// Homo sapiens homeodomain interacting protein kinase 1 (HIPK1), transcript variant 4, mRNA.	homeodomain interacting protein kinase 1	0.009937791	1.150503935
ZNF624	zinc finger protein 624 gene:ENSG00000197566 /// Homo sapiens zinc finger protein 624, mRNA (cDNA clone MGC:119602 IMAGE:40008882), complete cds. /// Homo sapiens zinc finger protein 624 (ZNF624), mRNA.	zinc finger protein 624	0.006764563	1.298925379

ZFP36	Similar to Butyrate response factor 2 gene:ENSG00000128016 /// H.sapiens zinc finger transcriptional regulator mRNA, complete cds. /// Homo sapiens zinc finger protein 36, C3H type, homolog (mouse) (ZFP36), mRNA.	zinc finger protein 36, C3H type, homolog (mouse)	0.003252593	-1.264515417
ZNF101	Zinc finger protein 101 gene:ENSG00000181896 /// Homo sapiens cDNA FLJ39850 fis, clone SPLEN2014739, highly similar to Zinc finger protein 101. /// Homo sapiens zinc finger protein 101 (ZNF101), mRNA.	zinc finger protein 101	0.002078623	-1.156741455
ZNF79	Zinc finger protein 79 gene:ENSG00000196152 /// Homo sapiens zinc finger protein 79, mRNA (cDNA clone MGC:71125 IMAGE:3944657), complete cds. /// Homo sapiens zinc finger protein 79 (ZNF79), mRNA.	zinc finger protein 79	0.001381433	-1.246329313
RORA	Isoform Alpha-3 of Nuclear receptor ROR-alpha gene:ENSG00000069667 /// Isoform Alpha-1 of Nuclear receptor ROR-alpha gene:ENSG00000069667 /// Isoform Alpha-2 of Nuclear receptor ROR-alpha gene:ENSG00000069667 /// Human orphan hormone nuclear receptor RORalpha2 mRNA, complete cds. /// Homo sapiens RAR-related orphan receptor A (RORA), transcript variant 2, mRNA. /// Homo sapiens RAR-related orphan receptor A (RORA), transcript variant 1, mRNA. /// Homo sapiens RAR-related orphan receptor A (RORA), transcript variant 4, mRNA. /// Homo sapiens RAR-related orphan receptor A (RORA), transcript variant 3, mRNA.	RAR-related orphan receptor A	0.003579702	-1.23481767

TP53I3	Isoform 1 of Putative quinone oxidoreductase gene:ENSG00000115129 /// Isoform 2 of Putative quinone oxidoreductase gene:ENSG00000115129 /// Isoform 1 of Putative quinone oxidoreductase gene:ENSG00000115129 /// Isoform 2 of Putative quinone oxidoreductase gene:ENSG00000115129 /// Homo sapiens mRNA for tumor protein p53 inducible protein 3 variant, clone: FCC106D10. /// Homo sapiens tumor protein p53 inducible protein 3 (TP53I3), transcript variant 2, mRNA. /// Homo sapiens tumor protein p53 inducible protein 3 (TP53I3), transcript variant 1, mRNA.	tumor protein p53 inducible protein 3	0.000171373	-1.284050788
RFXANK	Isoform Long of DNA-binding protein RFXANK gene:ENSG00000064490 /// Regulatory factor X-associated ankyrin-containing protein gene:ENSG00000064490 /// Regulatory factor X-associated ankyrin-containing protein gene:ENSG00000064490 /// Isoform Long of DNA-binding protein RFXANK gene:ENSG00000064490 /// Homo sapiens regulatory factor X-associated ankyrin-containing protein, transcript variant 1, mRNA (cDNA clone MGC:138628 IMAGE:40032319), complete cds. /// Homo sapiens regulatory factor X-associated ankyrin-containing protein (RFXANK), transcript variant 2, mRNA. /// Homo sapiens regulatory factor X-associated ankyrin-containing protein (RFXANK), transcript variant 1, mRNA.	regulatory factor X-associated ankyrin-containing protein	0.000718138	1.255361495

TTC3	Isoform TRPDI of Tetratricopeptide repeat protein 3 gene:ENSG00000182670 /// Putative uncharacterized protein TTC3 gene:ENSG00000182670 /// Isoform TRPDI of Tetratricopeptide repeat protein 3 gene:ENSG00000182670 /// Isoform TRPDI of Tetratricopeptide repeat protein 3 gene:ENSG00000182670 /// Homo sapiens mRNA for possible protein TPRDII, complete cds. /// Homo sapiens tetratricopeptide repeat domain 3 (TTC3), transcript variant 1, mRNA. /// Homo sapiens tetratricopeptide repeat domain 3 (TTC3), transcript variant 2, mRNA.	tetratricopeptide repeat domain 3	0.003725 363	1.197740 027
AFF3	AF4/FMR2 family, member 3 isoform 1 gene:ENSG00000144218 /// AF4/FMR2 family, member 3 isoform 2 gene:ENSG00000144218 /// cdna:known chromosome:NCBI36:2:99534015:100 089022:-1 gene:ENSG00000144218 /// cdna:known chromosome:NCBI36:2:99534248:100 087610:-1 gene:ENSG00000144218 /// Human lymphoid nuclear protein (LAF-4) mRNA, complete cds. /// Homo sapiens AF4/FMR2 family, member 3 (AFF3), transcript variant 1, mRNA. /// Homo sapiens AF4/FMR2 family, member 3 (AFF3), transcript variant 2, mRNA.	AF4/FMR2 family, member 3	0.007118 051	- 1.315258 268
AHR	Aryl hydrocarbon receptor gene:ENSG00000106546 /// Aryl hydrocarbon receptor gene:ENSG00000106546 /// Homo sapiens aryl hydrocarbon receptor, mRNA (cDNA clone MGC:87401 IMAGE:30342582), complete cds. /// Homo sapiens aryl hydrocarbon receptor (AHR), mRNA.	aryl hydrocarbon receptor	0.000716 366	- 1.279894 642

LPP	Lipoma-preferred partner gene:ENSG00000145012 /// Homo sapiens LIM domain containing preferred translocation partner in lipoma, mRNA (cDNA clone MGC:163456 IMAGE:40146615), complete cds. /// Homo sapiens LIM domain containing preferred translocation partner in lipoma (LPP), mRNA.	LIM domain containing preferred translocation partner in lipoma	0.000590115	-1.2073213
ZRSR1	U2 small nuclear ribonucleoprotein auxiliary factor 35 kDa subunit-related protein 1 gene:ENSG00000212643 /// Homo sapiens zinc finger (CCCH type), RNA-binding motif and serine/arginine rich 1, mRNA (cDNA clone MGC:132471 IMAGE:8143814), complete cds.	zinc finger (CCCH type), RNA-binding motif and serine/arginine rich 1	2.41964E-06	-1.590036605
PRDM14	PR domain zinc finger protein 14 gene:ENSG00000147596 /// Homo sapiens PR-domain containing protein 14 (PRDM14) mRNA, complete cds. /// Homo sapiens PR domain containing 14 (PRDM14), mRNA.	PR domain containing 14	1.30483E-05	1.316307536
ZNF28	Isoform 1 of Zinc finger protein 28 gene:ENSG00000198538 /// Homo sapiens cDNA FLJ58669 complete cds, highly similar to Zinc finger protein 28. /// Homo sapiens zinc finger protein 28 (ZNF28), mRNA.	zinc finger protein 28	0.002340631	-1.179948066
ZBED4	Zinc finger BED domain-containing protein 4 gene:ENSG00000100426 /// Zinc finger BED domain-containing protein 4 gene:ENSG00000100426 /// Homo sapiens zinc finger, BED-type containing 4, mRNA (cDNA clone MGC:176685 IMAGE:8862564), complete cds. /// Homo sapiens zinc finger, BED-type containing 4 (ZBED4), mRNA.	zinc finger, BED-type containing 4	0.0012558	1.242612363

DNAJA4	DnaJ (Hsp40) homolog, subfamily A, member 4 isoform 1 gene:ENSG00000140403 /// DnaJ homolog subfamily A member 4 gene:ENSG00000140403 /// DnaJ homolog subfamily A member 4 gene:ENSG00000140403 /// Homo sapiens DnaJ (Hsp40) homolog, subfamily A, member 4, mRNA (cDNA clone MGC:33001 IMAGE:4831235), complete cds. /// Homo sapiens DnaJ (Hsp40) homolog, subfamily A, member 4 (DNAJA4), transcript variant 1, mRNA. /// Homo sapiens DnaJ (Hsp40) homolog, subfamily A, member 4 (DNAJA4), transcript variant 2, mRNA. /// Homo sapiens DnaJ (Hsp40) homolog, subfamily A, member 4 (DNAJA4), transcript variant 3, mRNA.	DnaJ (Hsp40) homolog, subfamily A, member 4	0.00145855	-1.271389224
ASH1L	Probable histone-lysine N-methyltransferase ASH1L gene:ENSG00000116539 /// ash1 (absent, small, or homeotic)-like gene:ENSG00000116539 /// Homo sapiens ASH1 mRNA, complete cds. /// Homo sapiens ash1 (absent, small, or homeotic)-like (Drosophila) (ASH1L), mRNA.	ash1 (absent, small, or homeotic)-like (Drosophila)	0.006650968	-1.182650113

SMYD3	Isoform 1 of SET and MYND domain-containing protein 3 gene:ENSG00000185420 /// Isoform 2 of SET and MYND domain-containing protein 3 gene:ENSG00000185420 /// Isoform 3 of SET and MYND domain-containing protein 3 gene:ENSG00000185420 /// Isoform 3 of SET and MYND domain-containing protein 3 gene:ENSG00000185420 /// Isoform 2 of SET and MYND domain-containing protein 3 gene:ENSG00000185420 /// SET and MYND domain containing 3 gene:ENSG00000185420 /// Homo sapiens SMYD3 mRNA for histone methyltransferase, complete cds. /// Homo sapiens SET and MYND domain containing 3 (SMYD3), mRNA.	SET and MYND domain containing 3	0.007585 27	1.201479 429
POLR2F	Polymerase (RNA) II (DNA directed) polypeptide F gene:ENSG00000100142 /// DNA-directed RNA polymerases I, II, and III subunit RPABC2 gene:ENSG00000100142 /// Homo sapiens polymerase (RNA) II (DNA directed) polypeptide F, mRNA (cDNA clone MGC:2669 IMAGE:3546712), complete cds. /// Homo sapiens polymerase (RNA) II (DNA directed) polypeptide F (POLR2F), mRNA.	polymerase (RNA) II (DNA directed) polypeptide F	0.002220 047	1.202293 818
ZNF486	Zinc finger protein 486 gene:ENSG00000160229 /// Homo sapiens zinc finger protein 486, mRNA (cDNA clone IMAGE:4829685), partial cds. /// Homo sapiens zinc finger protein 486 (ZNF486), mRNA.	zinc finger protein 486	0.002249 4	- 1.160571 819

ATF3	Isoform 1 of Cyclic AMP-dependent transcription factor ATF-3 gene:ENSG00000162772 /// Isoform 1 of Cyclic AMP-dependent transcription factor ATF-3 gene:ENSG00000162772 /// activating transcription factor 3 isoform 2 gene:ENSG00000162772 /// Activating transcription factor 3 delta Zip3 gene:ENSG00000162772 /// Activating transcription factor 3 gene:ENSG00000162772 /// Activating transcription factor 3 delta Zip2c gene:ENSG00000162772 /// activating transcription factor 3 isoform 2 gene:ENSG00000162772 /// Activating transcription factor 3 delta Zip2c gene:ENSG00000162772 /// Homo sapiens mRNA for activating transcription factor 3 long isoform variant protein. /// Homo sapiens activating transcription factor 3 (ATF3), transcript variant 1, mRNA. /// Homo sapiens activating transcription factor 3 (ATF3), transcript variant 3, mRNA. /// Homo sapiens activating transcription factor 3 (ATF3), transcript variant 2, mRNA. /// Homo sapiens activating transcription factor 3 (ATF3), transcript variant 4, mRNA.	activating transcription factor 3	0.000737282	-1.356703295

FANCL	Putative uncharacterized protein FANCL gene:ENSG00000115392 /// E3 ubiquitin-protein ligase FANCL gene:ENSG00000115392 /// Fanconi anemia, complementation group L isoform 1 gene:ENSG00000115392 /// Putative uncharacterized protein FANCL gene:ENSG00000115392 /// Homo sapiens Fanconi anemia, complementation group L, mRNA (cDNA clone MGC:60295 IMAGE:5180184), complete cds. /// Homo sapiens Fanconi anemia, complementation group L (FANCL), transcript variant 2, mRNA. /// Homo sapiens Fanconi anemia, complementation group L (FANCL), transcript variant 1, mRNA.	Fanconi anemia, complementation group L	0.006341 69	1.239930 586
NSMCE1	Non-structural maintenance of chromosomes element 1 homolog gene:ENSG00000169189 /// Putative uncharacterized protein NSMCE1 (Fragment) gene:ENSG00000169189 /// Homo sapiens cDNA FLJ32233 fis, clone PLACE6004663. /// Homo sapiens non-SMC element 1 homolog (S. cerevisiae) (NSMCE1), mRNA.	non-SMC element 1 homolog (S. cerevisiae)	0.005526 628	1.158631 589
TBL1X	transducin beta-like 1X isoform a gene:ENSG00000101849 /// F-box-like/WD repeat-containing protein TBL1X gene:ENSG00000101849 /// transducin beta-like 1X isoform a gene:ENSG00000101849 /// Homo sapiens transducin (beta)-like 1X-linked, mRNA (cDNA clone MGC:45225 IMAGE:5496758), complete cds. /// Homo sapiens transducin (beta)-like 1X-linked (TBL1X), mRNA.	transducin (beta)-like 1X-linked	0.002114 75	1.234673 068

GTF3C1	Putative uncharacterized protein GTF3C1 gene:ENSG00000077235 /// Isoform 1 of General transcription factor 3C polypeptide 1 gene:ENSG00000077235 /// GTF3C1 protein gene:ENSG00000077235 /// Homo sapiens general transcription factor IIIC, polypeptide 1, alpha 220kDa, mRNA (cDNA clone MGC:168849 IMAGE:9021226), complete cds. /// Homo sapiens general transcription factor IIIC, polypeptide 1, alpha 220kDa (GTF3C1), mRNA.	general transcription factor IIIC, polypeptide 1, alpha 220kDa	0.009933 241	- 1.140062 433
PSIP1	Isoform 1 of PC4 and SFRS1-interacting protein gene:ENSG00000164985 /// Isoform 1 of PC4 and SFRS1-interacting protein gene:ENSG00000164985 /// PSIP1 protein gene:ENSG00000164985 /// PSIP1 protein gene:ENSG00000164985 /// Isoform 2 of PC4 and SFRS1-interacting protein gene:ENSG00000164985 /// Homo sapiens lens epithelium-derived growth factor mRNA, complete cds. /// Homo sapiens PC4 and SFRS1 interacting protein 1 (PSIP1), transcript variant 2, mRNA. /// Homo sapiens PC4 and SFRS1 interacting protein 1 (PSIP1), transcript variant 1, mRNA. /// Homo sapiens PC4 and SFRS1 interacting protein 1 (PSIP1), transcript variant 3, mRNA.	PC4 and SFRS1 interacting protein 1	0.002345 201	1.189283 64
ACTL6A	Isoform 2 of Actin-like protein 6A gene:ENSG00000136518 /// Isoform 1 of Actin-like protein 6A gene:ENSG00000136518 /// Homo sapiens actin-like 6A, mRNA (cDNA clone MGC:26443 IMAGE:4827785), complete cds. /// Homo sapiens actin-like 6A (ACTL6A), transcript variant 1, mRNA. /// Homo sapiens actin-like 6A (ACTL6A), transcript variant 2, mRNA. /// Homo sapiens actin-like 6A (ACTL6A), transcript variant 3, mRNA.	actin-like 6A	0.005685 442	1.184736 011

TFAP4	Transcription factor AP-4 (Activating enhancer binding protein 4) variant (Fragment) gene:ENSG00000090447 /// Transcription factor AP-4 gene:ENSG00000090447 /// AP-4=basic helix-loop-helix DNA-binding protein [human, cervical carcinoma, HeLa cells, mRNA, 2149 nt]. /// Homo sapiens transcription factor AP-4 (activating enhancer binding protein 4) (TFAP4), mRNA.	transcription factor AP-4 (activating enhancer binding protein 4)	0.005101753	1.227006686
ZNF440	Zinc finger protein 440 gene:ENSG00000171295 /// Homo sapiens zinc finger protein 440, mRNA (cDNA clone MGC:46665 IMAGE:5556302), complete cds. /// Homo sapiens zinc finger protein 440 (ZNF440), mRNA.	zinc finger protein 440	0.008568226	-1.235795754
ZMAT3	Zinc finger matrin-type protein 3 gene:ENSG00000172667 /// Homo sapiens zinc finger, matrin type 3, mRNA (cDNA clone MGC:10613 IMAGE:3943641), complete cds. /// Homo sapiens zinc finger, matrin type 3 (ZMAT3), transcript variant 2, mRNA. /// Homo sapiens zinc finger, matrin type 3 (ZMAT3), transcript variant 1, mRNA.	zinc finger, matrin type 3	0.003712906	-1.21206409
SP4	Transcription factor Sp4 gene:ENSG00000105866 /// Homo sapiens cDNA FLJ76740 complete cds, highly similar to Homo sapiens Sp4 transcription factor (SP4), mRNA. /// Homo sapiens Sp4 transcription factor (SP4), mRNA.	Sp4 transcription factor	0.001577765	1.183771619
ZBTB25	Zinc finger and BTB domain-containing protein 25 gene:ENSG00000089775 /// Zinc finger and BTB domain-containing protein 25 gene:ENSG00000089775 /// Homo sapiens zinc finger and BTB domain containing 25, mRNA (cDNA clone MGC:46128 IMAGE:5745935), complete cds. /// Homo sapiens zinc finger and BTB domain containing 25 (ZBTB25), mRNA.	zinc finger and BTB domain containing 25	0.008132439	1.220112727

TRIM13	Isoform Alpha of Tripartite motif-containing protein 13 gene:ENSG00000204977 /// ret finger protein 2 isoform 2 gene:ENSG00000204977 /// Isoform Alpha of Tripartite motif-containing protein 13 gene:ENSG00000204977 /// Isoform Alpha of Tripartite motif-containing protein 13 gene:ENSG00000204977 /// Isoform 2 of Putative potassium channel regulatory protein gene:ENSG00000198553 /// Isoform 1 of Putative potassium channel regulatory protein gene:ENSG00000198553 /// Homo sapiens putative potassium channel proteins regulator protein variant A (KCNRG) mRNA, complete cds; alternatively spliced. /// Homo sapiens leu5 (RFP2) mRNA, complete cds. /// Homo sapiens potassium channel regulator (KCNRG), transcript variant 1, mRNA. /// Homo sapiens tripartite motif-containing 13 (TRIM13), transcript variant 4, mRNA. /// Homo sapiens potassium channel regulator (KCNRG), transcript variant 2, mRNA. /// Homo sapiens tripartite motif-containing 13 (TRIM13), transcript variant 1, mRNA. /// Homo sapiens tripartite motif-containing 13 (TRIM13), transcript variant 2, mRNA. /// Homo sapiens tripartite motif-containing 13 (TRIM13), transcript variant 3, mRNA.	potassium channel regulator /// tripartite motif-containing 13	0.008562 401	1.232643 517

MAP3K7IP3	Isoform 1 of Mitogen-activated protein kinase kinase kinase 7-interacting protein 3 gene:ENSG00000157625 /// Isoform 1 of Mitogen-activated protein kinase kinase kinase 7-interacting protein 3 gene:ENSG00000157625 /// Isoform 1 of Mitogen-activated protein kinase kinase kinase 7-interacting protein 3 gene:ENSG00000157625 /// cDNA FLJ60687, highly similar to Mitogen-activated protein kinase kinase kinase7-interacting protein 3 gene:ENSG00000157625 /// Putative uncharacterized protein MAP3K7IP3 gene:ENSG00000157625 /// Homo sapiens NFkB activating protein 1 isoform A (TAB3) mRNA, complete cds, alternatively spliced. /// Homo sapiens mitogen-activated protein kinase kinase kinase 7 interacting protein 3 (MAP3K7IP3), mRNA.	mitogen-activated protein kinase kinase kinase 7 interacting protein 3	0.003752 719	- 1.210958 631
DZIP3	Isoform 2 of E3 ubiquitin-protein ligase DZIP3 gene:ENSG00000198919 /// Isoform 1 of E3 ubiquitin-protein ligase DZIP3 gene:ENSG00000198919 /// Homo sapiens DAZ interacting protein 3, zinc finger, mRNA (cDNA clone MGC:75477 IMAGE:30387514), complete cds. /// Homo sapiens DAZ interacting protein 3, zinc finger (DZIP3), mRNA.	DAZ interacting protein 3, zinc finger	0.000680 286	- 1.196710 337
MYOCD	cDNA FLJ78477, highly similar to Homo sapiens myocardin gene:ENSG00000141052 /// Putative uncharacterized protein DKFZp686O15128 gene:ENSG00000141052 /// Isoform 1 of Myocardin gene:ENSG00000141052 /// Isoform 2 of Myocardin gene:ENSG00000141052 /// Homo sapiens myocardin mRNA, complete cds, alternatively spliced. /// Homo sapiens myocardin (MYOCD), mRNA.	myocardin	0.001318 582	- 1.320402 281

TRIM14	Isoform Alpha of Tripartite motif-containing protein 14 gene:ENSG00000106785 /// Isoform Beta of Tripartite motif-containing protein 14 gene:ENSG00000106785 /// Isoform Alpha of Tripartite motif-containing protein 14 gene:ENSG00000106785 /// Isoform Alpha of Tripartite motif-containing protein 14 gene:ENSG00000106785 /// Tripartite motif protein TRIM14 alpha gene:ENSG00000106785 /// Homo sapiens mRNA for KIAA0129 gene, complete cds. /// Homo sapiens tripartite motif-containing 14 (TRIM14), transcript variant 3, mRNA. /// Homo sapiens tripartite motif-containing 14 (TRIM14), transcript variant 4, mRNA. /// Homo sapiens tripartite motif-containing 14 (TRIM14), transcript variant 2, mRNA. /// Homo sapiens tripartite motif-containing 14 (TRIM14), transcript variant 1, mRNA.	tripartite motif-containing 14	0.006973195	-1.185822344
ZC3H11A	Zinc finger CCCH domain-containing protein 11A gene:ENSG00000058673 /// Zinc finger CCCH domain-containing protein 11A gene:ENSG00000058673 /// Zinc finger CCCH domain-containing protein 11A gene:ENSG00000058673 /// Zinc finger CCCH domain-containing protein 11A gene:ENSG00000058673 /// Homo sapiens zinc finger CCCH-type containing 11A, mRNA (cDNA clone IMAGE:5262797). /// Homo sapiens zinc finger CCCH-type containing 11A (ZC3H11A), mRNA.	zinc finger CCCH-type containing 11A	0.587235771	1.027739519
HMG20A	Isoform 1 of High mobility group protein 20A gene:ENSG00000140382 /// Isoform 1 of High mobility group protein 20A gene:ENSG00000140382 /// Homo sapiens high-mobility group 20A, mRNA (cDNA clone MGC:8813 IMAGE:3908842), complete cds. /// Homo sapiens high-mobility group 20A (HMG20A), mRNA.	high-mobility group 20A	0.008452154	1.152143821

TRAIP	TRAF-interacting protein gene:ENSG00000183763 /// Homo sapiens TRAF interacting protein, mRNA (cDNA clone MGC:3999 IMAGE:2821007), complete cds. /// Homo sapiens TRAF interacting protein (TRAIP), mRNA.	TRAF interacting protein	0.004638021	1.182918593
ZC3H6	cdna:known chromosome:NCBI36:2:112749642:112814105:1 gene:ENSG00000188177 /// Zinc finger CCCH domain-containing protein 6 gene:ENSG00000188177 /// Homo sapiens zinc finger CCCH-type containing 6 (ZC3H6), mRNA.	zinc finger CCCH-type containing 6	0.005746652	1.30018392
PHF21B	Isoform 2 of PHD finger protein 21B gene:ENSG00000056487 /// PHD finger protein 21B, isoform CRA_c gene:ENSG00000056487 /// Isoform 1 of PHD finger protein 21B gene:ENSG00000056487 /// 46 kDa protein gene:ENSG00000056487 /// Homo sapiens PHD finger protein 21B, mRNA (cDNA clone MGC:20476 IMAGE:4634631), complete cds. /// Homo sapiens PHD finger protein 21B (PHF21B), transcript variant 1, mRNA. /// Homo sapiens PHD finger protein 21B (PHF21B), transcript variant 2, mRNA.	PHD finger protein 21B	0.000924157	-1.245954349

BRDT	Isoform 2 of Bromodomain testis-specific protein gene:ENSG00000137948 /// Isoform 1 of Bromodomain testis-specific protein gene:ENSG00000137948 /// Isoform 2 of Bromodomain testis-specific protein gene:ENSG00000137948 /// Isoform 1 of Bromodomain testis-specific protein gene:ENSG00000137948 /// Isoform 1 of Bromodomain testis-specific protein gene:ENSG00000137948 /// Homo sapiens testis-specific BRDT protein mRNA, complete cds. /// Homo sapiens bromodomain, testis-specific (BRDT), transcript variant 2, mRNA. /// Homo sapiens bromodomain, testis-specific (BRDT), transcript variant 1, mRNA.	bromodomain, testis-specific	4.80202E-05	-1.685988704
MORC4	MORC family CW-type zinc finger protein 4 gene:ENSG00000133131 /// zinc finger, CW type with coiled-coil domain 2 isoform b gene:ENSG00000133131 /// MORC family CW-type zinc finger protein 4 gene:ENSG00000133131 /// Homo sapiens clone IMAGE: IMGCLO30344723 MORC family CW-type zinc finger protein 4 (MORC4) mRNA, complete cds, alternatively spliced. /// Homo sapiens MORC family CW-type zinc finger 4 (MORC4), transcript variant 1, mRNA. /// Homo sapiens MORC family CW-type zinc finger 4 (MORC4), transcript variant 2, mRNA.	MORC family CW-type zinc finger 4	0.00331424	-1.332075391

HDAC6	cDNA FLJ14021 fis, clone HEMBA1002513, highly similar to Histone deacetylase 6 (Fragment) gene:ENSG00000094631 /// cDNA FLJ14021 fis, clone HEMBA1002513, highly similar to Histone deacetylase 6 (Fragment) gene:ENSG00000094631 /// cDNA FLJ56474, highly similar to Histone deacetylase 6 gene:ENSG00000094631 /// cDNA FLJ56474, highly similar to Histone deacetylase 6 gene:ENSG00000094631 /// Homo sapiens histone deacetylase 6, mRNA (cDNA clone MGC:78509 IMAGE:6584427), complete cds. /// Homo sapiens histone deacetylase 6 (HDAC6), mRNA.	histone deacetylase 6	0.001852969	1.237739731
SMARCB1	Isoform A of SWI/SNF-related matrix-associated actin-dependent regulator of chromatin subfamily B member 1 gene:ENSG00000099956 /// cDNA FLJ13963 fis, clone Y79AA1001299, highly similar to Homo sapiens integrase interactor 1b protein gene:ENSG00000099956 /// Isoform B of SWI/SNF-related matrix-associated actin-dependent regulator of chromatin subfamily B member 1 gene:ENSG00000099956 /// Putative uncharacterized protein SMARCB1 gene:ENSG00000099956 /// Homo sapiens cDNA FLJ13963 fis, clone Y79AA1001299, highly similar to Homo sapiens mRNA for integrase interactor 1b protein (INI1B). /// Homo sapiens SWI/SNF related, matrix associated, actin dependent regulator of chromatin, subfamily b, member 1 (SMARCB1), transcript variant 1, mRNA. /// Homo sapiens SWI/SNF related, matrix associated, actin dependent regulator of chromatin, subfamily b, member 1 (SMARCB1), transcript variant 2, mRNA.	SWI/SNF related, matrix associated, actin dependent regulator of chromatin, subfamily b, member 1	0.005212506	1.159324321

ZBTB5	Zinc finger and BTB domain-containing protein 5 gene:ENSG00000168795 /// Homo sapiens cDNA FLJ77230 complete cds, highly similar to Homo sapiens zinc finger and BTB domain containing 5 (ZBTB5), mRNA. /// Homo sapiens zinc finger and BTB domain containing 5 (ZBTB5), mRNA.	zinc finger and BTB domain containing 5	0.002886046	-1.255032589
ZRANB3	Isoform 2 of Zinc finger Ran-binding domain-containing protein 3 gene:ENSG00000121988 /// Isoform 2 of Zinc finger Ran-binding domain-containing protein 3 gene:ENSG00000121988 /// Isoform 1 of Zinc finger Ran-binding domain-containing protein 3 gene:ENSG00000121988 /// Isoform 1 of Zinc finger Ran-binding domain-containing protein 3 gene:ENSG00000121988 /// cDNA FLJ38043 fis, clone CTONG2014058, weakly similar to HepA-related protein gene:ENSG00000121988 /// Homo sapiens mRNA; cDNA DKFZp686D0128 (from clone DKFZp686D0128). /// Homo sapiens zinc finger, RAN-binding domain containing 3 (ZRANB3), mRNA.	zinc finger, RAN-binding domain containing 3	0.060927073	1.149254077
HELZ	Probable helicase with zinc finger domain gene:ENSG00000198265 /// Homo sapiens helicase with zinc finger, mRNA (cDNA clone MGC:163454 IMAGE:40146613), complete cds. /// Homo sapiens helicase with zinc finger (HELZ), mRNA.	helicase with zinc finger	1.11093E-07	-1.384436629

ZNF385A	zinc finger protein 385A isoform b gene:ENSG00000161642 /// Isoform 1 of Zinc finger protein 385A gene:ENSG00000161642 /// Isoform 1 of Zinc finger protein 385A gene:ENSG00000161642 /// Homo sapiens hematopoietic zinc finger protein (HZF) mRNA, complete cds. /// Homo sapiens zinc finger protein 385A (ZNF385A), transcript variant 1, mRNA. /// Homo sapiens zinc finger protein 385A (ZNF385A), transcript variant 2, mRNA. /// Homo sapiens zinc finger protein 385A (ZNF385A), transcript variant 3, mRNA.	zinc finger protein 385A	0.006703349	-1.184322958
FOXJ1	Forkhead box protein J1 gene:ENSG00000129654 /// Homo sapiens forkhead box J1, mRNA (cDNA clone MGC:35202 IMAGE:5171637), complete cds. /// Homo sapiens forkhead box J1 (FOXJ1), mRNA.	forkhead box J1	0.000604848	-1.290465848
LCORL	Isoform 3 of Ligand-dependent nuclear receptor corepressor-like protein gene:ENSG00000178177 /// Isoform 1 of Ligand-dependent nuclear receptor corepressor-like protein gene:ENSG00000178177 /// Isoform 1 of Ligand-dependent nuclear receptor corepressor-like protein gene:ENSG00000178177 /// Homo sapiens ligand dependent nuclear receptor corepressor-like, mRNA (cDNA clone MGC:43101 IMAGE:5260413), complete cds. /// Homo sapiens ligand dependent nuclear receptor corepressor-like (LCORL), mRNA.	ligand dependent nuclear receptor corepressor-like	0.007581431	1.212379527

LMO3	LIM domain only protein 3 gene:ENSG00000048540 /// Neuronal specific transcription factor DAT1 gene:ENSG00000048540 /// LIM domain only protein 3 gene:ENSG00000048540 /// Putative uncharacterized protein Nbla03267 gene:ENSG00000048540 /// Homo sapiens LIM domain only 3 (rhombotin-like 2), mRNA (cDNA clone MGC:26081 IMAGE:4799836), complete cds. /// Homo sapiens LIM domain only 3 (rhombotin-like 2) (LMO3), transcript variant 1, mRNA. /// Homo sapiens LIM domain only 3 (rhombotin-like 2) (LMO3), transcript variant 2, mRNA.	LIM domain only 3 (rhombotin-like 2)	0.003242393	1.343951303
NSBP1	Nucleosome-binding protein 1 gene:ENSG00000198157 /// Nucleosomal binding protein 1 gene:ENSG00000198157 /// Nucleosomal binding protein 1 gene:ENSG00000198157 /// Nucleosome-binding protein 1 gene:ENSG00000198157 /// Homo sapiens cDNA FLJ36739 fis, clone UTERU2013262. /// Homo sapiens nucleosomal binding protein 1 (NSBP1), mRNA.	nucleosomal binding protein 1	0.008542632	1.222686506
E2F7	Isoform 1 of Transcription factor E2F7 gene:ENSG00000165891 /// Putative uncharacterized protein E2F7 gene:ENSG00000165891 /// Homo sapiens E2F transcription factor 7, mRNA (cDNA clone MGC:167976 IMAGE:9020353), complete cds. /// Homo sapiens E2F transcription factor 7 (E2F7), mRNA.	E2F transcription factor 7	1.68088E-05	-1.315656104

FMNL2	cdna:known chromosome:NCBI36:2:152899997:153214594:1 gene:ENSG00000157827 /// Isoform 3 of Formin-like protein 2 gene:ENSG00000157827 /// Homo sapiens formin-like 2, mRNA (cDNA clone MGC:176702 IMAGE:8862581), complete cds. /// Homo sapiens formin-like 2 (FMNL2), mRNA.	formin-like 2	0.007124974	-1.189287247
POLR2L	DNA-directed RNA polymerases I, II, and III subunit RPABC5 gene:ENSG00000177700 /// Homo sapiens polymerase (RNA) II (DNA directed) polypeptide L, 7.6kDa, mRNA (cDNA clone MGC:2137 IMAGE:2958832), complete cds. /// Homo sapiens polymerase (RNA) II (DNA directed) polypeptide L, 7.6kDa (POLR2L), mRNA.	polymerase (RNA) II (DNA directed) polypeptide L, 7.6kDa	0.002227826	1.192438112
NKX2-4	Homeobox protein Nkx-2.4 gene:ENSG00000125816 /// Homo sapiens NK2 homeobox 4 (NKX2-4), mRNA.	NK2 homeobox 4	0.000665485	-1.265204559
TXNIP	Thioredoxin-interacting protein gene:ENSG00000117289 /// Homo sapiens brain-expressed HHCPA78 homolog VDUP1 (Gene) mRNA, complete cds. /// Homo sapiens thioredoxin interacting protein (TXNIP), mRNA.	thioredoxin interacting protein	0.000113579	1.270264433
PARP14	PARP14 protein gene:ENSG00000173193 /// poly (ADP-ribose) polymerase family, member 14 gene:ENSG00000173193 /// Isoform 1 of Poly [ADP-ribose] polymerase 14 gene:ENSG00000173193 /// Isoform 5 of Poly [ADP-ribose] polymerase 14 gene:ENSG00000173193 /// Homo sapiens mRNA; cDNA DKFZp686O21109 (from clone DKFZp686O21109). /// Homo sapiens poly (ADP-ribose) polymerase family, member 14 (PARP14), mRNA.	poly (ADP-ribose) polymerase family, member 14	0.004544963	-1.202786113

SCML1	Sex comb on midleg-like protein 1 gene:ENSG00000047634 /// Sex comb on midleg-like protein 1 gene:ENSG00000047634 /// sex comb on midleg-like 1 isoform b gene:ENSG00000047634 /// sex comb on midleg-like 1 isoform a gene:ENSG00000047634 /// Sex comb on midleg-like protein 1 gene:ENSG00000047634 /// Homo sapiens mRNA; cDNA DKFZp686H0231 (from clone DKFZp686H0231). /// Homo sapiens sex comb on midleg-like 1 (Drosophila) (SCML1), transcript variant 2, mRNA. /// Homo sapiens sex comb on midleg-like 1 (Drosophila) (SCML1), transcript variant 3, mRNA. /// Homo sapiens sex comb on midleg-like 1 (Drosophila) (SCML1), transcript variant 4, mRNA. /// Homo sapiens sex comb on midleg-like 1 (Drosophila) (SCML1), transcript variant 1, mRNA.	sex comb on midleg-like 1 (Drosophila)	3.55161E-05	-1.563168667
ZNF468	Isoform 1 of Zinc finger protein 468 gene:ENSG00000204604 /// Isoform 2 of Zinc finger protein 468 gene:ENSG00000204604 /// Homo sapiens zinc finger protein ZNF468.2 mRNA, complete cds. /// Homo sapiens zinc finger protein 468 (ZNF468), transcript variant 2, mRNA. /// Homo sapiens zinc finger protein 468 (ZNF468), transcript variant 1, mRNA.	zinc finger protein 468	5.52971E-05	-1.313398464

ZNRD1	DNA-directed RNA polymerase I subunit RPA12 gene:ENSG00000206429 /// DNA-directed RNA polymerase I subunit RPA12 gene:ENSG00000206429 /// DNA-directed RNA polymerase I subunit RPA12 gene:ENSG00000206429 /// DNA-directed RNA polymerase I subunit RPA12 gene:ENSG00000206429 /// DNA-directed RNA polymerase I subunit RPA12 gene:ENSG00000206502 /// DNA-directed RNA polymerase I subunit RPA12 gene:ENSG00000206502 /// DNA-directed RNA polymerase I subunit RPA12 gene:ENSG00000206502 /// DNA-directed RNA polymerase I subunit RPA12 gene:ENSG00000206502 /// DNA-directed RNA polymerase I subunit RPA12 gene:ENSG00000066379 /// DNA-directed RNA polymerase I subunit RPA12 gene:ENSG00000066379 /// DNA-directed RNA polymerase I subunit RPA12 gene:ENSG00000066379 /// DNA-directed RNA polymerase I subunit RPA12 gene:ENSG00000066379 /// Homo sapiens unknown mRNA. /// Homo sapiens zinc ribbon domain containing 1 (ZNRD1), transcript variant a, mRNA. /// Homo sapiens zinc ribbon domain containing 1 (ZNRD1), transcript variant b, mRNA.	zinc ribbon domain containing 1	0.022210689	1.223992444

ALS2CR8	Isoform 1 of Amyotrophic lateral sclerosis 2 chromosomal region candidate gene 8 protein gene:ENSG00000138380 /// Isoform 1 of Amyotrophic lateral sclerosis 2 chromosomal region candidate gene 8 protein gene:ENSG00000138380 /// Homo sapiens amyotrophic lateral sclerosis 2 (juvenile) chromosome region, candidate 8, mRNA (cDNA clone MGC:44837 IMAGE:4521845), complete cds. /// Homo sapiens amyotrophic lateral sclerosis 2 (juvenile) chromosome region, candidate 8 (ALS2CR8), mRNA. /// Homo sapiens amyotrophic lateral sclerosis 2 (juvenile) chromosome region, candidate 8 (ALS2CR8), mRNA.	amyotrophic lateral sclerosis 2 (juvenile) chromosome region, candidate 8	0.005081142	1.390537787
MAFA	v-maf musculoaponeurotic fibrosarcoma oncogene homolog A gene:ENSG00000182759 /// Homo sapiens v-maf musculoaponeurotic fibrosarcoma oncogene homolog A (avian) (MAFA), mRNA.	v-maf musculoaponeurotic fibrosarcoma oncogene homolog A (avian)	0.006853041	-1.260242866
ANKRD1	Ankyrin repeat domain-containing protein 1 gene:ENSG00000148677 /// Homo sapiens ankyrin repeat domain 1 (cardiac muscle), mRNA (cDNA clone MGC:27140 IMAGE:4245591), complete cds. /// Homo sapiens ankyrin repeat domain 1 (cardiac muscle) (ANKRD1), mRNA.	ankyrin repeat domain 1 (cardiac muscle)	0.002642587	-1.185271645
ZNF660	Zinc finger protein 660 gene:ENSG00000144792 /// Homo sapiens zinc finger protein 660, mRNA (cDNA clone MGC:103985 IMAGE:30915393), complete cds. /// Homo sapiens zinc finger protein 660 (ZNF660), mRNA.	zinc finger protein 660	0.00055386	1.34873812

FEZF1	Isoform 3 of Fez family zinc finger protein 1 gene:ENSG00000128610 /// Homo sapiens cDNA FLJ54502 complete cds, highly similar to Homo sapiens zinc finger protein 312-like (LOC389549), mRNA. /// Homo sapiens FEZ family zinc finger 1 (FEZF1), mRNA.	FEZ family zinc finger 1	0.002294603	-1.354659296
PHF20	Isoform 1 of PHD finger protein 20 gene:ENSG00000025293 /// 18 kDa protein gene:ENSG00000025293 /// Isoform 2 of PHD finger protein 20 gene:ENSG00000025293 /// PHD finger protein 20 gene:ENSG00000025293 /// Homo sapiens PHD finger protein 20, mRNA (cDNA clone MGC:164895 IMAGE:40148074), complete cds. /// Homo sapiens PHD finger protein 20 (PHF20), mRNA.	PHD finger protein 20	0.001217769	1.240176021
BTG2	Protein BTG2 gene:ENSG00000159388 /// Human BTG2 (BTG2) mRNA, complete cds. /// Homo sapiens BTG family, member 2 (BTG2), mRNA.	BTG family, member 2	0.000131374	-1.338043485
ZNF790	Zinc finger protein 790 gene:ENSG00000197863 /// Homo sapiens zinc finger protein 790, mRNA (cDNA clone IMAGE:6712908). /// Homo sapiens zinc finger protein 790 (ZNF790), mRNA.	zinc finger protein 790	0.001199618	-1.239368069

MTA3	61 kDa protein gene:ENSG00000057935 /// 61 kDa protein gene:ENSG00000057935 /// Isoform 2 of Metastasis-associated protein MTA3 gene:ENSG00000057935 /// Isoform 1 of Metastasis-associated protein MTA3 gene:ENSG00000057935 /// 59 kDa protein gene:ENSG00000057935 /// cdna:known chromosome:NCBI36:2:42649382:42834704:1 gene:ENSG00000057935 /// Isoform 1 of Metastasis-associated protein MTA3 gene:ENSG00000057935 /// Homo sapiens cDNA FLJ45312 fis, clone BRHIP3005037, moderately similar to Metastasis-associated protein MTA1. /// Homo sapiens metastasis associated 1 family, member 3 (MTA3), mRNA.	metastasis associated 1 family, member 3	0.001439493	1.188019482
ZNF559	Zinc finger protein 559 gene:ENSG00000188321 /// Zinc finger protein 559 gene:ENSG00000188321 /// Homo sapiens zinc finger protein 559, mRNA (cDNA clone MGC:13105 IMAGE:3957973), complete cds. /// Homo sapiens zinc finger protein 559 (ZNF559), mRNA.	zinc finger protein 559	0.004642251	1.173094787
TAF13	cdna:known supercontig::NT_113870:35367:37336:-1 gene:ENSG00000215719 /// Transcription initiation factor TFIID subunit 13 gene:ENSG00000197780 /// Homo sapiens TAF13 RNA polymerase II, TATA box binding protein (TBP)-associated factor, 18kDa, mRNA (cDNA clone MGC:150596 IMAGE:40123243), complete cds. /// Homo sapiens TAF13 RNA polymerase II, TATA box binding protein (TBP)-associated factor, 18kDa (TAF13), mRNA.	TAF13 RNA polymerase II, TATA box binding protein (TBP)-associated factor, 18kDa	0.000303924	-1.237284517

ZNF721	zinc finger protein 721 gene:ENSG00000182903 /// Homo sapiens cDNA clone IMAGE:3882186, partial cds. /// Homo sapiens zinc finger protein 721 (ZNF721), mRNA.	ATP-binding cassette, sub-family A (ABC1), member 11 (pseudogene) /// zinc finger protein 721	0.000544553	-1.208580354
ZNF121	Zinc finger protein 121 gene:ENSG00000197961 /// Homo sapiens cDNA FLJ59039 complete cds, highly similar to Homo sapiens zinc finger protein 121 (ZNF121), mRNA. /// Homo sapiens zinc finger protein 121 (ZNF121), mRNA.	zinc finger protein 121	0.000947076	-1.196408101
ZNF682	Isoform 1 of Zinc finger protein 682 gene:ENSG00000197124 /// zinc finger protein 682 isoform 2 gene:ENSG00000197124 /// BC39498_2 gene:ENSG00000197124 /// Homo sapiens cDNA FLJ40845 fis, clone TRACH2014483, highly similar to Zinc finger protein 682. /// Homo sapiens zinc finger protein 682 (ZNF682), transcript variant 1, mRNA. /// Homo sapiens zinc finger protein 682 (ZNF682), transcript variant 2, mRNA.	zinc finger protein 682	1.03864E-08	-1.503956443
ZNF542	Zinc finger protein 542 gene:ENSG00000197025 /// Homo sapiens zinc finger protein 542, mRNA (cDNA clone MGC:105006 IMAGE:3078727), complete cds. /// Homo sapiens zinc finger protein 542 (ZNF542), non-coding RNA. /// Homo sapiens zinc finger protein 542 (ZNF542), non-coding RNA. /// Homo sapiens zinc finger protein 542 (ZNF542), non-coding RNA. /// Homo sapiens zinc finger protein 542 (ZNF542), non-coding RNA.	zinc finger protein 542	0.006894357	-1.196802555

NFE2L3	Nuclear factor erythroid 2-related factor 3 gene:ENSG00000050344 /// Homo sapiens nuclear factor (erythroid-derived 2)-like 3, mRNA (cDNA clone MGC:87043 IMAGE:30347127), complete cds. /// Homo sapiens nuclear factor (erythroid-derived 2)-like 3 (NFE2L3), mRNA.	nuclear factor (erythroid-derived 2)-like 3	0.000711581	1.257435284
PTOV1	Isoform 2 of Prostate tumor overexpressed gene 1 protein gene:ENSG00000104960 /// Isoform 1 of Prostate tumor overexpressed gene 1 protein gene:ENSG00000104960 /// Homo sapiens cDNA FLJ25088 fis, clone CBL08845, highly similar to Homo sapiens PTOV1 (PTOV1) gene. /// Homo sapiens prostate tumor overexpressed 1 (PTOV1), mRNA.	prostate tumor overexpressed 1	0.00280995	1.191462401
LOC339742	full-length cDNA clone CS0DF033YF19 of Fetal brain of Homo sapiens (human). /// PREDICTED: Homo sapiens hypothetical protein LOC100134157 (LOC100134157), mRNA. /// PREDICTED: Homo sapiens similar to LOC339742 protein (LOC100134240), mRNA. /// PREDICTED: Homo sapiens hypothetical protein LOC339742 (LOC339742), mRNA.	hypothetical protein LOC100134157 /// similar to LOC339742 protein /// hypothetical protein LOC339742	0.009613021	-1.206233407
ZFP36L1	Butyrate response factor 1 gene:ENSG00000185650 /// Homo sapiens cDNA FLJ14140 fis, clone MAMMA1002858, highly similar to Butyrate response factor 1. /// Homo sapiens zinc finger protein 36, C3H type-like 1 (ZFP36L1), mRNA.	zinc finger protein 36, C3H type-like 1	0.008789807	1.143767204

JMJD1A	cdna:known chromosome:NCBI36:2:86521435:86573350:1 gene:ENSG00000115548 /// JmjC domain-containing histone demethylation protein 2A gene:ENSG00000115548 /// cdna:known chromosome:NCBI36:2:86522095:86573350:1 gene:ENSG00000115548 /// Homo sapiens mRNA; cDNA DKFZp686B22109 (from clone DKFZp686B22109). /// Homo sapiens jumonji domain containing 1A (JMJD1A), mRNA.	jumonji domain containing 1A	0.008568719	1.175489124
FOXG1	Forkhead box protein G1 gene:ENSG00000176165 /// Forkhead box protein G1 gene:ENSG00000176165 /// Homo sapiens forkhead box G1, mRNA (cDNA clone MGC:48322 IMAGE:5284335), complete cds. /// Homo sapiens forkhead box G1 (FOXG1), mRNA.	forkhead box G1	0.001909934	-1.280015942
ZNF383	Zinc finger protein 383 gene:ENSG00000188283 /// Zinc finger protein 383 gene:ENSG00000188283 /// Homo sapiens zinc finger protein 383 mRNA, complete cds. /// Homo sapiens zinc finger protein 383 (ZNF383), mRNA.	zinc finger protein 383	1.82504E-05	-1.373579211
ZNF600	Zinc finger protein 600 gene:ENSG00000189190 /// 14 kDa protein gene:ENSG00000189190 /// Human zinc finger protein (kr-znf1) mRNA, partial cds. /// Homo sapiens zinc finger protein 600 (ZNF600), mRNA.	zinc finger protein 600	0.000129022	-1.335911933

TES	Isoform 1 of Testin gene:ENSG00000135269 /// Isoform 2 of Testin gene:ENSG00000135269 /// 48 kDa protein gene:ENSG00000135269 /// Isoform 2 of Testin gene:ENSG00000135269 /// Homo sapiens TESTIN 2 mRNA, complete cds. /// Homo sapiens testis derived transcript (3 LIM domains) (TES), transcript variant 2, mRNA. /// Homo sapiens testis derived transcript (3 LIM domains) (TES), transcript variant 1, mRNA.	testis derived transcript (3 LIM domains)	0.003543198	-1.142458099
CHN1	cdna:known chromosome:NCBI36:2:175372337:175578200:-1 gene:ENSG00000128656 /// Isoform Alpha-2 of N-chimaerin gene:ENSG00000128656 /// Chimerin (Chimaerin) 1 variant (Fragment) gene:ENSG00000128656 /// cdna:known chromosome:NCBI36:2:175372466:175420523:-1 gene:ENSG00000128656 /// cdna:known chromosome:NCBI36:2:175372489:175419379:-1 gene:ENSG00000128656 /// cdna:known chromosome:NCBI36:2:175372963:175578343:-1 gene:ENSG00000128656 /// Homo sapiens cDNA FLJ76755 complete cds, highly similar to Homo sapiens chimerin (chimaerin) 1 (CHN1), mRNA. /// Homo sapiens chimerin (chimaerin) 1 (CHN1), transcript variant 2, mRNA. /// Homo sapiens chimerin (chimaerin) 1 (CHN1), transcript variant 1, mRNA.	chimerin (chimaerin) 1	1.11385E-05	1.329098757
POLR2G	DNA-directed RNA polymerase II subunit RPB7 gene:ENSG00000168002 /// Human RNA polymerase II subunit hsRPB7 mRNA, complete cds. /// Homo sapiens polymerase (RNA) II (DNA directed) polypeptide G (POLR2G), mRNA.	polymerase (RNA) II (DNA directed) polypeptide G	0.001797464	1.27458602

POLR1E	Isoform 2 of DNA-directed RNA polymerase I subunit RPA49 gene:ENSG00000137054 /// Isoform 1 of DNA-directed RNA polymerase I subunit RPA49 gene:ENSG00000137054 /// Homo sapiens polymerase (RNA) I polypeptide E, 53kDa, mRNA (cDNA clone MGC:5609 IMAGE:3461710), complete cds. /// Homo sapiens polymerase (RNA) I polypeptide E, 53kDa (POLR1E), mRNA.	polymerase (RNA) I polypeptide E, 53kDa	0.005571166	1.163774111
OSGEPL1	Isoform 3 of Probable O-sialoglycoprotein endopeptidase 2 gene:ENSG00000128694 /// Homo sapiens O-sialoglycoprotein endopeptidase-like 1, mRNA (cDNA clone MGC:20293 IMAGE:4121450), complete cds. /// Homo sapiens O-sialoglycoprotein endopeptidase-like 1 (OSGEPL1), mRNA.	O-sialoglycoprotein endopeptidase-like 1	0.007468574	1.235325034
ZMYM3	Isoform 2 of Zinc finger MYM-type protein 3 gene:ENSG00000147130 /// Isoform 1 of Zinc finger MYM-type protein 3 gene:ENSG00000147130 /// Isoform 1 of Zinc finger MYM-type protein 3 gene:ENSG00000147130 /// Putative uncharacterized protein ZMYM3 gene:ENSG00000147130 /// Putative uncharacterized protein ZMYM3 gene:ENSG00000147130 /// Putative uncharacterized protein ZMYM3 gene:ENSG00000147130 /// Zinc finger protein 261 gene:ENSG00000147130 /// Putative uncharacterized protein ZMYM3 gene:ENSG00000147130 /// H.sapiens mRNA for protein encoded by a candidate gene, DXS6673E, for mental retardation. /// Homo sapiens zinc finger, MYM-type 3 (ZMYM3), transcript variant 1, mRNA. /// Homo sapiens zinc finger, MYM-type 3 (ZMYM3), transcript variant 2, mRNA.	zinc finger, MYM-type 3	0.002351868	1.173522542

PRICKLE1	Prickle-like protein 1 gene:ENSG00000139174 /// Homo sapiens REST/NRSF-interacting lim domain protein (RILP) mRNA, complete cds. /// Homo sapiens prickle homolog 1 (Drosophila) (PRICKLE1), mRNA.	prickle homolog 1 (Drosophila)	7.83665E-09	1.620483371
RPS27L	40S ribosomal protein S27-like protein gene:ENSG00000185088 /// 13 kDa protein gene:ENSG00000185088 /// Homo sapiens ribosomal protein S27-like, mRNA (cDNA clone MGC:12175 IMAGE:3827362), complete cds. /// Homo sapiens ribosomal protein S27-like (RPS27L), mRNA.	ribosomal protein S27-like	0.001063446	-1.254328897
RNF43	RING finger protein 43 gene:ENSG00000108375 /// RING finger protein 43 gene:ENSG00000108375 /// Homo sapiens cDNA FLJ77466 complete cds. /// Homo sapiens ring finger protein 43 (RNF43), mRNA.	ring finger protein 43	0.000262242	-1.403402441
RNF26	RING finger protein 26 gene:ENSG00000173456 /// Homo sapiens ring finger protein 26, mRNA (cDNA clone MGC:2642 IMAGE:3507662), complete cds. /// Homo sapiens ring finger protein 26 (RNF26), mRNA.	ring finger protein 26	0.002465445	1.192581632
RNF138	Isoform 1 of E3 ubiquitin-protein ligase RNF138 gene:ENSG00000134758 /// Isoform 2 of E3 ubiquitin-protein ligase RNF138 gene:ENSG00000134758 /// Isoform 1 of E3 ubiquitin-protein ligase RNF138 gene:ENSG00000134758 /// Homo sapiens ring finger protein 138, mRNA (cDNA clone MGC:8758 IMAGE:3914953), complete cds. /// Homo sapiens ring finger protein 138 (RNF138), transcript variant 1, mRNA. /// Homo sapiens ring finger protein 138 (RNF138), transcript variant 2, mRNA.	ring finger protein 138	0.005227981	1.167172517

PLEKHF1	cDNA FLJ55258, highly similar to Homo sapiens pleckstrin homology domain containing, family F member 1, mRNA gene:ENSG00000166289 /// Homo sapiens apoptosis-inducing protein (APPD) mRNA, complete cds. /// Homo sapiens pleckstrin homology domain containing, family F (with FYVE domain) member 1 (PLEKHF1), mRNA.	pleckstrin homology domain containing, family F (with FYVE domain) member 1	0.002726793	-1.207098368
ZMAT4	Isoform 1 of Zinc finger matrin-type protein 4 gene:ENSG00000165061 /// Putative uncharacterized protein ZMAT4 gene:ENSG00000165061 /// Isoform 2 of Zinc finger matrin-type protein 4 gene:ENSG00000165061 /// Homo sapiens zinc finger, matrin type 4, mRNA (cDNA clone MGC:24845 IMAGE:4934088), complete cds. /// Homo sapiens zinc finger, matrin type 4 (ZMAT4), transcript variant 1, mRNA. /// Homo sapiens zinc finger, matrin type 4 (ZMAT4), transcript variant 2, mRNA.	zinc finger, matrin type 4	0.003094951	-1.227431955
TP53INP1	Isoform 1 of Tumor protein p53-inducible nuclear protein 1 gene:ENSG00000164938 /// Isoform 2 of Tumor protein p53-inducible nuclear protein 1 gene:ENSG00000164938 /// Homo sapiens p53-induced protein SIP18 mRNA, complete cds, alternatively spliced. /// Homo sapiens tumor protein p53 inducible nuclear protein 1 (TP53INP1), transcript variant 1, mRNA. /// Homo sapiens tumor protein p53 inducible nuclear protein 1 (TP53INP1), transcript variant 2, mRNA.	tumor protein p53 inducible nuclear protein 1	0.003404145	-1.184735098

ZFAND2A	cDNA FLJ76351, highly similar to Homo sapiens zinc finger, AN1-type domain 2A (ZFAND2A), mRNA gene:ENSG00000178381 /// Putative uncharacterized protein ZFAND2A gene:ENSG00000178381 /// AN1-type zinc finger protein 2A gene:ENSG00000178381 /// Homo sapiens zinc finger, AN1-type domain 2A, mRNA (cDNA clone MGC:39501 IMAGE:5263788), complete cds. /// Homo sapiens zinc finger, AN1-type domain 2A (ZFAND2A), mRNA.	zinc finger, AN1-type domain 2A	0.000191425	-1.355119998
NOB1	RNA-binding protein NOB1 gene:ENSG00000141101 /// Homo sapiens RNA-binding protein NOB1 mRNA, complete cds. /// Homo sapiens NIN1/RPN12 binding protein 1 homolog (S. cerevisiae) (NOB1), mRNA.	NIN1/RPN12 binding protein 1 homolog (S. cerevisiae)	0.003266145	1.19290308
ZNF808	Isoform 2 of Zinc finger protein 808 gene:ENSG00000198482 /// Homo sapiens zinc finger protein 808, mRNA (cDNA clone IMAGE:3542548), complete cds. /// Homo sapiens zinc finger protein 808 (ZNF808), mRNA.	zinc finger protein 808	0.000347744	-1.234566821
SNAI3	Zinc finger protein SNAI3 gene:ENSG00000185669 /// Homo sapiens snail homolog 3 (Drosophila), mRNA (cDNA clone MGC:129606 IMAGE:40005947), complete cds. /// Homo sapiens snail homolog 3 (Drosophila) (SNAI3), mRNA.	snail homolog 3 (Drosophila)	0.002000567	-1.227756685
LOC91431	Isoform 1 of Uncharacterized protein FLJ44066 gene:ENSG00000188014 /// Homo sapiens mRNA; cDNA DKFZp313L226 (from clone DKFZp313L226). /// Homo sapiens prematurely terminated mRNA decay factor-like (LOC91431), mRNA.	prematurely terminated mRNA decay factor-like	0.000309304	-1.283344003

RNF170	Isoform 3 of RING finger protein 170 gene:ENSG00000120925 /// 37 kDa protein gene:ENSG00000120925 /// Isoform 4 of RING finger protein 170 gene:ENSG00000120925 /// Isoform 1 of RING finger protein 170 gene:ENSG00000120925 /// Homo sapiens cDNA FLJ33545 fis, clone BRAMY2008382. /// Homo sapiens ring finger protein 170 (RNF170), mRNA.	ring finger protein 170	0.003313338	1.229727891
SLC39A11	Isoform 1 of Zinc transporter ZIP11 gene:ENSG00000133195 /// Isoform 2 of Zinc transporter ZIP11 gene:ENSG00000133195 /// Homo sapiens solute carrier family 39 (metal ion transporter), member 11, mRNA (cDNA clone MGC:45399 IMAGE:5219031), complete cds. /// Homo sapiens solute carrier family 39 (metal ion transporter), member 11 (SLC39A11), mRNA.	solute carrier family 39 (metal ion transporter), member 11	0.009180741	1.21952808
ZNF654	Zinc finger protein 654 gene:ENSG00000175105 /// Homo sapiens zinc finger protein 654 (ZNF654), mRNA.	zinc finger protein 654	2.52882E-05	-1.39790768
RNF168	RING finger protein 168 gene:ENSG00000163961 /// Homo sapiens ring finger protein 168, mRNA (cDNA clone MGC:45398 IMAGE:5163887), complete cds. /// Homo sapiens ring finger protein 168 (RNF168), mRNA.	ring finger protein 168	0.004412193	-1.155658324
**Functional Group 39**	**Transcript Description**	**Gene Title**	**Bayes.lnp**	**Fold**

PRKG1	cGMP-dependent protein kinase 1, alpha isozyme gene:ENSG00000185532 /// cGMP-dependent protein kinase 1, alpha isozyme gene:ENSG00000185532 /// cGMP-dependent protein kinase 1, beta isozyme gene:ENSG00000185532 /// Protein kinase, cGMP-dependent, type I gene:ENSG00000185532 /// Protein kinase, cGMP-dependent, type I gene:ENSG00000185532 /// Protein kinase, cGMP-dependent, type I gene:ENSG00000185532 /// Homo sapiens protein kinase, cGMP-dependent, type I, mRNA (cDNA clone MGC:150609 IMAGE:40123332), complete cds. /// Homo sapiens protein kinase, cGMP-dependent, type I (PRKG1), transcript variant 1, mRNA. /// Homo sapiens protein kinase, cGMP-dependent, type I (PRKG1), transcript variant 2, mRNA.	protein kinase, cGMP-dependent, type I	0.006091131	-1.26106844
KATNAL2	40 kDa protein gene:ENSG00000167216 /// Isoform 2 of Katanin p60 ATPase-containing subunit A-like 2 gene:ENSG00000167216 /// Homo sapiens katanin p60 subunit A-like 2, mRNA (cDNA clone MGC:33211 IMAGE:4829800), complete cds. /// Homo sapiens katanin p60 subunit A-like 2 (KATNAL2), mRNA.	katanin p60 subunit A-like 2	0.006896265	-1.186175082
KIT	Mast/stem cell growth factor receptor gene:ENSG00000157404 /// Homo sapiens v-kit Hardy-Zuckerman 4 feline sarcoma viral oncogene homolog, mRNA (cDNA clone MGC:87427 IMAGE:4375615), complete cds. /// Homo sapiens v-kit Hardy-Zuckerman 4 feline sarcoma viral oncogene homolog (KIT), transcript variant 1, mRNA. /// Homo sapiens v-kit Hardy-Zuckerman 4 feline sarcoma viral oncogene homolog (KIT), transcript variant 2, mRNA.	v-kit Hardy-Zuckerman 4 feline sarcoma viral oncogene homolog	0.001851755	1.266088114

PIM2	Serine/threonine-protein kinase Pim-2 gene:ENSG00000102096 /// Homo sapiens pim-2 oncogene, mRNA (cDNA clone MGC:8925 IMAGE:3913552), complete cds. /// Homo sapiens pim-2 oncogene (PIM2), mRNA.	pim-2 oncogene	0.005706315	1.165492577
FGFR2	Isoform 17 of Fibroblast growth factor receptor 2 gene:ENSG00000066468 /// fibroblast growth factor receptor 2 isoform 2 precursor gene:ENSG00000066468 /// fibroblast growth factor receptor 2 isoform 1 precursor gene:ENSG00000066468 /// Isoform 16 of Fibroblast growth factor receptor 2 gene:ENSG00000066468 /// Isoform 1 of Fibroblast growth factor receptor 2 gene:ENSG00000066468 /// Isoform 18 of Fibroblast growth factor receptor 2 gene:ENSG00000066468 /// Isoform of FGFR2 gene:ENSG00000066468 /// Isoform 15 of Fibroblast growth factor receptor 2 gene:ENSG00000066468 /// Isoform 20 of Fibroblast growth factor receptor 2 gene:ENSG00000066468 /// 79 kDa protein gene:ENSG00000066468 /// Isoform 5 of Fibroblast growth factor receptor 2 gene:ENSG00000066468 /// fibroblast growth factor receptor 2 isoform 2 precursor gene:ENSG00000066468 /// Isoform 6 of Fibroblast growth factor receptor 2 gene:ENSG00000066468 /// Isoform 18 of Fibroblast growth factor receptor 2 gene:ENSG00000066468 /// Isoform 17 of Fibroblast growth factor receptor 2 gene:ENSG00000066468 /// Isoform 14 of Fibroblast growth factor receptor 2 gene:ENSG00000066468 /// Human fibroblast growth factor receptor (K-sam) mRNA, complete cds. /// Homo sapiens fibroblast growth factor receptor 2 (FGFR2),	fibroblast growth factor receptor 2	0.000420397	1.229409481

	transcript variant 2, mRNA. /// Homo sapiens fibroblast growth factor receptor 2 (FGFR2), transcript variant 1, mRNA.			

FGFR1	Isoform 1 of Basic fibroblast growth factor receptor 1 gene:ENSG00000077782 /// Isoform 2 of Basic fibroblast growth factor receptor 1 gene:ENSG00000077782 /// Isoform 6 of Basic fibroblast growth factor receptor 1 gene:ENSG00000077782 /// Isoform 14 of Basic fibroblast growth factor receptor 1 gene:ENSG00000077782 /// Isoform 14 of Basic fibroblast growth factor receptor 1 gene:ENSG00000077782 /// Isoform 4 of Basic fibroblast growth factor receptor 1 gene:ENSG00000077782 /// Isoform 8 of Basic fibroblast growth factor receptor 1 gene:ENSG00000077782 /// Isoform 15 of Basic fibroblast growth factor receptor 1 gene:ENSG00000077782 /// Fibroblast growth factor receptor 1 THIRD immunoglobulin domain, FGFR 1 IG domain III gene:ENSG00000077782 /// Isoform 16 of Basic fibroblast growth factor receptor 1 gene:ENSG00000077782 /// Isoform 3 of Basic fibroblast growth factor receptor 1 gene:ENSG00000077782 /// Homo sapiens fibroblast growth factor receptor 1, mRNA (cDNA clone MGC:9218 IMAGE:3896359), complete cds. /// Homo sapiens fibroblast growth factor receptor 1 (FGFR1), transcript variant 2, mRNA. /// Homo sapiens fibroblast growth factor receptor 1 (FGFR1), transcript variant 3, mRNA. /// Homo sapiens fibroblast growth factor receptor 1 (FGFR1), transcript variant 4, mRNA. /// Homo sapiens fibroblast growth factor receptor 1 (FGFR1), transcript variant 1, mRNA. /// Homo sapiens fibroblast growth factor receptor 1 (FGFR1), transcript variant 5, mRNA. /// Homo sapiens fibroblast growth factor receptor 1 (FGFR1), transcript variant 6, mRNA. /// Homo sapiens	fibroblast growth factor receptor 1	0.000142 033	1.291247 488

	fibroblast growth factor receptor 1 (FGFR1), transcript variant 9, mRNA.			
TGFBR2	Isoform 2 of TGF-beta receptor type-2 gene:ENSG00000163513 /// Isoform 1 of TGF-beta receptor type-2 gene:ENSG00000163513 /// Transforming growth factor beta receptor type IIC gene:ENSG00000163513 /// Homo sapiens cDNA FLJ53920 complete cds, highly similar to TGF-beta receptor type-2 precursor (EC 2.7.11.30). /// Homo sapiens transforming growth factor, beta receptor II (70/80kDa) (TGFBR2), transcript variant 1, mRNA. /// Homo sapiens transforming growth factor, beta receptor II (70/80kDa) (TGFBR2), transcript variant 2, mRNA.	transforming growth factor, beta receptor II (70/80kDa)	0.001114476	1.270866649

DAPK3	Death-associated protein kinase 3 gene:ENSG00000167657 /// Homo sapiens cDNA FLJ14684 fis, clone NT2RP2004933, highly similar to Death-associated protein kinase 3 (EC 2.7.11.1). /// Homo sapiens death-associated protein kinase 3 (DAPK3), mRNA.	death-associated protein kinase 3	0.002658432	-1.192977254
UCK2	Isoform 1 of Uridine-cytidine kinase 2 gene:ENSG00000143179 /// Homo sapiens uridine-cytidine kinase 2, mRNA (cDNA clone MGC:10318 IMAGE:3940564), complete cds. /// Homo sapiens uridine-cytidine kinase 2 (UCK2), mRNA.	uridine-cytidine kinase 2	0.001546214	1.170690178
IGF1R	Insulin-like growth factor 1 receptor gene:ENSG00000140443 /// Homo sapiens IGF1R mRNA for insulin-like growth factor 1 receptor, partial cds. /// Homo sapiens insulin-like growth factor 1 receptor (IGF1R), mRNA.	insulin-like growth factor 1 receptor	0.955932191	1.001369574
HELZ	Probable helicase with zinc finger domain gene:ENSG00000198265 /// Homo sapiens helicase with zinc finger, mRNA (cDNA clone MGC:163454 IMAGE:40146613), complete cds. /// Homo sapiens helicase with zinc finger (HELZ), mRNA.	helicase with zinc finger	1.11093E-07	-1.384436629
KDR	Vascular endothelial growth factor receptor 2 gene:ENSG00000128052 /// Homo sapiens KDR/flk-1 protein mRNA, complete cds. /// Homo sapiens kinase insert domain receptor (a type III receptor tyrosine kinase) (KDR), mRNA.	kinase insert domain receptor (a type III receptor tyrosine kinase)	0.002021804	-1.176761484
TYRO3	Tyrosine-protein kinase receptor TYRO3 gene:ENSG00000092445 /// Homo sapiens mRNA for Sky, complete cds. /// Homo sapiens TYRO3 protein tyrosine kinase (TYRO3), mRNA.	TYRO3 protein tyrosine kinase	0.000354804	1.231611604

JAK1	Tyrosine-protein kinase JAK1 gene:ENSG00000162434 /// Homo sapiens Janus kinase 1 (a protein tyrosine kinase), mRNA (cDNA clone MGC:164360 IMAGE:40146751), complete cds. /// Homo sapiens Janus kinase 1 (a protein tyrosine kinase) (JAK1), mRNA.	Janus kinase 1 (a protein tyrosine kinase)	0.00451496	-1.142309634
AURKB	Serine/threonine-protein kinase 12 gene:ENSG00000178999 /// Homo sapiens aurora kinase B, mRNA (cDNA clone MGC:8406 IMAGE:2820719), complete cds. /// Homo sapiens aurora kinase B (AURKB), mRNA.	aurora kinase B	0.000561556	1.227580402
DDX43	Probable ATP-dependent RNA helicase DDX43 gene:ENSG00000080007 /// Homo sapiens mRNA for DEAD-box protein (HAGE gene). /// Homo sapiens DEAD (Asp-Glu-Ala-Asp) box polypeptide 43 (DDX43), mRNA.	DEAD (Asp-Glu-Ala-Asp) box polypeptide 43	2.80542E-06	-1.812446668
HSPA1B	Heat shock 70 kDa protein 1 gene:ENSG00000215292 /// cDNA FLJ54389, highly similar to Heat shock 70 kDa protein 1 gene:ENSG00000215292 /// cDNA FLJ54392, highly similar to Heat shock 70 kDa protein 1 gene:ENSG00000215328 /// cDNA FLJ54389, highly similar to Heat shock 70 kDa protein 1 gene:ENSG00000215328 /// cDNA FLJ54389, highly similar to Heat shock 70 kDa protein 1 gene:ENSG00000204389 /// Heat shock 70 kDa protein 1 gene:ENSG00000204389 /// Homo sapiens cDNA FLJ54328 complete cds, highly similar to Heat shock 70 kDa protein 1. /// Homo sapiens heat shock 70kDa protein 1A, mRNA (cDNA clone MGC:1309 IMAGE:3345864), complete cds. /// Homo sapiens heat shock 70kDa protein 1A (HSPA1A), mRNA.	heat shock 70kDa protein 1B /// heat shock 70kDa protein 1A	2.08E-10	-1.825669476

INSR	insulin receptor isoform Short precursor gene:ENSG00000171105 /// Insulin receptor precursor gene:ENSG00000171105 /// Human insulin receptor mRNA, complete cds. /// Homo sapiens insulin receptor (INSR), transcript variant 1, mRNA. /// Homo sapiens insulin receptor (INSR), transcript variant 2, mRNA.	insulin receptor	0.007080331	-1.17113768
CDC42BPG	Serine/threonine-protein kinase MRCK gamma gene:ENSG00000171219 /// Homo sapiens myotonic dystrophy kinase-related CDC42-binding kinase gamma mRNA, complete cds. /// Homo sapiens CDC42 binding protein kinase gamma (DMPK-like) (CDC42BPG), mRNA.	CDC42 binding protein kinase gamma (DMPK-like)	0.001831502	-1.274942688
PRKD3	Isoform 1 of Serine/threonine-protein kinase D3 gene:ENSG00000115825 /// Isoform 1 of Serine/threonine-protein kinase D3 gene:ENSG00000115825 /// Homo sapiens EPK2 mRNA for serine/threonine kinase, complete cds. /// Homo sapiens protein kinase D3 (PRKD3), mRNA.	protein kinase D3	0.009198204	1.286906608
PRKAG1	cDNA FLJ40287 fis, clone TESTI2027909, highly similar to 5'-AMP-ACTIVATED PROTEIN KINASE, GAMMA-1 SUBUNIT gene:ENSG00000181929 /// 5'-AMP-activated protein kinase subunit gamma-1 gene:ENSG00000181929 /// Homo sapiens protein kinase, AMP-activated, gamma 1 non-catalytic subunit, mRNA (cDNA clone MGC:8666 IMAGE:2964434), complete cds. /// Homo sapiens protein kinase, AMP-activated, gamma 1 non-catalytic subunit (PRKAG1), transcript variant 1, mRNA. /// Homo sapiens protein kinase, AMP-activated, gamma 1 non-catalytic subunit (PRKAG1), transcript variant 2, mRNA.	protein kinase, AMP-activated, gamma 1 non-catalytic subunit	0.00035763	1.212655185

CSNK1G1	Isoform 1L of Casein kinase I isoform gamma-1 gene:ENSG00000169118 /// Isoform 1S of Casein kinase I isoform gamma-1 gene:ENSG00000169118 /// Homo sapiens casein kinase 1, gamma 1, mRNA (cDNA clone MGC:163485 IMAGE:40146644), complete cds. /// Homo sapiens casein kinase 1, gamma 1 (CSNK1G1), mRNA.	casein kinase 1, gamma 1	2.72735E-05	-1.352555123
EIF2AK3	eukaryotic translation initiation factor 2-alpha kinase 3 gene:ENSG00000172071 /// Homo sapiens eukaryotic translation initiation factor 2 alpha kinase PEK mRNA, complete cds. /// Homo sapiens eukaryotic translation initiation factor 2-alpha kinase 3 (EIF2AK3), mRNA.	eukaryotic translation initiation factor 2-alpha kinase 3	0.001124391	-1.22830076
PFKM	cDNA FLJ44241 fis, clone THYMU3008436, highly similar to 6-phosphofructokinase, muscle type gene:ENSG00000152556 /// Isoform 1 of 6-phosphofructokinase, muscle type gene:ENSG00000152556 /// Isoform 2 of 6-phosphofructokinase, muscle type gene:ENSG00000152556 /// Homo sapiens cDNA FLJ44241 fis, clone THYMU3008436, highly similar to 6-phosphofructokinase, muscle type (EC 2.7.1.11). /// Homo sapiens phosphofructokinase, muscle (PFKM), mRNA.	phosphofructokinase, muscle	0.008182424	1.16915528
HSPA2	Heat shock-related 70 kDa protein 2 gene:ENSG00000126803 /// Heat shock-related 70 kDa protein 2 gene:ENSG00000126803 /// Homo sapiens heat shock 70kDa protein 2, mRNA (cDNA clone MGC:33922 IMAGE:5276121), complete cds. /// Homo sapiens heat shock 70kDa protein 2 (HSPA2), mRNA.	heat shock 70kDa protein 2	0.006917989	-1.155762729

CDC2	cell division cycle 2 protein isoform 1 gene:ENSG00000170312 /// cell division cycle 2 protein isoform 1 gene:ENSG00000170312 /// Isoform 2 of Cell division control protein 2 homolog gene:ENSG00000170312 /// Putative uncharacterized protein DKFZp686L20222 gene:ENSG00000170312 /// Isoform 2 of Cell division control protein 2 homolog gene:ENSG00000170312 /// Homo sapiens cell division cycle 2, G1 to S and G2 to M, mRNA (cDNA clone IMAGE:6710672). /// Homo sapiens cell division cycle 2, G1 to S and G2 to M (CDC2), transcript variant 2, mRNA. /// Homo sapiens cell division cycle 2, G1 to S and G2 to M (CDC2), transcript variant 3, mRNA. /// Homo sapiens cell division cycle 2, G1 to S and G2 to M (CDC2), transcript variant 1, mRNA.	cell division cycle 2, G1 to S and G2 to M	0.008860211	1.220305846
NUAK2	cDNA FLJ56013, highly similar to NUAK family SNF1-like kinase 2 gene:ENSG00000163545 /// Homo sapiens cDNA FLJ56013 complete cds, highly similar to NUAK family SNF1-like kinase 2 (EC 2.7.11.1). /// Homo sapiens NUAK family, SNF1-like kinase, 2 (NUAK2), mRNA.	NUAK family, SNF1-like kinase, 2	0.00355275	-1.223639628
PLK2	Serine/threonine-protein kinase PLK2 gene:ENSG00000145632 /// Homo sapiens serum-inducible kinase (SNK) mRNA, complete cds. /// Homo sapiens polo-like kinase 2 (Drosophila) (PLK2), mRNA.	polo-like kinase 2 (Drosophila)	0.001472681	-1.23023176
HSPA7	Heat shock 70 kDa protein 6 gene:ENSG00000173110 /// Homo sapiens heat shock 70kDa protein 6 (HSP70B'), mRNA (cDNA clone MGC:46216 IMAGE:5723718), complete cds. /// Homo sapiens heat shock 70kDa protein 6 (HSP70B') (HSPA6), mRNA. /// Homo sapiens heat shock 70kDa protein 7 (HSP70B) (HSPA7), non-coding RNA.	heat shock 70kDa protein 6 (HSP70B') /// heat shock 70kDa protein 7 (HSP70B)	8.65E-05	-1.420883396

DQX1	Isoform 1 of ATP-dependent RNA helicase DQX1 gene:ENSG00000144045 /// Isoform 1 of ATP-dependent RNA helicase DQX1 gene:ENSG00000144045 /// Isoform 2 of ATP-dependent RNA helicase DQX1 gene:ENSG00000144045 /// Homo sapiens DEAQ box polypeptide 1 (RNA-dependent ATPase), mRNA (cDNA clone MGC:104000 IMAGE:30915408), complete cds. /// Homo sapiens DEAQ box polypeptide 1 (RNA-dependent ATPase) (DQX1), mRNA.	DEAQ box polypeptide 1 (RNA-dependent ATPase)	1.18422E-05	-1.604766031
MAPKAPK2	Isoform 1 of MAP kinase-activated protein kinase 2 gene:ENSG00000162889 /// Isoform 2 of MAP kinase-activated protein kinase 2 gene:ENSG00000162889 /// Homo sapiens mitogen-activated protein kinase-activated protein kinase 2, mRNA (cDNA clone MGC:59706 IMAGE:6267183), complete cds. /// Homo sapiens mitogen-activated protein kinase-activated protein kinase 2 (MAPKAPK2), transcript variant 1, mRNA. /// Homo sapiens mitogen-activated protein kinase-activated protein kinase 2 (MAPKAPK2), transcript variant 2, mRNA.	mitogen-activated protein kinase-activated protein kinase 2	0.008088491	1.14559268
MAPKAPK3	MAP kinase-activated protein kinase 3 gene:ENSG00000114738 /// Homo sapiens MAPKAP kinase (3pK) mRNA, complete cds. /// Homo sapiens mitogen-activated protein kinase-activated protein kinase 3 (MAPKAPK3), mRNA.	mitogen-activated protein kinase-activated protein kinase 3	0.001820874	1.193094365
EPHB1	Isoform 1 of Ephrin type-B receptor 1 gene:ENSG00000154928 /// Isoform 4 of Ephrin type-B receptor 1 gene:ENSG00000154928 /// Homo sapiens (clone FBK III 16) protein tyrosine kinase (NET PTK) mRNA, complete cds. /// Homo sapiens EPH receptor B1 (EPHB1), mRNA.	EPH receptor B1	0.000880803	-1.270478492

EPHA2	ephrin receptor EphA2 gene:ENSG00000142627 /// ephrin receptor EphA2 gene:ENSG00000142627 /// Homo sapiens EPH receptor A2, mRNA (cDNA clone MGC:29717 IMAGE:5090654), complete cds. /// Homo sapiens EPH receptor A2 (EPHA2), mRNA.	EPH receptor A2	3.16267E-05	-1.358836438
SEPHS1	Selenophosphate synthetase 1 gene:ENSG00000086475 /// Selenide, water dikinase 1 gene:ENSG00000086475 /// Selenophosphate synthetase 1 gene:ENSG00000086475 /// Homo sapiens selenophosphate synthetase 1, mRNA (cDNA clone MGC:74471 IMAGE:6186560), complete cds. /// Homo sapiens selenophosphate synthetase 1 (SEPHS1), mRNA.	selenophosphate synthetase 1	0.004247486	1.150551013
PLK1	Serine/threonine-protein kinase PLK1 gene:ENSG00000166851 /// Polo-like kinase 1 delta gene:ENSG00000166851 /// Homo sapiens mRNA for polo-like kinase variant protein. /// Homo sapiens polo-like kinase 1 (Drosophila) (PLK1), mRNA.	polo-like kinase 1 (Drosophila)	0.000696133	1.218092061
WEE1	Wee1-like protein kinase gene:ENSG00000166483 /// Homo sapiens WEE1 homolog (S. pombe), mRNA (cDNA clone MGC:60116 IMAGE:6147695), complete cds. /// Homo sapiens WEE1 homolog (S. pombe) (WEE1), mRNA.	WEE1 homolog (S. pombe)	0.146954219	-1.07568797
HK2	Hexokinase-2 gene:ENSG00000159399 /// cdna:known chromosome:NCBI36:2:74915805:74973994:1 gene:ENSG00000159399 /// Homo sapiens hexokinase 2, mRNA (cDNA clone MGC:74789 IMAGE:5586773), complete cds. /// Homo sapiens hexokinase 2 (HK2), mRNA.	hexokinase 2	9.23437E-09	1.598668999

DCLK1	Isoform 3 of Serine/threonine-protein kinase DCLK1 gene:ENSG00000133083 /// Isoform 1 of Serine/threonine-protein kinase DCLK1 gene:ENSG00000133083 /// Isoform 2 of Serine/threonine-protein kinase DCLK1 gene:ENSG00000133083 /// Isoform 4 of Serine/threonine-protein kinase DCLK1 gene:ENSG00000133083 /// Doublecortin and CaM kinase-like 1 gene:ENSG00000133083 /// Homo sapiens doublecortin-like kinase 1, mRNA (cDNA clone MGC:176710 IMAGE:8862589), complete cds. /// Homo sapiens doublecortin-like kinase 1 (DCLK1), mRNA.	doublecortin-like kinase 1	3.20356E-08	-1.537273858
STK39	STE20/SPS1-related proline-alanine-rich protein kinase gene:ENSG00000198648 /// Homo sapiens Ste-20 related kinase SPAK mRNA, complete cds. /// Homo sapiens serine threonine kinase 39 (STE20/SPS1 homolog, yeast) (STK39), mRNA.	serine threonine kinase 39 (STE20/SPS1 homolog, yeast)	0.000889872	1.182857802
BMP2K	Isoform 1 of BMP-2-inducible protein kinase gene:ENSG00000138756 /// Isoform 2 of BMP-2-inducible protein kinase gene:ENSG00000138756 /// Isoform 3 of BMP-2-inducible protein kinase gene:ENSG00000138756 /// Homo sapiens mRNA; cDNA DKFZp434P0116 (from clone DKFZp434P0116). /// Homo sapiens BMP2 inducible kinase (BMP2K), transcript variant 2, mRNA. /// Homo sapiens BMP2 inducible kinase (BMP2K), transcript variant 1, mRNA.	BMP2 inducible kinase	0.008509011	-1.165971055

HIPK1	Isoform 4 of Homeodomain-interacting protein kinase 1 gene:ENSG00000163349 /// cDNA FLJ57817, highly similar to Homeodomain-interacting protein kinase 1 gene:ENSG00000163349 /// Homeodomain interacting protein kinase 1 gene:ENSG00000163349 /// Isoform 2 of Homeodomain-interacting protein kinase 1 gene:ENSG00000163349 /// cDNA FLJ57817, highly similar to Homeodomain-interacting protein kinase 1 gene:ENSG00000163349 /// Homeodomain interacting protein kinase 1 gene:ENSG00000163349 /// Isoform 3 of Homeodomain-interacting protein kinase 1 gene:ENSG00000163349 /// Isoform 4 of Homeodomain-interacting protein kinase 1 gene:ENSG00000163349 /// Isoform 4 of Homeodomain-interacting protein kinase 1 gene:ENSG00000163349 /// Homo sapiens HIPK1 mRNA for homeodomain-interacting protein kinase-1, complete cds. /// Homo sapiens homeodomain interacting protein kinase 1 (HIPK1), transcript variant 2, mRNA. /// Homo sapiens homeodomain interacting protein kinase 1 (HIPK1), transcript variant 1, mRNA. /// Homo sapiens homeodomain interacting protein kinase 1 (HIPK1), transcript variant 3, mRNA. /// Homo sapiens homeodomain interacting protein kinase 1 (HIPK1), transcript variant 4, mRNA.	homeodomain interacting protein kinase 1	0.009937791	1.150503935
PLK3	Serine/threonine-protein kinase PLK3 gene:ENSG00000173846 /// Homo sapiens mRNA for FNK serine/threonine protein kinase. /// Homo sapiens polo-like kinase 3 (Drosophila) (PLK3), mRNA.	polo-like kinase 3 (Drosophila)	0.00256224	-1.228146892

NEK3	NIMA (Never in mitosis gene a)-related kinase 3, isoform CRA_a gene:ENSG00000136098 /// NIMA (Never in mitosis gene a)-related kinase 3, isoform CRA_a gene:ENSG00000136098 /// Isoform 2 of Serine/threonine-protein kinase Nek3 gene:ENSG00000136098 /// NIMA (Never in mitosis gene a)-related kinase 3, isoform CRA_a gene:ENSG00000136098 /// Homo sapiens cDNA FLJ75370 complete cds, highly similar to Homo sapiens NIMA (never in mitosis gene a)-related kinase 3 (NEK3), transcript variant 2, mRNA. /// Homo sapiens NIMA (never in mitosis gene a)-related kinase 3 (NEK3), transcript variant 1, mRNA. /// Homo sapiens NIMA (never in mitosis gene a)-related kinase 3 (NEK3), transcript variant 2, mRNA.	NIMA (never in mitosis gene a)-related kinase 3	0.003582 46	1.187510 726
HSPH1	cDNA FLJ52364, highly similar to Heat-shock protein 105 kDa gene:ENSG00000120694 /// Isoform 3 of Heat shock protein 105 kDa gene:ENSG00000120694 /// Isoform Beta of Heat shock protein 105 kDa gene:ENSG00000120694 /// Heat shock 105kDa/110kDa protein 1 gene:ENSG00000120694 /// Homo sapiens heat shock 105kDa/110kDa protein 1, mRNA (cDNA clone MGC:40320 IMAGE:4540927), complete cds. /// Homo sapiens heat shock 105kDa/110kDa protein 1 (HSPH1), mRNA.	heat shock 105kDa/110kDa protein 1	0.000133 782	- 1.228477 359

TLK1	Isoform 2 of Serine/threonine-protein kinase tousled-like 1 gene:ENSG00000198586 /// Isoform 1 of Serine/threonine-protein kinase tousled-like 1 gene:ENSG00000198586 /// cdna:known chromosome:NCBI36:2:171558483:171631729:-1 gene:ENSG00000198586 /// Homo sapiens cDNA FLJ77613 complete cds, highly similar to Homo sapiens tousled-like kinase 1 (TLK1), mRNA. /// Homo sapiens tousled-like kinase 1 (TLK1), transcript variant 1, mRNA. /// Homo sapiens tousled-like kinase 1 (TLK1), transcript variant 2, mRNA. /// Homo sapiens tousled-like kinase 1 (TLK1), transcript variant 3, mRNA.	tousled-like kinase 1	0.009455382	1.137161626
PASK	Isoform 2 of PAS domain-containing serine/threonine-protein kinase gene:ENSG00000115687 /// Isoform 1 of PAS domain-containing serine/threonine-protein kinase gene:ENSG00000115687 /// Isoform 1 of PAS domain-containing serine/threonine-protein kinase gene:ENSG00000115687 /// PASK protein gene:ENSG00000115687 /// Homo sapiens PAS-kinase (PASK) mRNA, complete cds. /// Homo sapiens PAS domain containing serine/threonine kinase (PASK), mRNA.	PAS domain containing serine/threonine kinase	0.006873993	1.193090479
GK	#N/A	#N/A	#N/A	#N/A
STYK1	Tyrosine protein-kinase STYK1 gene:ENSG00000060140 /// Homo sapiens kinase domain protein mRNA, complete cds. /// Homo sapiens serine/threonine/tyrosine kinase 1 (STYK1), mRNA.	serine/threonine/tyrosine kinase 1	0.000979519	-1.342514754
GK3P	Homo sapiens glycerol kinase 3 pseudogene, mRNA (cDNA clone MGC:87453 IMAGE:4821051), complete cds.	glycerol kinase 3 pseudogene	0.708098968	-1.036486798

RIOK3	Isoform 1 of Serine/threonine-protein kinase RIO3 gene:ENSG00000101782 /// Homo sapiens RIO kinase 3 (yeast), mRNA (cDNA clone IMAGE:4828351). /// Homo sapiens RIO kinase 3 (yeast) (RIOK3), mRNA.	RIO kinase 3 (yeast)	0.000166833	-1.244652245
NLRP10	NACHT, LRR and PYD domains-containing protein 10 gene:ENSG00000182261 /// Homo sapiens NLR family, pyrin domain containing 10, mRNA (cDNA clone MGC:132617 IMAGE:8143960), complete cds. /// Homo sapiens NLR family, pyrin domain containing 10 (NLRP10), mRNA.	NLR family, pyrin domain containing 10	0.004550419	-1.334584327
ZRANB3	Isoform 2 of Zinc finger Ran-binding domain-containing protein 3 gene:ENSG00000121988 /// Isoform 2 of Zinc finger Ran-binding domain-containing protein 3 gene:ENSG00000121988 /// Isoform 1 of Zinc finger Ran-binding domain-containing protein 3 gene:ENSG00000121988 /// Isoform 1 of Zinc finger Ran-binding domain-containing protein 3 gene:ENSG00000121988 /// cDNA FLJ38043 fis, clone CTONG2014058, weakly similar to HepA-related protein gene:ENSG00000121988 /// Homo sapiens mRNA; cDNA DKFZp686D0128 (from clone DKFZp686D0128). /// Homo sapiens zinc finger, RAN-binding domain containing 3 (ZRANB3), mRNA.	zinc finger, RAN-binding domain containing 3	0.060927073	1.149254077
NRBP2	cDNA FLJ60460, moderately similar to Nuclear receptor-binding protein gene:ENSG00000185189 /// Homo sapiens nuclear receptor binding protein 2, mRNA (cDNA clone IMAGE:4375917). /// Homo sapiens nuclear receptor binding protein 2 (NRBP2), mRNA.	nuclear receptor binding protein 2	0.005356912	-1.224520969

HSPA6	Heat shock 70 kDa protein 6 gene:ENSG00000173110 /// Homo sapiens heat shock 70kDa protein 6 (HSP70B'), mRNA (cDNA clone MGC:46216 IMAGE:5723718), complete cds. /// Homo sapiens heat shock 70kDa protein 6 (HSP70B') (HSPA6), mRNA.	heat shock 70kDa protein 6 (HSP70B')	2.08988E-08	-1.740985354
STC1	Stanniocalcin-1 gene:ENSG00000159167 /// Homo sapiens stanniocalcin precursor (STC) mRNA, complete cds. /// Homo sapiens stanniocalcin 1 (STC1), mRNA.	stanniocalcin 1	0.000182276	1.474049406
HSPA4L	cDNA FLJ55529, highly similar to Heat shock 70 kDa protein 4L gene:ENSG00000164070 /// Homo sapiens heat shock protein apg-1 mRNA, complete cds. /// Homo sapiens heat shock 70kDa protein 4-like (HSPA4L), mRNA.	heat shock 70kDa protein 4-like	0.000679124	-1.242054577
**Functional Group 40**	**Transcript Description**	**Gene Title**	**Bayes.lnp**	**Fold**
SYNCRIP	Isoform 3 of Heterogeneous nuclear ribonucleoprotein Q gene:ENSG00000135316 /// Isoform 1 of Heterogeneous nuclear ribonucleoprotein Q gene:ENSG00000135316 /// Homo sapiens RRM RNA binding protein Gry-rbp (GRY-RBP) mRNA, complete cds. /// Homo sapiens synaptotagmin binding, cytoplasmic RNA interacting protein (SYNCRIP), mRNA.	synaptotagmin binding, cytoplasmic RNA interacting protein	0.00986641	1.134711189

CUGBP2	Isoform 3 of CUG-BP- and ETR-3-like factor 2 gene:ENSG00000048740 /// Isoform 2 of CUG-BP- and ETR-3-like factor 2 gene:ENSG00000048740 /// Isoform 2 of CUG-BP- and ETR-3-like factor 2 gene:ENSG00000048740 /// Isoform 1 of CUG-BP- and ETR-3-like factor 2 gene:ENSG00000048740 /// Isoform 1 of CUG-BP- and ETR-3-like factor 2 gene:ENSG00000048740 /// Isoform 2 of CUG-BP- and ETR-3-like factor 2 gene:ENSG00000048740 /// Homo sapiens apoptosis-related RNA binding protein (NAPOR-3) mRNA, complete cds. /// Homo sapiens CUG triplet repeat, RNA binding protein 2 (CUGBP2), transcript variant 1, mRNA. /// Homo sapiens CUG triplet repeat, RNA binding protein 2 (CUGBP2), transcript variant 4, mRNA. /// Homo sapiens CUG triplet repeat, RNA binding protein 2 (CUGBP2), transcript variant 3, mRNA. /// Homo sapiens CUG triplet repeat, RNA binding protein 2 (CUGBP2), transcript variant 2, mRNA.	CUG triplet repeat, RNA binding protein 2	0.001933061	-1.228518213
SNRPB	Isoform SM-B of Small nuclear ribonucleoprotein-associated proteins B and B' gene:ENSG00000125835 /// 30 kDa protein gene:ENSG00000125835 /// Isoform SM-B' of Small nuclear ribonucleoprotein-associated proteins B and B' gene:ENSG00000125835 /// Putative uncharacterized protein SNRPB gene:ENSG00000125835 /// Homo sapiens small nuclear ribonucleoprotein polypeptides B and B1, mRNA (cDNA clone MGC:99669 IMAGE:2822685), complete cds. /// Homo sapiens small nuclear ribonucleoprotein polypeptides B and B1 (SNRPB), transcript variant 2, mRNA. /// Homo sapiens small nuclear ribonucleoprotein	small nuclear ribonucleoprotein polypeptides B and B1	0.003451924	1.183670243

	polypeptides B and B1 (SNRPB), transcript variant 1, mRNA.			
LSM6	U6 snRNA-associated Sm-like protein LSm6 gene:ENSG00000164167 /// Homo sapiens, Sm protein F, clone IMAGE:4390329, mRNA. /// Homo sapiens LSM6 homolog, U6 small nuclear RNA associated (S. cerevisiae) (LSM6), mRNA.	LSM6 homolog, U6 small nuclear RNA associated (S. cerevisiae)	0.000822123	1.436021997
**Functional Group 41**	**Transcript Description**	**Gene Title**	**Bayes.lnp**	**Fold**
PLAU	urokinase plasminogen activator preproprotein gene:ENSG00000122861 /// Plasminogen activator, urokinase gene:ENSG00000122861 /// Plasminogen activator, urokinase gene:ENSG00000122861 /// Homo sapiens plasminogen activator, urokinase, mRNA (cDNA clone MGC:9223 IMAGE:3890980), complete cds. /// Homo sapiens plasminogen activator, urokinase (PLAU), mRNA.	plasminogen activator, urokinase	0.003780029	-1.172106338

TMPRSS11E	Transmembrane protease, serine 11E gene:ENSG00000087128 /// Transmembrane protease, serine 11E2 gene:ENSG00000173487 /// Homo sapiens cDNA FLJ75331 complete cds, highly similar to Homo sapiens serine protease mRNA. /// Homo sapiens clone DNA68871 serine protease (UNQ742) mRNA, complete cds. /// Homo sapiens transmembrane protease, serine 11E (TMPRSS11E), mRNA.	transmembrane protease, serine 11E2 /// transmembrane protease, serine 11E	2.20E-06	-1.554814264
PCSK1	Neuroendocrine convertase 1 gene:ENSG00000175426 /// H.sapiens PC1 (NEC1) mRNA, complete cds. /// Homo sapiens proprotein convertase subtilisin/kexin type 1 (PCSK1), mRNA.	proprotein convertase subtilisin/kexin type 1	0.001541315	-1.474321169
PLAT	Isoform 2 of Tissue-type plasminogen activator gene:ENSG00000104368 /// cDNA FLJ59358, highly similar to Tissue-type plasminogen activator gene:ENSG00000104368 /// Isoform 1 of Tissue-type plasminogen activator gene:ENSG00000104368 /// Isoform 3 of Tissue-type plasminogen activator gene:ENSG00000104368 /// Homo sapiens plasminogen activator, tissue, mRNA (cDNA clone MGC:110886 IMAGE:30528441), complete cds. /// Homo sapiens plasminogen activator, tissue (PLAT), transcript variant 3, mRNA. /// Homo sapiens plasminogen activator, tissue (PLAT), transcript variant 1, mRNA.	plasminogen activator, tissue	1.12245E-05	-1.317797286
**Functional Group 42**	**Transcript Description**	**Gene Title**	**Bayes.lnp**	**Fold**

CMAS	Isoform 1 of N-acylneuraminate cytidylyltransferase gene:ENSG00000111726 /// Homo sapiens cytidine monophosphate N-acetylneuraminic acid synthetase, mRNA (cDNA clone MGC:5424 IMAGE:3448277), complete cds. /// Homo sapiens cytidine monophosphate N-acetylneuraminic acid synthetase (CMAS), mRNA.	cytidine monophosphate N-acetylneuraminic acid synthetase	0.00042861	1.199287134
AGL	Isoform 1 of Glycogen debranching enzyme gene:ENSG00000162688 /// Putative uncharacterized protein AGL (Fragment) gene:ENSG00000162688 /// Isoform 1 of Glycogen debranching enzyme gene:ENSG00000162688 /// Isoform 1 of Glycogen debranching enzyme gene:ENSG00000162688 /// Isoform 1 of Glycogen debranching enzyme gene:ENSG00000162688 /// amylo-1, 6-glucosidase, 4-alpha-glucanotransferase isoform 3 gene:ENSG00000162688 /// amylo-1, 6-glucosidase, 4-alpha-glucanotransferase isoform 3 gene:ENSG00000162688 /// Isoform 5 of Glycogen debranching enzyme gene:ENSG00000162688 /// Human glycogen debranching enzyme isoform 4 (AGL) mRNA, alternatively spliced isoform, complete cds. /// Homo sapiens amylo-1, 6-glucosidase, 4-alpha-glucanotransferase (AGL), transcript variant 5, mRNA. /// Homo sapiens amylo-1, 6-glucosidase, 4-alpha-glucanotransferase (AGL), transcript variant 6, mRNA. /// Homo sapiens amylo-1, 6-glucosidase, 4-alpha-glucanotransferase (AGL), transcript variant 3, mRNA. /// Homo sapiens amylo-1, 6-glucosidase, 4-alpha-glucanotransferase (AGL), transcript variant 2, mRNA. /// Homo sapiens amylo-1, 6-glucosidase, 4-alpha-glucanotransferase (AGL), transcript variant 4, mRNA. /// Homo sapiens amylo-1, 6-glucosidase, 4-	amylo-1, 6-glucosidase, 4-alpha-glucanotransferase	0.007199741	1.175440036

	alpha-glucanotransferase (AGL), transcript variant 1, mRNA.			
GYG2	Isoform Beta of Glycogenin-2 gene:ENSG00000056998 /// Isoform Alpha of Glycogenin-2 gene:ENSG00000056998 /// Isoform Delta of Glycogenin-2 gene:ENSG00000056998 /// Isoform Gamma of Glycogenin-2 gene:ENSG00000056998 /// Isoform Zeta of Glycogenin-2 gene:ENSG00000056998 /// Isoform Delta of Glycogenin-2 gene:ENSG00000056998 /// Homo sapiens glycogenin-2 alpha (glycogenin-2) mRNA, complete cds. /// Homo sapiens glycogenin 2 (GYG2), transcript variant 2, mRNA.	glycogenin 2	0.008784861	1.158471866

	/// Homo sapiens glycogenin 2 (GYG2), transcript variant 1, mRNA.			
GYG1	Isoform GN-1 of Glycogenin-1 gene:ENSG00000163754 /// Isoform GN-1L of Glycogenin-1 gene:ENSG00000163754 /// Isoform GN-1S of Glycogenin-1 gene:ENSG00000163754 /// Homo sapiens glycogenin-1L mRNA, complete cds. /// Homo sapiens glycogenin 1 (GYG1), mRNA.	glycogenin 1	0.001347 231	1.198090 579
GBE1	1,4-alpha-glucan-branching enzyme gene:ENSG00000114480 /// Homo sapiens cDNA FLJ43930 fis, clone TESTI4013441, highly similar to 1,4-alpha-glucan branching enzyme (EC 2.4.1.18). /// Homo sapiens glucan (1,4-alpha-), branching enzyme 1 (GBE1), mRNA.	glucan (1,4-alpha-), branching enzyme 1	0.001161 591	- 1.181464 145
**Functional Group 43**	**Transcript Description**	**Gene Title**	**Bayes.ln p**	**Fold**

DNAJA4	DnaJ (Hsp40) homolog, subfamily A, member 4 isoform 1 gene:ENSG00000140403 /// DnaJ homolog subfamily A member 4 gene:ENSG00000140403 /// DnaJ homolog subfamily A member 4 gene:ENSG00000140403 /// Homo sapiens DnaJ (Hsp40) homolog, subfamily A, member 4, mRNA (cDNA clone MGC:33001 IMAGE:4831235), complete cds. /// Homo sapiens DnaJ (Hsp40) homolog, subfamily A, member 4 (DNAJA4), transcript variant 1, mRNA. /// Homo sapiens DnaJ (Hsp40) homolog, subfamily A, member 4 (DNAJA4), transcript variant 2, mRNA. /// Homo sapiens DnaJ (Hsp40) homolog, subfamily A, member 4 (DNAJA4), transcript variant 3, mRNA.	DnaJ (Hsp40) homolog, subfamily A, member 4	0.00145855	-1.271389224
DNAJB1	DnaJ homolog subfamily B member 1 gene:ENSG00000132002 /// Homo sapiens hsp40 mRNA for heat-shock protein 40, complete cds. /// Homo sapiens DnaJ (Hsp40) homolog, subfamily B, member 1 (DNAJB1), mRNA.	DnaJ (Hsp40) homolog, subfamily B, member 1	1.03457E-07	-1.545182101
DNAJC15	DnaJ homolog subfamily C member 15 gene:ENSG00000120675 /// Homo sapiens DnaJ (Hsp40) homolog, subfamily C, member 15, mRNA (cDNA clone MGC:110875 IMAGE:30530999), complete cds. /// Homo sapiens DnaJ (Hsp40) homolog, subfamily C, member 15 (DNAJC15), mRNA.	DnaJ (Hsp40) homolog, subfamily C, member 15	0.002111086	1.248706673
DNAJB4	DnaJ homolog subfamily B member 4 gene:ENSG00000162616 /// Homo sapiens DnaJ (Hsp40) homolog, subfamily B, member 4, mRNA (cDNA clone MGC:17027 IMAGE:4340658), complete cds. /// Homo sapiens DnaJ (Hsp40) homolog, subfamily B, member 4 (DNAJB4), mRNA.	DnaJ (Hsp40) homolog, subfamily B, member 4	0.008889347	-1.17899242

**Functional Group 44**	**Transcript Description**	**Gene Title**	**Bayes.lnp**	**Fold**
MYO1D	Isoform 1 of Myosin-Id gene:ENSG00000176658 /// Isoform 2 of Myosin-Id gene:ENSG00000176658 /// Homo sapiens myosin ID, mRNA (cDNA clone MGC:166833 IMAGE:9007203), complete cds. /// Homo sapiens myosin ID (MYO1D), mRNA.	myosin ID	0.004287663	-1.170542076
KIF20A	Kinesin-like protein KIF20A gene:ENSG00000112984 /// Kinesin-like protein KIF20A gene:ENSG00000112984 /// Homo sapiens kinesin family member 20A, mRNA (cDNA clone MGC:3571 IMAGE:3604291), complete cds. /// Homo sapiens kinesin family member 20A (KIF20A), mRNA.	kinesin family member 20A	0.000390543	1.232560547
DNAL4	Dynein light chain 4, axonemal gene:ENSG00000100246 /// Dynein, axonemal, light chain 4 gene:ENSG00000100246 /// Homo sapiens dynein, axonemal, light chain 4, mRNA (cDNA clone MGC:1838 IMAGE:3543962), complete cds. /// Homo sapiens dynein, axonemal, light chain 4 (DNAL4), mRNA.	dynein, axonemal, light chain 4	0.006784268	1.230144814
MAP7	Isoform 1 of Ensconsin gene:ENSG00000135525 /// Putative uncharacterized protein MAP7 gene:ENSG00000135525 /// Homo sapiens microtubule-associated protein 7, mRNA (cDNA clone MGC:34092 IMAGE:5222987), complete cds. /// Homo sapiens microtubule-associated protein 7 (MAP7), mRNA.	microtubule-associated protein 7	0.000184608	1.255111882
KIF3C	Kinesin-like protein KIF3C gene:ENSG00000084731 /// Kinesin-like protein KIF3C gene:ENSG00000084731 /// Homo sapiens kinesin-related protein (KIF3C) mRNA, complete cds. ///	kinesin family member 3C	0.005222742	1.217588424

	Homo sapiens kinesin family member 3C (KIF3C), mRNA.			
KIF21A	Isoform 1 of Kinesin-like protein KIF21A gene:ENSG00000139116 /// Isoform 4 of Kinesin-like protein KIF21A gene:ENSG00000139116 /// Isoform 3 of Kinesin-like protein KIF21A gene:ENSG00000139116 /// Isoform 2 of Kinesin-like protein KIF21A gene:ENSG00000139116 /// Homo sapiens kinesin family member 21A (KIF21A) mRNA, complete cds, alternatively spliced. /// Homo sapiens kinesin family member 21A (KIF21A), mRNA.	kinesin family member 21A	0.008568736	1.197613115
KIF4A	Isoform 2 of Chromosome-associated kinesin KIF4A gene:ENSG00000090889 /// Isoform 1 of Chromosome-associated kinesin KIF4A gene:ENSG00000090889 /// Homo sapiens kinesin superfamily motor KIF4 mRNA, complete cds. /// Homo sapiens kinesin family member 4A (KIF4A), mRNA.	kinesin family member 4A	0.00023618	1.303272785
DYNC1LI1	Cytoplasmic dynein 1 light intermediate chain 1 gene:ENSG00000144635 /// Homo sapiens zinc finger, RAN-binding domain containing 3, mRNA (cDNA clone IMAGE:5575956), complete cds. /// Homo sapiens dynein, cytoplasmic 1, light intermediate chain 1, mRNA (cDNA clone MGC:149683 IMAGE:40117391), complete cds. /// Homo sapiens dynein, cytoplasmic 1, light intermediate chain 1 (DYNC1LI1), mRNA.	zinc finger, RAN-binding domain containing 3 /// dynein, cytoplasmic 1, light intermediate chain 1	0.008493891	1.164885701

KIF22	cdna:known supercontig::NT_113929:89369:97443:1 gene:ENSG00000215670 /// OriP binding protein like gene product gene:ENSG00000215669 /// Kinesin-like protein KIF22 gene:ENSG00000079616 /// Homo sapiens mRNA for kinesin-like DNA binding protein, complete cds. /// Homo sapiens kinesin family member 22 (KIF22), mRNA.	kinesin family member 22	0.003496917	1.17255889
**Functional Group 45**	**Transcript Description**	**Gene Title**	**Bayes.lnp**	**Fold**
UNC5B	Isoform 1 of Netrin receptor UNC5B gene:ENSG00000107731 /// Isoform 2 of Netrin receptor UNC5B gene:ENSG00000107731 /// Homo sapiens p53RDL1 mRNA for p53-regulated receptor for death and life, complete cds. /// Homo sapiens unc-5 homolog B (C. elegans) (UNC5B), mRNA.	unc-5 homolog B (C. elegans)	0.001325655	-1.218302468
IL7R	Isoform H20 of Interleukin-7 receptor subunit alpha gene:ENSG00000168685 /// Isoform H6 of Interleukin-7 receptor subunit alpha gene:ENSG00000168685 /// Homo sapiens interleukin 7 receptor, mRNA (cDNA clone MGC:79450 IMAGE:6971846), complete cds. /// Homo sapiens interleukin 7 receptor (IL7R), mRNA.	interleukin 7 receptor	3.25743E-05	-1.560892995

IL6ST	Isoform 1 of Interleukin-6 receptor subunit beta gene:ENSG00000134352 /// Isoform 1 of Interleukin-6 receptor subunit beta gene:ENSG00000134352 /// IL6ST nirs variant 4 gene:ENSG00000134352 /// IL6ST nirs variant 3 gene:ENSG00000134352 /// Isoform 1 of Interleukin-6 receptor subunit beta gene:ENSG00000134352 /// IL6ST nirs variant 6 gene:ENSG00000134352 /// Isoform 2 of Interleukin-6 receptor subunit beta gene:ENSG00000134352 /// IL6ST nirs variant 5 gene:ENSG00000134352 /// IL6ST nirs variant 1 gene:ENSG00000134352 /// IL6ST nirs variant 2 gene:ENSG00000134352 /// Human membrane glycoprotein gp130 mRNA, complete cds. /// Homo sapiens interleukin 6 signal transducer (gp130, oncostatin M receptor) (IL6ST), transcript variant 2, mRNA. /// Homo sapiens interleukin 6 signal transducer (gp130, oncostatin M receptor) (IL6ST), transcript variant 1, mRNA.	interleukin 6 signal transducer (gp130, oncostatin M receptor)	0.004311509	-1.202321214
SLC11A2	Isoform 2 of Natural resistance-associated macrophage protein 2 gene:ENSG00000110911 /// Isoform 1 of Natural resistance-associated macrophage protein 2 gene:ENSG00000110911 /// Divalent metal transporter gene:ENSG00000110911 /// Homo sapiens cDNA FLJ46799 fis, clone TRACH3031678, highly similar to Natural resistance-associated macrophage protein 2. /// Homo sapiens solute carrier family 11 (proton-coupled divalent metal ion transporters), member 2 (SLC11A2), mRNA.	solute carrier family 11 (proton-coupled divalent metal ion transporters), member 2	0.001989436	1.200926252

GABRE	Gamma-aminobutyric acid receptor subunit epsilon gene:ENSG00000102287 /// Putative uncharacterized protein GABRE gene:ENSG00000102287 /// Putative uncharacterized protein GABRE gene:ENSG00000102287 /// 42 kDa protein gene:ENSG00000102287 /// Homo sapiens gamma-aminobutyric acid (GABA) A receptor, epsilon, mRNA (cDNA clone MGC:26504 IMAGE:4798754), complete cds. /// Homo sapiens gamma-aminobutyric acid (GABA) A receptor, epsilon (GABRE), mRNA.	gamma-aminobutyric acid (GABA) A receptor, epsilon	4.29924E-10	-2.02462221
CHRNA9	Neuronal acetylcholine receptor subunit alpha-9 gene:ENSG00000174343 /// Homo sapiens cholinergic receptor, nicotinic, alpha 9, mRNA (cDNA clone MGC:142109 IMAGE:8322601), complete cds. /// Homo sapiens cholinergic receptor, nicotinic, alpha 9 (CHRNA9), mRNA.	cholinergic receptor, nicotinic, alpha 9	0.008365153	-1.272045213
ITGA2	Integrin alpha-2 gene:ENSG00000164171 /// Homo sapiens integrin, alpha 2 (CD49B, alpha 2 subunit of VLA-2 receptor) (ITGA2), mRNA.	integrin, alpha 2 (CD49B, alpha 2 subunit of VLA-2 receptor)	1.87799E-05	-1.323995521

HLA-DQB2	Major histocompatibility complex, class II, DQ beta 2 gene:ENSG00000215008 /// Major histocompatibility complex, class II, DQ beta 2 gene:ENSG00000215008 /// Major histocompatibility complex, class II, DQ beta 2 gene:ENSG00000215008 /// Major histocompatibility complex, class II, DQ beta 2 gene:ENSG00000215008 /// Major histocompatibility complex, class II, DQ beta 2 gene:ENSG00000196610 /// Major histocompatibility complex, class II, DQ beta 2 gene:ENSG00000196610 /// Major histocompatibility complex, class II, DQ beta 2 gene:ENSG00000196610 /// cdna:known chromosome:NCBI36:c6_QBL:32795451:32802909:-1 gene:ENSG00000196610 /// Major histocompatibility complex, class II, DQ beta 2 gene:ENSG00000204275 /// Major histocompatibility complex, class II, DQ beta 2 gene:ENSG00000204275 /// Major histocompatibility complex, class II, DQ beta 2 gene:ENSG00000204275 /// Major histocompatibility complex, class II, DQ beta 2 gene:ENSG00000204275 /// Homo sapiens cDNA FLJ40688 fis, clone THYMU2024185, highly similar to HLA class II histocompatibility antigen, DX beta chain precursor. /// Homo sapiens major histocompatibility complex, class II, DQ beta 2 (HLA-DQB2), non-coding RNA.	major histocompatibility complex, class II, DQ beta 2	0.003586271	-1.177848967

MICA	MHC class I polypeptide-related sequence A gene:ENSG00000184444 /// Putative uncharacterized protein gene:ENSG00000184444 /// MHC class I polypeptide-related sequence A gene:ENSG00000184444 /// cdna:known chromosome:NCBI36:c6_COX:31509120:31520840:1 gene:ENSG00000184444 /// MHC class I polypeptide-related sequence A gene:ENSG00000183214 /// MHC class I polypeptide-related sequence A gene:ENSG00000183214 /// MHC class I polypeptide-related sequence A gene:ENSG00000183214 /// MHC class I polypeptide-related sequence A gene:ENSG00000204520 /// Putative uncharacterized protein gene:ENSG00000204520 /// MHC class I polypeptide-related sequence A gene:ENSG00000204520 /// cdna:known chromosome:NCBI36:6:31479389:31490895:1 gene:ENSG00000204520 /// cdna:known chromosome:NCBI36:6:31484427:31491015:1 gene:ENSG00000204520 /// Homo sapiens cDNA FLJ60820 complete cds, highly similar to Homo sapiens MHC class I polypeptide-related sequence A (MICA), mRNA. /// Homo sapiens MHC class I polypeptide-related sequence A (MICA), mRNA.	MHC class I polypeptide-related sequence A	0.405683878	1.047499821

HLA-A	Major histocompatibility complex, class I, C gene:ENSG00000206435 /// Major histocompatibility complex, class I, C gene:ENSG00000206435 /// Major histocompatibility complex, class I, C gene:ENSG00000206435 /// Major histocompatibility complex, class I, C gene:ENSG00000206435 /// cdna:known chromosome:NCBI36:c6_COX:31374864:31378212:-1 gene:ENSG00000206435 /// HLA class I histocompatibility antigen, B-8 alpha chain gene:ENSG00000206341 /// Major histocompatibility complex class I C gene:ENSG00000206452 /// MHC class I antigen gene:ENSG00000206452 /// Major histocompatibility complex class I C gene:ENSG00000206452 /// Major histocompatibility complex class I C gene:ENSG00000206452 /// MHC class I antigen gene:ENSG00000206452 /// MHC class I antigen gene:ENSG00000206452 /// HLA-Cw12 gene:ENSG00000206452 /// HLA class I histocompatibility antigen, B-18 alpha chain gene:ENSG00000206450 /// cdna:known chromosome:NCBI36:c6_QBL:31456916:31457994:-1 gene:ENSG00000206450 /// Major histocompatibility complex, class I, C gene:ENSG00000204525 /// Major histocompatibility complex, class I, C gene:ENSG00000204525 /// HLA class I histocompatibility antigen, Cw-7 alpha chain gene:ENSG00000204525 /// Major histocompatibility complex, class I, C gene:ENSG00000204525 /// HLA class I histocompatibility antigen, B-7 alpha chain gene:ENSG00000204525 /// Homo sapiens major histocompatibility complex, class I, C, mRNA (cDNA clone MGC:2285 IMAGE:3345005),	major histocompatibility complex, class I, C /// major histocompatibility complex, class I, B /// MHC class I polypeptide-related sequence A /// major histocompatibility complex, class I, A	0.006852189	-1.144039305

	complete cds. /// Homo sapiens major histocompatibility complex, class I, B, mRNA (cDNA clone MGC:17189 IMAGE:4344323), complete cds. /// Homo sapiens MHC class I polypeptide-related sequence A, mRNA (cDNA clone MGC:111087 IMAGE:30386550), complete cds. /// Homo sapiens HLA-A24AK mRNA, complete cds. /// Homo sapiens major histocompatibility complex, class I, C (HLA-C), mRNA. /// Homo sapiens major histocompatibility complex, class I, B (HLA-B), mRNA.			

HLA-C	Major histocompatibility complex, class I, C gene:ENSG00000206435 /// Major histocompatibility complex, class I, C gene:ENSG00000206435 /// Major histocompatibility complex, class I, C gene:ENSG00000206435 /// Major histocompatibility complex, class I, C gene:ENSG00000206435 /// cdna:known chromosome:NCBI36:c6_COX:31374864:31378212:-1 gene:ENSG00000206435 /// cdna:known chromosome:NCBI36:c6_COX:31374864:31378212:-1 gene:ENSG00000206435 /// HLA class I histocompatibility antigen, B-8 alpha chain gene:ENSG00000206341 /// Major histocompatibility complex class I C gene:ENSG00000206452 /// MHC class I antigen gene:ENSG00000206452 /// Major histocompatibility complex class I C gene:ENSG00000206452 /// Major histocompatibility complex class I C gene:ENSG00000206452 /// MHC class I antigen gene:ENSG00000206452 /// MHC class I antigen gene:ENSG00000206452 /// HLA-Cw12 gene:ENSG00000206452 /// Major histocompatibility complex, class I, C gene:ENSG00000204525 /// Major histocompatibility complex, class I, C gene:ENSG00000204525 /// HLA class I histocompatibility antigen, Cw-7 alpha chain gene:ENSG00000204525 /// Major histocompatibility complex, class I, C gene:ENSG00000204525 /// Homo sapiens major histocompatibility complex, class I, B, mRNA (cDNA clone MGC:17189 IMAGE:4344323), complete cds. /// Homo sapiens major histocompatibility complex, class I, C, mRNA (cDNA clone MGC:24960 IMAGE:4780246), complete cds. /// Homo sapiens MHC	major histocompatibility complex, class I, B /// major histocompatibility complex, class I, C /// MHC class I polypeptide-related sequence A	0.05510185	-1.114334938

	class I polypeptide-related sequence A, mRNA (cDNA clone MGC:111087 IMAGE:30386550), complete cds. /// Homo sapiens major histocompatibility complex, class I, C (HLA-C), mRNA.			
DCBLD2	Isoform 1 of Discoidin, CUB and LCCL domain-containing protein 2 gene:ENSG00000057019 /// Isoform 2 of Discoidin, CUB and LCCL domain-containing protein 2 gene:ENSG00000057019 /// 42 kDa protein gene:ENSG00000057019 /// Homo sapiens mRNA for CLCP1, complete cds. /// Homo sapiens discoidin, CUB and LCCL domain containing 2 (DCBLD2), mRNA.	discoidin, CUB and LCCL domain containing 2	0.004394744	-1.154840632

COL17A1	Isoform 2 of Collagen alpha-1(XVII) chain gene:ENSG00000065618 /// Isoform 1 of Collagen alpha-1(XVII) chain gene:ENSG00000065618 /// Collagen type XVII alpha 1 gene:ENSG00000065618 /// Homo sapiens collagen, type XVII, alpha 1 (COL17A1), mRNA.	collagen, type XVII, alpha 1	2.82636E-11	-1.968830888
SLC19A2	Solute carrier family 19 (Thiamine transporter), member 2 gene:ENSG00000117479 /// Thiamine transporter 1 gene:ENSG00000117479 /// Solute carrier family 19 (Thiamine transporter), member 2, isoform CRA_c gene:ENSG00000117479 /// Homo sapiens high affinity thiamine transporter mRNA, complete cds. /// Homo sapiens solute carrier family 19 (thiamine transporter), member 2 (SLC19A2), mRNA.	solute carrier family 19 (thiamine transporter), member 2	0.009821935	1.173602075
PTAFR	Platelet-activating factor receptor gene:ENSG00000169403 /// Platelet-activating factor receptor gene:ENSG00000169403 /// cdna:known chromosome:NCBI36:1:28392629:28393024:-1 gene:ENSG00000215903 /// platelet-activating factor receptor [human, heart ventricle, mRNA Partial, 1541 nt]. /// Homo sapiens platelet-activating factor receptor (PTAFR), mRNA.	platelet-activating factor receptor	0.007269296	-1.226927777
PCDHB5	Protocadherin beta-5 gene:ENSG00000113209 /// Homo sapiens protocadherin beta 5, mRNA (cDNA clone MGC:2649 IMAGE:3528826), complete cds. /// Homo sapiens protocadherin beta 5 (PCDHB5), mRNA.	protocadherin beta 5	0.004059543	1.170733987
CCKBR	Isoform 1 of Gastrin/cholecystokinin type B receptor gene:ENSG00000110148 /// Homo sapiens CCK-B/gastrin receptor mRNA, complete cds; alternatively spliced. /// Homo sapiens cholecystokinin B receptor (CCKBR), mRNA.	cholecystokinin B receptor	0.007359642	1.256475897

MCHR1	G-protein coupled receptor 24 isoform 1 gene:ENSG00000128285 /// Melanin-concentrating hormone receptor 1 gene:ENSG00000128285 /// Homo sapiens G protein-coupled receptor 24 (GPR24) mRNA, complete cds. /// Homo sapiens melanin-concentrating hormone receptor 1 (MCHR1), mRNA.	melanin-concentrating hormone receptor 1	0.004686217	-1.230678471
ICAM1	Intercellular adhesion molecule 1 gene:ENSG00000090339 /// Human major group rhinovirus receptor (HRV) mRNA, complete cds. /// Homo sapiens intercellular adhesion molecule 1 (ICAM1), mRNA.	intercellular adhesion molecule 1	7.61225E-05	-1.515303835
PODXL	Podocalyxin-like protein 1 gene:ENSG00000128567 /// podocalyxin-like isoform 1 precursor gene:ENSG00000128567 /// Homo sapiens podocalyxin-like protein mRNA, complete cds. /// Homo sapiens podocalyxin-like (PODXL), transcript variant 1, mRNA. /// Homo sapiens podocalyxin-like (PODXL), transcript variant 2, mRNA.	podocalyxin-like	0.001142461	1.219198904
LGI2	Leucine-rich repeat LGI family member 2 gene:ENSG00000153012 /// 22 kDa protein gene:ENSG00000153012 /// Homo sapiens leucine-rich repeat LGI family, member 2, mRNA (cDNA clone MGC:126808 IMAGE:8069265), complete cds. /// Homo sapiens leucine-rich repeat LGI family, member 2 (LGI2), mRNA.	leucine-rich repeat LGI family, member 2	0.005632471	1.219235768
TNFSF9	Tumor necrosis factor ligand superfamily member 9 gene:ENSG00000125657 /// Human receptor 4-1BB ligand mRNA, complete cds. /// Homo sapiens tumor necrosis factor (ligand) superfamily, member 9 (TNFSF9), mRNA.	tumor necrosis factor (ligand) superfamily, member 9	6.73812E-05	-1.368642193

GPC1	Glypican-1 gene:ENSG00000063660 /// Human mRNA for heparan sulfate proteaglycan (glypican). /// Homo sapiens glypican 1 (GPC1), mRNA.	glypican 1	0.000536077	-1.254215314
EPHB1	Isoform 1 of Ephrin type-B receptor 1 gene:ENSG00000154928 /// Isoform 4 of Ephrin type-B receptor 1 gene:ENSG00000154928 /// Homo sapiens (clone FBK III 16) protein tyrosine kinase (NET PTK) mRNA, complete cds. /// Homo sapiens EPH receptor B1 (EPHB1), mRNA.	EPH receptor B1	0.000880803	-1.270478492
CDH11	Isoform 2 of Cadherin-11 gene:ENSG00000140937 /// Isoform 1 of Cadherin-11 gene:ENSG00000140937 /// Isoform 2 of Cadherin-11 gene:ENSG00000140937 /// Homo sapiens mRNA for OB-cadherin-2, complete cds. /// Homo sapiens cadherin 11, type 2, OB-cadherin (osteoblast) (CDH11), mRNA.	cadherin 11, type 2, OB-cadherin (osteoblast)	0.000459146	-1.2560768
SELPLG	P-selectin glycoprotein ligand 1 gene:ENSG00000110876 /// selectin P ligand gene:ENSG00000110876 /// Homo sapiens selectin P ligand, mRNA (cDNA clone MGC:34401 IMAGE:5180824), complete cds. /// Homo sapiens selectin P ligand (SELPLG), mRNA.	selectin P ligand	1.53006E-05	-1.54513417
CDH10	Cadherin-10 gene:ENSG00000040731 /// Homo sapiens CDH10 mRNA for cadherin-10, complete cds. /// Homo sapiens cadherin 10, type 2 (T2-cadherin) (CDH10), mRNA.	cadherin 10, type 2 (T2-cadherin)	0.000193699	-1.496845555
GPRC5A	Retinoic acid-induced protein 3 gene:ENSG00000013588 /// Homo sapiens cDNA FLJ16117 fis, clone ASTRO2003632, highly similar to Retinoic acid-induced protein 3 (G-proteincoupled receptor family C group 5 member A). /// Homo sapiens G protein-coupled receptor, family C, group 5, member A (GPRC5A), mRNA.	G protein-coupled receptor, family C, group 5, member A	4.48256E-06	-1.572536281

NPY1R	Neuropeptide Y receptor type 1 gene:ENSG00000164128 /// Human neuropeptide Y receptor Y1 (NPYY1) mRNA, exon 2-3 and complete cds. /// Homo sapiens neuropeptide Y receptor Y1 (NPY1R), mRNA.	neuropeptide Y receptor Y1	0.001569123	-1.244647364
OR52N4	Olfactory receptor 52N4 gene:ENSG00000181074 /// Homo sapiens olfactory receptor, family 52, subfamily N, member 4, mRNA (cDNA clone MGC:168654 IMAGE:9021031), complete cds. /// Homo sapiens olfactory receptor, family 52, subfamily N, member 4 (OR52N4), mRNA.	olfactory receptor, family 52, subfamily N, member 4	0.000471799	-1.48967733
AREG	Amphiregulin gene:ENSG00000109321 /// Amphiregulin gene:ENSG00000109321 /// Amphiregulin gene:ENSG00000205595 /// Homo sapiens amphiregulin, mRNA (cDNA clone MGC:13647 IMAGE:4277616), complete cds. /// Homo sapiens amphiregulin (AREG), mRNA.	amphiregulin	7.1454E-13	-2.274668861
GPR39	Probable G-protein coupled receptor 39 gene:ENSG00000183840 /// Homo sapiens G protein-coupled receptor 39 (GPR39) mRNA, complete cds. /// Homo sapiens G protein-coupled receptor 39 (GPR39), mRNA.	G protein-coupled receptor 39	1.90394E-05	1.580011466

CD44	CD44 antigen isoform 5 precursor gene:ENSG00000026508 /// Isoform CD44 of CD44 antigen gene:ENSG00000026508 /// Isoform 4 of CD44 antigen gene:ENSG00000026508 /// Isoform 10 of CD44 antigen gene:ENSG00000026508 /// Isoform 12 of CD44 antigen gene:ENSG00000026508 /// Putative uncharacterized protein CD44 gene:ENSG00000026508 /// Homo sapiens mRNA; cDNA DKFZp451K1918 (from clone DKFZp451K1918); complete cds. /// Homo sapiens CD44 molecule (Indian blood group) (CD44), transcript variant 1, mRNA. /// Homo sapiens CD44 molecule (Indian blood group) (CD44), transcript variant 2, mRNA. /// Homo sapiens CD44 molecule (Indian blood group) (CD44), transcript variant 3, mRNA. /// Homo sapiens CD44 molecule (Indian blood group) (CD44), transcript variant 4, mRNA. /// Homo sapiens CD44 molecule (Indian blood group) (CD44), transcript variant 5, mRNA.	CD44 molecule (Indian blood group)	0.000512 308	- 1.224125 799
GPC4	Glypican-4 gene:ENSG00000076716 /// Homo sapiens glypican-4 (GPC4) mRNA, complete cds. /// Homo sapiens glypican 4 (GPC4), mRNA.	glypican 4	0.000462 664	1.248198 109
HTR1D	5-hydroxytryptamine receptor 1D gene:ENSG00000179546 /// 5-hydroxytryptamine receptor 1D gene:ENSG00000179546 /// Homo sapiens 5-hydroxytryptamine (serotonin) receptor 1D, mRNA (cDNA clone MGC:12645 IMAGE:4299633), complete cds. /// Homo sapiens 5-hydroxytryptamine (serotonin) receptor 1D (HTR1D), mRNA.	5-hydroxytryptamine (serotonin) receptor 1D	0.002215 237	1.333056 646

BTN3A2	Butyrophilin subfamily 3 member A2 gene:ENSG00000186470 /// Butyrophilin subfamily 3 member A2 gene:ENSG00000186470 /// Butyrophilin subfamily 3 member A2 gene:ENSG00000186470 /// Homo sapiens butyrophilin, subfamily 3, member A2, mRNA (cDNA clone MGC:31880 IMAGE:4870774), complete cds. /// Homo sapiens butyrophilin, subfamily 3, member A2 (BTN3A2), mRNA.	butyrophilin, subfamily 3, member A2	0.003331506	1.332230205
EDNRA	21 kDa protein gene:ENSG00000151617 /// Isoform 2 of Endothelin-1 receptor gene:ENSG00000151617 /// Isoform 1 of Endothelin-1 receptor gene:ENSG00000151617 /// Isoform 4 of Endothelin-1 receptor gene:ENSG00000151617 /// Homo sapiens mRNA for endothelin-1 receptor, complete cds. /// Homo sapiens endothelin receptor type A (EDNRA), mRNA.	endothelin receptor type A	0.002523627	1.370171
SEZ6L	Seizure related 6 homolog (Mouse)-like gene:ENSG00000100095 /// Isoform 4 of Seizure 6-like protein gene:ENSG00000100095 /// Isoform 3 of Seizure 6-like protein gene:ENSG00000100095 /// Seizure related 6 homolog (Mouse)-like gene:ENSG00000100095 /// Isoform 5 of Seizure 6-like protein gene:ENSG00000100095 /// Seizure related 6 homolog (Mouse)-like gene:ENSG00000100095 /// Seizure related 6 homolog (Mouse)-like gene:ENSG00000100095 /// Homo sapiens clone DNA96995 SEZ6L (UNQ2542) mRNA, complete cds. /// Homo sapiens seizure related 6 homolog (mouse)-like (SEZ6L), mRNA.	seizure related 6 homolog (mouse)-like	0.0062394	1.333996711

SLC1A1	Excitatory amino acid transporter 3 gene:ENSG00000106688 /// Solute carrier family 1 (Neuronal/epithelial high affinity glutamate transporter, system Xag), member 1 gene:ENSG00000106688 /// Homo sapiens solute carrier family 1 (neuronal/epithelial high affinity glutamate transporter, system Xag), member 1, mRNA (cDNA clone MGC:33786 IMAGE:5261168), complete cds. /// Homo sapiens solute carrier family 1 (neuronal/epithelial high affinity glutamate transporter, system Xag), member 1 (SLC1A1), mRNA.	solute carrier family 1 (neuronal/epithelial high affinity glutamate transporter, system Xag), member 1	0.000392958	1.343599293
NGFR	Tumor necrosis factor receptor superfamily member 16 gene:ENSG00000064300 /// Homo sapiens nerve growth factor receptor (TNFR superfamily, member 16), mRNA (cDNA clone MGC:48326 IMAGE:5263715), complete cds. /// Homo sapiens nerve growth factor receptor (TNFR superfamily, member 16) (NGFR), mRNA.	nerve growth factor receptor (TNFR superfamily, member 16)	4.39467E-06	-1.39902999
GPR87	Probable G-protein coupled receptor 87 gene:ENSG00000138271 /// Homo sapiens KPG_002 mRNA, complete cds. /// Homo sapiens G protein-coupled receptor 87 (GPR87), mRNA.	G protein-coupled receptor 87	9.09167E-05	-1.493376242

PTPRU	Receptor-type tyrosine-protein phosphatase U gene:ENSG00000060656 /// protein tyrosine phosphatase, receptor type, U isoform 2 precursor gene:ENSG00000060656 /// protein tyrosine phosphatase, receptor type, U isoform 3 precursor gene:ENSG00000060656 /// cDNA FLJ37530 fis, clone BRCAN2012713, highly similar to Receptor-type tyrosine-protein phosphatase U gene:ENSG00000060656 /// FMI protein gene:ENSG00000060656 /// Homo sapiens protein tyrosine phosphatase, receptor type, U, mRNA (cDNA clone MGC:164848 IMAGE:40147934), complete cds. /// Homo sapiens protein tyrosine phosphatase, receptor type, U (PTPRU), transcript variant 3, mRNA. /// Homo sapiens protein tyrosine phosphatase, receptor type, U (PTPRU), transcript variant 2, mRNA. /// Homo sapiens protein tyrosine phosphatase, receptor type, U (PTPRU), transcript variant 1, mRNA.	protein tyrosine phosphatase, receptor type, U	0.004666787	-1.191944005
TNFRSF9	Tumor necrosis factor receptor superfamily member 9 gene:ENSG00000049249 /// Human activation dependent T cell mRNA, complete cds. /// Homo sapiens tumor necrosis factor receptor superfamily, member 9 (TNFRSF9), mRNA.	tumor necrosis factor receptor superfamily, member 9	3.28208E-06	-1.98832707
FZD7	Frizzled-7 gene:ENSG00000155760 /// Homo sapiens mRNA for frizzled-7, complete cds. /// Homo sapiens frizzled homolog 7 (Drosophila) (FZD7), mRNA.	frizzled homolog 7 (Drosophila)	0.000402709	1.210947967

EDNRB	Isoform A of Endothelin B receptor gene:ENSG00000136160 /// Isoform C of Endothelin B receptor gene:ENSG00000136160 /// Homo sapiens endothelin ET-B receptor mRNA, complete cds. /// Homo sapiens endothelin receptor type B (EDNRB), transcript variant 2, mRNA. /// Homo sapiens endothelin receptor type B (EDNRB), transcript variant 1, mRNA. /// Homo sapiens endothelin receptor type B (EDNRB), transcript variant 3, mRNA.	endothelin receptor type B	0.007709 296	1.213146 514
TNFRSF10D	Tumor necrosis factor receptor superfamily member 10D gene:ENSG00000173530 /// Homo sapiens clone DNA35663 DcR2-TNFR (UNQ251) mRNA, complete cds. /// Homo sapiens tumor necrosis factor receptor superfamily, member 10d, decoy with truncated death domain (TNFRSF10D), mRNA.	tumor necrosis factor receptor superfamily, member 10d, decoy with truncated death domain	9.2738E-06	-1.372480 179
CD36	Platelet glycoprotein 4 gene:ENSG00000135218 /// Platelet glycoprotein 4 gene:ENSG00000135218 /// Platelet glycoprotein 4 gene:ENSG00000135218 /// Human antigen CD36 (clone 13) mRNA, complete cds. /// Homo sapiens CD36 molecule (thrombospondin receptor) (CD36), transcript variant 1, mRNA. /// Homo sapiens CD36 molecule (thrombospondin receptor) (CD36), transcript variant 2, mRNA. /// Homo sapiens CD36 molecule (thrombospondin receptor) (CD36), transcript variant 3, mRNA. /// Homo sapiens CD36 molecule (thrombospondin receptor) (CD36), transcript variant 4, mRNA. /// Homo sapiens CD36 molecule (thrombospondin receptor) (CD36), transcript variant 5, mRNA.	CD36 molecule (thrombospondin receptor)	0.002137 002	-1.364933 341

ANTXR2	Isoform 4 of Anthrax toxin receptor 2 gene:ENSG00000163297 /// ANTXR2 protein gene:ENSG00000163297 /// Isoform 4 of Anthrax toxin receptor 2 gene:ENSG00000163297 /// Isoform 1 of Anthrax toxin receptor 2 gene:ENSG00000163297 /// Isoform 2 of Anthrax toxin receptor 2 gene:ENSG00000163297 /// Isoform 3 of Anthrax toxin receptor 2 gene:ENSG00000163297 /// Homo sapiens cDNA FLJ31074 fis, clone HSYRA2001476. /// Homo sapiens anthrax toxin receptor 2 (ANTXR2), mRNA.	anthrax toxin receptor 2	0.000582301	-1.248326997
OR10P1	Seven transmembrane helix receptor gene:ENSG00000175398 /// Homo sapiens olfactory receptor, family 10, subfamily P, member 1 (OR10P1), mRNA.	olfactory receptor, family 10, subfamily P, member 1	0.000510633	1.428520049
SLC2A9	Isoform 1 of Solute carrier family 2, facilitated glucose transporter member 9 gene:ENSG00000109667 /// Isoform 2 of Solute carrier family 2, facilitated glucose transporter member 9 gene:ENSG00000109667 /// Homo sapiens facilitative glucose transporter family member GLUT9 (SLC2A9) mRNA, complete cds. /// Homo sapiens solute carrier family 2 (facilitated glucose transporter), member 9 (SLC2A9), transcript variant 1, mRNA. /// Homo sapiens solute carrier family 2 (facilitated glucose transporter), member 9 (SLC2A9), transcript variant 2, mRNA.	solute carrier family 2 (facilitated glucose transporter), member 9	0.004482523	-1.385852195
IL8RB	High affinity interleukin-8 receptor B gene:ENSG00000180871 /// Homo sapiens interleukin 8 receptor beta (IL8RB) mRNA, complete cds. /// Homo sapiens interleukin 8 receptor, beta (IL8RB), mRNA.	interleukin 8 receptor, beta	0.001773824	-1.457311024

HTR2C	5-hydroxytryptamine receptor 2C gene:ENSG00000147246 /// 5-hydroxytryptamine receptor 2C gene:ENSG00000147246 /// 5-hydroxytryptamine (Serotonin) receptor 2C gene:ENSG00000147246 /// Human serotonin 5-HT1C receptor mRNA, complete cds. /// Homo sapiens 5-hydroxytryptamine (serotonin) receptor 2C (HTR2C), mRNA.	5-hydroxytryptamine (serotonin) receptor 2C	7.31336E-06	-1.562541147
PLSCR3	cdna:known chromosome:NCBI36:17:7233779:7238567:-1 gene:ENSG00000187838 /// cdna:known chromosome:NCBI36:17:7233779:7238572:-1 gene:ENSG00000187838 /// cdna:known chromosome:NCBI36:17:7234428:7238153:-1 gene:ENSG00000187838 /// Homo sapiens phospholipid scramblase 3, mRNA (cDNA clone MGC:110921 IMAGE:30341717), complete cds. /// Homo sapiens phospholipid scramblase 3 (PLSCR3), mRNA.	phospholipid scramblase 3	0.2369122	1.083089441
ERVWE1	HERV-W_7q21.2 provirus ancestral Env polyprotein gene:ENSG00000197604 /// HERV-W_7q21.2 provirus ancestral Env polyprotein gene:ENSG00000197604 /// Homo sapiens syncytin precursor, mRNA, complete cds. /// Homo sapiens endogenous retroviral family W, env(C7), member 1 (ERVWE1), transcript variant 2, mRNA. /// Homo sapiens endogenous retroviral family W, env(C7), member 1 (ERVWE1), transcript variant 1, mRNA.	endogenous retroviral family W, env(C7), member 1	4.70755E-05	-1.633324962
SLITRK1	SLIT and NTRK-like protein 1 gene:ENSG00000178235 /// Homo sapiens SLIT and NTRK-like family, member 1, mRNA (cDNA clone MGC:51091 IMAGE:4816570), complete cds. /// Homo sapiens SLIT and NTRK-like family, member 1 (SLITRK1), mRNA.	SLIT and NTRK-like family, member 1	0.001538595	-1.29836266

FAS	Tumor necrosis factor receptor superfamily, member 6 isoform 1 variant (Fragment) gene:ENSG00000026103 /// Isoform 6 of Tumor necrosis factor receptor superfamily member 6 gene:ENSG00000026103 /// 38 kDa protein gene:ENSG00000026103 /// Isoform 4 of Tumor necrosis factor receptor superfamily member 6 gene:ENSG00000026103 /// Isoform 4 of Tumor necrosis factor receptor superfamily member 6 gene:ENSG00000026103 /// Isoform 2 of Tumor necrosis factor receptor superfamily member 6 gene:ENSG00000026103 /// Isoform 5 of Tumor necrosis factor receptor superfamily member 6 gene:ENSG00000026103 /// Truncated tumor necrosis factor receptor superfamily member 6 gene:ENSG00000026103 /// Isoform 1 of Tumor necrosis factor receptor superfamily member 6 gene:ENSG00000026103 /// Isoform 3 of Tumor necrosis factor receptor superfamily member 6 gene:ENSG00000026103 /// Homo sapiens Fas (TNF receptor superfamily, member 6), mRNA (cDNA clone MGC:21432 IMAGE:4514272), complete cds. /// Homo sapiens Fas (TNF receptor superfamily, member 6) (FAS), transcript variant 1, mRNA. /// Homo sapiens Fas (TNF receptor superfamily, member 6) (FAS), transcript variant 2, mRNA. /// Homo sapiens Fas (TNF receptor superfamily, member 6) (FAS), transcript variant 3, mRNA. /// Homo sapiens Fas (TNF receptor superfamily, member 6) (FAS), transcript variant 4, mRNA. /// Homo sapiens Fas (TNF receptor superfamily, member 6) (FAS), transcript variant 8, mRNA. /// Homo	Fas (TNF receptor superfamily, member 6)	8.13415E-06	-1.318693504

	sapiens Fas (TNF receptor superfamily, member 6) (FAS), transcript variant 5, mRNA. /// Homo sapiens Fas (TNF receptor superfamily, member 6) (FAS), transcript variant 6, mRNA. /// Homo sapiens Fas (TNF receptor superfamily, member 6) (FAS), transcript variant 7, mRNA.			
GABBR2	Gamma-aminobutyric acid type B receptor subunit 2 gene:ENSG00000136928 /// Homo sapiens GABA-B receptor mRNA, complete cds. /// Homo sapiens gamma-aminobutyric acid (GABA) B receptor, 2 (GABBR2), mRNA.	gamma-aminobutyric acid (GABA) B receptor, 2	0.000211238	-1.423400991
CD83	CD83 antigen gene:ENSG00000112149 /// Homo sapiens CD83 molecule, mRNA (cDNA clone MGC:26322 IMAGE:4818856), complete cds. /// Homo sapiens CD83 molecule (CD83), transcript variant 1, mRNA. /// Homo sapiens CD83 molecule (CD83), transcript variant 2, mRNA.	CD83 molecule	0.009333693	-1.200761833

KDR	Vascular endothelial growth factor receptor 2 gene:ENSG00000128052 /// Homo sapiens KDR/flk-1 protein mRNA, complete cds. /// Homo sapiens kinase insert domain receptor (a type III receptor tyrosine kinase) (KDR), mRNA.	kinase insert domain receptor (a type III receptor tyrosine kinase)	0.002021804	-1.176761484
TNFRSF10A	Tumor necrosis factor receptor superfamily member 10A gene:ENSG00000104689 /// Homo sapiens tumor necrosis factor receptor superfamily, member 10a, mRNA (cDNA clone MGC:9365 IMAGE:3857315), complete cds. /// Homo sapiens tumor necrosis factor receptor superfamily, member 10a (TNFRSF10A), mRNA.	tumor necrosis factor receptor superfamily, member 10a	0.000301718	-1.327680885
GP1BB	Human glycoprotein Ib beta mRNA, complete cds.	glycoprotein Ib (platelet), beta polypeptide	0.390110093	-1.073658187
TMPRSS11E	Transmembrane protease, serine 11E gene:ENSG00000087128 /// Transmembrane protease, serine 11E2 gene:ENSG00000173487 /// Homo sapiens cDNA FLJ75331 complete cds, highly similar to Homo sapiens serine protease mRNA. /// Homo sapiens clone DNA68871 serine protease (UNQ742) mRNA, complete cds. /// Homo sapiens transmembrane protease, serine 11E (TMPRSS11E), mRNA.	transmembrane protease, serine 11E2 /// transmembrane protease, serine 11E	2.20E-06	-1.554814264
DSC3	Isoform 3A of Desmocollin-3 gene:ENSG00000134762 /// Isoform 3B of Desmocollin-3 gene:ENSG00000134762 /// Homo sapiens mRNA for desmocollin type 4, complete cds. /// Homo sapiens desmocollin 3 (DSC3), transcript variant Dsc3a, mRNA. /// Homo sapiens desmocollin 3 (DSC3), transcript variant Dsc3b, mRNA.	desmocollin 3	5.21652E-09	-1.8865042

GPR50	G protein-coupled receptor 50 gene:ENSG00000102195 /// Human melatonin-related receptor mRNA, complete cds. /// Homo sapiens G protein-coupled receptor 50 (GPR50), mRNA.	G protein-coupled receptor 50	0.000133863	-1.454577025
GRPR	Gastrin-releasing peptide receptor gene:ENSG00000126010 /// Human gastrin releasing peptide receptor (GRPR) mRNA, complete cds. /// Homo sapiens gastrin-releasing peptide receptor (GRPR), mRNA.	gastrin-releasing peptide receptor	1.80275E-05	1.367066636
PTGER4	Prostaglandin E2 receptor EP4 subtype gene:ENSG00000171522 /// Homo sapiens prostaglandin E2 receptor EP2 subtype mRNA, complete cds. /// Homo sapiens prostaglandin E receptor 4 (subtype EP4) (PTGER4), mRNA.	prostaglandin E receptor 4 (subtype EP4)	0.000576642	-1.37325741
CXCR4	cdna:known chromosome:NCBI36:2:136588389:136590283:-1 gene:ENSG00000121966 /// Isoform 2 of C-X-C chemokine receptor type 4 gene:ENSG00000121966 /// Homo sapiens chemokine receptor CXCR4-Lo (CXCR4) mRNA, alternatively spliced, complete cds. /// Homo sapiens chemokine (C-X-C motif) receptor 4 (CXCR4), transcript variant 1, mRNA. /// Homo sapiens chemokine (C-X-C motif) receptor 4 (CXCR4), transcript variant 2, mRNA.	chemokine (C-X-C motif) receptor 4	0.000686099	1.285260181
GPR25	Probable G-protein coupled receptor 25 gene:ENSG00000170128 /// Homo sapiens G protein-coupled receptor 25, mRNA (cDNA clone MGC:161664 IMAGE:8992102), complete cds. /// Homo sapiens G protein-coupled receptor 25 (GPR25), mRNA.	G protein-coupled receptor 25	0.000683671	-1.402211558

CA14	Carbonic anhydrase 14 gene:ENSG00000118298 /// Homo sapiens cDNA FLJ90284 fis, clone NT2RP1000613, weakly similar to CARBONIC ANHYDRASE VI (EC 4.2.1.1). /// Homo sapiens carbonic anhydrase XIV (CA14), mRNA.	carbonic anhydrase XIV	0.000351389	1.233556271
GLIPR1	Glioma pathogenesis-related protein 1 gene:ENSG00000139278 /// Homo sapiens GLI pathogenesis-related 1, mRNA (cDNA clone MGC:21203 IMAGE:4453841), complete cds. /// Homo sapiens GLI pathogenesis-related 1 (GLIPR1), mRNA.	GLI pathogenesis-related 1	0.001216405	-1.219278501
CEACAM1	Putative uncharacterized protein CEACAM1 gene:ENSG00000079385 /// Isoform D of Carcinoembryonic antigen-related cell adhesion molecule 1 gene:ENSG00000079385 /// Isoform C of Carcinoembryonic antigen-related cell adhesion molecule 1 gene:ENSG00000079385 /// Isoform B of Carcinoembryonic antigen-related cell adhesion molecule 1 gene:ENSG00000079385 /// Isoform A of Carcinoembryonic antigen-related cell adhesion molecule 1 gene:ENSG00000079385 /// Biliary glycoprotein gene:ENSG00000079385 /// carcinoembryonic antigen-related cell adhesion molecule 1 isoform 2 precursor gene:ENSG00000079385 /// Putative carcinoembryonic antigen-related cell adhesion molecule 1 short form 3 gene:ENSG00000079385 /// BGP protein gene:ENSG00000079385 /// Carcinoembryonic antigen-related cell adhesion molecule 1 (Biliary glycoprotein), isoform CRA_g gene:ENSG00000079385 /// Putative uncharacterized protein CEACAM1 gene:ENSG00000079385 /// Isoform I of Carcinoembryonic antigen-related cell adhesion molecule 1	carcinoembryonic antigen-related cell adhesion molecule 1 (biliary glycoprotein)	1.21086E-05	-1.484050255

	gene:ENSG00000079385 /// Isoform H of Carcinoembryonic antigen-related cell adhesion molecule 1 gene:ENSG00000079385 /// Human biliary glycoprotein I (BGP I) mRNA, complete cds. /// Homo sapiens carcinoembryonic antigen-related cell adhesion molecule 1 (biliary glycoprotein) (CEACAM1), transcript variant 1, mRNA. /// Homo sapiens carcinoembryonic antigen-related cell adhesion molecule 1 (biliary glycoprotein) (CEACAM1), transcript variant 2, mRNA.			
CD99	Isoform I of CD99 antigen gene:ENSG00000002586 /// Putative uncharacterized protein CD99 gene:ENSG00000002586 /// CD99 antigen isoform b precursor gene:ENSG00000002586 /// Putative uncharacterized protein CD99 gene:ENSG00000002586 /// Putative uncharacterized protein CD99 gene:ENSG00000002586 /// Homo sapiens CD99 molecule, mRNA (cDNA clone MGC:19734 IMAGE:3606974), complete cds. /// Homo sapiens CD99 molecule (CD99), transcript variant 1, mRNA. /// Homo sapiens CD99 molecule (CD99), transcript variant 2, mRNA.	CD99 molecule	0.004074288	1.190899882

NETO2	Isoform 1 of Neuropilin and tolloid-like protein 2 gene:ENSG00000171208 /// Homo sapiens clone DNA84912 Neto2 (UNQ1926) mRNA, complete cds. /// Homo sapiens neuropilin (NRP) and tolloid (TLL)-like 2 (NETO2), mRNA.	neuropilin (NRP) and tolloid (TLL)-like 2	1.91967E-06	-1.335000414
LANCL1	LanC-like protein 1 gene:ENSG00000115365 /// Homo sapiens LanC lantibiotic synthetase component C-like 1 (bacterial), mRNA (cDNA clone MGC:26280 IMAGE:4829574), complete cds. /// Homo sapiens LanC lantibiotic synthetase component C-like 1 (bacterial) (LANCL1), transcript variant 3, mRNA. /// Homo sapiens LanC lantibiotic synthetase component C-like 1 (bacterial) (LANCL1), transcript variant 1, mRNA. /// Homo sapiens LanC lantibiotic synthetase component C-like 1 (bacterial) (LANCL1), transcript variant 2, mRNA.	LanC lantibiotic synthetase component C-like 1 (bacterial)	0.005249954	1.171764212
DKFZP564O0823	Protein PARM-1 gene:ENSG00000169116 /// Homo sapiens clone DNA92223 VYKT1879 (UNQ1879) mRNA, complete cds. /// Homo sapiens DKFZP564O0823 protein (DKFZP564O0823), mRNA.	DKFZP564O0823 protein	5.61324E-05	-1.348721612
CD9	CD9 antigen gene:ENSG00000010278 /// CD9 antigen gene:ENSG00000010278 /// Bladder cancer related CD9 variant gene:ENSG00000010278 /// Putative uncharacterized protein CD9 gene:ENSG00000010278 /// Homo sapiens bladder cancer related CD9 variant (BTCC-1) mRNA, complete cds. /// Homo sapiens CD9 molecule (CD9), mRNA.	CD9 molecule	0.005874662	1.178748245

LRRN1	Leucine-rich repeat neuronal protein 1 gene:ENSG00000175928 /// Homo sapiens leucine rich repeat neuronal 1, mRNA (cDNA clone MGC:26098 IMAGE:4826337), complete cds. /// Homo sapiens leucine rich repeat neuronal 1 (LRRN1), mRNA.	leucine rich repeat neuronal 1	0.002870616	1.17794733
MPZL1	Isoform 3 of Myelin protein zero-like protein 1 gene:ENSG00000197965 /// Isoform 1 of Myelin protein zero-like protein 1 gene:ENSG00000197965 /// Myelin protein zero-like 1 gene:ENSG00000197965 /// Isoform 3 of Myelin protein zero-like protein 1 gene:ENSG00000197965 /// Homo sapiens clone DNA76510 MPZL1 (UNQ849) mRNA, complete cds. /// Homo sapiens myelin protein zero-like 1 (MPZL1), transcript variant 1, mRNA. /// Homo sapiens myelin protein zero-like 1 (MPZL1), transcript variant 2, mRNA.	myelin protein zero-like 1	0.003136446	1.168400169
FZD5	Frizzled-5 gene:ENSG00000163251 /// Homo sapiens FZD5 mRNA for seven-transmembrane receptor Frizzled-5, complete cds. /// Homo sapiens frizzled homolog 5 (Drosophila) (FZD5), mRNA.	frizzled homolog 5 (Drosophila)	0.000188846	1.237215483

SEMA6D	Isoform 7 of Semaphorin-6D gene:ENSG00000137872 /// semaphorin 6D isoform 5 precursor gene:ENSG00000137872 /// Isoform 4 of Semaphorin-6D gene:ENSG00000137872 /// Isoform 1 of Semaphorin-6D gene:ENSG00000137872 /// Isoform 5 of Semaphorin-6D gene:ENSG00000137872 /// Isoform 6 of Semaphorin-6D gene:ENSG00000137872 /// Isoform 3 of Semaphorin-6D gene:ENSG00000137872 /// Isoform 2 of Semaphorin-6D gene:ENSG00000137872 /// Homo sapiens semaphorin 6D isoform 4 (SEMA6D) mRNA, complete cds; alternatively spliced. /// Homo sapiens sema domain, transmembrane domain (TM), and cytoplasmic domain, (semaphorin) 6D (SEMA6D), transcript variant 6, mRNA. /// Homo sapiens sema domain, transmembrane domain (TM), and cytoplasmic domain, (semaphorin) 6D (SEMA6D), transcript variant 2, mRNA. /// Homo sapiens sema domain, transmembrane domain (TM), and cytoplasmic domain, (semaphorin) 6D (SEMA6D), transcript variant 3, mRNA. /// Homo sapiens sema domain, transmembrane domain (TM), and cytoplasmic domain, (semaphorin) 6D (SEMA6D), transcript variant 1, mRNA. /// Homo sapiens sema domain, transmembrane domain (TM), and cytoplasmic domain, (semaphorin) 6D (SEMA6D), transcript variant 4, mRNA. /// Homo sapiens sema domain, transmembrane domain (TM), and cytoplasmic domain, (semaphorin) 6D (SEMA6D), transcript variant 5, mRNA.	sema domain, transmembrane domain (TM), and cytoplasmic domain, (semaphorin) 6D	0.000561386	1.445633375

GRM4	Metabotropic glutamate receptor 4 gene:ENSG00000124493 /// cDNA FLJ16766 fis, clone BRAWH3013197, highly similar to Metabotropic glutamate receptor 4 gene:ENSG00000124493 /// Homo sapiens cDNA FLJ78562 complete cds, highly similar to Homo sapiens glutamate receptor, metabotropic 4 (GRM4), mRNA. /// Homo sapiens glutamate receptor, metabotropic 4 (GRM4), mRNA.	glutamate receptor, metabotropic 4	0.002429438	-1.21928761
DSG3	Desmoglein-3 gene:ENSG00000134757 /// Human 130-kD pemphigus vulgaris antigen mRNA, complete cds. /// Homo sapiens desmoglein 3 (pemphigus vulgaris antigen) (DSG3), mRNA.	desmoglein 3 (pemphigus vulgaris antigen)	0.000556576	-1.547738515
CDH8	Cadherin-8 gene:ENSG00000150394 /// Putative uncharacterized protein CDH8 gene:ENSG00000150394 /// Homo sapiens cDNA FLJ42744 fis, clone BRAWH3000088, highly similar to CADHERIN-8 PRECURSOR. /// Homo sapiens cadherin 8, type 2 (CDH8), mRNA.	cadherin 8, type 2	1.36429E-05	-1.344971305
PRRG4	Transmembrane gamma-carboxyglutamic acid protein 4 gene:ENSG00000135378 /// Homo sapiens proline rich Gla (G-carboxyglutamic acid) 4 (transmembrane), mRNA (cDNA clone MGC:74683 IMAGE:5183337), complete cds. /// Homo sapiens proline rich Gla (G-carboxyglutamic acid) 4 (transmembrane) (PRRG4), mRNA.	proline rich Gla (G-carboxyglutamic acid) 4 (transmembrane)	0.007345094	-1.283143148
TYRO3	Tyrosine-protein kinase receptor TYRO3 gene:ENSG00000092445 /// Homo sapiens mRNA for Sky, complete cds. /// Homo sapiens TYRO3 protein tyrosine kinase (TYRO3), mRNA.	TYRO3 protein tyrosine kinase	0.000354804	1.231611604

RPRM	Protein reprimo gene:ENSG00000177519 /// Homo sapiens reprimo, TP53 dependent G2 arrest mediator candidate, mRNA (cDNA clone MGC:11260 IMAGE:3942270), complete cds. /// Homo sapiens reprimo, TP53 dependent G2 arrest mediator candidate (RPRM), mRNA.	reprimo, TP53 dependent G2 arrest mediator candidate	0.005161311	1.194146899
PVRL1	Isoform Alpha of Poliovirus receptor-related protein 1 gene:ENSG00000110400 /// Isoform Delta of Poliovirus receptor-related protein 1 gene:ENSG00000110400 /// Isoform Gamma of Poliovirus receptor-related protein 1 gene:ENSG00000110400 /// Homo sapiens herpesvirus entry protein C (HVEC) mRNA, complete cds. /// Homo sapiens poliovirus receptor-related 1 (herpesvirus entry mediator C) (PVRL1), transcript variant 1, mRNA. /// Homo sapiens poliovirus receptor-related 1 (herpesvirus entry mediator C) (PVRL1), transcript variant 2, mRNA. /// Homo sapiens poliovirus receptor-related 1 (herpesvirus entry mediator C) (PVRL1), transcript variant 3, mRNA.	poliovirus receptor-related 1 (herpesvirus entry mediator C)	0.004707814	-1.182618931
LPHN3	Isoform 3 of Latrophilin-3 gene:ENSG00000150471 /// Isoform 1 of Latrophilin-3 gene:ENSG00000150471 /// latrophilin 3 precursor gene:ENSG00000150471 /// Homo sapiens latrophilin 3 (LPHN3), mRNA.	latrophilin 3	0.005154898	-1.217792516
HAS1	Hyaluronan synthase 1 gene:ENSG00000105509 /// Hyaluronan synthase 1 variant Va gene:ENSG00000105509 /// Hyaluronan synthase 1 variant Vb gene:ENSG00000105509 /// Homo sapiens hyaluronan synthase 1, mRNA (cDNA clone MGC:46218 IMAGE:5589083), complete cds. /// Homo sapiens hyaluronan synthase 1 (HAS1), mRNA.	hyaluronan synthase 1	0.003500202	-1.247958981

HAS2	Hyaluronan synthase 2 gene:ENSG00000170961 /// Human Has2 mRNA, complete cds. /// Homo sapiens hyaluronan synthase 2 (HAS2), mRNA.	hyaluronan synthase 2	0.000787201	-1.275012183
PTPRM	Receptor-type tyrosine-protein phosphatase mu gene:ENSG00000173482 /// PTPRM protein gene:ENSG00000173482 /// cDNA FLJ55898, highly similar to Receptor-type tyrosine-protein phosphatase mu gene:ENSG00000173482 /// Homo sapiens protein tyrosine phosphatase, receptor type, M, mRNA (cDNA clone MGC:166994 IMAGE:8860327), complete cds. /// Homo sapiens protein tyrosine phosphatase, receptor type, M (PTPRM), transcript variant 2, mRNA. /// Homo sapiens protein tyrosine phosphatase, receptor type, M (PTPRM), transcript variant 1, mRNA.	protein tyrosine phosphatase, receptor type, M	0.001477125	-1.226370184
PTPRE	Receptor-type tyrosine-protein phosphatase epsilon precursor gene:ENSG00000132334 /// Isoform 2 of Receptor-type tyrosine-protein phosphatase epsilon gene:ENSG00000132334 /// Homo sapiens protein tyrosine phosphatase, receptor type, E, mRNA (cDNA clone MGC:48280 IMAGE:5274802), complete cds. /// Homo sapiens protein tyrosine phosphatase, receptor type, E (PTPRE), transcript variant 2, mRNA. /// Homo sapiens protein tyrosine phosphatase, receptor type, E (PTPRE), transcript variant 1, mRNA.	protein tyrosine phosphatase, receptor type, E	3.81593E-06	-1.667249907

MDGA2	MAM domain-containing glycosylphosphatidylinositol anchor protein 2 gene:ENSG00000139915 /// MAM domain containing 1 isoform 2 gene:ENSG00000139915 /// MAM domain containing 1 isoform 2 gene:ENSG00000139915 /// Homo sapiens MAM domain-containing glycosylphosphatidylinositol anchor 2 (MDGA2) mRNA, complete cds. /// Homo sapiens MAM domain containing glycosylphosphatidylinositol anchor 2 (MDGA2), transcript variant 2, mRNA. /// Homo sapiens MAM domain containing glycosylphosphatidylinositol anchor 2 (MDGA2), transcript variant 1, mRNA.	MAM domain containing glycosylphosphatidylinositol anchor 2	0.002967012	-1.356547479
PVRL3	Isoform 1 of Poliovirus receptor-related protein 3 gene:ENSG00000177707 /// cDNA FLJ90624 fis, clone PLACE1002911, weakly similar to POLIOVIRUS RECEPTOR HOMOLOG gene:ENSG00000177707 /// Homo sapiens cDNA FLJ90624 fis, clone PLACE1002911, weakly similar to POLIOVIRUS RECEPTOR HOMOLOG PRECURSOR. /// Homo sapiens poliovirus receptor-related 3 (PVRL3), mRNA.	poliovirus receptor-related 3	0.008995663	1.155047855

SLC44A5	Isoform 2 of Choline transporter-like protein 5 gene:ENSG00000137968 /// Isoform 2 of Choline transporter-like protein 5 gene:ENSG00000137968 /// Isoform 1 of Choline transporter-like protein 5 gene:ENSG00000137968 /// Putative uncharacterized protein SLC44A5 gene:ENSG00000137968 /// Homo sapiens solute carrier family 44, member 5, mRNA (cDNA clone MGC:42574 IMAGE:4824861), complete cds. /// Homo sapiens solute carrier family 44, member 5 (SLC44A5), transcript variant 1, mRNA. /// Homo sapiens solute carrier family 44, member 5 (SLC44A5), transcript variant 2, mRNA.	solute carrier family 44, member 5	0.001243544	-1.364925707
TMEM9	Transmembrane protein 9 gene:ENSG00000116857 /// Transmembrane protein 9 gene:ENSG00000116857 /// Transmembrane protein 9 gene:ENSG00000116857 /// Transmembrane protein 9 gene:ENSG00000116857 /// Transmembrane protein 9 gene:ENSG00000116857 /// Transmembrane protein 9 gene:ENSG00000116857 /// 16 kDa protein gene:ENSG00000116857 /// Homo sapiens cDNA FLJ34407 fis, clone HEART1000173. /// Homo sapiens transmembrane protein 9 (TMEM9), mRNA.	transmembrane protein 9	0.001251575	1.213576556
LGR5	Leucine-rich repeat-containing G-protein coupled receptor 5 gene:ENSG00000139292 /// Homo sapiens orphan G protein-coupled receptor HG38 mRNA, complete cds. /// Homo sapiens leucine-rich repeat-containing G protein-coupled receptor 5 (LGR5), mRNA.	leucine-rich repeat-containing G protein-coupled receptor 5	4.09021E-05	1.573148422

EMP3	Epithelial membrane protein 3 gene:ENSG00000142227 /// Homo sapiens epithelial membrane protein 3, mRNA (cDNA clone MGC:9511 IMAGE:3891050), complete cds. /// Homo sapiens epithelial membrane protein 3 (EMP3), mRNA.	epithelial membrane protein 3	0.006650587	1.18997891
SEZ6L2	Isoform 3 of Seizure 6-like protein 2 gene:ENSG00000174938 /// Isoform 1 of Seizure 6-like protein 2 gene:ENSG00000174938 /// seizure related 6 homolog (mouse)-like 2 isoform 4 gene:ENSG00000174938 /// Homo sapiens clone DNA84909 PSK-1 (UNQ1903) mRNA, complete cds. /// Homo sapiens seizure related 6 homolog (mouse)-like 2 (SEZ6L2), transcript variant 2, mRNA. /// Homo sapiens seizure related 6 homolog (mouse)-like 2 (SEZ6L2), transcript variant 4, mRNA. /// Homo sapiens seizure related 6 homolog (mouse)-like 2 (SEZ6L2), transcript variant 3, mRNA. /// Homo sapiens seizure related 6 homolog (mouse)-like 2 (SEZ6L2), transcript variant 1, mRNA.	seizure related 6 homolog (mouse)-like 2	0.005945588	1.180542277
ASAM	Adipocyte adhesion molecule gene:ENSG00000166250 /// Uncharacterized protein ENSP00000376525 (Fragment) gene:ENSG00000166250 /// Homo sapiens adipocyte adhesion molecule mRNA, complete cds. /// Homo sapiens adipocyte-specific adhesion molecule (ASAM), mRNA.	adipocyte-specific adhesion molecule	0.004035831	1.172514888
OR52A1	Olfactory receptor 52A1 gene:ENSG00000182070 /// Olfactory receptor 52A1 gene:ENSG00000182070 /// Homo sapiens olfactory receptor, family 52, subfamily A, member 1 (OR52A1), mRNA.	olfactory receptor, family 52, subfamily A, member 1	0.000979942	-1.468881348

CNTNAP3	cdna:known supercontig::NT_113912:33219:143176:-1 gene:ENSG00000219070 /// Isoform 1 of Contactin-associated protein-like 3 gene:ENSG00000106714 /// Putative uncharacterized protein CNTNAP3 gene:ENSG00000106714 /// cDNA FLJ58810, highly similar to Contactin-associated protein-like 3 gene:ENSG00000106714 /// Contactin associated protein-like 3 gene:ENSG00000106714 /// Putative uncharacterized protein CNTNAP3 gene:ENSG00000106714 /// Contactin associated protein-like 3 gene:ENSG00000106714 /// Contactin associated protein-like 3 gene:ENSG00000106714 /// cdna:known chromosome:NCBI36:9:40297346:40329528:1 gene:ENSG00000185020 /// 81 kDa protein gene:ENSG00000154529 /// Protein gene:ENSG00000154529 /// Isoform 1 of Contactin-associated protein-like 3B gene:ENSG00000154529 /// hypothetical protein gene:ENSG00000154529 /// Isoform 3 of Contactin-associated protein-like 3B gene:ENSG00000154529 /// Isoform 2 of Contactin-associated protein-like 3B gene:ENSG00000154529 /// hypothetical protein gene:ENSG00000188377 /// similar to cell recognition molecule CASPR3 gene:ENSG00000188377 /// hypothetical protein gene:ENSG00000204798 /// similar to cell recognition molecule CASPR3 gene:ENSG00000204798 /// Homo sapiens cell recognition molecule CASPR3 mRNA, complete cds. /// Homo sapiens cDNA FLJ30083 fis, clone BGGI12001097, weakly similar to Homo sapiens contactin associated protein (Caspr) mRNA. /// Homo	contactin associated protein-like 3 /// similar to cell recognition molecule CASPR3	0.025225101	-1.147983011

	sapiens contactin associated protein-like 3 (CNTNAP3), mRNA.			

OR4F3	Olfactory receptor 4F21 gene:ENSG00000176269 /// Olfactory receptor 4F3/4F16/4F29 gene:ENSG00000183127 /// Olfactory receptor 4F3/4F16/4F29 gene:ENSG00000177799 /// Olfactory receptor 4F3/4F16/4F29 gene:ENSG00000185097 /// Homo sapiens olfactory receptor, family 4, subfamily F, member 29, mRNA (cDNA clone MGC:169170 IMAGE:9021547), complete cds. /// Homo sapiens olfactory receptor, family 4, subfamily F, member 21 (OR4F21), mRNA. /// Homo sapiens olfactory receptor, family 4, subfamily F, member 29 (OR4F29), mRNA. /// Homo sapiens olfactory receptor, family 4, subfamily F, member 3 (OR4F3), mRNA. /// Homo sapiens olfactory receptor, family 4, subfamily F, member 16 (OR4F16), mRNA.	olfactory receptor, family 4, subfamily F, member 3 /// olfactory receptor, family 4, subfamily F, member 16 /// olfactory receptor, family 4, subfamily F, member 29 /// olfactory receptor, family 4, subfamily F, member 21	0.002942 496	- 1.387172 027
SLC24A6	Isoform 1 of Sodium/potassium/calcium exchanger 6 gene:ENSG00000089060 /// Isoform 2 of Sodium/potassium/calcium exchanger 6 gene:ENSG00000089060 /// Homo sapiens solute carrier family 24 (sodium/potassium/calcium exchanger), member 6, mRNA (cDNA clone MGC:120605 IMAGE:40026360), complete cds. /// Homo sapiens solute carrier family 24 (sodium/potassium/calcium exchanger), member 6 (SLC24A6), mRNA.	solute carrier family 24 (sodium/potassium/calcium exchanger), member 6	0.008297 168	- 1.172206 281
TMED4	Isoform 1 of Transmembrane emp24 domain-containing protein 4 gene:ENSG00000158604 /// Homo sapiens transmembrane emp24 protein transport domain containing 4, mRNA (cDNA clone IMAGE:5184403). /// Homo sapiens transmembrane emp24 protein transport domain containing 4 (TMED4), mRNA.	transmembrane emp24 protein transport domain containing 4	0.006377 635	1.175563 905

LRFN5	Leucine-rich repeat and fibronectin type-III domain-containing protein 5 gene:ENSG00000165379 /// Putative uncharacterized protein LRFN5 gene:ENSG00000165379 /// Homo sapiens cDNA FLJ56792 complete cds, highly similar to Leucine-rich repeat and fibronectin type-III domain-containing protein 5 precursor. /// Homo sapiens leucine rich repeat and fibronectin type III domain containing 5 (LRFN5), mRNA.	leucine rich repeat and fibronectin type III domain containing 5	0.00036211	-1.41648526
LRIG3	Isoform 1 of Leucine-rich repeats and immunoglobulin-like domains protein 3 gene:ENSG00000139263 /// Isoform 2 of Leucine-rich repeats and immunoglobulin-like domains protein 3 gene:ENSG00000139263 /// Homo sapiens clone DNA37140 SAPS287 (UNQ287) mRNA, complete cds. /// Homo sapiens leucine-rich repeats and immunoglobulin-like domains 3 (LRIG3), transcript variant 1, mRNA. /// Homo sapiens leucine-rich repeats and immunoglobulin-like domains 3 (LRIG3), transcript variant 2, mRNA.	leucine-rich repeats and immunoglobulin-like domains 3	6.0119E-05	1.364636467
ICOSLG	Isoform 1 of ICOS ligand gene:ENSG00000160223 /// Isoform 2 of ICOS ligand gene:ENSG00000160223 /// Isoform 2 of ICOS ligand gene:ENSG00000160223 /// Putative uncharacterized protein ICOSLG gene:ENSG00000160223 /// Putative uncharacterized protein ICOSLG gene:ENSG00000160223 /// Isoform 1 of ICOS ligand gene:ENSG00000160223 /// Homo sapiens mRNA for KIAA0653 protein, partial cds. /// Homo sapiens inducible T-cell co-stimulator ligand (ICOSLG), mRNA.	inducible T-cell co-stimulator ligand	0.009116454	-1.21641206

LRRTM4	cdna:known chromosome:NCBI36:2:76828353:77602973:-1 gene:ENSG00000176204 /// cdna:known chromosome:NCBI36:2:76829187:77602907:-1 gene:ENSG00000176204 /// cdna:known chromosome:NCBI36:2:76829192:77603002:-1 gene:ENSG00000176204 /// Isoform 1 of Leucine-rich repeat transmembrane neuronal protein 4 gene:ENSG00000176204 /// cdna:known chromosome:NCBI36:2:77597302:77603010:-1 gene:ENSG00000176204 /// cdna:known chromosome:NCBI36:2:77598150:77602844:-1 gene:ENSG00000176204 /// Homo sapiens clone DNA142392 GFHL3075 (UNQ3075) mRNA, complete cds. /// Homo sapiens leucine rich repeat transmembrane neuronal 4 (LRRTM4), transcript variant 1, mRNA. /// Homo sapiens leucine rich repeat transmembrane neuronal 4 (LRRTM4), transcript variant 2, mRNA.	leucine rich repeat transmembrane neuronal 4	0.00327488	-1.221016835
GPR158	Probable G-protein coupled receptor 158 gene:ENSG00000151025 /// Homo sapiens G protein coupled receptor 158 (GPR158) mRNA, complete cds. /// Homo sapiens G protein-coupled receptor 158 (GPR158), mRNA.	G protein-coupled receptor 158	0.008561092	-1.287914435
C9orf46	Transmembrane protein C9orf46 gene:ENSG00000107020 /// Homo sapiens uncharacterized hematopoietic stem/progenitor cells protein MDS030 mRNA, complete cds. /// Homo sapiens AD025 mRNA, complete cds.	chromosome 9 open reading frame 46	0.003702152	1.261456637
TCTN2	Tectonic-2 gene:ENSG00000168778 /// Homo sapiens TECT2 (TECT2) mRNA, complete cds. /// Homo sapiens tectonic family member 2 (TCTN2), mRNA.	tectonic family member 2	0.003700585	1.224374462

C6orf162	Uncharacterized protein C6orf162 gene:ENSG00000111850 /// Uncharacterized protein C6orf162 gene:ENSG00000111850 /// Homo sapiens chromosome 6 open reading frame 162, mRNA (cDNA clone MGC:88247 IMAGE:6452586), complete cds. /// Homo sapiens chromosome 6 open reading frame 162 (C6orf162), transcript variant 2, mRNA. /// Homo sapiens chromosome 6 open reading frame 162 (C6orf162), transcript variant 1, mRNA.	chromosome 6 open reading frame 162	0.003066727	1.199326745
GPR63	Probable G-protein coupled receptor 63 gene:ENSG00000112218 /// Probable G-protein coupled receptor 63 gene:ENSG00000112218 /// Homo sapiens mRNA for PSP24, complete cds. /// Homo sapiens G protein-coupled receptor 63 (GPR63), mRNA.	G protein-coupled receptor 63	0.006011659	1.18859154
GPR172A	Porcine endogenous retrovirus A receptor 1 gene:ENSG00000185803 /// Porcine endogenous retrovirus A receptor 1 gene:ENSG00000185803 /// Homo sapiens cDNA FLJ11856 fis, clone HEMBA1006789. /// Homo sapiens G protein-coupled receptor 172A (GPR172A), mRNA.	G protein-coupled receptor 172A	0.002766543	1.174820046
**Functional Group 46**	**Transcript Description**	**Gene Title**	**Bayes.lnp**	**Fold**

FBXW7	Isoform 4 of F-box/WD repeat-containing protein 7 gene:ENSG00000109670 /// Isoform 1 of F-box/WD repeat-containing protein 7 gene:ENSG00000109670 /// Isoform 2 of F-box/WD repeat-containing protein 7 gene:ENSG00000109670 /// Isoform 1 of F-box/WD repeat-containing protein 7 gene:ENSG00000109670 /// Homo sapiens archipelago alpha form mRNA, complete cds. /// Homo sapiens F-box and WD repeat domain containing 7 (FBXW7), transcript variant 1, mRNA. /// Homo sapiens F-box and WD repeat domain containing 7 (FBXW7), transcript variant 3, mRNA. /// Homo sapiens F-box and WD repeat domain containing 7 (FBXW7), transcript variant 2, mRNA.	F-box and WD repeat domain containing 7	0.000408298	-1.278626563
WDR6	WD repeat domain 6 protein gene:ENSG00000178252 /// WD repeat-containing protein 6 gene:ENSG00000178252 /// cDNA FLJ10218 fis, clone HEMBA1006936 gene:ENSG00000178252 /// Homo sapiens WD-repeat protein 6 (WDR6) mRNA, complete cds. /// Homo sapiens WD repeat domain 6 (WDR6), mRNA.	WD repeat domain 6	0.001638454	1.179558541

PPP2R2C	gamma isoform of regulatory subunit B55, protein phosphatase 2 isoform b gene:ENSG00000074211 /// Protein phosphatase 2 (Formerly 2A), regulatory subunit B (PR 52), gamma isoform, isoform CRA_b gene:ENSG00000074211 /// Serine/threonine-protein phosphatase 2A 55 kDa regulatory subunit B gamma isoform gene:ENSG00000074211 /// Homo sapiens protein phosphatase 2A1 B gamma subunit mRNA, complete cds. /// Homo sapiens protein phosphatase 2 (formerly 2A), regulatory subunit B, gamma isoform (PPP2R2C), transcript variant 1, mRNA. /// Homo sapiens protein phosphatase 2 (formerly 2A), regulatory subunit B, gamma isoform (PPP2R2C), transcript variant 2, mRNA.	protein phosphatase 2 (formerly 2A), regulatory subunit B, gamma isoform	0.000746615	-1.256320113

PPP2R2B	Serine/threonine-protein phosphatase 2A 55 kDa regulatory subunit B beta isoform gene:ENSG00000156475 /// Serine/threonine-protein phosphatase 2A 55 kDa regulatory subunit B beta isoform gene:ENSG00000156475 /// Serine/threonine-protein phosphatase 2A 55 kDa regulatory subunit B beta isoform gene:ENSG00000156475 /// beta isoform of regulatory subunit B55, protein phosphatase 2 isoform d gene:ENSG00000156475 /// Serine/threonine-protein phosphatase 2A 55 kDa regulatory subunit B beta isoform gene:ENSG00000156475 /// Serine/threonine-protein phosphatase 2A 55 kDa regulatory subunit B beta isoform gene:ENSG00000156475 /// beta isoform of regulatory subunit B55, protein phosphatase 2 isoform b gene:ENSG00000156475 /// Human protein phosphatase 2A beta subunit mRNA, complete cds. /// Homo sapiens protein phosphatase 2 (formerly 2A), regulatory subunit B, beta isoform (PPP2R2B), transcript variant 6, mRNA. /// Homo sapiens protein phosphatase 2 (formerly 2A), regulatory subunit B, beta isoform (PPP2R2B), transcript variant 7, mRNA. /// Homo sapiens protein phosphatase 2 (formerly 2A), regulatory subunit B, beta isoform (PPP2R2B), transcript variant 1, mRNA. /// Homo sapiens protein phosphatase 2 (formerly 2A), regulatory subunit B, beta isoform (PPP2R2B), transcript variant 2, mRNA. /// Homo sapiens protein phosphatase 2 (formerly 2A), regulatory subunit B, beta isoform (PPP2R2B), transcript variant 3, mRNA. /// Homo sapiens protein	protein phosphatase 2 (formerly 2A), regulatory subunit B, beta isoform	0.000647202	1.272802742

	phosphatase 2 (formerly 2A), regulatory subunit B, beta isoform (PPP2R2B), transcript variant 4, mRNA. /// Homo sapiens protein phosphatase 2 (formerly 2A), regulatory subunit B, beta isoform (PPP2R2B), transcript variant 5, mRNA.			
WDR23	Isoform 1 of WD repeat-containing protein 23 gene:ENSG00000100897 /// Isoform 3 of WD repeat-containing protein 23 gene:ENSG00000100897 /// Isoform 3 of WD repeat-containing protein 23 gene:ENSG00000100897 /// Isoform 2 of WD repeat-containing protein 23 gene:ENSG00000100897 /// Homo sapiens GL014 mRNA, complete cds. /// Homo sapiens WD repeat domain 23 (WDR23), transcript variant 2, mRNA. /// Homo sapiens WD repeat domain 23 (WDR23), transcript variant 1, mRNA.	WD repeat domain 23	0.007992413	1.194928291

WIPI1	Isoform 1 of WD repeat domain phosphoinositide-interacting protein 1 gene:ENSG00000070540 /// Homo sapiens WD repeat domain, phosphoinositide interacting 1, mRNA (cDNA clone MGC:49021 IMAGE:6055425), complete cds. /// Homo sapiens WD repeat domain, phosphoinositide interacting 1 (WIPI1), mRNA.	WD repeat domain, phosphoinositide interacting 1	0.008353017	1.185284565
NEDD1	Protein NEDD1 gene:ENSG00000139350 /// Homo sapiens cDNA, FLJ79470 complete cds, highly similar to Protein NEDD1. /// Homo sapiens neural precursor cell expressed, developmentally down-regulated 1 (NEDD1), transcript variant 1, mRNA. /// Homo sapiens neural precursor cell expressed, developmentally down-regulated 1 (NEDD1), transcript variant 2, mRNA. /// Homo sapiens neural precursor cell expressed, developmentally down-regulated 1 (NEDD1), transcript variant 3, mRNA. /// Homo sapiens neural precursor cell expressed, developmentally down-regulated 1 (NEDD1), transcript variant 4, mRNA.	neural precursor cell expressed, developmentally down-regulated 1	0.003434285	1.195487635
**Functional Group 47**	**Transcript Description**	**Gene Title**	**Bayes.lnp**	**Fold**
TNFAIP1	BTB/POZ domain-containing protein TNFAIP1 gene:ENSG00000109079 /// Human B12 protein mRNA, complete cds. /// Homo sapiens tumor necrosis factor, alpha-induced protein 1 (endothelial) (TNFAIP1), mRNA.	tumor necrosis factor, alpha-induced protein 1 (endothelial)	0.000362902	-1.234772617

TRPC4	transient receptor potential cation channel, subfamily C, member 4 isoform epsilon gene:ENSG00000133107 /// Transient receptor potential cation channel, subfamily C, member 4, isoform CRA_b gene:ENSG00000133107 /// transient receptor potential cation channel, subfamily C, member 4 isoform zeta gene:ENSG00000133107 /// Isoform Alpha of Short transient receptor potential channel 4 gene:ENSG00000133107 /// transient receptor potential cation channel, subfamily C, member 4 isoform zeta gene:ENSG00000133107 /// Isoform Delta of Short transient receptor potential channel 4 gene:ENSG00000133107 /// Isoform Gamma of Short transient receptor potential channel 4 gene:ENSG00000133107 /// Isoform Beta of Short transient receptor potential channel 4 gene:ENSG00000133107 /// Homo sapiens mRNA; cDNA DKFZp686M09131 (from clone DKFZp686M09131). /// Homo sapiens transient receptor potential cation channel, subfamily C, member 4 (TRPC4), transcript variant alpha, mRNA. /// Homo sapiens transient receptor potential cation channel, subfamily C, member 4 (TRPC4), transcript variant epsilon, mRNA. /// Homo sapiens transient receptor potential cation channel, subfamily C, member 4 (TRPC4), transcript variant beta, mRNA. /// Homo sapiens transient receptor potential cation channel, subfamily C, member 4 (TRPC4), transcript variant gamma, mRNA. /// Homo sapiens transient receptor potential cation channel, subfamily C, member 4 (TRPC4), transcript variant delta, mRNA. /// Homo sapiens transient receptor potential cation channel, subfamily C,	transient receptor potential cation channel, subfamily C, member 4	0.00880903	-1.183446434

	member 4 (TRPC4), transcript variant zeta, mRNA.			

CACNA1B	Isoform Alpha-1B-1 of Voltage-dependent N-type calcium channel subunit alpha-1B gene:ENSG00000148408 /// Calcium channel, voltage-dependent, N type, alpha 1B subunit gene:ENSG00000148408 /// Calcium channel, voltage-dependent, N type, alpha 1B subunit gene:ENSG00000148408 /// Calcium channel, voltage-dependent, N type, alpha 1B subunit gene:ENSG00000148408 /// Isoform Alpha-1B-1 of Voltage-dependent N-type calcium channel subunit alpha-1B gene:ENSG00000148408 /// Calcium channel, voltage-dependent, N type, alpha 1B subunit gene:ENSG00000148408 /// Calcium channel alpha12.2 subunit (Fragment) gene:ENSG00000148408 /// Calcium channel, voltage-dependent, N type, alpha 1B subunit gene:ENSG00000148408 /// Human N-type calcium channel alpha-1 subunit mRNA, complete cds. /// Homo sapiens calcium channel, voltage-dependent, N type, alpha 1B subunit (CACNA1B), mRNA.	calcium channel, voltage-dependent, N type, alpha 1B subunit	0.008512488	-1.218059768
CNGB1	cyclic nucleotide gated channel beta 1 isoform a gene:ENSG00000070729 /// cyclic nucleotide gated channel beta 1 isoform b gene:ENSG00000070729 /// Human cGMP-gated cation channel beta subunit (CNCG2) mRNA, complete cds. /// Homo sapiens cyclic nucleotide gated channel beta 1 (CNGB1), transcript variant 1, mRNA. /// Homo sapiens cyclic nucleotide gated channel beta 1 (CNGB1), transcript variant 2, mRNA.	cyclic nucleotide gated channel beta 1	0.008471151	-1.237879459

KCNH8	Potassium voltage-gated channel subfamily H member 8 gene:ENSG00000183960 /// Homo sapiens mRNA for potassium voltage-gated channel, subfamily H, member 8 variant protein. /// Homo sapiens potassium voltage-gated channel, subfamily H (eag-related), member 8 (KCNH8), mRNA.	potassium voltage-gated channel, subfamily H (eag-related), member 8	0.00168409	-1.34383154
CACNA2D1	Voltage-dependent calcium channel subunit alpha-2/delta-1 gene:ENSG00000153956 /// Dihydropyridine receptor alpha 2 subunit gene:ENSG00000153956 /// Neuronal voltage-dependent calcium channel alpha 2A subunit gene:ENSG00000153956 /// Homo sapiens calcium channel, voltage-dependent, alpha 2/delta subunit 1, mRNA (cDNA clone MGC:151077 IMAGE:40126019), complete cds. /// Homo sapiens calcium channel, voltage-dependent, alpha 2/delta subunit 1 (CACNA2D1), mRNA.	calcium channel, voltage-dependent, alpha 2/delta subunit 1	0.003043243	-1.170202839
CHRNA9	Neuronal acetylcholine receptor subunit alpha-9 gene:ENSG00000174343 /// Homo sapiens cholinergic receptor, nicotinic, alpha 9, mRNA (cDNA clone MGC:142109 IMAGE:8322601), complete cds. /// Homo sapiens cholinergic receptor, nicotinic, alpha 9 (CHRNA9), mRNA.	cholinergic receptor, nicotinic, alpha 9	0.008365153	-1.272045213
GABRE	Gamma-aminobutyric acid receptor subunit epsilon gene:ENSG00000102287 /// Putative uncharacterized protein GABRE gene:ENSG00000102287 /// Putative uncharacterized protein GABRE gene:ENSG00000102287 /// 42 kDa protein gene:ENSG00000102287 /// Homo sapiens gamma-aminobutyric acid (GABA) A receptor, epsilon, mRNA (cDNA clone MGC:26504 IMAGE:4798754), complete cds. /// Homo sapiens gamma-aminobutyric	gamma-aminobutyric acid (GABA) A receptor, epsilon	4.29924E-10	-2.02462221

	acid (GABA) A receptor, epsilon (GABRE), mRNA.			
PLLP	Plasmolipin gene:ENSG00000102934 /// Homo sapiens plasma membrane proteolipid (plasmolipin), mRNA (cDNA clone MGC:3357 IMAGE:3633345), complete cds. /// Homo sapiens plasma membrane proteolipid (plasmolipin) (PLLP), mRNA.	plasma membrane proteolipid (plasmolipin)	0.009037296	-1.20315359
CD83	CD83 antigen gene:ENSG00000112149 /// Homo sapiens CD83 molecule, mRNA (cDNA clone MGC:26322 IMAGE:4818856), complete cds. /// Homo sapiens CD83 molecule (CD83), transcript variant 1, mRNA. /// Homo sapiens CD83 molecule (CD83), transcript variant 2, mRNA.	CD83 molecule	0.009333693	-1.200761833

KCNRG	Isoform Alpha of Tripartite motif-containing protein 13 gene:ENSG00000204977 /// ret finger protein 2 isoform 2 gene:ENSG00000204977 /// Isoform Alpha of Tripartite motif-containing protein 13 gene:ENSG00000204977 /// Isoform Alpha of Tripartite motif-containing protein 13 gene:ENSG00000204977 /// Isoform 2 of Putative potassium channel regulatory protein gene:ENSG00000198553 /// Isoform 1 of Putative potassium channel regulatory protein gene:ENSG00000198553 /// Homo sapiens putative potassium channel proteins regulator protein variant A (KCNRG) mRNA, complete cds; alternatively spliced. /// Homo sapiens leu5 (RFP2) mRNA, complete cds. /// Homo sapiens potassium channel regulator (KCNRG), transcript variant 1, mRNA. /// Homo sapiens tripartite motif-containing 13 (TRIM13), transcript variant 4, mRNA. /// Homo sapiens potassium channel regulator (KCNRG), transcript variant 2, mRNA. /// Homo sapiens tripartite motif-containing 13 (TRIM13), transcript variant 1, mRNA. /// Homo sapiens tripartite motif-containing 13 (TRIM13), transcript variant 2, mRNA. /// Homo sapiens tripartite motif-containing 13 (TRIM13), transcript variant 3, mRNA.	potassium channel regulator /// tripartite motif-containing 13	0.008562401	1.232643517

SLC4A11	cDNA FLJ56098, highly similar to Sodium bicarbonate transporter-like protein11 gene:ENSG00000088836 /// Isoform 1 of Sodium bicarbonate transporter-like protein 11 gene:ENSG00000088836 /// Homo sapiens solute carrier family 4, sodium borate transporter, member 11, mRNA (cDNA clone IMAGE:40035523), complete cds. /// Homo sapiens solute carrier family 4, sodium borate transporter, member 11 (SLC4A11), mRNA.	solute carrier family 4, sodium borate transporter, member 11	0.001164745	-1.202804336
KCNJ2	Inward rectifier potassium channel 2 gene:ENSG00000123700 /// Homo sapiens inwardly-rectifying potassium channel Kir2.1 (KCNJ2) mRNA, complete cds. /// Homo sapiens potassium inwardly-rectifying channel, subfamily J, member 2 (KCNJ2), mRNA.	potassium inwardly-rectifying channel, subfamily J, member 2	0.000274911	-1.367730218
KCNJ16	Inward rectifier potassium channel 16 gene:ENSG00000153822 /// Inward rectifier potassium channel 16 gene:ENSG00000153822 /// Inward rectifier potassium channel 16 gene:ENSG00000153822 /// Homo sapiens potassium inwardly-rectifying channel, subfamily J, member 16, mRNA (cDNA clone MGC:33717 IMAGE:5262150), complete cds. /// Homo sapiens potassium inwardly-rectifying channel, subfamily J, member 16 (KCNJ16), transcript variant 2, mRNA. /// Homo sapiens potassium inwardly-rectifying channel, subfamily J, member 16 (KCNJ16), transcript variant 3, mRNA. /// Homo sapiens potassium inwardly-rectifying channel, subfamily J, member 16 (KCNJ16), transcript variant 1, mRNA.	potassium inwardly-rectifying channel, subfamily J, member 16	0.00941639	1.352781385

CLCA2	Calcium-activated chloride channel regulator 2 gene:ENSG00000137975 /// Homo sapiens CLCA family member 2, chloride channel regulator, mRNA (cDNA clone MGC:46036 IMAGE:5752653), complete cds. /// Homo sapiens CLCA family member 2, chloride channel regulator (CLCA2), mRNA.	chloride channel regulator 2	0.001357236	-1.486668361
FXYD3	Isoform 2 of FXYD domain-containing ion transport regulator 3 gene:ENSG00000089356 /// Isoform 1 of FXYD domain-containing ion transport regulator 3 gene:ENSG00000089356 /// Isoform 2 of FXYD domain-containing ion transport regulator 3 gene:ENSG00000089356 /// Isoform 1 of FXYD domain-containing ion transport regulator 3 gene:ENSG00000089356 /// Human 11kd protein mRNA, complete cds. /// Homo sapiens FXYD domain containing ion transport regulator 3 (FXYD3), transcript variant 1, mRNA. /// Homo sapiens FXYD domain containing ion transport regulator 3 (FXYD3), transcript variant 2, mRNA. /// Homo sapiens FXYD domain containing ion transport regulator 3 (FXYD3), transcript variant 3, mRNA. /// Homo sapiens FXYD domain containing ion transport regulator 3 (FXYD3), transcript variant 4, mRNA. /// Homo sapiens FXYD domain containing ion transport regulator 3 (FXYD3), transcript variant 5, mRNA. /// Homo sapiens FXYD domain containing ion transport regulator 3 (FXYD3), transcript variant 6, mRNA. /// Homo sapiens FXYD domain containing ion transport regulator 3 (FXYD3), transcript variant 7, mRNA. /// Homo sapiens FXYD domain containing ion transport regulator 3 (FXYD3), transcript variant 8, mRNA.	FXYD domain containing ion transport regulator 3	2.12739E-05	-1.394300248

VDAC3	Isoform 1 of Voltage-dependent anion-selective channel protein 3 gene:ENSG00000078668 /// Homo sapiens voltage-dependent anion channel 3, mRNA (cDNA clone MGC:64870 IMAGE:6163890), complete cds. /// Homo sapiens voltage-dependent anion channel 3 (VDAC3), transcript variant 2, mRNA. /// Homo sapiens voltage-dependent anion channel 3 (VDAC3), transcript variant 1, mRNA.	voltage-dependent anion channel 3	0.003217151	1.182094071
PODXL	Podocalyxin-like protein 1 gene:ENSG00000128567 /// podocalyxin-like isoform 1 precursor gene:ENSG00000128567 /// Homo sapiens podocalyxin-like protein mRNA, complete cds. /// Homo sapiens podocalyxin-like (PODXL), transcript variant 1, mRNA. /// Homo sapiens podocalyxin-like (PODXL), transcript variant 2, mRNA.	podocalyxin-like	0.001142461	1.219198904
HEPH	hephaestin isoform b gene:ENSG00000089472 /// hephaestin isoform a gene:ENSG00000089472 /// hephaestin isoform b gene:ENSG00000089472 /// hephaestin isoform a gene:ENSG00000089472 /// Homo sapiens clone DNA104875 HEPH (UNQ2562) mRNA, complete cds. /// Homo sapiens hephaestin (HEPH), transcript variant 3, mRNA. /// Homo sapiens hephaestin (HEPH), transcript variant 1, mRNA. /// Homo sapiens hephaestin (HEPH), transcript variant 2, mRNA.	hephaestin	0.003484698	-1.159984964

SLC11A2	Isoform 2 of Natural resistance-associated macrophage protein 2 gene:ENSG00000110911 /// Isoform 1 of Natural resistance-associated macrophage protein 2 gene:ENSG00000110911 /// Divalent metal transporter gene:ENSG00000110911 /// Homo sapiens cDNA FLJ46799 fis, clone TRACH3031678, highly similar to Natural resistance-associated macrophage protein 2. /// Homo sapiens solute carrier family 11 (proton-coupled divalent metal ion transporters), member 2 (SLC11A2), mRNA.	solute carrier family 11 (proton-coupled divalent metal ion transporters), member 2	0.001989436	1.200926252
SLC9A1	Solute carrier family 9 (Sodium/hydrogen exchanger), member 1 gene:ENSG00000090020 /// Isoform 1 of Sodium/hydrogen exchanger 1 gene:ENSG00000090020 /// Isoform 2 of Sodium/hydrogen exchanger 1 gene:ENSG00000090020 /// Human Na/H antiporter (APNH1) mRNA, complete cds. /// Homo sapiens solute carrier family 9 (sodium/hydrogen exchanger), member 1 (SLC9A1), mRNA.	solute carrier family 9 (sodium/hydrogen exchanger), member 1	0.001554104	-1.242202257
SLC24A6	Isoform 1 of Sodium/potassium/calcium exchanger 6 gene:ENSG00000089060 /// Isoform 2 of Sodium/potassium/calcium exchanger 6 gene:ENSG00000089060 /// Homo sapiens solute carrier family 24 (sodium/potassium/calcium exchanger), member 6, mRNA (cDNA clone MGC:120605 IMAGE:40026360), complete cds. /// Homo sapiens solute carrier family 24 (sodium/potassium/calcium exchanger), member 6 (SLC24A6), mRNA.	solute carrier family 24 (sodium/potassium/calcium exchanger), member 6	0.008297168	-1.172206281
**Functional Group 48**	**Transcript Description**	**Gene Title**	**Bayes.lnp**	**Fold**

SLC1A1	Excitatory amino acid transporter 3 gene:ENSG00000106688 /// Solute carrier family 1 (Neuronal/epithelial high affinity glutamate transporter, system Xag), member 1 gene:ENSG00000106688 /// Homo sapiens solute carrier family 1 (neuronal/epithelial high affinity glutamate transporter, system Xag), member 1, mRNA (cDNA clone MGC:33786 IMAGE:5261168), complete cds. /// Homo sapiens solute carrier family 1 (neuronal/epithelial high affinity glutamate transporter, system Xag), member 1 (SLC1A1), mRNA.	solute carrier family 1 (neuronal/epithelial high affinity glutamate transporter, system Xag), member 1	0.000392958	1.343599293
SLC25A12	cDNA FLJ33752 fis, clone BRCAN2000364, highly similar to Calcium-binding mitochondrial carrier protein Aralar1 gene:ENSG00000115840 /// Calcium-binding mitochondrial carrier protein Aralar1 gene:ENSG00000115840 /// Homo sapiens mRNA for mitochondrial aspartate-glutamate carrier protein (SLC25A12 gene). /// Homo sapiens solute carrier family 25 (mitochondrial carrier, Aralar), member 12 (SLC25A12), nuclear gene encoding mitochondrial protein, mRNA.	solute carrier family 25 (mitochondrial carrier, Aralar), member 12	0.007177625	1.196424029

SLC7A8	Integral membrane protein E16H gene:ENSG00000092068 /// Putative uncharacterized protein (Fragment) gene:ENSG00000092068 /// solute carrier family 7 (cationic amino acid transporter, y+ system), member 8 isoform b gene:ENSG00000092068 /// Large neutral amino acids transporter small subunit 2 gene:ENSG00000092068 /// TA1 gene:ENSG00000092068 /// Homo sapiens hLAT2 mRNA for L-type amino acid transporter 2, complete cds. /// Homo sapiens solute carrier family 7 (cationic amino acid transporter, y+ system), member 8 (SLC7A8), transcript variant 1, mRNA. /// Homo sapiens solute carrier family 7 (cationic amino acid transporter, y+ system), member 8 (SLC7A8), transcript variant 2, mRNA.	solute carrier family 7 (cationic amino acid transporter, y+ system), member 8	5.26602E-05	-1.276203738
SLC7A14	Homo sapiens solute carrier family 7 (cationic amino acid transporter, y+ system), member 14 (SLC7A14), mRNA.	solute carrier family 7 (cationic amino acid transporter, y+ system), member 14	0.724825055	-1.037307393
SLC25A15	Mitochondrial ornithine transporter 1 gene:ENSG00000102743 /// Solute carrier family 25 (Mitochondrial carrier gene:ENSG00000102743 /// Homo sapiens ornithine transporter (ORNT1) mRNA, complete cds; nuclear gene for mitochondrial product. /// Homo sapiens solute carrier family 25 (mitochondrial carrier; ornithine transporter) member 15 (SLC25A15), nuclear gene encoding mitochondrial protein, mRNA.	solute carrier family 25 (mitochondrial carrier; ornithine transporter) member 15	0.00683359	1.178797763
**Functional Group 49**	**Transcript Description**	**Gene Title**	**Bayes.lnp**	**Fold**

BCS1L	Mitochondrial chaperone BCS1 gene:ENSG00000074582 /// Mitochondrial chaperone BCS1 gene:ENSG00000074582 /// Mitochondrial chaperone BCS1 gene:ENSG00000074582 /// Mitochondrial chaperone BCS1 gene:ENSG00000074582 /// Homo sapiens cDNA FLJ38891 fis, clone NHNPC2000062, highly similar to Mitochondrial chaperone BCS1. /// Homo sapiens BCS1-like (yeast) (BCS1L), transcript variant 1, mRNA. /// Homo sapiens BCS1-like (yeast) (BCS1L), nuclear gene encoding mitochondrial protein, transcript variant 2, mRNA.	BCS1-like (yeast)	0.006166574	1.209016657
ABCB1	Multidrug resistance protein 1 gene:ENSG00000085563 /// P-glycoprotein 1 (Fragment) gene:ENSG00000085563 /// Homo sapiens P-glycoprotein (PGY1) mRNA, complete cds. /// Homo sapiens ATP-binding cassette, sub-family B (MDR/TAP), member 1 (ABCB1), mRNA.	ATP-binding cassette, sub-family B (MDR/TAP), member 1	0.00068705	-1.506968833
ABCA12	Isoform 1 of ATP-binding cassette sub-family A member 12 gene:ENSG00000144452 /// Isoform 2 of ATP-binding cassette sub-family A member 12 gene:ENSG00000144452 /// Homo sapiens ABCA12 transporter subfamily A mRNA, complete cds. /// Homo sapiens ATP-binding cassette, sub-family A (ABC1), member 12 (ABCA12), transcript variant 2, mRNA. /// Homo sapiens ATP-binding cassette, sub-family A (ABC1), member 12 (ABCA12), transcript variant 1, mRNA.	ATP-binding cassette, sub-family A (ABC1), member 12	1.23056E-05	-1.767430322

KATNAL2	40 kDa protein gene:ENSG00000167216 /// Isoform 2 of Katanin p60 ATPase-containing subunit A-like 2 gene:ENSG00000167216 /// Homo sapiens katanin p60 subunit A-like 2, mRNA (cDNA clone MGC:33211 IMAGE:4829800), complete cds. /// Homo sapiens katanin p60 subunit A-like 2 (KATNAL2), mRNA.	katanin p60 subunit A-like 2	0.006896265	-1.186175082
**Functional Group 50**	**Transcript Description**	**Gene Title**	**Bayes.lnp**	**Fold**
DNAJC15	DnaJ homolog subfamily C member 15 gene:ENSG00000120675 /// Homo sapiens DnaJ (Hsp40) homolog, subfamily C, member 15, mRNA (cDNA clone MGC:110875 IMAGE:30530999), complete cds. /// Homo sapiens DnaJ (Hsp40) homolog, subfamily C, member 15 (DNAJC15), mRNA.	DnaJ (Hsp40) homolog, subfamily C, member 15	0.002111086	1.248706673
TXNDC15	Isoform 1 of Thioredoxin domain-containing protein 15 gene:ENSG00000113621 /// Homo sapiens mRNA; cDNA DKFZp686L0872 (from clone DKFZp686L0872). /// Homo sapiens thioredoxin domain containing 15 (TXNDC15), mRNA.	thioredoxin domain containing 15	0.009044932	1.164926261
REEP5	Receptor expression-enhancing protein 5 gene:ENSG00000129625 /// 21 kDa protein gene:ENSG00000129625 /// Homo sapiens receptor accessory protein 5, mRNA (cDNA clone MGC:2267 IMAGE:3350749), complete cds. /// Homo sapiens receptor accessory protein 5 (REEP5), mRNA.	receptor accessory protein 5	0.002075343	1.179950903
SLC7A14	Homo sapiens solute carrier family 7 (cationic amino acid transporter, y+ system), member 14 (SLC7A14), mRNA.	solute carrier family 7 (cationic amino acid transporter, y+ system), member 14	0.724825055	-1.037307393

DCBLD2	Isoform 1 of Discoidin, CUB and LCCL domain-containing protein 2 gene:ENSG00000057019 /// Isoform 2 of Discoidin, CUB and LCCL domain-containing protein 2 gene:ENSG00000057019 /// 42 kDa protein gene:ENSG00000057019 /// Homo sapiens mRNA for CLCP1, complete cds. /// Homo sapiens discoidin, CUB and LCCL domain containing 2 (DCBLD2), mRNA.	discoidin, CUB and LCCL domain containing 2	0.004394744	-1.154840632
TNFRSF9	Tumor necrosis factor receptor superfamily member 9 gene:ENSG00000049249 /// Human activation dependent T cell mRNA, complete cds. /// Homo sapiens tumor necrosis factor receptor superfamily, member 9 (TNFRSF9), mRNA.	tumor necrosis factor receptor superfamily, member 9	3.28208E-06	-1.98832707
GPR172A	Porcine endogenous retrovirus A receptor 1 gene:ENSG00000185803 /// Porcine endogenous retrovirus A receptor 1 gene:ENSG00000185803 /// Homo sapiens cDNA FLJ11856 fis, clone HEMBA1006789. /// Homo sapiens G protein-coupled receptor 172A (GPR172A), mRNA.	G protein-coupled receptor 172A	0.002766543	1.174820046
PITPNM2	Isoform 1 of Membrane-associated phosphatidylinositol transfer protein 2 gene:ENSG00000090975 /// Isoform 2 of Membrane-associated phosphatidylinositol transfer protein 2 gene:ENSG00000090975 /// Isoform 3 of Membrane-associated phosphatidylinositol transfer protein 2 gene:ENSG00000090975 /// Homo sapiens mRNA for KIAA1457 protein, partial cds. /// Homo sapiens phosphatidylinositol transfer protein, membrane-associated 2 (PITPNM2), mRNA.	phosphatidylinositol transfer protein, membrane-associated 2	0.005935749	-1.244589029

TMEM30A	Isoform 1 of Cell cycle control protein 50A gene:ENSG00000112697 /// Isoform 2 of Cell cycle control protein 50A gene:ENSG00000112697 /// Homo sapiens cDNA FLJ53706 complete cds, highly similar to Cell cycle control protein 50A. /// Homo sapiens transmembrane protein 30A (TMEM30A), mRNA.	transmembrane protein 30A	0.0021512	-1.16088408
FRMD5	Putative uncharacterized protein FRMD5 gene:ENSG00000171877 /// Isoform 1 of FERM domain-containing protein 5 gene:ENSG00000171877 /// Homo sapiens cDNA FLJ33253 fis, clone ASTRO2005343, weakly similar to BAND 4.1-LIKE PROTEIN 4. /// Homo sapiens FERM domain containing 5 (FRMD5), transcript variant 2, mRNA.	FERM domain containing 5	0.0008612	-1.256314411
LETMD1	cDNA FLJ45633 fis, clone CHONS2002419, highly similar to Homo sapiens LETM1 domain containing 1 (LETMD1), transcript variant 1, mRNA gene:ENSG00000050426 /// Isoform 3 of LETM1 domain-containing protein 1 gene:ENSG00000050426 /// Isoform 6 of LETM1 domain-containing protein 1 gene:ENSG00000050426 /// Homo sapiens cervical cancer 1 protooncogene protein p40 mRNA, complete cds. /// Homo sapiens LETM1 domain containing 1 (LETMD1), transcript variant 1, mRNA. /// Homo sapiens LETM1 domain containing 1 (LETMD1), transcript variant 2, mRNA.	LETM1 domain containing 1	0.0017737	1.263994996

PODXL	Podocalyxin-like protein 1 gene:ENSG00000128567 /// podocalyxin-like isoform 1 precursor gene:ENSG00000128567 /// Homo sapiens podocalyxin-like protein mRNA, complete cds. /// Homo sapiens podocalyxin-like (PODXL), transcript variant 1, mRNA. /// Homo sapiens podocalyxin-like (PODXL), transcript variant 2, mRNA.	podocalyxin-like	0.001142461	1.219198904
SCAMP2	Secretory carrier-associated membrane protein 2 gene:ENSG00000140497 /// Homo sapiens cDNA FLJ39475 fis, clone PROST2013121, highly similar to SECRETORY CARRIER-ASSOCIATED MEMBRANE PROTEIN 2. /// Homo sapiens secretory carrier membrane protein 2 (SCAMP2), mRNA.	secretory carrier membrane protein 2	0.001183903	1.201401381
CNIH	Putative uncharacterized protein CNIH gene:ENSG00000100528 /// Protein cornichon homolog gene:ENSG00000100528 /// Homo sapiens cornichon mRNA, complete cds. /// Homo sapiens cornichon homolog (Drosophila) (CNIH), mRNA.	cornichon homolog (Drosophila)	0.004930261	1.175375923
CNIH3	Protein cornichon homolog 3 gene:ENSG00000143786 /// Homo sapiens cornichon homolog 3 (Drosophila), mRNA (cDNA clone MGC:9297 IMAGE:3894832), complete cds. /// Homo sapiens cornichon homolog 3 (Drosophila) (CNIH3), mRNA.	cornichon homolog 3 (Drosophila)	0.003939682	-1.24164788
SLC46A1	Isoform 1 of Proton-coupled folate transporter gene:ENSG00000076351 /// Isoform 2 of Proton-coupled folate transporter gene:ENSG00000076351 /// Homo sapiens solute carrier family 46 (folate transporter), member 1, mRNA (cDNA clone MGC:9564 IMAGE:3872267), complete cds. /// Homo sapiens solute carrier family 46 (folate transporter), member 1 (SLC46A1), mRNA.	solute carrier family 46 (folate transporter), member 1	0.000986004	-1.222343379

MFSD3	Major facilitator superfamily domain-containing protein 3 gene:ENSG00000167700 /// Homo sapiens major facilitator superfamily domain containing 3, mRNA (cDNA clone MGC:9810 IMAGE:3860434), complete cds. /// Homo sapiens major facilitator superfamily domain containing 3 (MFSD3), mRNA.	major facilitator superfamily domain containing 3	1.64025E-05	1.337808249
LRRTM4	cdna:known chromosome:NCBI36:2:76828353:77602973:-1 gene:ENSG00000176204 /// cdna:known chromosome:NCBI36:2:76829187:77602907:-1 gene:ENSG00000176204 /// cdna:known chromosome:NCBI36:2:76829192:77603002:-1 gene:ENSG00000176204 /// Isoform 1 of Leucine-rich repeat transmembrane neuronal protein 4 gene:ENSG00000176204 /// cdna:known chromosome:NCBI36:2:77597302:77603010:-1 gene:ENSG00000176204 /// cdna:known chromosome:NCBI36:2:77598150:77602844:-1 gene:ENSG00000176204 /// Homo sapiens clone DNA142392 GFHL3075 (UNQ3075) mRNA, complete cds. /// Homo sapiens leucine rich repeat transmembrane neuronal 4 (LRRTM4), transcript variant 1, mRNA. /// Homo sapiens leucine rich repeat transmembrane neuronal 4 (LRRTM4), transcript variant 2, mRNA.	leucine rich repeat transmembrane neuronal 4	0.00327488	-1.221016835
LAYN	Isoform 2 of Layilin gene:ENSG00000204381 /// Isoform 1 of Layilin gene:ENSG00000204381 /// Homo sapiens cDNA FLJ30977 fis, clone HHDPC2000095, highly similar to Cricetulus griseus layilin mRNA. /// Homo sapiens layilin (LAYN), mRNA.	layilin	0.00662727	-1.230555767

IMPAD1	Homo sapiens inositol monophosphatase domain containing 1 (IMPAD1), mRNA.	inositol monophosphatase domain containing 1	0.314382425	1.135431175
GRAMD1B	cDNA FLJ45549 fis, clone BRTHA2036295 gene:ENSG00000023171 /// Homo sapiens cDNA FLJ45549 fis, clone BRTHA2036295. /// Homo sapiens GRAM domain containing 1B (GRAMD1B), mRNA.	GRAM domain containing 1B	0.008827007	1.234069336
ASAM	Adipocyte adhesion molecule gene:ENSG00000166250 /// Uncharacterized protein ENSP00000376525 (Fragment) gene:ENSG00000166250 /// Homo sapiens adipocyte adhesion molecule mRNA, complete cds. /// Homo sapiens adipocyte-specific adhesion molecule (ASAM), mRNA.	adipocyte-specific adhesion molecule	0.004035831	1.172514888
TOR1AIP2	Torsin-1A-interacting protein 2 gene:ENSG00000169905 /// IFRG15 protein gene:ENSG00000218839 /// Homo sapiens cDNA FLJ77012 complete cds, highly similar to Homo sapiens interferon responsive gene 15 (IFRG15), mRNA. /// Homo sapiens torsin A interacting protein 2, mRNA (cDNA clone MGC:126581 IMAGE:8069038), complete cds. /// Homo sapiens torsin A interacting protein 2 (TOR1AIP2), mRNA.	interferon responsive gene 15 /// torsin A interacting protein 2	0.001893375	-1.192769275
IER3IP1	Homo sapiens PRO2309 mRNA, complete cds.	immediate early response 3 interacting protein 1	0.416335366	-1.125829568
TCTN2	Tectonic-2 gene:ENSG00000168778 /// Homo sapiens TECT2 (TECT2) mRNA, complete cds. /// Homo sapiens tectonic family member 2 (TCTN2), mRNA.	tectonic family member 2	0.003700585	1.224374462

EMP3	Epithelial membrane protein 3 gene:ENSG00000142227 /// Homo sapiens epithelial membrane protein 3, mRNA (cDNA clone MGC:9511 IMAGE:3891050), complete cds. /// Homo sapiens epithelial membrane protein 3 (EMP3), mRNA.	epithelial membrane protein 3	0.006650587	1.18997891
CD99	Isoform I of CD99 antigen gene:ENSG00000002586 /// Putative uncharacterized protein CD99 gene:ENSG00000002586 /// CD99 antigen isoform b precursor gene:ENSG00000002586 /// Putative uncharacterized protein CD99 gene:ENSG00000002586 /// Putative uncharacterized protein CD99 gene:ENSG00000002586 /// Homo sapiens CD99 molecule, mRNA (cDNA clone MGC:19734 IMAGE:3606974), complete cds. /// Homo sapiens CD99 molecule (CD99), transcript variant 1, mRNA. /// Homo sapiens CD99 molecule (CD99), transcript variant 2, mRNA.	CD99 molecule	0.004074288	1.190899882
PPAP2C	phosphatidic acid phosphatase type 2C isoform 3 gene:ENSG00000141934 /// Lipid phosphate phosphohydrolase 2 gene:ENSG00000141934 /// Homo sapiens phosphatidic acid phosphatase type 2C, mRNA (cDNA clone MGC:3813 IMAGE:3659728), complete cds. /// Homo sapiens phosphatidic acid phosphatase type 2C (PPAP2C), transcript variant 2, mRNA. /// Homo sapiens phosphatidic acid phosphatase type 2C (PPAP2C), transcript variant 1, mRNA. /// Homo sapiens phosphatidic acid phosphatase type 2C (PPAP2C), transcript variant 3, mRNA.	phosphatidic acid phosphatase type 2C	0.001639994	-1.198286466

MPZL1	Isoform 3 of Myelin protein zero-like protein 1 gene:ENSG00000197965 /// Isoform 1 of Myelin protein zero-like protein 1 gene:ENSG00000197965 /// Myelin protein zero-like 1 gene:ENSG00000197965 /// Isoform 3 of Myelin protein zero-like protein 1 gene:ENSG00000197965 /// Homo sapiens clone DNA76510 MPZL1 (UNQ849) mRNA, complete cds. /// Homo sapiens myelin protein zero-like 1 (MPZL1), transcript variant 1, mRNA. /// Homo sapiens myelin protein zero-like 1 (MPZL1), transcript variant 2, mRNA.	myelin protein zero-like 1	0.003136446	1.168400169
GLIPR1	Glioma pathogenesis-related protein 1 gene:ENSG00000139278 /// Homo sapiens GLI pathogenesis-related 1, mRNA (cDNA clone MGC:21203 IMAGE:4453841), complete cds. /// Homo sapiens GLI pathogenesis-related 1 (GLIPR1), mRNA.	GLI pathogenesis-related 1	0.001216405	-1.219278501
C1orf43	Isoform 2 of Uncharacterized protein C1orf43 gene:ENSG00000143612 /// Isoform 4 of Uncharacterized protein C1orf43 gene:ENSG00000143612 /// Isoform 1 of Uncharacterized protein C1orf43 gene:ENSG00000143612 /// Isoform 5 of Uncharacterized protein C1orf43 gene:ENSG00000143612 /// Isoform 3 of Uncharacterized protein C1orf43 gene:ENSG00000143612 /// 18 kDa protein gene:ENSG00000143612 /// Homo sapiens chromosome 1 open reading frame 43, mRNA (cDNA clone MGC:5332 IMAGE:2901006), complete cds. /// Homo sapiens chromosome 1 open reading frame 43 (C1orf43), transcript variant 3, mRNA. /// Homo sapiens chromosome 1 open reading frame 43 (C1orf43), transcript variant 2, mRNA. /// Homo sapiens chromosome 1 open reading frame 43 (C1orf43), transcript variant 1, mRNA.	chromosome 1 open reading frame 43	1.23337E-05	1.303548616

REEP3	Isoform 1 of Receptor expression-enhancing protein 3 gene:ENSG00000165476 /// Isoform 1 of Receptor expression-enhancing protein 3 gene:ENSG00000165476 /// Homo sapiens receptor accessory protein 3, mRNA (cDNA clone MGC:87479 IMAGE:30339865), complete cds. /// Homo sapiens receptor accessory protein 3 (REEP3), mRNA.	receptor accessory protein 3	0.001551293	1.229753274
SMCR7L	Putative uncharacterized protein DKFZp434F0116 gene:ENSG00000100335 /// Smith-Magenis syndrome chromosome region candidate gene 7 protein-like gene:ENSG00000100335 /// Putative uncharacterized protein DKFZp434F0116 gene:ENSG00000100335 /// Smith-Magenis syndrome chromosome region candidate gene 7 protein-like gene:ENSG00000100335 /// Homo sapiens Smith-Magenis syndrome chromosome region, candidate 7-like, mRNA (cDNA clone MGC:15774 IMAGE:3502711), complete cds. /// Homo sapiens Smith-Magenis syndrome chromosome region, candidate 7-like (SMCR7L), mRNA.	Smith-Magenis syndrome chromosome region, candidate 7-like	0.003381395	1.214924348
RNF170	Isoform 3 of RING finger protein 170 gene:ENSG00000120925 /// 37 kDa protein gene:ENSG00000120925 /// Isoform 4 of RING finger protein 170 gene:ENSG00000120925 /// Isoform 1 of RING finger protein 170 gene:ENSG00000120925 /// Homo sapiens cDNA FLJ33545 fis, clone BRAMY2008382. /// Homo sapiens ring finger protein 170 (RNF170), mRNA.	ring finger protein 170	0.003313338	1.229727891

LOC399491	cdna:known chromosome:NCBI36:16:14713047:14727651:1 gene:ENSG00000069651 /// Putative uncharacterized protein A-589H1.1 gene:ENSG00000069651 /// Nuclear pore complex-interacting protein gene:ENSG00000185208 /// Putative uncharacterized protein A-589H1.1 gene:ENSG00000185208 /// cdna:known chromosome:NCBI36:16:14924160:14953402:1 gene:ENSG00000183793 /// nuclear pore complex interacting protein gene:ENSG00000183426 /// Putative uncharacterized protein ENSP00000349390 (Fragment) gene:ENSG00000188599 /// Isoform 1 of GPS, PLAT and transmembrane domain-containing protein FLJ00285 gene:ENSG00000188599 /// cdna:known chromosome:NCBI36:16:15365326:15371268:-1 gene:ENSG00000196908 /// Uncharacterized protein ENSP00000345016 gene:ENSG00000214967 /// cDNA FLJ44848 fis, clone BRACE3051621, highly similar to Polycystin gene:ENSG00000183458 /// cDNA FLJ42525 fis, clone BRACE3001391, highly similar to Polycystin gene:ENSG00000183458 /// Uncharacterized protein ENSP00000345016 gene:ENSG00000205746 /// NPIP-like protein LOC440350 gene:ENSG00000196877 /// NPIP-like protein LOC440350 gene:ENSG00000198156 /// cdna:known chromosome:NCBI36:16:28957477:28970549:1 gene:ENSG00000196161 /// Homo sapiens mRNA for FLJ00285 protein. /// Homo sapiens mRNA for FLJ00322 protein. /// Homo sapiens nuclear pore complex interacting protein (NPIP), mRNA. /// Homo sapiens similar to nuclear pore	LOC399491 protein /// nuclear pore complex interacting protein /// similar to nuclear pore complex interacting protein	0.028871971	-1.112605265

	complex interacting protein (LOC440350), mRNA.			
ARV1	Protein ARV1 gene:ENSG00000173409 /// 26 kDa protein gene:ENSG00000173409 /// Homo sapiens ARV1 mRNA, complete cds. /// Homo sapiens ARV1 homolog (S. cerevisiae) (ARV1), mRNA.	ARV1 homolog (S. cerevisiae)	0.000498282	1.290297236
TMEM126A	Transmembrane protein 126A gene:ENSG00000171202 /// Homo sapiens transmembrane protein 126A, mRNA (cDNA clone MGC:14138 IMAGE:3948518), complete cds. /// Homo sapiens transmembrane protein 126A (TMEM126A), mRNA.	transmembrane protein 126A	0.003177148	1.274946674

NRM	Isoform 2 of Nurim gene:ENSG00000206391 /// Isoform 1 of Nurim gene:ENSG00000206391 /// Isoform 2 of Nurim gene:ENSG00000206484 /// Isoform 1 of Nurim gene:ENSG00000206484 /// Isoform 1 of Nurim gene:ENSG00000137404 /// Isoform 2 of Nurim gene:ENSG00000137404 /// Isoform 4 of Nurim gene:ENSG00000137404 /// Isoform 5 of Nurim gene:ENSG00000137404 /// Isoform 1 of Nurim gene:ENSG00000137404 /// Homo sapiens cDNA PSEC0207 fis, clone HEMBA1002981, highly similar to Homo sapiens nurim (nuclear envelope membrane protein) (NRM), mRNA. /// Homo sapiens nurim (nuclear envelope membrane protein) (NRM), mRNA.	nurim (nuclear envelope membrane protein)	0.019683966	1.16282963
C17orf61	UPF0451 protein C17orf61 gene:ENSG00000205544 /// Homo sapiens cDNA FLJ31260 fis, clone KIDNE2005854, highly similar to Phospholipid scramblase 3. /// Homo sapiens cDNA FLJ77856 complete cds, highly similar to Homo sapiens phospholipid scramblase 3 (PLSCR3), mRNA. /// Homo sapiens chromosome 17 open reading frame 61, mRNA (cDNA clone MGC:40107 IMAGE:5418259), complete cds.	phospholipid scramblase 3 /// chromosome 17 open reading frame 61	0.001099691	1.276122626
DKFZP564O0823	Protein PARM-1 gene:ENSG00000169116 /// Homo sapiens clone DNA92223 VYKT1879 (UNQ1879) mRNA, complete cds. /// Homo sapiens DKFZP564O0823 protein (DKFZP564O0823), mRNA.	DKFZP564O0823 protein	5.61324E-05	-1.348721612
CCDC56	Coiled-coil domain-containing protein 56 gene:ENSG00000183978 /// Homo sapiens mRNA for HSPC009 variant, clone: PNC06538. /// Homo sapiens coiled-coil domain containing 56 (CCDC56), mRNA.	coiled-coil domain containing 56	0.003281488	1.232223987

RPRM	Protein reprimo gene:ENSG00000177519 /// Homo sapiens reprimo, TP53 dependent G2 arrest mediator candidate, mRNA (cDNA clone MGC:11260 IMAGE:3942270), complete cds. /// Homo sapiens reprimo, TP53 dependent G2 arrest mediator candidate (RPRM), mRNA.	reprimo, TP53 dependent G2 arrest mediator candidate	0.005161311	1.194146899
SERAC1	Isoform 1 of Protein SERAC1 gene:ENSG00000122335 /// Isoform 2 of Protein SERAC1 gene:ENSG00000122335 /// Isoform 2 of Protein SERAC1 gene:ENSG00000122335 /// Homo sapiens serine active site containing 1, mRNA (cDNA clone MGC:3108 IMAGE:3350731), complete cds. /// Homo sapiens serine active site containing 1 (SERAC1), mRNA.	serine active site containing 1	0.008576676	1.170782982
CCDC136	Isoform 3 of Coiled-coil domain-containing protein 136 gene:ENSG00000128596 /// Isoform 1 of Coiled-coil domain-containing protein 136 gene:ENSG00000128596 /// Isoform 2 of Coiled-coil domain-containing protein 136 gene:ENSG00000128596 /// Putative uncharacterized protein CCDC136 gene:ENSG00000128596 /// Putative uncharacterized protein CCDC136 (Fragment) gene:ENSG00000128596 /// Homo sapiens coiled-coil domain containing 136, mRNA (cDNA clone MGC:167819 IMAGE:8860498), complete cds. /// Homo sapiens coiled-coil domain containing 136 (CCDC136), mRNA.	coiled-coil domain containing 136	0.001733181	-1.317000765
KRTCAP3	Isoform 1 of Keratinocytes-associated protein 3 gene:ENSG00000157992 /// Isoform 2 of Keratinocytes-associated protein 3 gene:ENSG00000157992 /// Homo sapiens keratinocytes associated protein 3 (KCP3) mRNA, complete cds. /// Homo sapiens keratinocyte associated protein 3 (KRTCAP3), mRNA.	keratinocyte associated protein 3	0.008535343	1.184052989

C7orf53	Isoform 1 of Coiled-coil domain-containing transmembrane protein C7orf53 gene:ENSG00000181016 /// Homo sapiens chromosome 7 open reading frame 53, mRNA (cDNA clone IMAGE:4830480). /// Homo sapiens chromosome 7 open reading frame 53 (C7orf53), transcript variant 1, mRNA. /// Homo sapiens chromosome 7 open reading frame 53 (C7orf53), transcript variant 2, mRNA.	chromosome 7 open reading frame 53	0.000271813	-1.576876768
SLC44A5	Isoform 2 of Choline transporter-like protein 5 gene:ENSG00000137968 /// Isoform 2 of Choline transporter-like protein 5 gene:ENSG00000137968 /// Isoform 1 of Choline transporter-like protein 5 gene:ENSG00000137968 /// Putative uncharacterized protein SLC44A5 gene:ENSG00000137968 /// Homo sapiens solute carrier family 44, member 5, mRNA (cDNA clone MGC:42574 IMAGE:4824861), complete cds. /// Homo sapiens solute carrier family 44, member 5 (SLC44A5), transcript variant 1, mRNA. /// Homo sapiens solute carrier family 44, member 5 (SLC44A5), transcript variant 2, mRNA.	solute carrier family 44, member 5	0.001243544	-1.364925707
FAM55B	hypothetical protein LOC120406 gene:ENSG00000204361 /// Homo sapiens cDNA FLJ25224 fis, clone STM00905. /// Homo sapiens family with sequence similarity 55, member B (FAM55B), mRNA.	family with sequence similarity 55, member B	0.002821793	-1.396186645
C9orf123	Isoform 2 of Transmembrane protein C9orf123 gene:ENSG00000137038 /// Homo sapiens cDNA FLJ76577 complete cds. /// Homo sapiens chromosome 9 open reading frame 123 (C9orf123), mRNA.	chromosome 9 open reading frame 123	2.58832E-05	1.339680345

MANBAL	Protein MANBAL gene:ENSG00000101363 /// Protein MANBAL gene:ENSG00000101363 /// Mannosidase, beta A, lysosomal-like gene:ENSG00000101363 /// Protein MANBAL gene:ENSG00000101363 /// Protein MANBAL gene:ENSG00000101363 /// Protein MANBAL gene:ENSG00000101363 /// Homo sapiens cDNA: FLJ23055 fis, clone LNG03262. /// Homo sapiens mannosidase, beta A, lysosomal-like (MANBAL), transcript variant 2, mRNA. /// Homo sapiens mannosidase, beta A, lysosomal-like (MANBAL), transcript variant 1, mRNA.	mannosidase, beta A, lysosomal-like	9.61227E-05	1.298557075
C12orf62	Uncharacterized protein C12orf62 gene:ENSG00000178449 /// Homo sapiens chromosome 12 open reading frame 62, mRNA (cDNA clone MGC:14288 IMAGE:4135996), complete cds.	chromosome 12 open reading frame 62	3.32807E-05	1.342134616
C9orf46	Transmembrane protein C9orf46 gene:ENSG00000107020 /// Homo sapiens uncharacterized hematopoietic stem/progenitor cells protein MDS030 mRNA, complete cds. /// Homo sapiens AD025 mRNA, complete cds.	chromosome 9 open reading frame 46	0.003702152	1.261456637
AADACL1	arylacetamide deacetylase-like 1 gene:ENSG00000144959 /// Homo sapiens arylacetamide deacetylase-like 1, mRNA (cDNA clone MGC:33503 IMAGE:4815834), complete cds. /// Homo sapiens arylacetamide deacetylase-like 1 (AADACL1), mRNA.	arylacetamide deacetylase-like 1	0.000709716	-1.22630721

C6orf162	Uncharacterized protein C6orf162 gene:ENSG00000111850 /// Uncharacterized protein C6orf162 gene:ENSG00000111850 /// Homo sapiens chromosome 6 open reading frame 162, mRNA (cDNA clone MGC:88247 IMAGE:6452586), complete cds. /// Homo sapiens chromosome 6 open reading frame 162 (C6orf162), transcript variant 2, mRNA. /// Homo sapiens chromosome 6 open reading frame 162 (C6orf162), transcript variant 1, mRNA.	chromosome 6 open reading frame 162	0.003066727	1.199326745
C3orf57	hypothetical protein gene:ENSG00000179522 /// Protein ADMP gene:ENSG00000196542 /// Homo sapiens cDNA FLJ41354 fis, clone BRAWH2014876. /// Homo sapiens chromosome 3 open reading frame 57 (C3orf57), mRNA.	chromosome 3 open reading frame 57	0.009462343	1.282247476
**Functional Group 51**	**Transcript Description**	**Gene Title**	**Bayes.lnp**	**Fold**
RAB25	Ras-related protein Rab-25 gene:ENSG00000132698 /// Homo sapiens RAB25, member RAS oncogene family, mRNA (cDNA clone MGC:15077 IMAGE:3926839), complete cds. /// Homo sapiens RAB25, member RAS oncogene family (RAB25), mRNA.	RAB25, member RAS oncogene family	0.006083256	1.177304304
RHEB	Homo sapiens ras-related GTP-binding protein mRNA, complete cds. /// Homo sapiens Ras family small GTP binding protein RHEB2 (RHEB2) mRNA, complete cds. /// Homo sapiens cDNA FLJ77896 complete cds, highly similar to Homo sapiens Ras homolog enriched in brain (RHEB), mRNA. /// Homo sapiens Ras homolog enriched in brain, mRNA (cDNA clone MGC:111559 IMAGE:6615350), complete cds. /// Homo sapiens mRNA for ras-related GTP-binding protein, complete cds.	Ras homolog enriched in brain	0.010436696	1.151146911

RAP1A	Ras-related protein Rap-1A gene:ENSG00000116473 /// Ras-related protein Rap-1A gene:ENSG00000116473 /// Homo sapiens RAP1A, member of RAS oncogene family, mRNA (cDNA clone MGC:20027 IMAGE:4422971), complete cds. /// Homo sapiens RAP1A, member of RAS oncogene family (RAP1A), transcript variant 2, mRNA. /// Homo sapiens RAP1A, member of RAS oncogene family (RAP1A), transcript variant 1, mRNA.	RAP1A, member of RAS oncogene family	0.006983909	1.164878638
RRAD	GTP-binding protein RAD gene:ENSG00000166592 /// Homo sapiens Ras-related associated with diabetes, mRNA (cDNA clone MGC:71750 IMAGE:30341066), complete cds. /// Homo sapiens Ras-related associated with diabetes (RRAD), transcript variant 2, mRNA. /// Homo sapiens Ras-related associated with diabetes (RRAD), transcript variant 1, mRNA.	Ras-related associated with diabetes	3.74123E-05	-1.560481717
RAB28	Isoform S of Ras-related protein Rab-28 gene:ENSG00000157869 /// Isoform L of Ras-related protein Rab-28 gene:ENSG00000157869 /// Isoform L of Ras-related protein Rab-28 gene:ENSG00000157869 /// RAB28 protein gene:ENSG00000157869 /// Homo sapiens RAB28, member RAS oncogene family, mRNA (cDNA clone MGC:41862 IMAGE:5263482), complete cds. /// Homo sapiens RAB28, member RAS oncogene family (RAB28), transcript variant 1, mRNA. /// Homo sapiens RAB28, member RAS oncogene family (RAB28), transcript variant 2, mRNA.	RAB28, member RAS oncogene family	0.005673199	1.238421919
RASD1	Dexamethasone-induced Ras-related protein 1 gene:ENSG00000108551 /// Homo sapiens activator of G protein signaling (AGS1) mRNA, complete cds. /// Homo sapiens RAS, dexamethasone-induced 1 (RASD1), mRNA.	RAS, dexamethasone-induced 1	0.006018949	-1.211124367

SAR1B	GTP-binding protein SAR1b gene:ENSG00000152700 /// GTP-binding protein SAR1b gene:ENSG00000152700 /// GTP-binding protein SAR1b gene:ENSG00000152700 /// Homo sapiens GTP binding protein mRNA, complete cds. /// Homo sapiens SAR1 homolog B (S. cerevisiae) (SAR1B), transcript variant 2, mRNA. /// Homo sapiens SAR1 homolog B (S. cerevisiae) (SAR1B), transcript variant 1, mRNA.	SAR1 homolog B (S. cerevisiae)	0.007631315	-1.149369021
GTPBP6	Putative GTP-binding protein 6 gene:ENSG00000178605 /// Homo sapiens cDNA FLJ90047 fis, clone HEMBA1002316, highly similar to GTP-binding protein 6. /// Homo sapiens GTP binding protein 6 (putative) (GTPBP6), mRNA.	GTP binding protein 6 (putative)	0.000608905	1.280156717
ERAL1	Isoform HERA-A of GTP-binding protein era homolog gene:ENSG00000132591 /// Homo sapiens cega mRNA for conserved ERA-like GTPase, complete cds. /// Homo sapiens Era G-protein-like 1 (E. coli) (ERAL1), mRNA.	Era G-protein-like 1 (E. coli)	0.007809797	1.149173663
RHOU	Isoform 1 of Rho-related GTP-binding protein RhoU gene:ENSG00000116574 /// Homo sapiens CDC42-like GTPase (CDC42L1) mRNA, complete cds. /// Homo sapiens ras homolog gene family, member U (RHOU), mRNA.	ras homolog gene family, member U	0.006062205	1.163299791
DYNC1LI1	Cytoplasmic dynein 1 light intermediate chain 1 gene:ENSG00000144635 /// Homo sapiens zinc finger, RAN-binding domain containing 3, mRNA (cDNA clone IMAGE:5575956), complete cds. /// Homo sapiens dynein, cytoplasmic 1, light intermediate chain 1, mRNA (cDNA clone MGC:149683 IMAGE:40117391), complete cds. /// Homo sapiens dynein, cytoplasmic 1, light intermediate chain 1 (DYNC1LI1), mRNA.	zinc finger, RAN-binding domain containing 3 /// dynein, cytoplasmic 1, light intermediate chain 1	0.008493891	1.164885701

Functional Group 52	Transcript Description	Gene Title	Bayes.lnp	Fold
PLEKHG6	Isoform 1 of Pleckstrin homology domain-containing family G member 6 gene:ENSG00000008323 /// Isoform 2 of Pleckstrin homology domain-containing family G member 6 gene:ENSG00000008323 /// Isoform 3 of Pleckstrin homology domain-containing family G member 6 gene:ENSG00000008323 /// Homo sapiens cDNA FLJ10665 fis, clone NT2RP2006200. /// Homo sapiens pleckstrin homology domain containing, family G (with RhoGef domain) member 6 (PLEKHG6), mRNA.	pleckstrin homology domain containing, family G (with RhoGef domain) member 6	0.009513359	1.243652033
TBC1D23	Isoform 2 of TBC1 domain family member 23 gene:ENSG00000036054 /// Isoform 1 of TBC1 domain family member 23 gene:ENSG00000036054 /// Homo sapiens TBC1 domain family, member 23, mRNA (cDNA clone MGC:8800 IMAGE:3847561), complete cds. /// Homo sapiens TBC1 domain family, member 23 (TBC1D23), mRNA.	TBC1 domain family, member 23	1.49374E-05	-1.287480855
PLEKHG1	Pleckstrin homology domain-containing family G member 1 gene:ENSG00000120278 /// Pleckstrin homology domain containing, family G (With RhoGef domain) member 1 gene:ENSG00000120278 /// Pleckstrin homology domain-containing family G member 1 gene:ENSG00000120278 /// Homo sapiens pleckstrin homology domain containing, family G (with RhoGef domain) member 1, mRNA (cDNA clone MGC:176541 IMAGE:9021732), complete cds. /// Homo sapiens pleckstrin homology domain containing, family G (with RhoGef domain) member 1 (PLEKHG1), mRNA.	pleckstrin homology domain containing, family G (with RhoGef domain) member 1	0.002992025	-1.334100959

PSD3	Isoform 3 of PH and SEC7 domain-containing protein 3 gene:ENSG00000156011 /// Isoform 2 of PH and SEC7 domain-containing protein 3 gene:ENSG00000156011 /// Homo sapiens pleckstrin and Sec7 domain containing 3, mRNA (cDNA clone IMAGE:4153308), complete cds. /// Homo sapiens pleckstrin and Sec7 domain containing 3 (PSD3), transcript variant 1, mRNA. /// Homo sapiens pleckstrin and Sec7 domain containing 3 (PSD3), transcript variant 2, mRNA.	pleckstrin and Sec7 domain containing 3	0.856220809	1.010476812
